# H-Bond Templated Oligomer Synthesis Using a Covalent Primer

**DOI:** 10.1021/jacs.2c08119

**Published:** 2022-09-09

**Authors:** Diego Núñez-Villanueva, Christopher A. Hunter

**Affiliations:** Yusuf Hamied Department of Chemistry, University of Cambridge, Cambridge CB2 1EW, United Kingdom

## Abstract

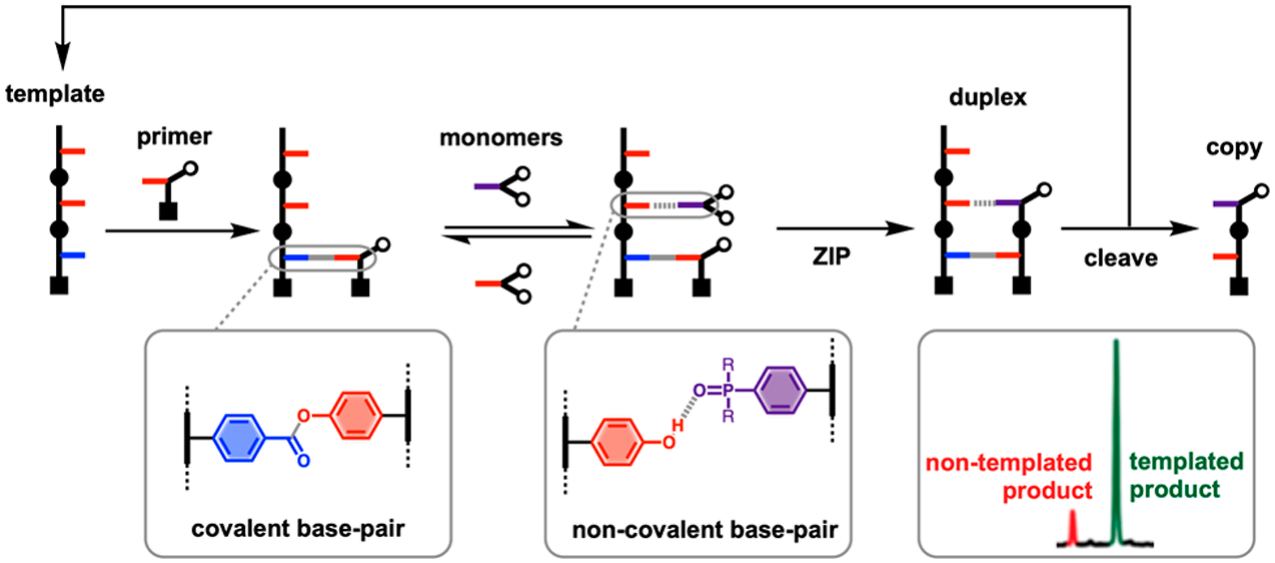

Template-directed synthesis of nucleic acids in the polymerase chain reaction is based on the use of a primer, which is elongated in the replication process. The attachment of a high affinity primer to the end of a template chain has been implemented for templating the synthesis of triazole oligomers. A covalent ester base-pair was used to attach a primer to a mixed sequence template. The resulting primed template has phenol recognition units on the template, which can form noncovalent base-pairs with phosphine oxide monomers via H-bonding, and an alkyne group on the primer, which can react with the azide group on a phosphine oxide monomer. Competition reactions between azides bearing phosphine oxide and phenol recognition groups were used to demonstrate a substantial template effect, due to H-bonding interactions between the phenols on the template and phosphine oxides on the azide. The largest rate acceleration was observed when a phosphine oxide 2-mer was used, because this compound binds to the template with a higher affinity than compounds that can only make one H-bond. The ^31^P NMR spectrum of the product duplex shows that the H-bonds responsible for the template effect are present in the product, and this result indicates that the covalent ester base-pairs and noncovalent H-bonded base-pairs developed here are geometrically compatible. Following the templated reaction, it is possible to regenerate the template and liberate the copy strand by hydrolysis of the ester base-pair used to attach the primer, thus completing a formal replication cycle.

## Introduction

The replication of chemical information is a fundamentally important process, responsible for the origin of life on Earth and the transmission of biological inheritance. Replication constitutes one of the essential processes needed for evolution, along with mutation and selection. Chemists have harnessed the power of evolution for the development of new functional biopolymers and to tailor existing biopolymers for therapeutic and manufacturing applications.^1−3^ These techniques have made a huge impact on industrial bioprocessing, with novel enzymes tuned to catalyze a wide variety of reactions and transformations not accessible to naturally occurring enzymes.^4−7^ Directed evolution relies on the replication and mutation of nucleic acids, so it can only be used to target nucleic acids and proteins.^8,9^ However, there are unexplored regions of chemical space where molecular evolution could have significant impact, for example, synthetic copolymers where function could be encoded by the sequence of different monomer units. The application of molecular evolution principles to synthetic polymers requires a replication method for copying sequence information from a synthetic template to a copy.^10^

**Figure 1:**
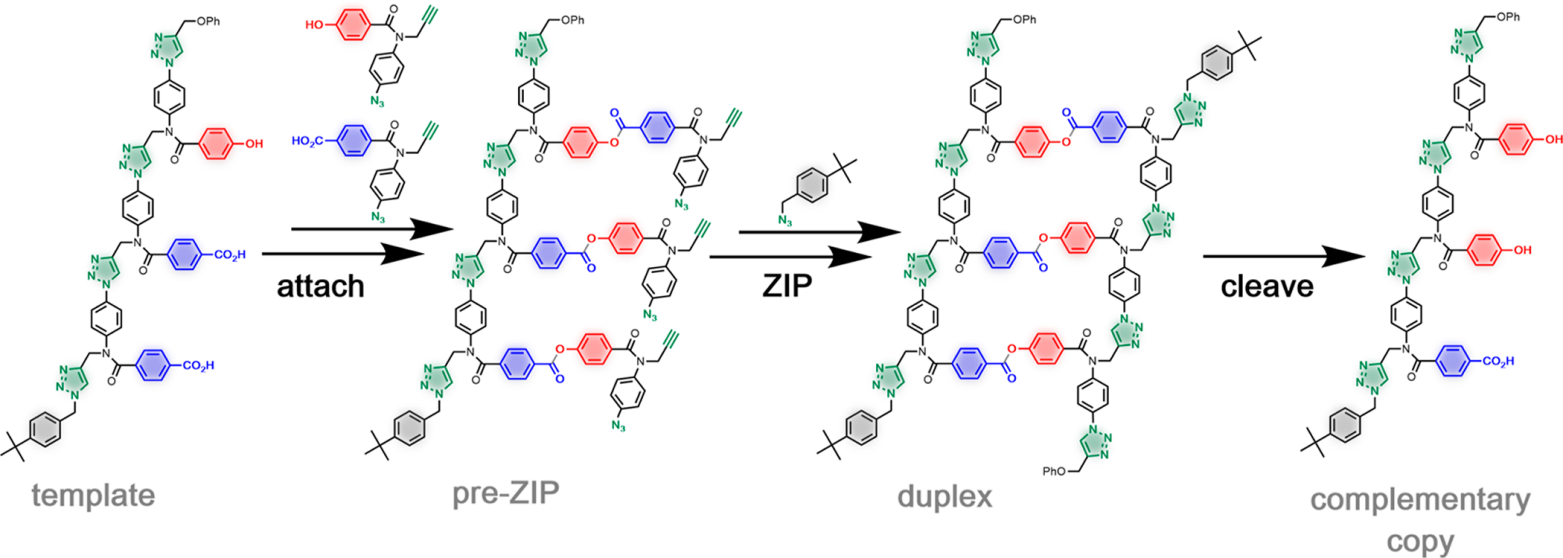
Sequence information transfer using covalent template-directed synthesis. In the attach step, phenol and benzoic acid monomers are coupled with complementary groups on the template using ester base-pairs. In the ZIP step, intramolecular CuAAC reactions lead to oligomerization of monomers on the template, in the presence of an azide chain capping agent. In the cleave step, the ester bonds connecting the daughter strand to the template are broken to regenerate the template and release the complementary copy.

We have developed a method for the replication of sequence information in synthetic oligomers using covalent template-directed synthesis (Figure 1).^11^ This method is based on triazole oligomers where information is encoded as a sequence of phenol and benzoic acid side chains, which can form covalent ester base-pairs. Using a series of orthogonal reactions, phenol protection, ester coupling, phenol deprotection, and ester coupling, the complementary monomers bearing reactive alkyne and azide groups can be efficiently loaded onto the template (attach step in Figure 1). These monomers can then be oligomerized by copper-catalyzed azide alkyne cycloaddition (CuAAC) at high dilution and in the presence of an end-capping group to intercept intermolecular side reactions (ZIP step in Figure 1).^12,13^ Finally, hydrolysis of the ester bonds in the product duplex releases the complementary copy and regenerates the original template, which can be used for further replication cycles. We have also shown that the information transferred in this process can be manipulated by using traceless linkers to attach monomers to the template, enabling direct replication, reciprocal replication, or mutation of information.^14−16^

**Figure 2:**
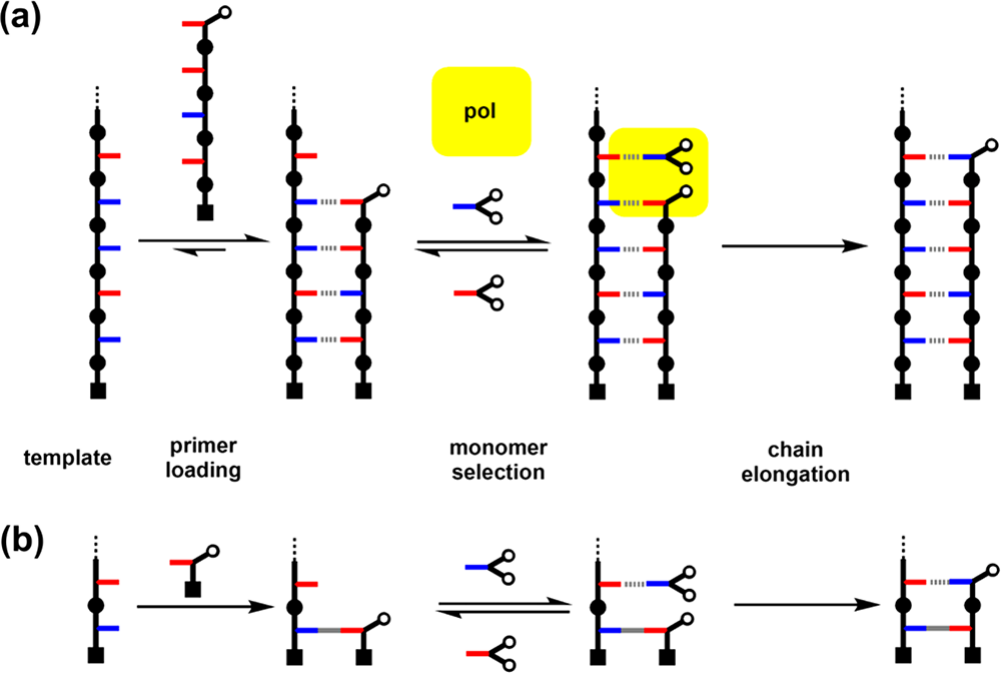
Primer-templated oligomer synthesis. (a) In PCR, a primer anneals with the template to provide a reactive chain end for attachment of the complementary monomer by a polymerase enzyme (pol). (b) In a synthetic system, a covalent base-pair can be used to load the primer, providing a reactive chain end bound to the template. Noncovalent base-pairing can then then be used to select the complementary monomer for chain elongation.

The rationale for the use of kinetically inert covalent base-pairs shown in Figure 1 is that weaker noncovalent base-pairing interactions would lead to inefficient loading of monomers onto the template. Nevertheless, replication of nucleic acids is achieved routinely using noncovalent base-pairs in the polymerase chain reaction (PCR).^17,18^ A key feature of this process is the primer, which anneals with the template in the first step (Figure 2a).^19,20^ Formation of this complex allows binding of the polymerase enzyme, which selects the complementary monomer to be attached to the end of the primer chain. The high affinity of the primer for the template means that only one weak noncovalent interaction is involved in elongation of the chain. Figure 2b illustrates how a similar strategy could be employed in a synthetic system. A covalent base-pair is used to attach a primer to the first base in the template. Then, the formation of a noncovalent base-pair is used to select the complementary monomer for chain elongation.

**Figure 3:**
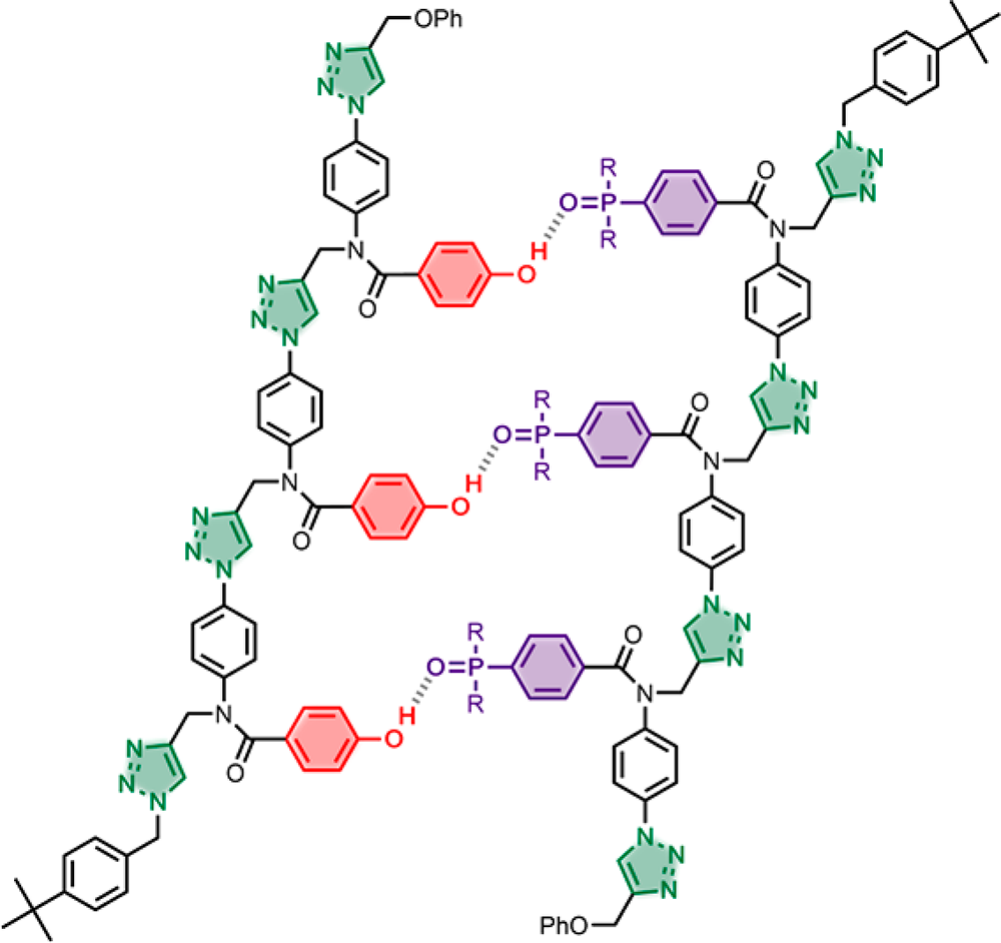
Duplex of two triazole oligomers assembled using noncovalent phenol·phosphine oxide base-pairing interactions.

The approach outlined in Figure 2b requires the development of covalent and noncovalent base-pairs, which have the same geometry, so that both interactions can be accommodated in hybrid duplexes. Figure 3 shows a noncovalent analogue of the covalent duplex formed in the template-directed replication cycle illustrated in Figure 1. The phenol·phosphine oxide base-pair is similar in geometry to the ester base-pair, and we have established previously that the H-bond used in this base-pairing interaction is sufficiently stable to support duplex formation in nonpolar solvents.^21−28^ In this paper, we describe the synthesis of a new family of phenol and phosphine oxide triazole oligomers, demonstrate duplex formation via H-bonding interactions, and show that a covalent base-pair can be used as a primer to direct H-bond template-directed oligomer synthesis.

## Results and Discussion

### H-Bonded Duplexes

**Scheme 1:**
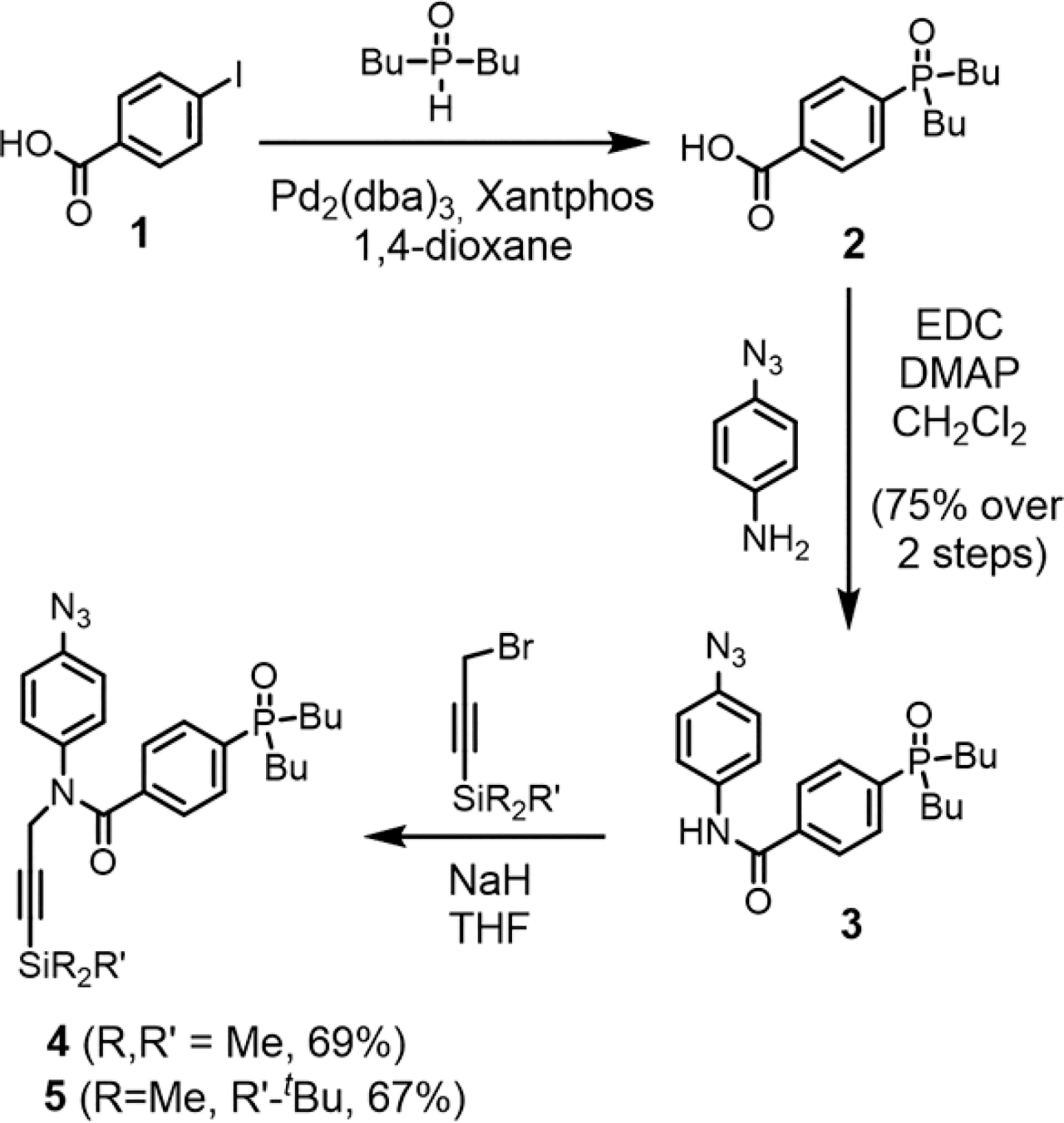
Synthesis of Protected Phosphine Oxide Monomers **4** and **5**

We first studied the ability of the oligotriazole backbone in Figure 1 to support noncovalent duplexes assembled using phenol·phosphine oxide base-pairs. To access homo-oligomers equipped with these recognition units, the corresponding monomers containing an alkyne, an azide, and the recognition module were synthesized. As shown in Scheme 1, the phosphine oxide monomers **4** and **5** were prepared in three steps from iodobenzoic acid **1**. Palladium-mediated P-arylation of **1** with di-*n*-butylphosphine gave **2**, which was subsequently coupled with 4-azidoaniline using EDC. Alkylation of **3** using TMS- or TBDMS-protected propargyl bromide and sodium hydride gave the protected monomers **4** and **5** in good yield. The phenol monomer was prepared according to a procedure previously reported.^11^

**Scheme 2:**
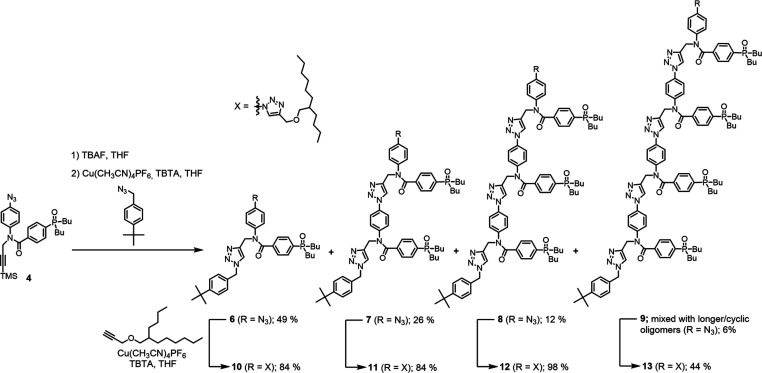
Synthesis of Phosphine Oxide Oligomers **10**–**13**

**Scheme 3:**
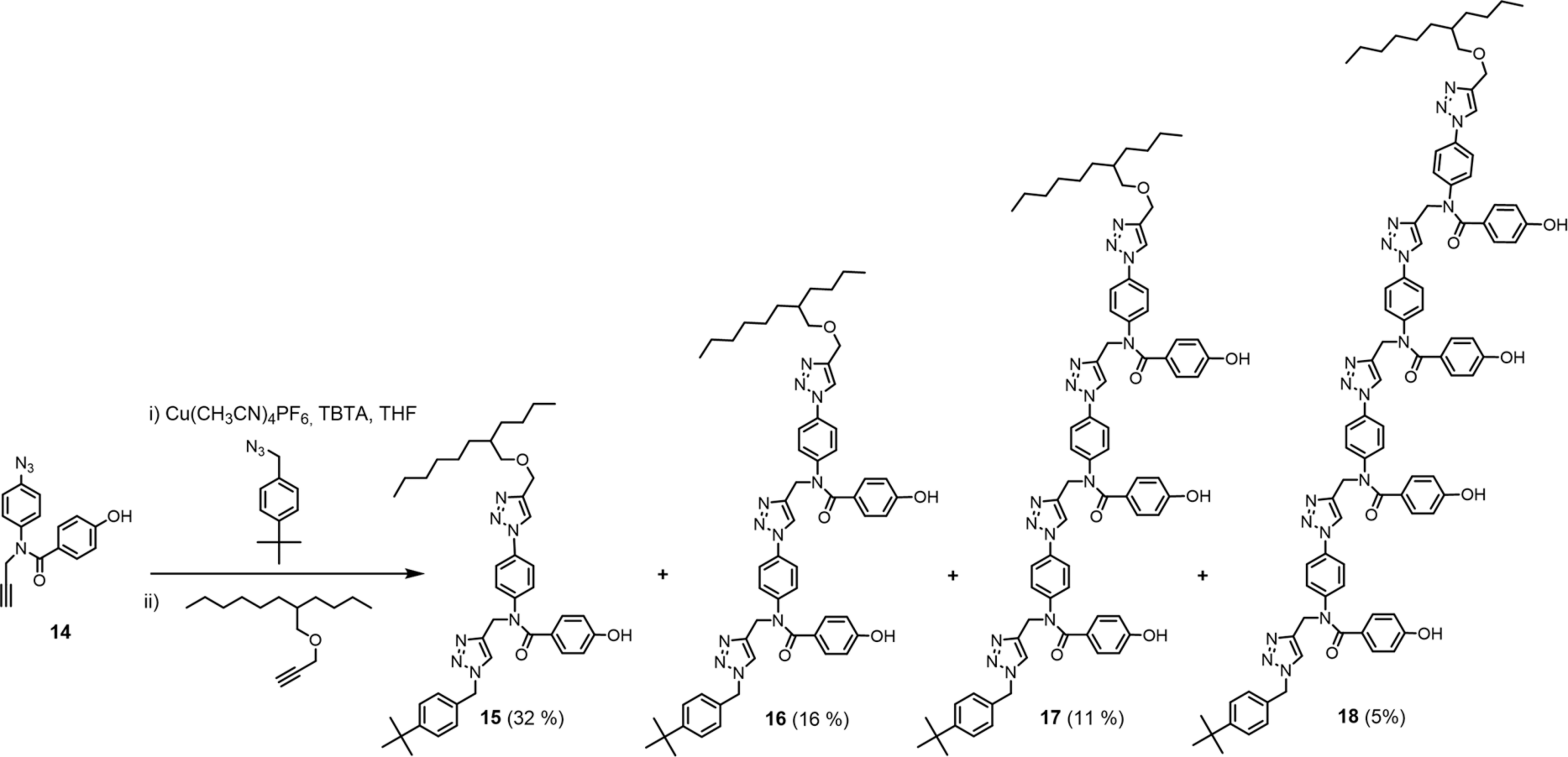
Synthesis of Phenol Oligomers **15**–**18**

CuAAC oligomerization in the presence of an end-capping azide was used to access homo-oligomers up to the 4-mer. For the phosphine oxide oligomers, monomer **4** was first deprotected using TBAF and then oligomerized using Cu(I)-TBTA catalysis in the presence of *p*-*tert*-butylbenzyl azide (Scheme 2). The products **6**–**9** were separated by chromatography and then capped with 2-butyloctyl propargyl ether to give oligomers **10**–**13**. For the phenol oligomers, CuAAC oligomerization of monomer **14** was carried out in the presence of *p*-*tert*-butylbenzyl azide, and then, 2-butyloctyl propargyl ether was added to the reaction mixture along with more Cu(I) catalyst to yield oligomers **15**–**18**, which were separated by chromatography (Scheme 3).

**Scheme 4:**
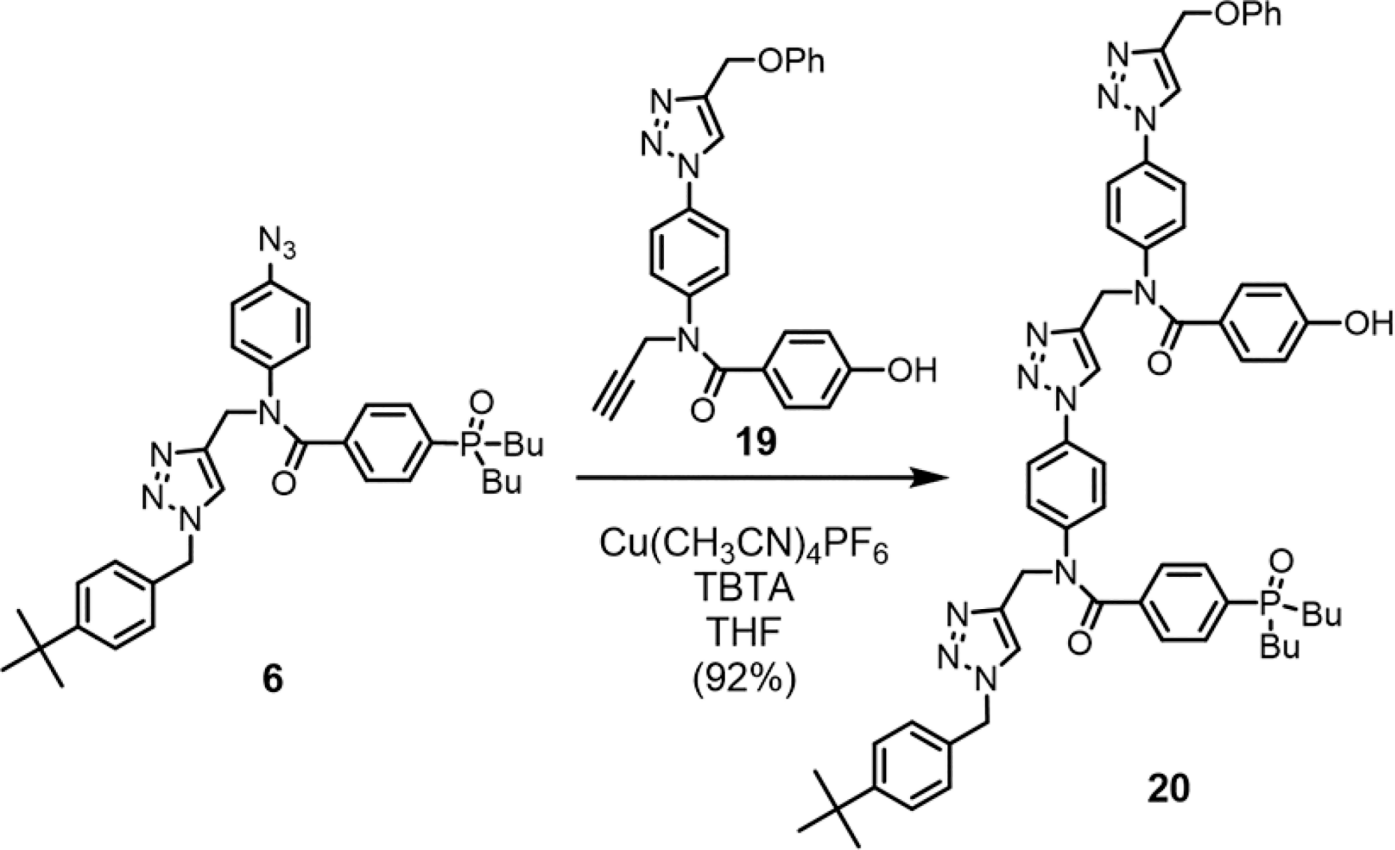
Synthesis of Self-Complementary 2-Mer **20**

In addition to the homo-oligomers, the self-complementary 2-mer **20** was prepared by CuAAC coupling of **6** with the previously reported phenol **19**(11) (Scheme 4).

**Figure 4:**
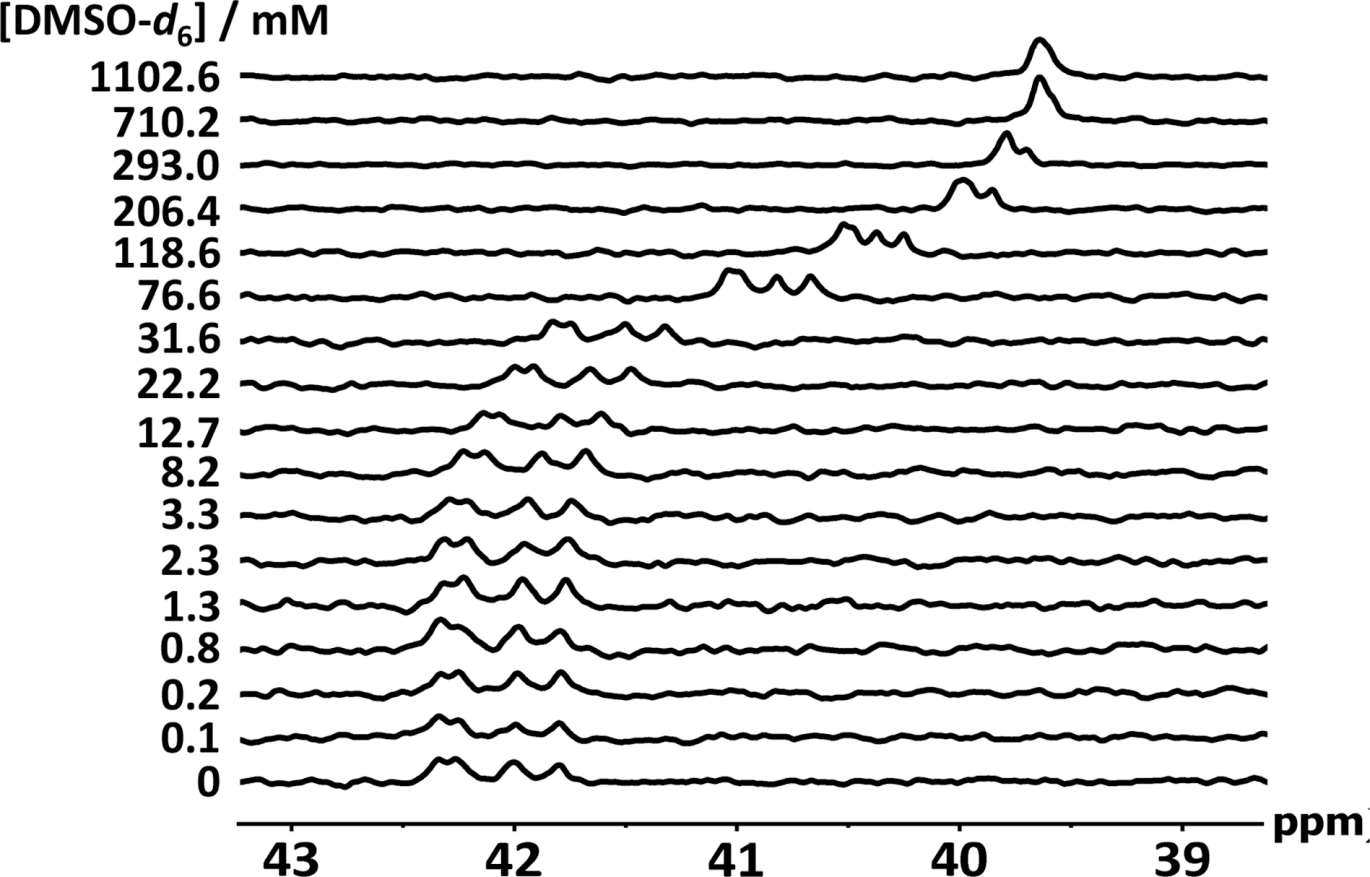
^31^P NMR spectra (202 MHz) for DMSO-*d*_6_ denaturation of an equimolar solution of DDDD and AAAA (1 mM) in CD_2_Cl_2_ at 298 K.

^31^P NMR titrations and DMSO denaturation experiments were carried out to characterize duplex formation between length-complementary homo-oligomers. Figure 4 shows the ^31^P NMR spectra of a 1:1 mixture of AAAA (**13**) and DDDD (**18**) in CD_2_Cl_2_. In the absence of DMSO, four signals due to the four nonequivalent phosphine oxide groups were observed at chemical shifts of around 42 ppm. When DMSO was added, upfield shifts of 2 ppm were observed for each of these signals. These results indicate that all four phosphine oxide groups of AAAA are involved in intermolecular H-bonding interactions with complementary phenol groups on the DDDD oligomer. The ddition of DMSO denatures the duplex, and all four ^31^P signals have the same chemical shift (39.5 ppm) at the end of the experiment, which indicates that the H-bonding interactions have been disrupted. Similar results were obtained for the AA·DD and AAA·DDD duplexes (see the Supporting Information (SI) for details).

**Figure 5:**
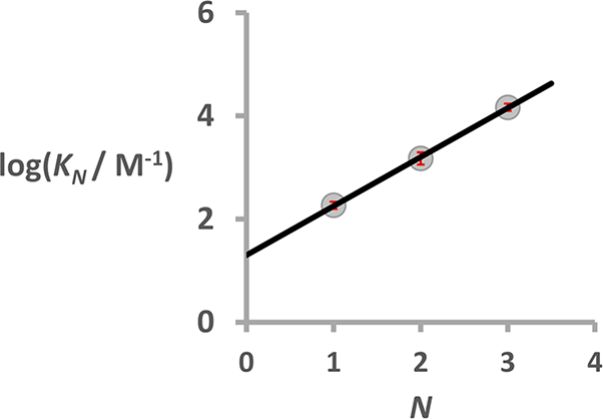
Association constants (*K*_*N*_) for duplex formation in CD_2_Cl_2_ at 298 K plotted as a function of the number of recognition modules in the oligomer, *N*. The best fit straight line is shown (log*K*_*N*_ = 0.95 *N* + 1.30; *R*^2^ = 0.999).

^31^P NMR titrations were used to measure association constants for the single base-pair formed in the A·D complex and for the AA·DD and AAA·DDD duplexes (see the SI for details). Self-association of the self-complementary 2-mer AD was also investigated using ^1^H and ^31^P NMR dilution experiments. The results are summarized in Table 1. The association constant (*K*_*N*_) increases by an order of magnitude for every recognition module added to the oligomer (Figure 5). In all cases, complexation is associated with a large downfield change in chemical shift for each of the ^31^P NMR signals (+3–5 ppm). These results suggest that the length-complementary oligomers form duplexes with all of the recognition modules involved in cooperative H-bonding interactions, as illustrated in Figure 3. The association constants in Table 1 can used to determine the effective molarity for the intramolecular H-bonding interactions that lead to duplex assembly. Equation 1 shows the relationship between the overall association constant for duplex formation between two homo-oligomers of length *N*, the association constant for formation of a single intermolecular H-bond (*K*) and the effective molarity (EM).
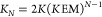
1

**Table 1:** Association Constants (*K*_*N*_), Effective Molarities (EM), and Limiting ^31^P NMR Chemical Shifts (ppm) for the Formation of Duplexes Measured by NMR Titrations in CD_2_Cl_2_ at 298 .^a^

complex		Log(*K*_*N*_/M^–1^)	EM/mM	*K*·EM	δ_free_/ppm	δ_bound_/ppm
A·D	(**10**·**15**)	2.3 ± 0.1			39.1 ± 0.1	44.2 ± 0.1
AA·DD	(**11**·**16**)	3.2 ± 0.1	22 ± 9	4 ± 1	38.9 ± 0.1	44.2 ± 0.1
AD·AD	(**20**·**20**)	3.2 ± 0.1	25 ± 8	5 ± 1	39.7 ± 0.1	42.9 ± 0.1
AAA·DDD	(**12**·**17**)	4.2 ± 0.1	34 ± 9	6 ± 1	38.9 ± 0.2	43.3 ± 0.4

a Each titration was repeated twice, and the average value is reported with errors at the 95% confidence limit.

The values of EM and *K*·EM calculated using eq 1 are reported in Table 1. The value of EM (20–30 mM) is similar to the values we have reported previously for a number of different H-bonded duplex architectures.^21−28^ The product *K*·EM measures the chelate cooperativity associated with duplex formation, and the value is significantly greater than one for this system, which is indicative of a fully assembled duplex.

### Template Effects on CuAAC Reactions

**Figure 6:**
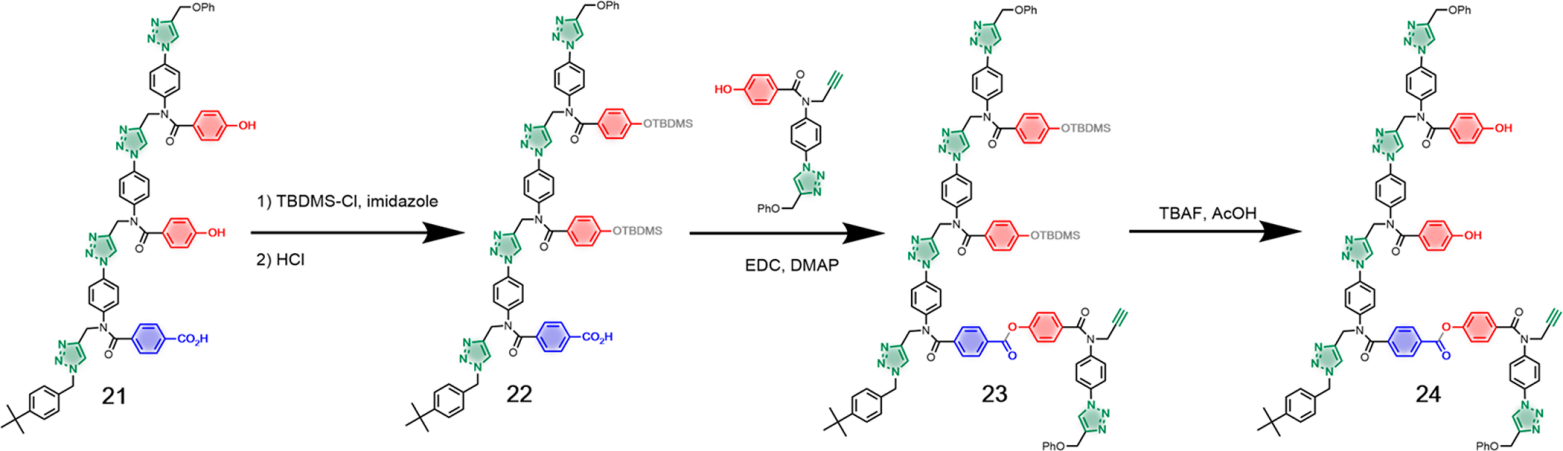
Protection-coupling-deprotection sequence of reactions used to load covalent primer **19** onto template **21**.

The template used to test the covalent primer approach is shown in Figure 6. The terminal benzoic acid unit was used as the point of attachment of the covalent primer, and the two phenol units serve as noncovalent bases that can form H-bonds with phosphine oxide monomers. The synthesis of template **21** and primer **19** was described previously.^11^ Primer **19** was loaded onto template **21** using a series of protection-coupling-deprotection reactions. Selective protection of the phenol bases afforded **22**, which was coupled with **19** using EDC. TBAF-mediated deprotection of the phenol bases yielded the primed template **24**. In each of these reactions, quantitative conversion of starting material to product was achieved with aqueous workup as the only purification required (see Figure S13, SI). Column chromatography after the final step removed the excess of primer **19** used in the attach step.

**Figure 7:**
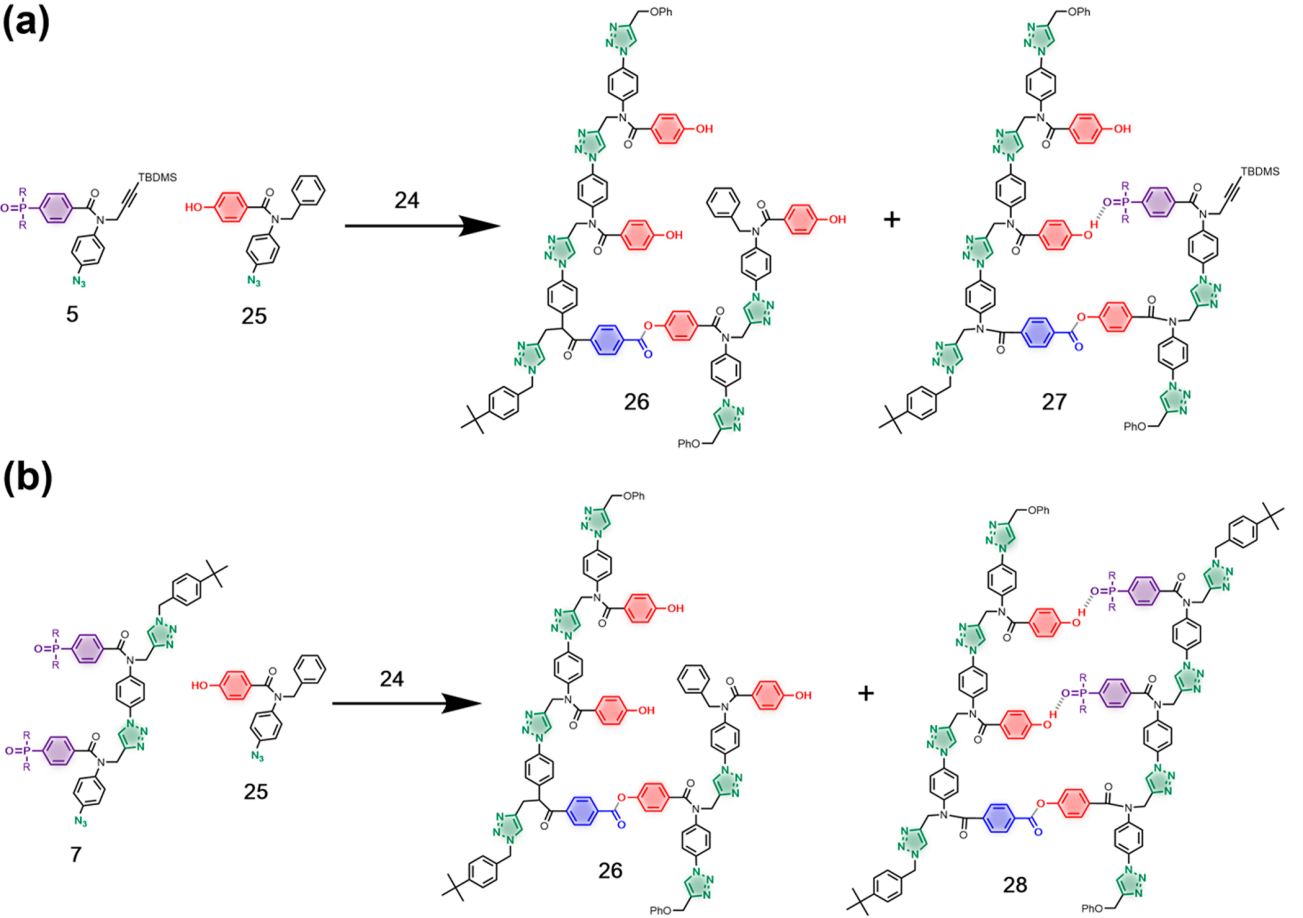
Competition CuAAC reactions used to quantify template effects. Reaction of primer-loaded template **24** with (a) a mixture of phenol and phosphine oxide monomers and (b) a mixture of phenol monomer and phosphine oxide 2-mer (R = Bu).

**Figure 8:**
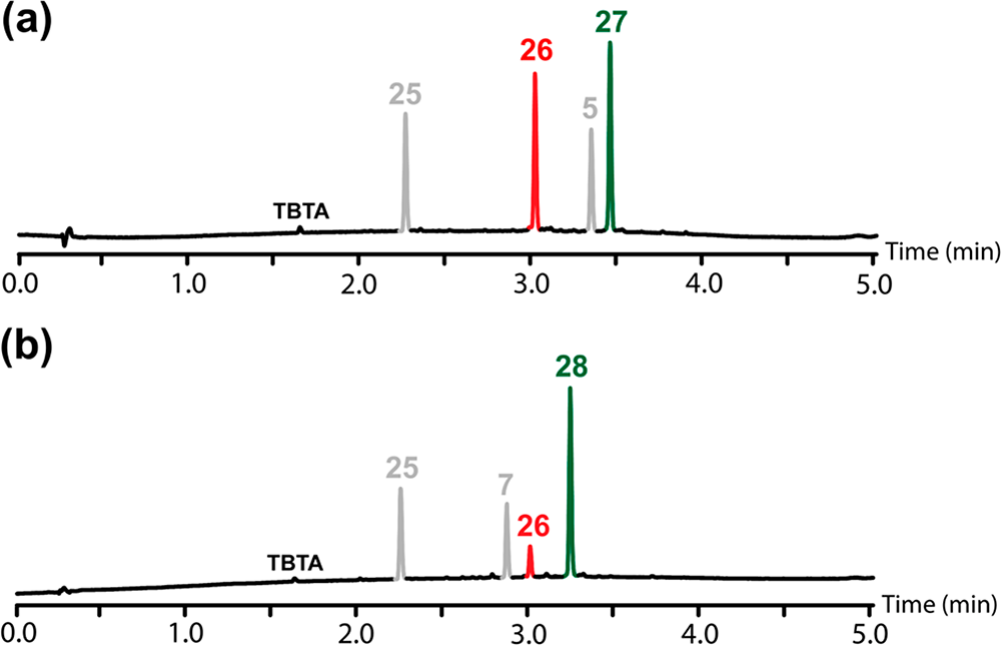
UPLC traces of crude reaction mixtures from the competition experiments shown in Figure 7 using 1:1 mixtures of azides: (a) phenol monomer **25** and phosphine oxide monomer **5** and (b) phenol monomer **25** and phosphine oxide 2-mer **7**. Reaction conditions: [**24**] = 0.1 mM, [**25**] = 0.15 mM, [**5**] or [**7**] = 0.15 mM, [Cu(CH_3_CN)_4_PF_6_·TBTA] = 0.2 mM in CH_2_Cl_2_, stirring at room temperature for 48 h. See Figure 7 for the chemical structures. UPLC conditions: C18 column at 40 °C (254 nm) using water + 0.1% formic acid (A) and CH_3_CN + 0.1% formic acid (B); Gradient of 0–4 min 5% −100% B + 1 min 100% B.

The primed template **24** was then used to carry out noncovalent template-directed synthesis of oligomers by CuAAC. The template effect was investigated using a competition reaction between two different azide monomers: **5** is equipped with a phosphine oxide recognition unit and can base-pair with the phenol recognition modules on the template; **25** is equipped with a phenol recognition unit, which cannot base-pair with the template (Figure 7a). Two equivalents of each azide monomer were added to the primed template, and the reaction was initiated by adding Cu(CH_3_CN)_4_PF_6_ and TBTA. The reaction was monitored by UPLC, and Figure 8a shows the UPLC trace after all of **24** had been consumed. There are no significant differences between the amounts of templated product (**27**, green peak) and nontemplated product (**26**, red peak) formed, and similar amounts of the two azide starting materials remain (gray peaks). In order to increase the binding affinity with the template, a second templating experiment was carried out using the phosphine oxide 2-mer **7** instead of monomer **5** (Figure 7b). Table 1 indicates that the affinity of the 2-mer for the template will be an order of magnitude higher than the monomer, and the increase in association constant should improve the template effect. Figure 8b shows the UPLC trace for the competition reaction between **7** and **25** after all of the primed template **24** had been consumed. In this case, a significant template effect is observed, and the templated product (**28**, green peak) is formed preferentially compared with the nontemplated product (**26**, red peak).

**Figure 9:**
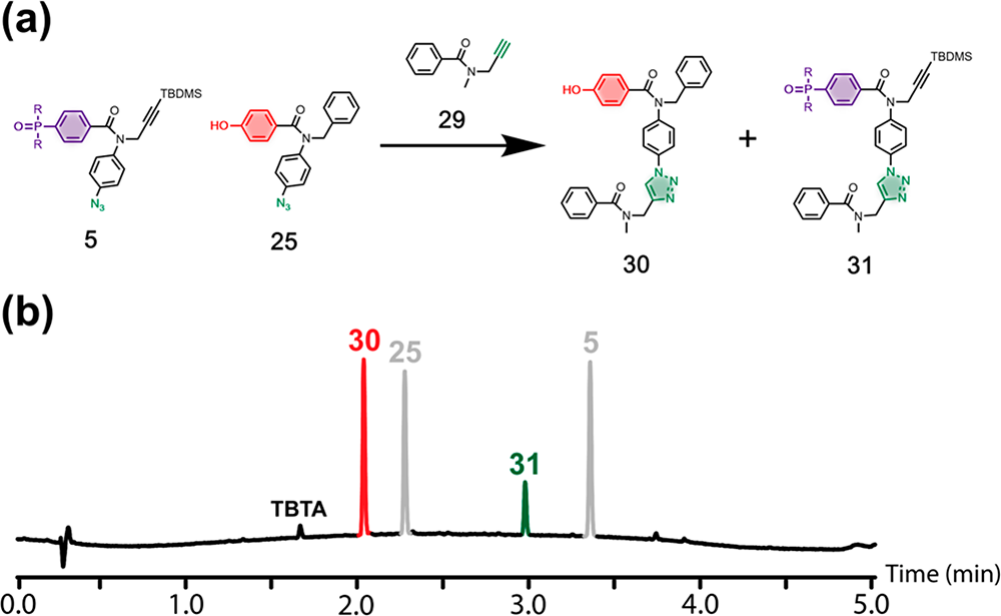
(a) Competition experiment used to quantify the relative reactivity of azides **25** and **5** in CuAAC reactions (R = Bu). (b) UPLC trace of the crude reaction mixture. Reaction conditions: [**29**] = 0.1 mM, [**25**] = 0.15 mM, [**5**] = 0.15 mM, [Cu(CH_3_CN)_4_PF_6_·TBTA] = 0.2 mM in CH_2_Cl_2_, stirring at room temperature for 48 h. UPLC conditions: C18 column at 40 °C (254 nm) using water + 0.1% formic acid (A) and CH_3_CN + 0.1% formic acid (B); Gradient of 0–4 min 5% −100% B + 1 min 100% B.

In order to quantify the template effects in Figure 8, control reactions were carried out using a simple alkyne in place of the primed template **24** (Figure 9a). Figure 9b shows the UPLC trace for the competition reaction between azides **5** and **25** after all of alkyne **29** had been consumed. The phenol monomer is clearly significantly more reactive than the phosphine oxide monomer, because the yield of **30** (red peak) is more than double the yield of **31** (green peak). This result suggests that the appearance of UPLC trace shown in Figure 8a masks a substantial template effect for the reaction with the phosphine oxide monomer **5**. Similar results were obtained for the competition reaction between azides **7** and **25** for alkyne **29** (see Figure S15, SI). The azide equipped with a phenol group is intrinsically more reactive than the azides equipped with phosphine oxides.

**Figure 10:**
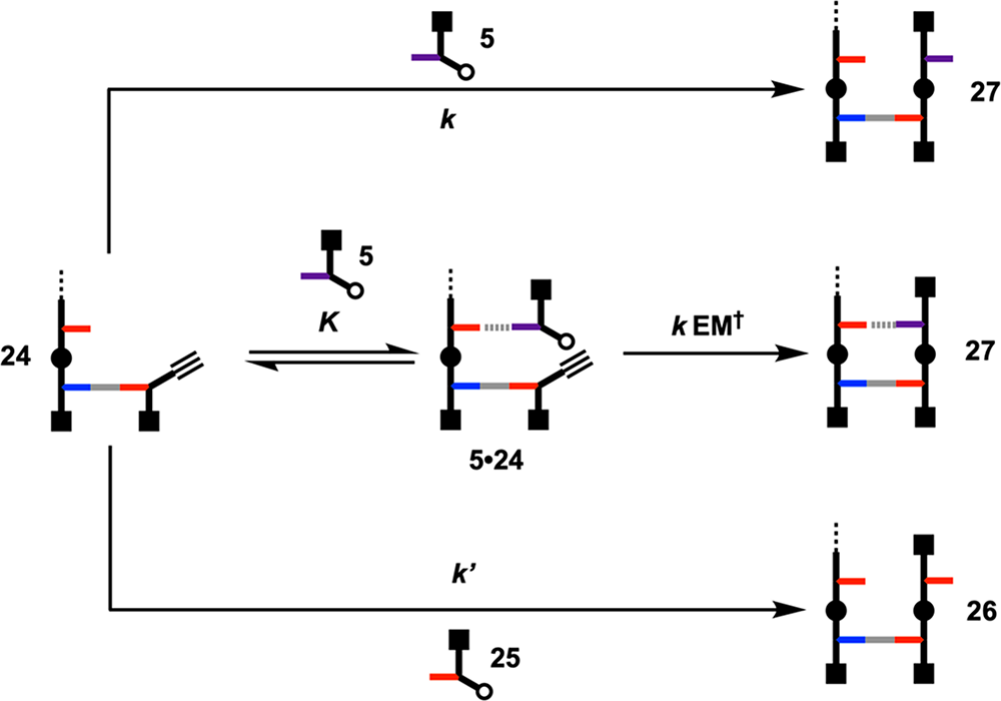
Different reaction pathways in a competition reaction between **5** and **25** for primed template **24**. *k* and *k*′ are the rate constants for the nontemplated pathways, *K* is the association constant for formation of the **5**·**24** complex, and EM^†^ is the kinetic effective molarity for the templated reaction.

Figure 10 shows the templated and nontemplated reaction pathways that are possible in the competition experiments. The relative rates of these processes can be determined from the product distributions, which can be quantified by integrating the peaks in the UPLC traces and correcting for differences in molar extinction coefficients (see the SI for details). Since the azides are present in excess, the ratio of the rate constants for the two nontemplated pathways can be estimated from the product distribution in the control experiment shown in Figure 9. For the competition experiment with the phosphine oxide monomer **5**:
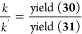
2

The rate acceleration for the templated reaction can be estimated from the product distribution in the reaction shown in Figure 8. For the templated pathway in the reaction between **5** and **24**, the rate is given by eq 3.
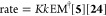
3where *K* is the association constant for formation of the H-bonded complex and EM^†^ is the kinetic effective molarity for the intramolecular reaction that takes place within this complex.

The rate of the nontemplated pathway is given by eq 4.
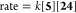
4

Since both pathways that lead to product **27**, the product distribution is given by eq 5.
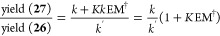
5

Equation 5 shows that the rate acceleration associated with the templated pathway is the product of the association constant for binding of the phosphine oxide to the template *K* and the kinetic effective molarity for the templated reaction EM^†^. Since the ratio *k*/*k*′ can be determined from the control experiment and the value of *K* is known from the NMR titrations reported in Table 1, the product distribution of the templated reaction can be used to determine EM^†^. For the **5**·**24** complex, the rate acceleration (*K* EM^†^) was found to be 1.2 ± 0.7 (Table S1, SI). This result means that the reaction on the template proceeds at the same rate as the nontemplated reaction, so the rate of formation of the templated product is doubled in the presence of the template. However, the templated and nontemplated products are formed in similar proportions, because the intrinsic reactivity of the phenol monomer **25** is roughly twice that of the phosphine oxide **5** (Table S1, SI). The value of EM^†^ for the **5**·**24** complex is 6 mM, which is slightly lower than the thermodynamic effective molarities measured for the H-bonded duplexes but of a similar order. This result implies that there is good geometric complementarity between the covalent ester base-pairs and the noncovalent phenol·phosphine oxide base-pairs.

A similar analysis can be applied to the templated reaction between **24** and the 2-mer phosphine oxide **7**. In this case, the rate acceleration in the **24**·**7** complex (*K* EM^†^) was found to be 5.2 ± 0.7 (Table S2, SI). Again the intrinsic reactivity of **25** is roughly twice that of **7**, but the rate acceleration in the templated pathway is now large enough to override this effect. The value of EM^†^ for the **7**·**24** complex is 3 mM, which is similar to the value measured for the **5**·**24** complex, so the origin of the enhanced rate acceleration in this system clearly lies in the higher binding affinity of the 2-mer phosphine oxide for the template (cf eq 5).

**Figure 11:**
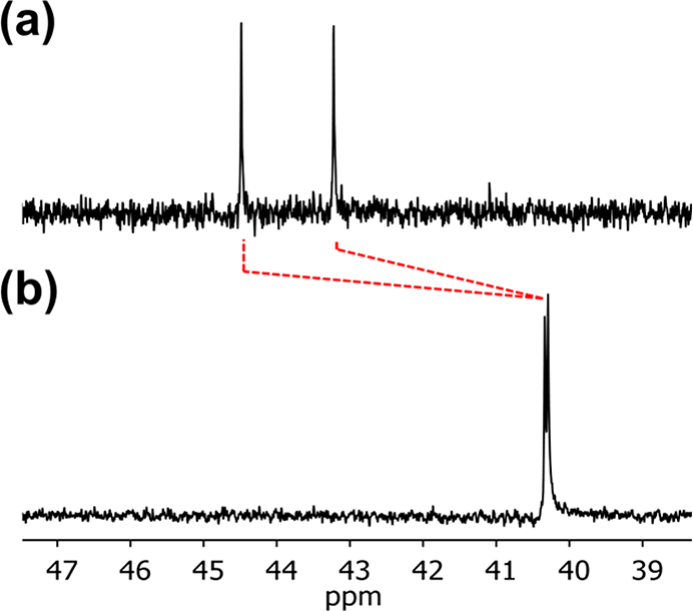
^31^P NMR spectra (500 MHz) recorded in CDCl_3_ at 298 K of (a) product duplex **28** (0.6 mM) and (b) the phosphine oxide 2-mer **7** (3.1 mM).

Evidence for the H-bonding interactions used to template the CuAAC reaction can be found in the ^31^P NMR spectrum of the product **28** (Figure 11). Compared with the ^31^P NMR spectrum of **7**, there is a significant downfield shift for both ^31^P signals (+3–4 ppm), which indicates that both phosphine oxide groups make intramolecular H-bonding interactions with the complementary phenol recognition units in the product duplex (cf titration data in Table 1). A similar downfield shift was observed for the templated product **27**, which contains a single phosphine oxide group (Figure S16). The fact that the noncovalent base-pairs are fully assembled in the product duplexes confirms that there is good geometric compatibility with the covalent ester base-pair.

### Template-Directed Oligomer Synthesis

**Figure 12:**
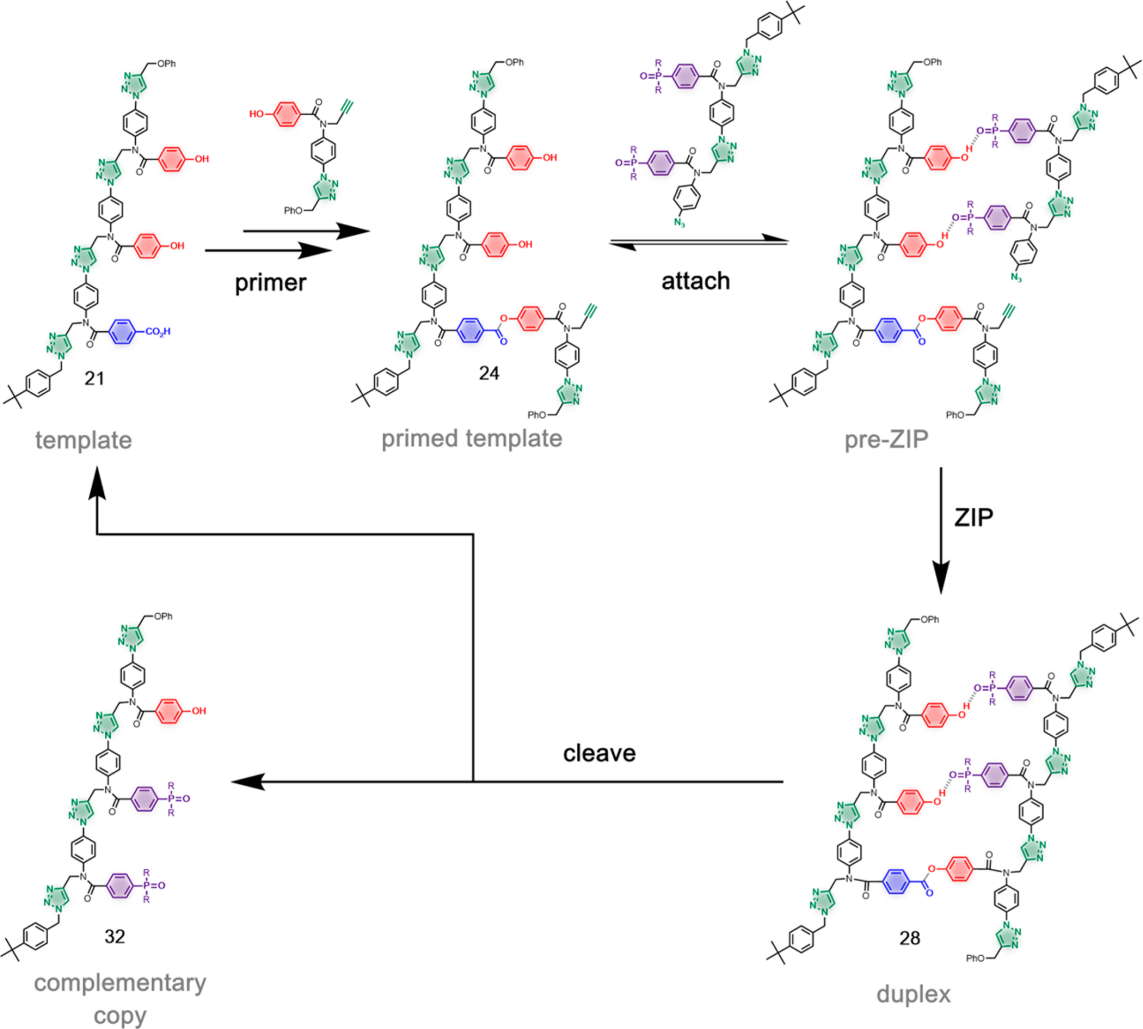
H-bond template-directed oligomer synthesis using a covalent primer. Covalent primer **19** was first loaded onto template **21** using the reaction sequence in Figure 6. H-bonding interactions between **24** and **7** lead to selective formation of duplex **28** in a CuAAC reaction. Hydrolysis of the ester base-pair gave the copy **32** and regenerated template **21**.

**Figure 13:**
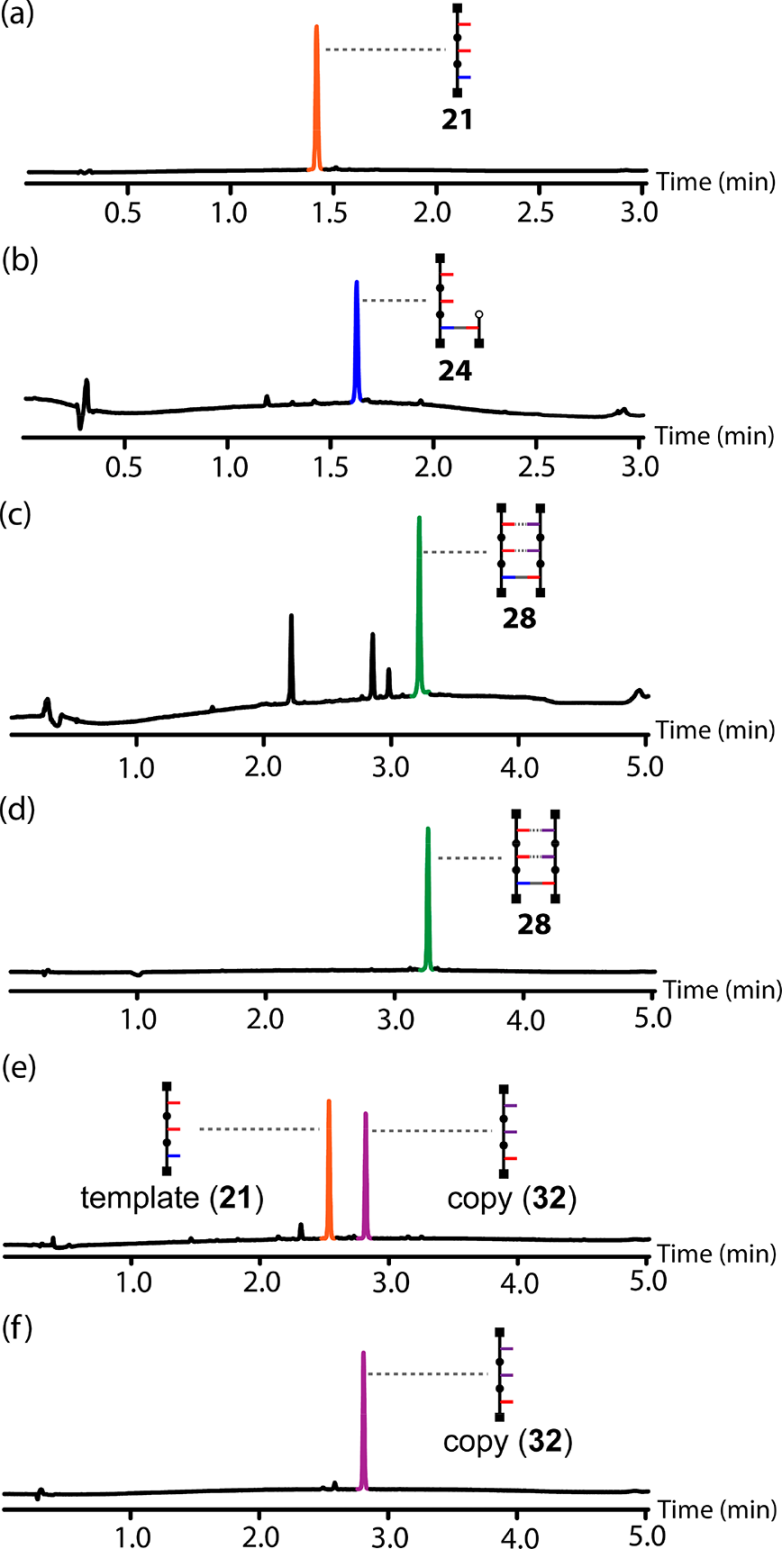
UPLC traces for H-bond template-directed oligomer synthesis using a covalent primer: (a) starting template **21**; (b) crude reaction mixture after primer loading (**24**); (c) crude reaction mixture obtained after the CuAAC reaction of **24** in the presence of equimolar amounts of **7** and **25** (the additional peaks correspond to unreacted **7** and **25** and the competition product **26**); (d) isolated duplex **28**; (e) crude reaction mixture obtained after hydrolysis of the ester base-pair; (f) isolated copy **32**. UPLC conditions: C18 column at 40 °C (254 nm) using water + 0.1% formic acid (A) and CH_3_CN + 0.1% formic acid (B); Gradient of 0–2 min 5% −100% B + 1 min 100% B for a and b and gradient of 0–4 min 5% −100% B + 1 min 100% B for c–f.

These results show that the 2-mer phosphine oxide maximizes the template effect on the CuAAC reaction, due to the higher association constant associated with the formation of two H-bonded base-pairs. This system was therefore used to conduct a complete replication cycle using template **21** and a covalent primer (Figure 12). In the first step, the covalent primer **19** was loaded onto the template. Figure 13a shows the UPLC trace of the starting template **21**, and Figure 13b shows the corresponding trace of the crude product mixture obtained after the sequence of protection-coupling-deprotection reactions shown in Figure 6. The only significant impurity detected in addition to the primed template **24** was the slight excess of **19** used in the coupling step. The template-directed CuAAC reaction was then carried out using a mixture of the complementary phosphine oxide 2-mer **7** and a competing phenol monomer **25**. Duplex **28** was obtained as the major product (Figure 13c) and isolated from the mixture by column chromatography (Figure 13d). Finally, cleavage of the ester base-pair regenerated the initial template **21** and released the copy **32** (Figure 13e), which was isolated (Figure 13f) and fully characterized (see the SI).

## Conclusions

Templated-directed synthesis of synthetic copolymer of defined sequence would open the way for the synthesis and evolution of sequence polymers that could rival the functional properties of biopolymers. We have previously developed templating methods that use covalent base-pairing interactions to attach monomer building blocks to a template and promote a CuAAC oligomerization reaction, where the sequence of the product is defined by the sequence of the template. Here, we expand the scope to include noncovalent interactions to bind monomer building blocks to a template. H-bonding between phenol and phosphine oxide side-chains was used as the noncovalent base-pairing interaction to attach monomers to a template oligomer. Ester formation between phenol and carboxylic acid side-chains was used a covalent base-pairing interaction to attach a primer to the template and ensure that templated processes were favored relative to off-template processes.

Azide–alkyne monomers equipped with either phenol or phosphine oxide recognitions were synthesized. CuAAC oligomerization reactions were then used to obtain families of homo-oligomers equipped with H-bonding recognition groups as the side chains. Length complementary phenol and phosphine oxide oligomers form duplexes in dichloromethane solution, and the stability of the duplex increases by an order of magnitude with each base-pair added to the chain. These experiments show that the triazole backbone is compatible with the noncovalent base-pairing motif and that this system is suitable for investigation of template-directed oligomer synthesis.

A covalent primer was attached to a mixed sequence template by formation of an ester base-pair with the terminal base on the template. The resulting primed template has two phenol recognition sites and an alkyne group ready for chain extension via CuAAC with building blocks equipped with azide groups. Competition reactions were used to show that azides equipped with phosphine oxide recognition units react more rapidly with the primer than azides equipped with phenol recognition units. A rate acceleration of a factor of 2 was observed for a phosphine oxide monomer, which binds the template with a relatively low affinity. A much higher rate acceleration was observed when a phosphine oxide 2-mer was used (factor of 6), because this compound binds the template with an order of magnitude higher affinity than the corresponding monomer. This system was used to carry out a complete cycle of templated-directed synthesis, starting from the mixed sequence template, loading the primer, followed by templated oligomer synthesis and then cleavage of the product duplex by ester hydrolysis to regenerate the template and release the sequence-complementary copy oligomer. The results show that the covalent and noncovalent base-pairing motifs developed here are mutually compatible, and in combination, these motifs provide a new primer-based approach to templated oligomer synthesis. The observation that a 2-mer building block leads to a higher rate acceleration compared with the monomer is reminiscent of nonenzymatic RNA templated reactions, where the rate and fidelity of primer extension is better with 2-mers than with monomers.^29^

## Supporting Information Available

Discussions of general experimental details, synthesis and characterization of building blocks, and binding studies, schemes of synthesis routes and reaction schemes, figures of NMR spectra, UPLC traces and MS spectra for the control reactions and templated oligomer synthesis, duplex denaturation data, and molecular models from a MacroModel conformational search, and tables of peak areas from the UPLC chromatograms (PDF)
