# Supplementary material for: H-Bond Templated Oligomer Synthesis Using a Covalent Primer
Source: J Am Chem Soc. 2022 Sep 9;144(37):17307–16. doi: 10.1021/jacs.2c08119 (PMC9501907; doi:10.1021/jacs.2c08119)
Supplement: Supplementary file 1 — ja2c08119_si_001.pdf [file ja2c08119_si_001.pdf]

Supporting information

H-bond templated oligomer synthesis using a covalent primer

Diego Núñez-Villanueva, Christopher A. Hunter*

Yusuf Hamied Department of Chemistry, University of Cambridge, Lensfield Road, Cambridge CB2 1EW, UK. E-mail: herchelsmith.orgchem@ch.cam.ac.uk

TABLE OF CONTENTS **Page**

1. General experimental details. S2

2. Synthesis and characterization of building blocks S3

3. Synthesis and characterization of oligomers S30

4. Binding studies S81

5. Primer loading S88

6. Template-directed synthesis S95

7. Hydrolysis of duplex 28 providing copy 32 S117

8. References S122

1. General experimental details.

All the reagents and materials used in the synthesis of the compounds described below were bought from commercial sources, without prior purification. Dry THF and CH_2Cl_2 were obtained from a solvent purification system (Pure Solv™, Innovative Technology, Inc.). Anhydrous DMF was purchased from Sigma-Aldrich. Thin layer chromatography was carried out using with silica gel 60F (Merck) on glass plates. Flash chromatography was carried out on an automated system (Combiflash Rf+ or Combiflash Rf Lumen) using prepacked cartridges of silica (25µ PuriFlash® columns). All NMR spectroscopy was carried out on a Bruker 400 MHz DPX400, 400 MHz AVIII400, 500 MHz DCH cryoprobe or 500 MHz TCI Cryoprobe spectrometer using the residual solvent as the internal standard. All chemical shifts (δ) are quoted in ppm and coupling constants given in Hz. Splitting patterns are given as follows: s (singlet), bs (broad singlet), d (doublet), t (triplet), q (quadruplet), m (multiplet). FT-IR spectra were measured on a Bruker Alpha spectrometer equipped with an ATR cell. UPLC analysis of samples was performed using Waters Acquity H-class UPLC coupled with a single quadrupole Waters SQD2. Acquity UPLC CSH C18 column, 130 Å, 1.7 µm, 2.1 mm x 50 mm or Acquity UPLC BEH C8 column, 130 Å, 1.7 µm, 2.1 mm x 50 mm were used as UPLC columns. The conditions of the UPLC method are as follows: gradients of water + 0.1% formic acid (solvent A) and acetonitrile + 0.1% formic acid (solvent B) as specified in each case. Flow rate: 0.6 ml/min; Column temperature of 40°C; Injection volume of 2 µL. The signal was monitored at 254 nm. HRMS analysis was performed in an Agilent walk up 6230 LC/TOF using a gradient from 5 to 100% of acetonitrile (0.25% formic acid) in water (0.25% formic acid) over 6 minutes.

2. Synthesis and characterization of building blocks

2.1. Synthesis of phosphine oxide building blocks

As shown in Scheme S1, phosphine oxide derivatives **4** and **5** were prepared from 4-iodobenzoic acid (**1**). First, palladium-mediated P-arylation of **1** with di-*n*-butylphosphine oxide gave **2**, which was used without further purification after work-up in the coupling reaction with 4-azidoaniline using EDC providing **3** in good yield over the two steps. Finally, **3** was subjected to alkylation using either TMS- or TBDMS-protected propargyl bromide, the later (**S2**) synthesized from propargyl bromide using BuLi and TBDMS-Cl. From commercial TMS-protected propargyl bromide, the alkylation reaction afforded TMS-protected 1-mer **4** in good yield along with the corresponding deprotected 1-mer **S1**. Similarly, from bromide **S2**, TBDMS-protected 1-mer **5** was obtained in good yield.

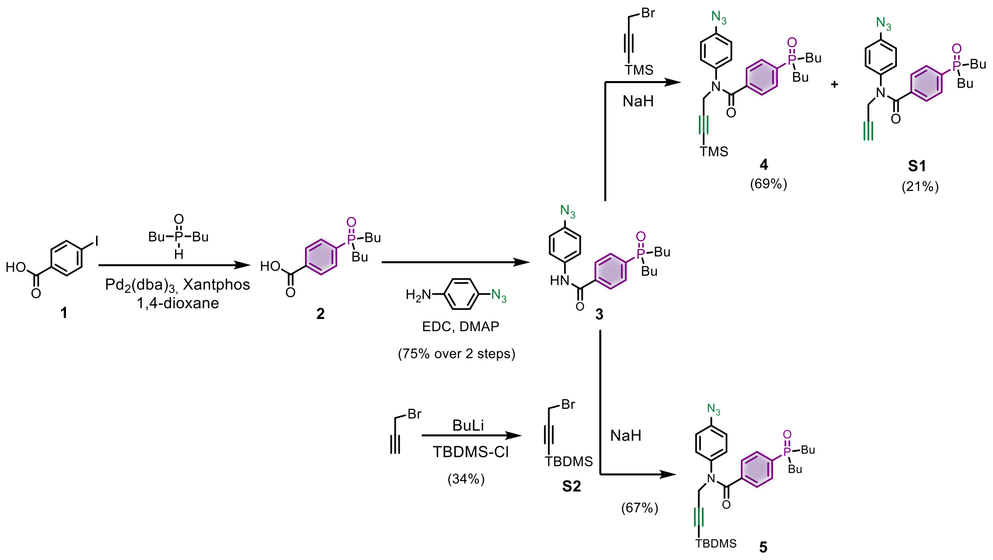

Scheme S1. Synthesis of phosphine oxide 1-mers **4** and **5**.

Synthesis of compound 3.

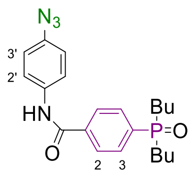

A neat mixture of di-*n*-butylphosphine oxide (0.118 g, 0.73 mmol) and *p*-iodobenzoic acid (0.150 g, 0.61 mmol) was prepared in a two-neck flask. The flask was then evacuated and refilled with nitrogen three times. Under an atmosphere of nitrogen, 1,4-dioxane (degassed by three freeze-pump-thaw cycles and stored under nitrogen, 2 mL) was added, and the resulting mixture was stirred at room temperature for 10 min. In a separate flask, a dry mixture of tris(dibenzylideneacetone)dipalladium (0.014 g, 0.03 mmol) and Xantphos (0.017 g, 0.03 mmol) was dissolved in 1,4-dioxane (1 mL). Triethylamine (0.100 mL, 0.73 mmol) was added and the resulting mixture was stirred overnight at 50 °C. Then, the reaction was diluted with CH_2Cl_2 and transferred to a separation funnel. The organic layer was washed with 1M NaOH soln (3x). The aqueous layer was washed with CH_2Cl_2 (2×), acidified with 1M HCl soln. and then extracted with EtOAc (3x). The organic layers were combined, washed with brine and dried over $MgSO_4$. The crude carboxylic acid derivative **2**, *p*-azidoaniline (0.082 g, 0.61 mmol), EDC (0.174 g, 0.91 mmol) and DMAP (0.074 g, 0.12 mmol) were dissolved in dry CH_2Cl_2 (10 ml) and the reaction was left stirring under N_2 atmosphere at room temperature for 1h. The crude was diluted with EtOAc (50 mL) and washed with 5% aq. soln. HCl (2x), 2N NaOH (2x), H_2O (2x) and brine. The organic phase was dried with $MgSO_4$ and concentrated *in vacuo*. The obtained residue was purified by flash chromatography (from 0% to 5% of MeOH in CH_2Cl_2) to afford compound **3** (0.180 g, 75%) as a brown amorphous solid.

^{1}H NMR (400 MHz, CDCl$_3$): δ_H = 9.99 and 9.95 (s, 1H, NH, rotamers), 8.01 (d, 2H, *J* = 9.0 Hz, 2-H), 7.89 (d, 2H, *J* = 8.0 Hz, 2'-H), 7.60 (t, 2H, *J* = 9.0 Hz, 3-H, rotamers), 7.03 (d, 2H, *J* = 9.0 Hz, 3'-H), 1.94 (m, 2H, 1''-H, Bu), 1.82 (m, 2H, 1''-H, Bu), 1.49 (m, 2H, 2''-H, Bu), 1.32 (m, 6H, 2''-H and 3''-H, Bu), 0.82 (t, 6H, *J* = 7.0 Hz, 4''-H, Bu).

^{13}C NMR (100.6 MHz, CDCl$_3$): δ_C = 165.9 (CO), 138.8 (d, *J* = 3.0 Hz, 1-C), 136.1 (1'-C), 135.8 (4'-C), 135.5 (d, *J* = 90.0 Hz, 4-C), 130.8 (d, *J* = 9.0 Hz, 3-C), 128.0 (d, *J* = 11.0 Hz, 2-C), 122.0 (2'-C), 119.5 (3'-C), 29.5 (d, *J* = 68.5 Hz, 1''-C, Bu), 24.1 (d, *J* = 14.5 Hz, 2''-C, Bu), 23.7 (d, *J* = 4.0 Hz, 3''-C, Bu), 13.7 (4''-C, Bu).

^{31}P NMR (161.3 MHz, CDCl$_3$): δ_P = 41.6.

HRMS (ES+): calcd $C_{21}H_{28}N_4O_2P$ 399.1944 $[M+H]^+$, found 399.1935 $[M+H]^+$.

FT-IR (ATR): ν_{max} 2953, 2927, 2867, 2111, 2080, 1673, 1537, 1504, 1295, 1158 and 748 cm^{-1}.

^{1}H-NMR (400 MHz, $CDCl_3$) compound 3

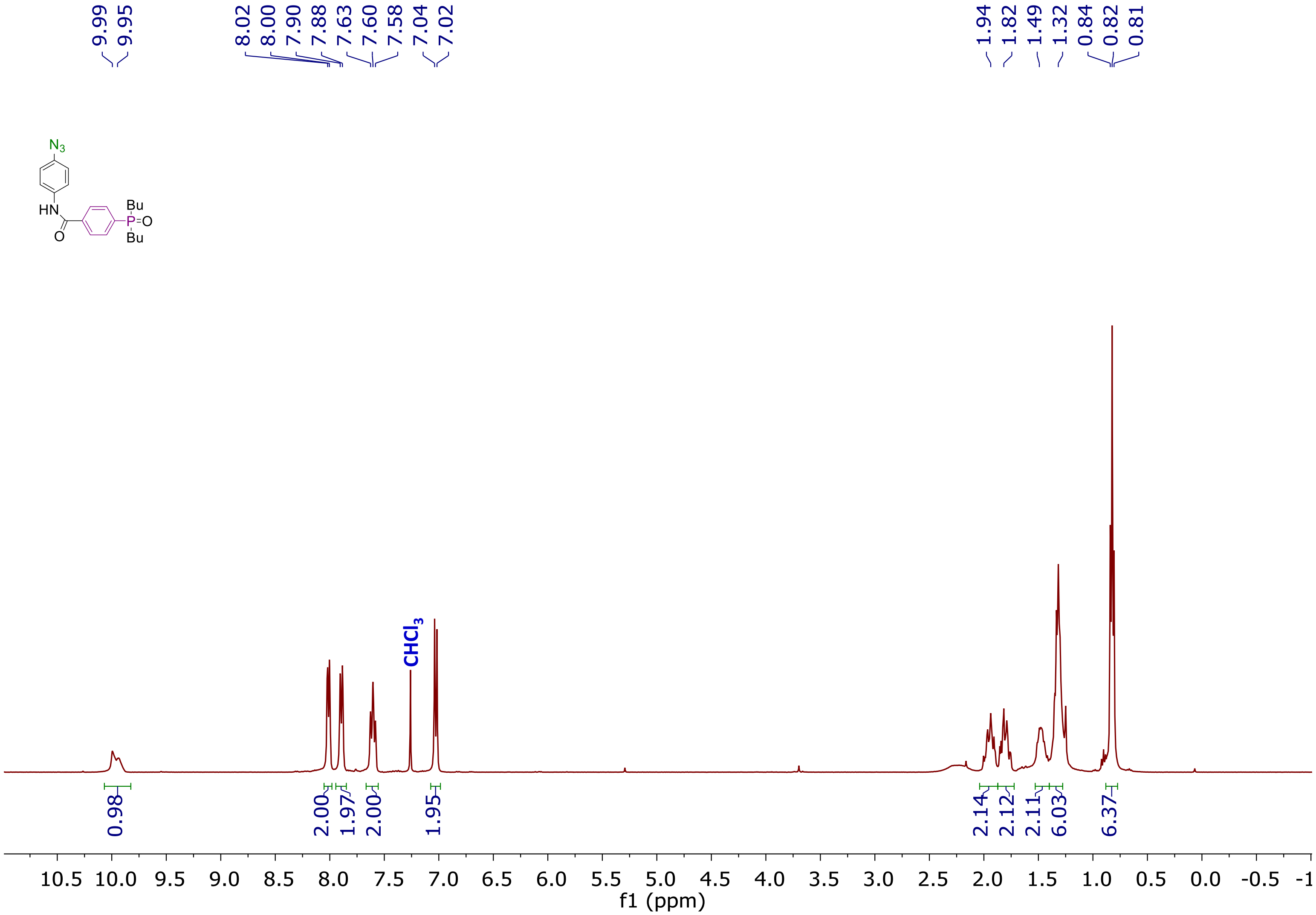

^{13}C-NMR (100.6 MHz, CDCl$_3$) compound 3

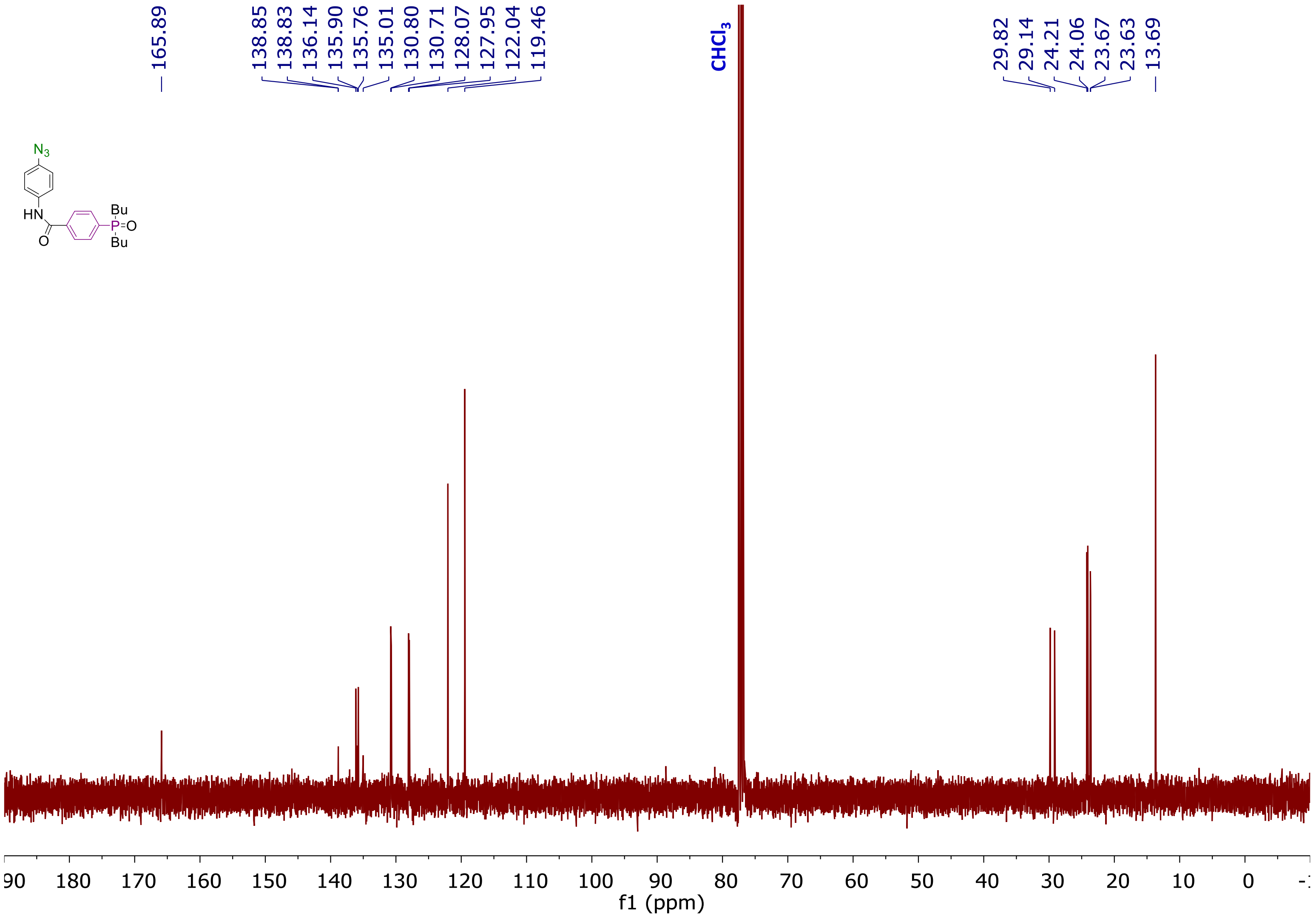

^{31}P NMR (161.3 MHz, $CDCl_3$) compound 3

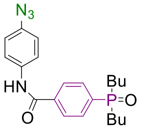

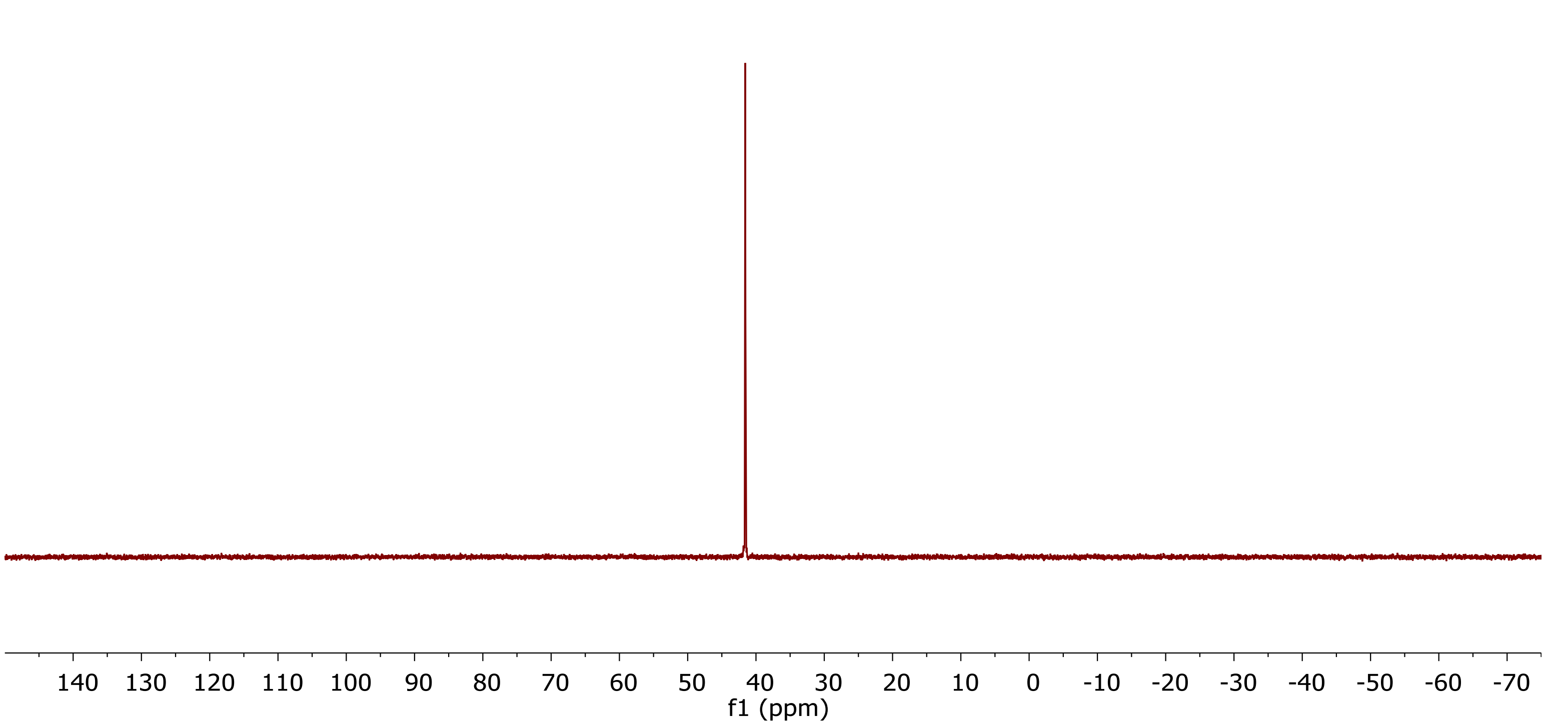

Alkylation of 3 with TMS-protected propargyl bromide

Compound **3** (0.102 g, 0.26 mmol) was dissolved in dry THF (2 mL) and added to a suspension of NaH (60% dispersion in mineral oil, 0.051 g, 1.28 mmol) in THF (8 mL) under inert atmosphere at 0 °C. The reaction was allowed to reach room temperature for 15 min. 3-Bromo-1-(trimethylsilyl)-1-propyne (0.20 mL, 1.28 mmol) was added dropwise and the reaction was vigorously stirred overnight. Satd. aq. NH_4Cl was carefully added at 0 °C and the reaction was extracted with EtOAc (3x). The combined organic phase was washed with brine (1x), dried with anhydrous $MgSO_4$, filtered, and the solvents evaporated. The obtained residue was purified by flash chromatography (from 0% to 3% of MeOH in CH_2Cl_2) to afford compound **4** (0.089 g, 69%) as a pale yellow amorphous solid, and deprotected 1-mer **S1** (0.023 g, 21%) as a pale yellow amorphous solid.

Characterization of compound 4

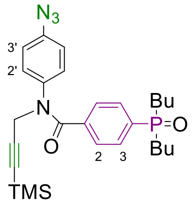

^{1}H NMR (400 MHz, $CDCl_3$): δ_H = 7.53 (m, 2H, 3-H, rotamers), 7.42 (d, 2H, *J* = 6.5 Hz, 2-H), 7.11 (d, 2H, *J* = 8.5 Hz, 2'-H), 6.88 (d, 2H, *J* = 8.5 Hz, 3'-H), 4.68 (s, 2H, N-CH_2), 1.91 (m, 2H, 1''-H, Bu), 1.78 (m, 2H, 1''-H, Bu), 1.52 (m, 2H, 2''-H, Bu), 1.32 (m, 6H, 2''-H and 3''-H, Bu), 0.82 (t, 6H, *J* = 7.0 Hz, 4''-H, Bu), 0.13 (s, 9H, TMS).

^{13}C NMR (100.6 MHz, $CDCl_3$): δ_C = 169.3 (CO), 139.6 (4'-C), 138.8 (1'-C), 138.4 (d, *J* = 3.0 Hz, 1-C), 134.7 (d, *J* = 90.0 Hz, 4-C), 130.2 (d, *J* = 9.0 Hz, 3-C), 129.7 (2'-C), 128.7 (d, *J* = 12.5 Hz, 2-C), 119.7 (3'-C), 100.3 (C-TMS), 90.4 (C, alkyne), 29.6 (d, *J* = 68.5 Hz, 1''-C, Bu), 24.2 (d, *J* = 14.5 Hz, 2''-C, Bu), 23.6 (d, *J* = 4.0 Hz, 3''-C, Bu), 13.7 (4''-C, Bu), -0.1 (TMS).

^{31}P NMR (161.3 MHz, $CDCl_3$): δ_P = 40.4.

HRMS (ES+): calcd $C_{27}H_{38}N_4O_2PSi$ 509.2496 $[M+H]^+$, found 509.2493 $[M+H]^+$.

FT-IR (ATR): ν_{max} 2959, 2925, 2870, 2856, 2125, 2094, 1656, 1506, 1295 and 844 cm^{-1}.

^{1}H-NMR (400 MHz, $CDCl_3$) compound 4

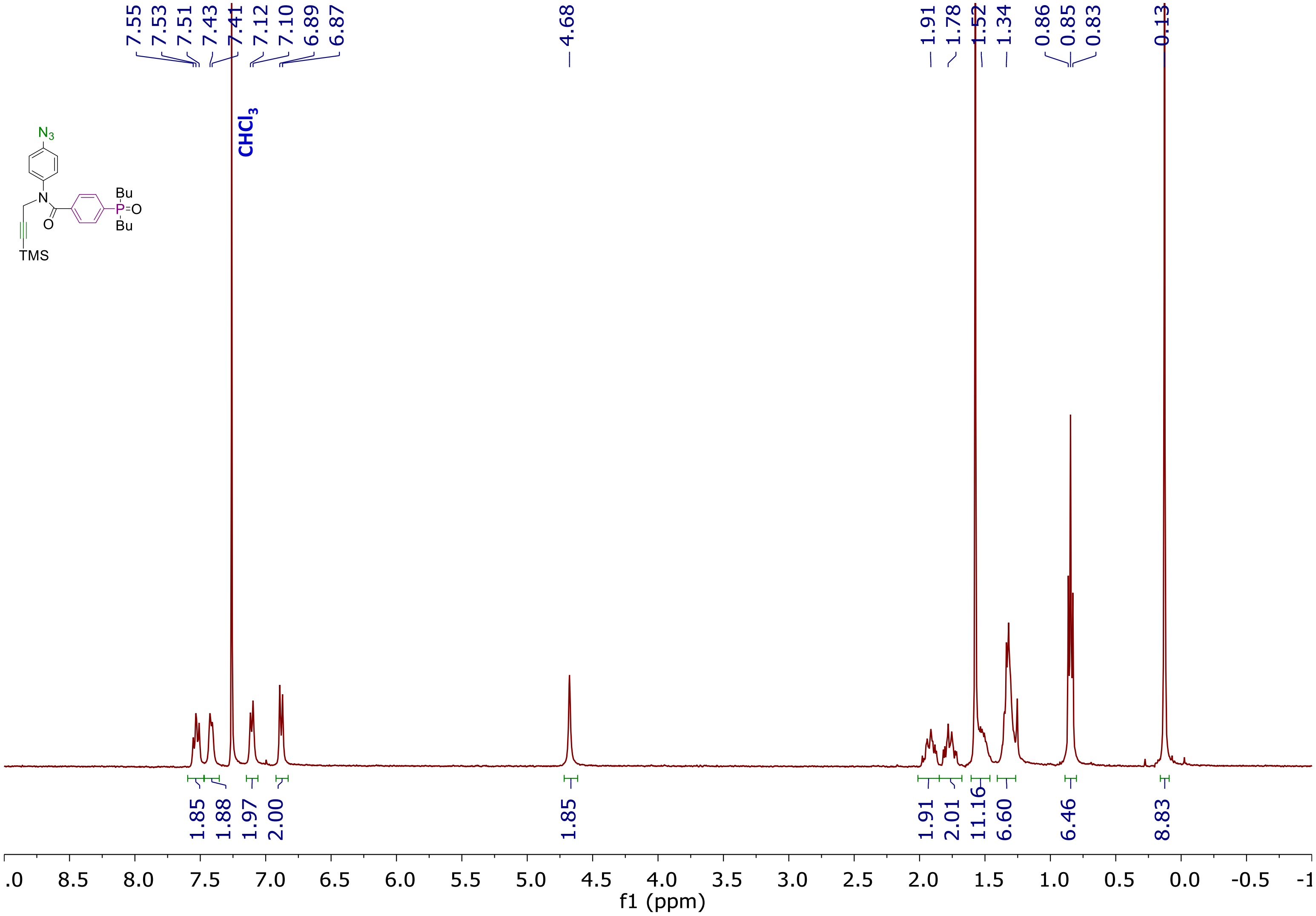

^{13}C-NMR (100.6 MHz, $CDCl_3$) compound 4

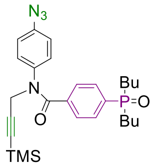

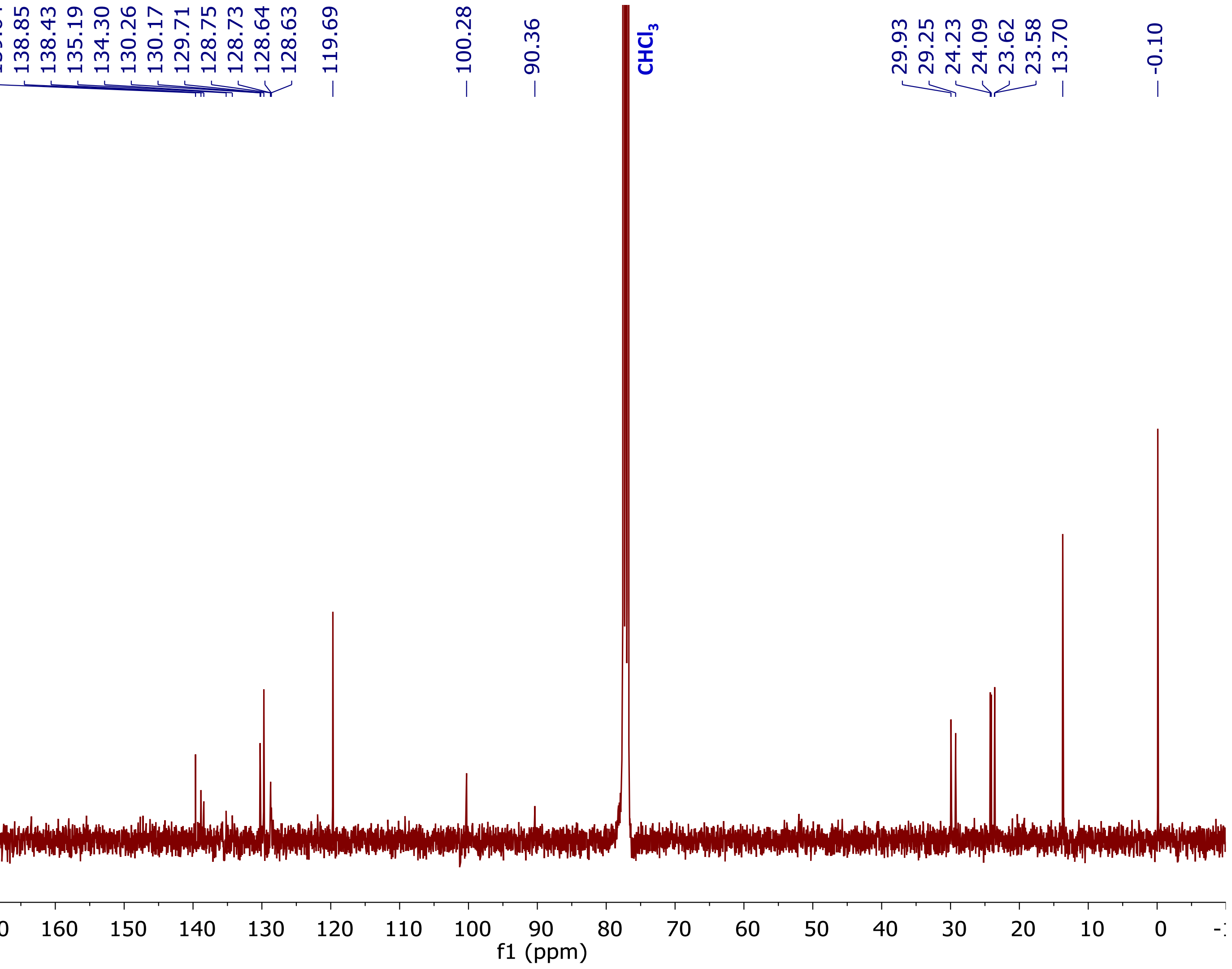

^{31}P NMR (161.3 MHz, $CDCl_3$) compound 4

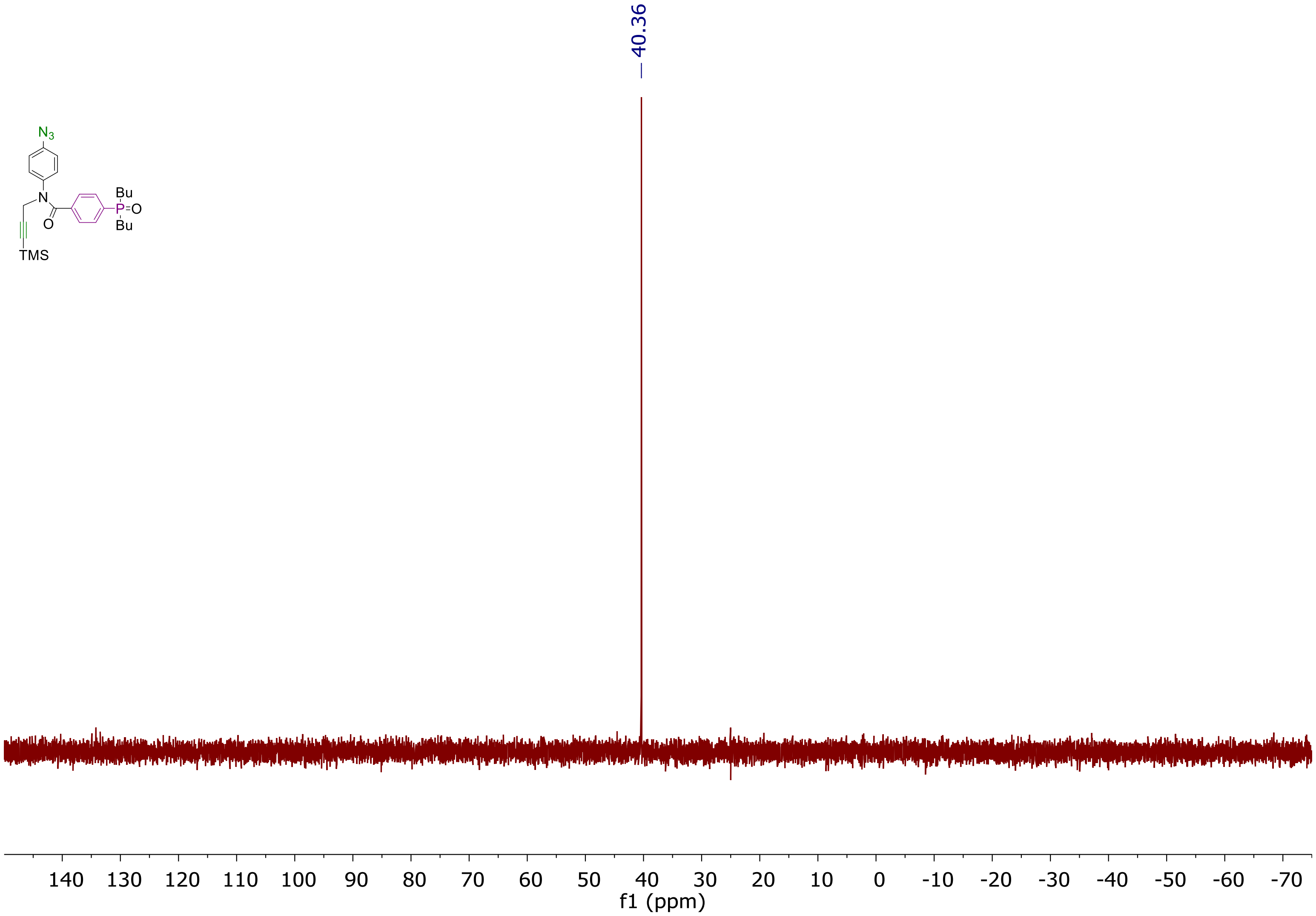

Characterization of compound S1.

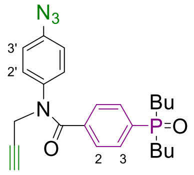

^{1}H NMR (400 MHz, CDCl$_3$): δ_H = 7.53 (m, 2H, 3-H, rotamers), 7.42 (d, 2H, *J* = 7.0 Hz, 2-H), 7.11 (d, 2H, *J* = 8.5 Hz, 2'-H), 6.89 (d, 2H, *J* = 8.5 Hz, 3'-H), 4.66 (d, 2H, *J* = 2.0 Hz, N-CH$_2$), 2.28 (t, 1H, *J* = 2.0 Hz, CH, alkyne), 1.91 (m, 2H, 1''-H, Bu), 1.77j (m, 2H, 1''-H, Bu), 1.52 (m, 2H, 2''-H, Bu), 1.32 (m, 6H, 2''-H and 3''-H, Bu), 0.84 (t, 6H, *J* = 7.0 Hz, 4''-H, Bu).

^{13}C NMR (125.7 MHz, CDCl$_3$): δ_C = 169.4 (CO), 139.7 (4'-C), 138.9 (1'-C), 138.2 (d, *J* = 3.0 Hz, 1-C), 134.9 (d, *J* = 89.5 Hz, 4-C), 130.2 (d, *J* = 9.0 Hz, 3-C), 129.4 (2'-C), 128.8 (d, *J* = 12.5 Hz, 2-C), 119.6 (3'-C), 78.6 (C. alkyne), 72.9 (CH, alkyne), 39.8 (N-CH$_2$), 29.6 (d, *J* = 68.5 Hz, 1''-C, Bu), 24.2 (d, *J* = 14.5 Hz, 2''-C, Bu), 23.6 (d, *J* = 4.0 Hz, 3''-C, Bu), 13.7 (4''-C, Bu).

^{31}P NMR (161.3 MHz, CDCl$_3$): δ_P = 40.5.

HRMS (ES+): calcd $C_{24}H_{30}N_4O_2P$ 437.2101 $[M+H]^+$, found 437.2116 $[M+H]^+$.

FT-IR (ATR): ν_{max} 2957, 2929, 2871, 2859, 2124, 2094, 1715, 1651, 1506, 1378 and 1295 cm^{-1}.

^{1}H-NMR (400 MHz, $CDCl_3$) compound S1

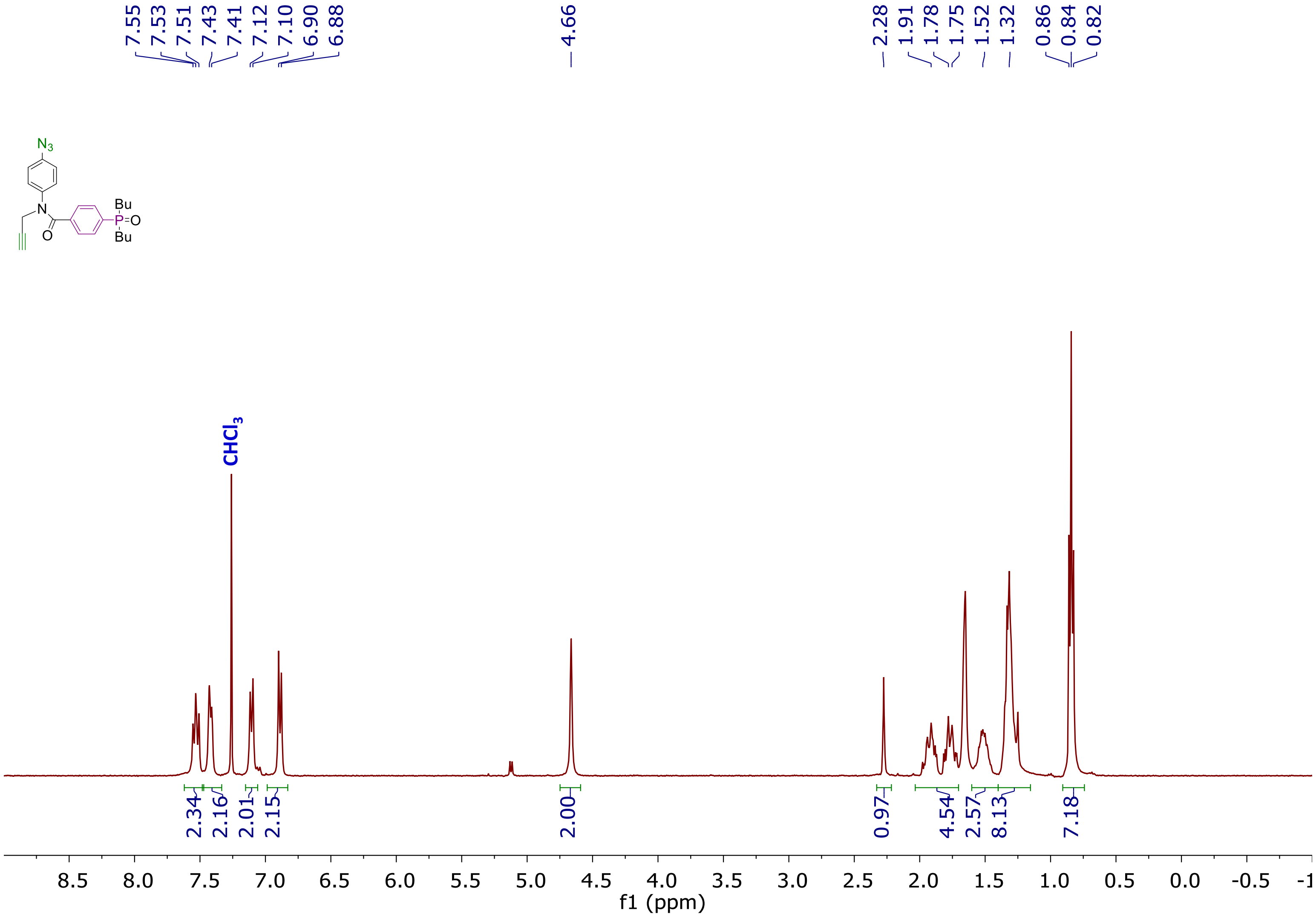

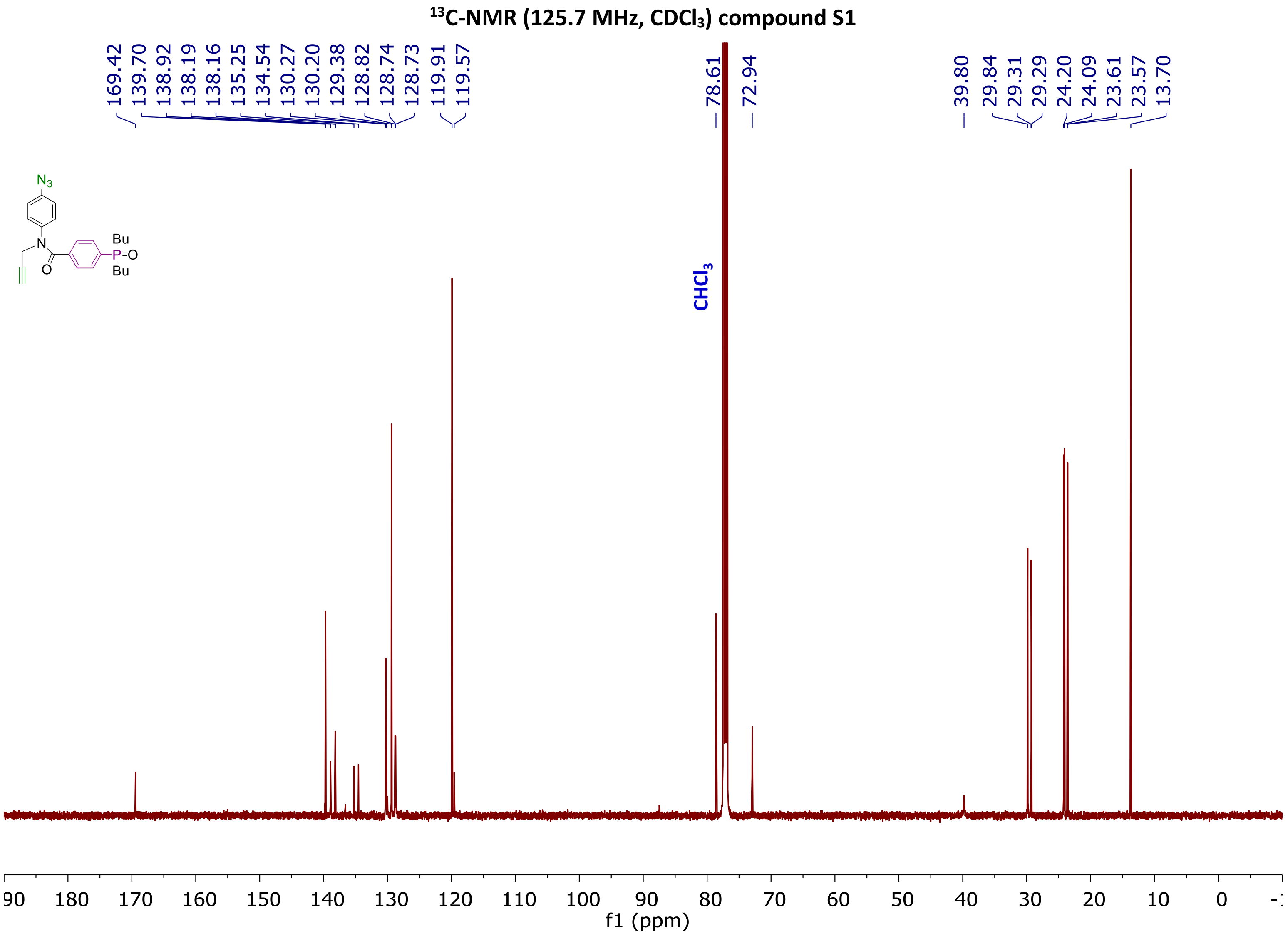

^{13}C-NMR (125.7 MHz, CDCl$_3$) compound S1
169.42
139.70
138.92
138.19
138.16
135.25
134.54
130.27
130.20
129.38
128.82
128.74
128.73
119.91
119.57
78.61
72.94
39.80
29.84
29.31
29.29
24.20
24.09
23.61
23.57
13.70
CHCl$_3$
N$_3$
N
O
Bu
P=O
Bu
f1 (ppm)

^{31}P NMR (161.3 MHz, $CDCl_3$) compound S1

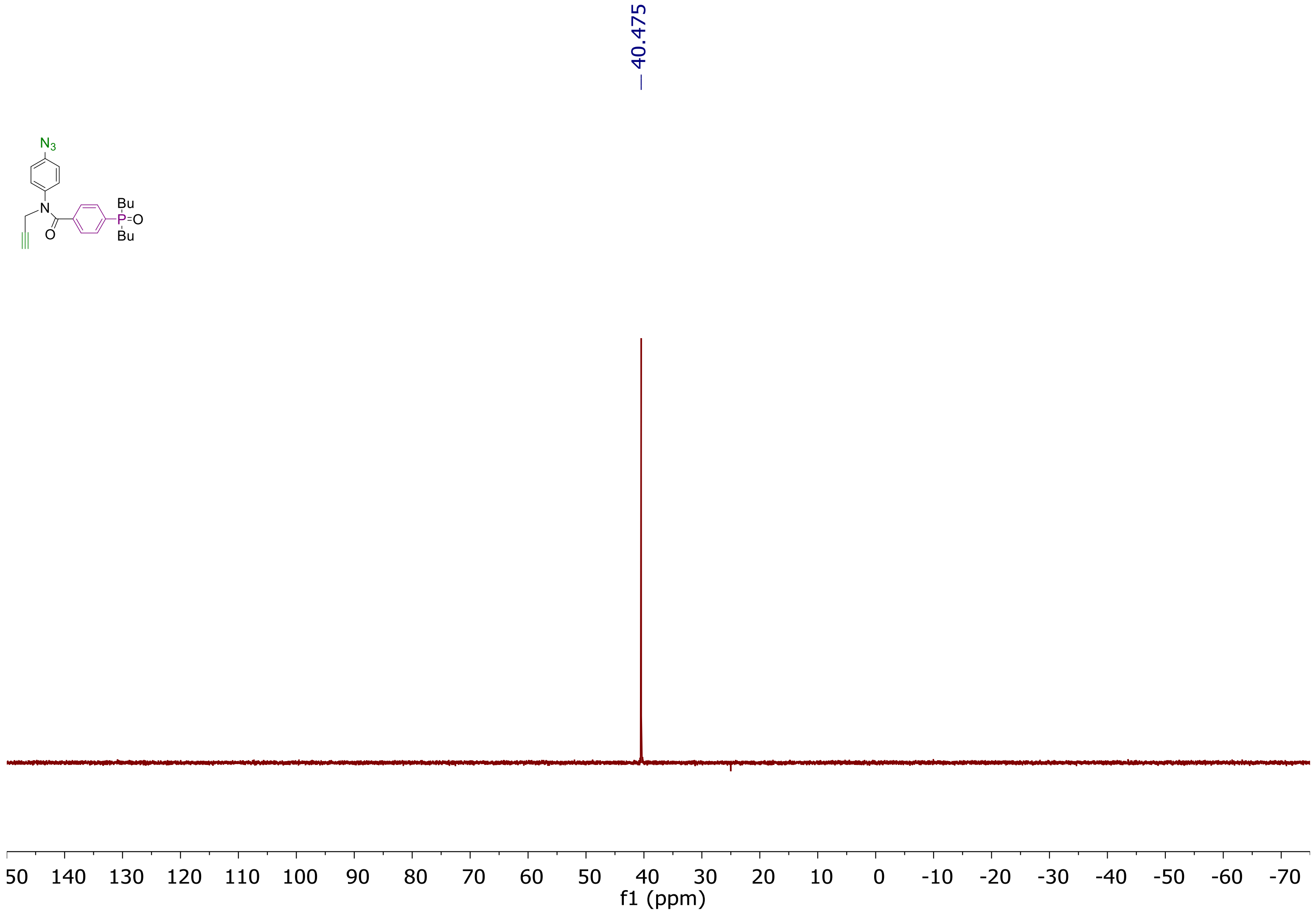

Synthesis of compound S2

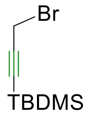

A solution of propargyl bromide (80% in toluene, 2.5 g, 16.8 mmol) in dry THF (45 mL) and under N_2 atmosphere was cooled down to -78 °C. The, BuLi (1.6 M in hexanes, 10.5 mL, 16.8 mmol) was added dropwise and the reaction stirred at -78 oC for 30 min. A solution of TBDMS-Cl (5.1 g, 33.6 mmol) in THF (5 mL) was added to the reaction vessel and the reacti on stirred at -78 oC for 2 h and left to 0 oC over 2h. Satd. aq. NH_4Cl was carefully added at 0 °C and the solution left to reach room temperature before extraction with EtOAc (3x). The combined organic phase was washed with brine (1x), dried with anhydrous $MgSO_4$, filtered, and the solvents evaporated. The obtained residue was purified by flash chromatography using pet. ether as solvent to afford **S2** (1.35 g, 34%) as a pale yellow oil, containing minor impurities of *tert*-butyl(hept-1-yn-1-yl)dimethylsilane.

^{1}H NMR (400 MHz, CDCl$_3$): δ_H = 3.92 (s, 2H, CH_2), 0.94 (s, 9H, ^{t}Bu, TBDMS), 0.11 (s, 6H, CH_3, TBDMS).

^{13}C NMR (100.6 MHz, CDCl$_3$): δ_C = 100.7 (C, alkyne), 91.0 (C, alkyne), 28.5 (CH_2), 26.2 (CH_3, ^{t}Bu, TBDMS), 14.9 (C, ^{t}Bu), -4.7 (CH_3, TBDMS).

FT-IR (ATR): ν_{max} 2954, 2930, 2858, 2176, 1715, 1251, 1205, 1035, 824, 775, 681 and 617 cm^{-1}.

^1H-NMR (400 MHz, $CDCl_3$) compound S2

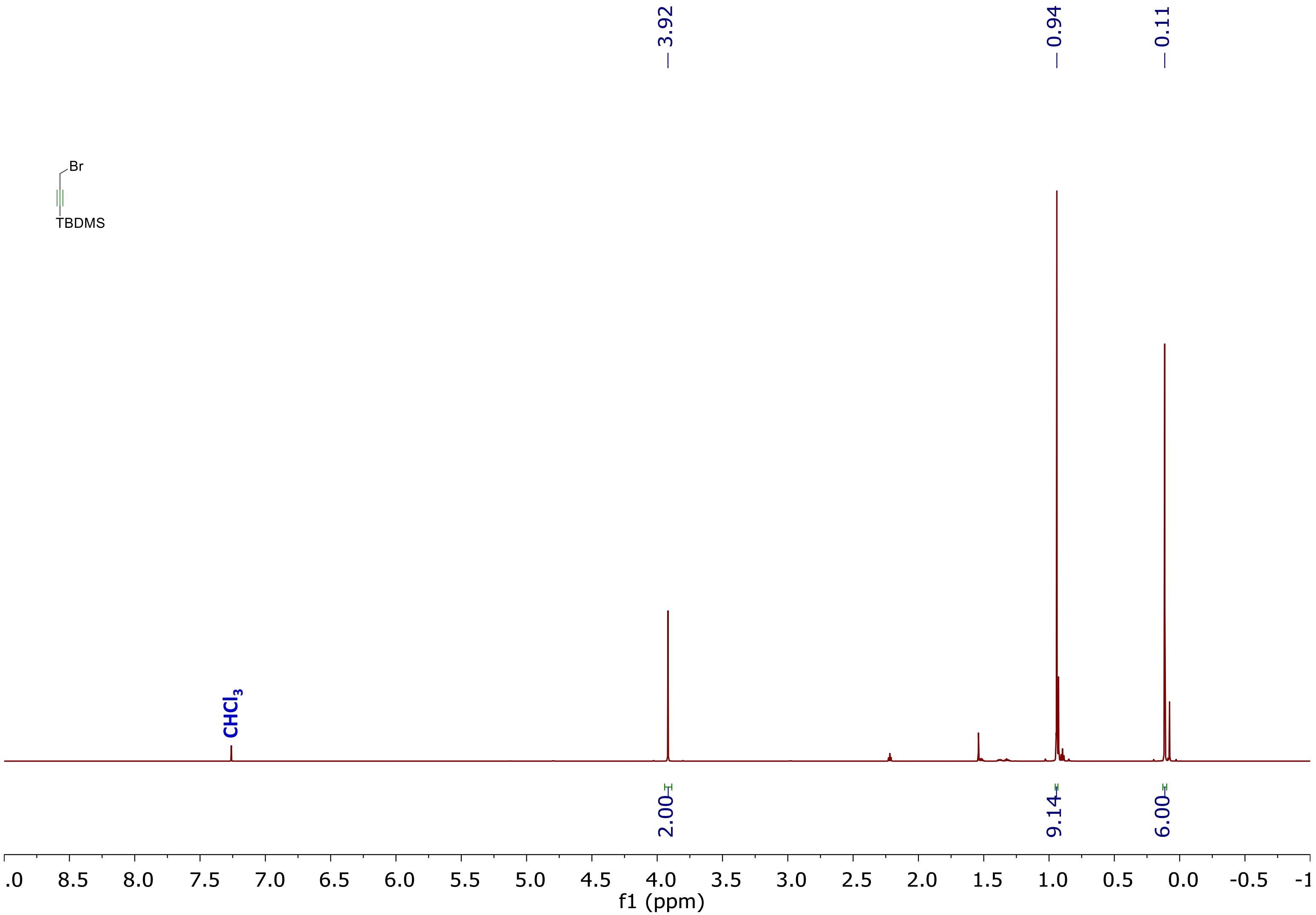

^{13}C-NMR (100.6 MHz, $CDCl_3$) compound S2

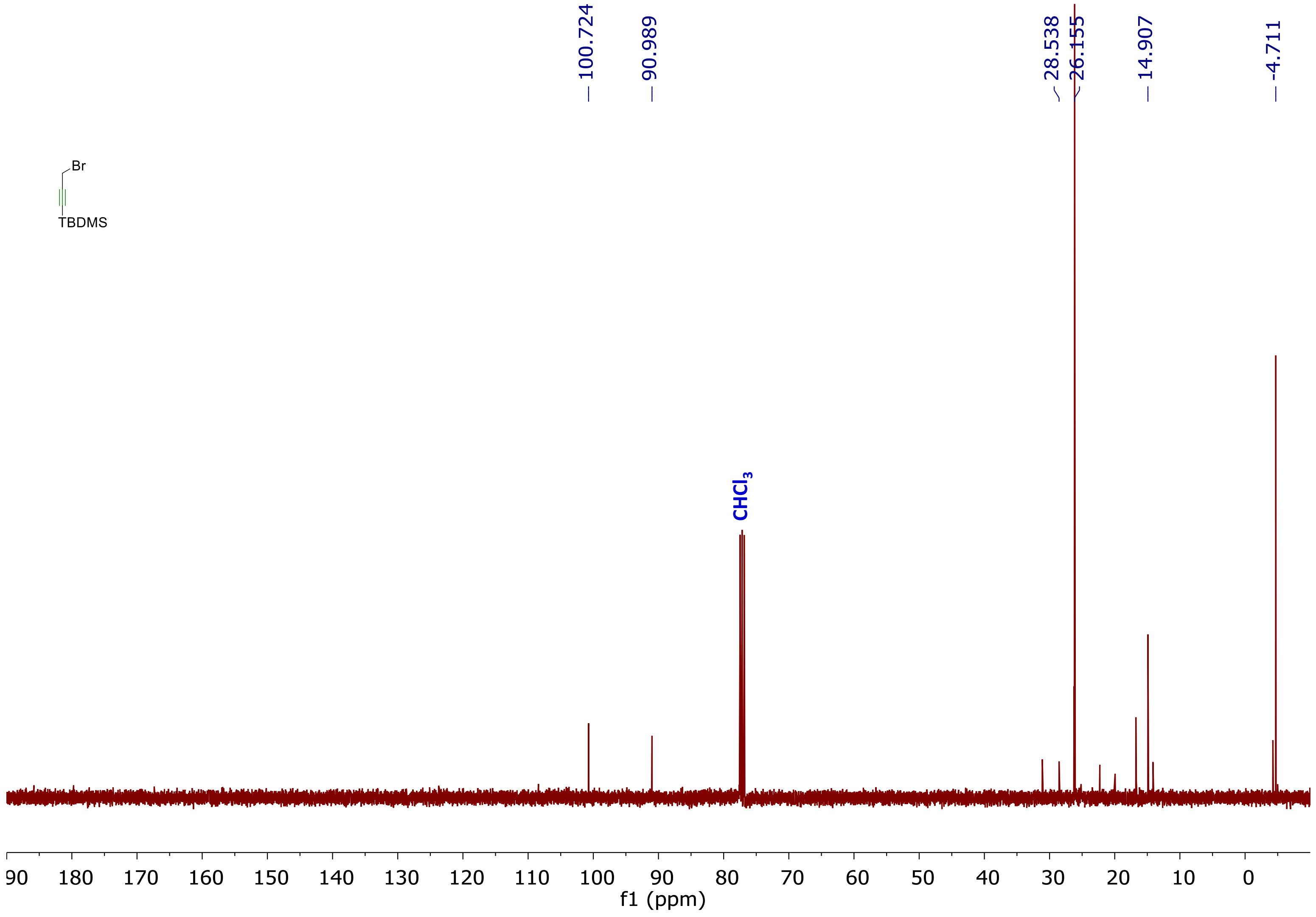

Synthesis of compound 5

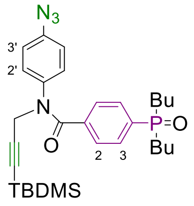

Compound **3** (0.027 g, 0.07 mmol) was dissolved in dry THF (1 mL) and added to a suspension of NaH (60% dispersion in mineral oil, 0.014 g, 0.34 mmol) in THF (4 mL) under inert atmosphere at 0 ^{o}C. The reaction was allowed to reach room temperature for 15 min. A solution of 3-Bromo-1-(*tert*-butyldimethylsilyl)-1-propyne **S2** (0.080 g, 0.34 mmol) in dry THF (0.5 mL) was added dropwise and the reaction was vigorously stirred overnight. Satd. aq. NH_4Cl was carefully added at 0 ^{o}C and the reaction was extracted with EtOAc (3x). The combined organic phase was washed with brine (1x), dried with anhydrous $MgSO_4$, filtered, and the solvents evaporated. The obtained residue was purified by flash chromatography (from 0% to 100% of EtOAc in pet. ether) to afford compound **5** (0.025 g, 67%) as a pale yellow amorphous solid.

^{1}H NMR (400 MHz, $CDCl_3$): δ_H = 7.53 (m, 2H, 3-H, rotamers), 7.40 (d, 2H, *J* = 6.5 Hz, 2-H), 7.10 (d, 2H, *J* = 8.5 Hz, 2'-H), 6.86 (d, 2H, *J* = 8.5 Hz, 3'-H), 4.70 (s, 2H, N-CH_2), 1.91 (m, 2H, 1''-H, Bu), 1.76 (m, 2H, 1''-H, Bu), 1.52 (m, 2H, 2''-H, Bu), 1.31 (m, 6H, 2''-H and 3''-H, Bu), 0.86 (s, 9H, ^{t}Bu, TBDMS), 0.84 (t, 6H, *J* = 7.0 Hz, 4''-H, Bu), -0.05 (s, 6H, CH_3, TBDMS).

^{13}C NMR (100.6 MHz, $CDCl_3$): δ_C = 169.2 (CO), 139.6 (4'-C), 138.8 (1'-C), 138.5 (d, *J* = 3.0 Hz, 1-C), 134.7 (d, *J* = 89.5 Hz, 4-C), 130.2 (d, *J* = 8.5 Hz, 3-C), 129.6 (2'-C), 128.6 (d, *J* = 11.0 Hz, 2-C), 119.7 (3'-C), 100.8 (C-Si), 87.5 (C-alkyne), 29.6 (d, *J* = 68.5 Hz, 1''-C, Bu), 26.1 (CH_3, ^{t}Bu, TBDMS), 24.2 (d, *J* = 14.5 Hz, 2''-C, Bu), 23.6 (d, *J* = 4.0 Hz, 3''-C, Bu), 16.6 (C, ^{t}Bu, TBDMS), 13.7 (4''-H, Bu), -4.6 (CH_3, TBDMS).

^{31}P NMR (161.3 MHz, $CDCl_3$): δ_P = 40.4.

HRMS (ES+): calcd $C_{30}H_{44}N_4O_2PSi$ 551.2966 $[M+H]^+$, found 551.2982 $[M+H]^+$.

FT-IR (ATR): ν_{max} 2957, 2930, 2127, 2095, 1713, 1655, 1507, 1362, 1295, 1221 and 840 cm^{-1}.

^{1}H-NMR (400 MHz, $CDCl_3$) compound 5

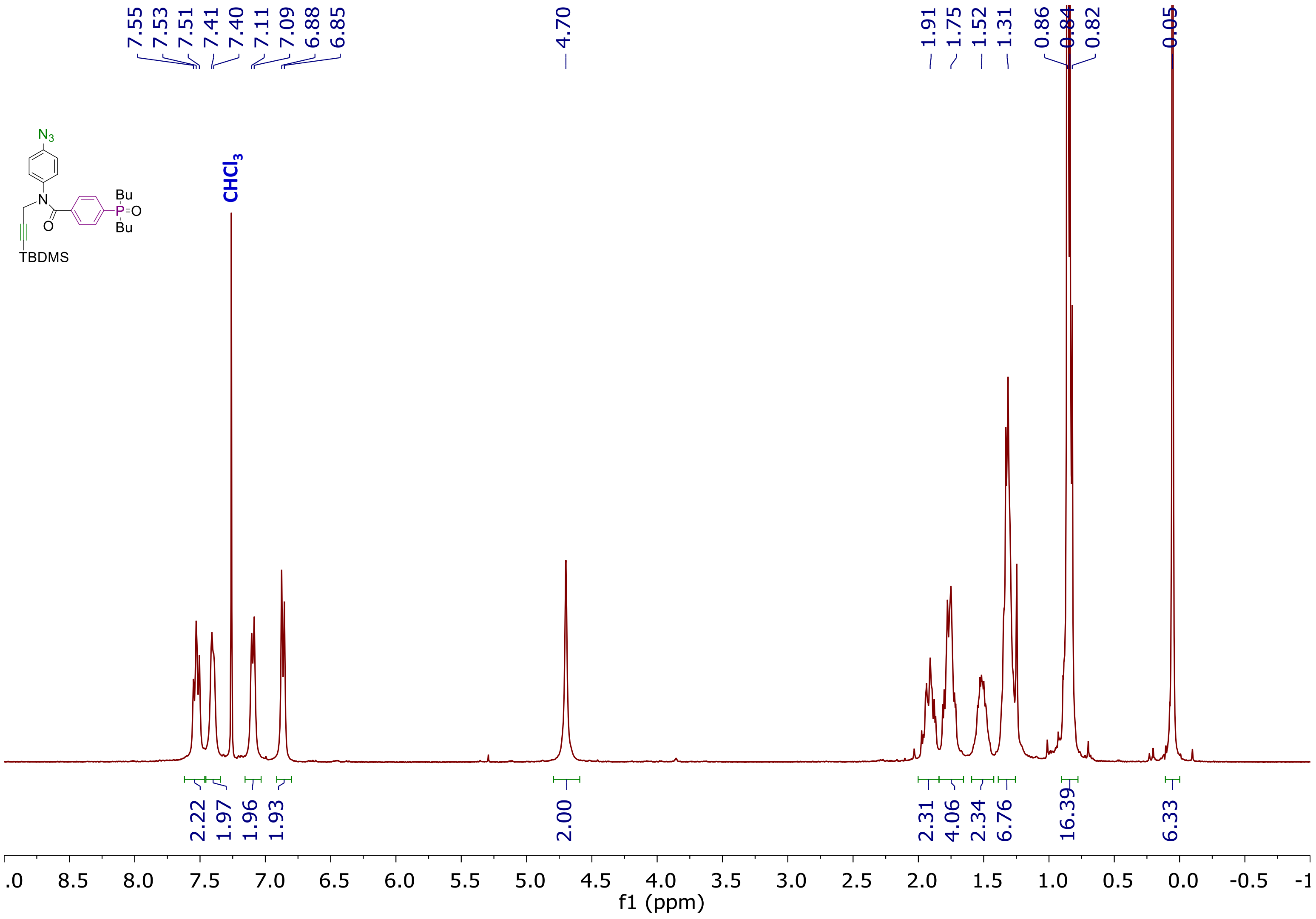

^{13}C-NMR (100.6 MHz, CDCl$_3$) compound 5

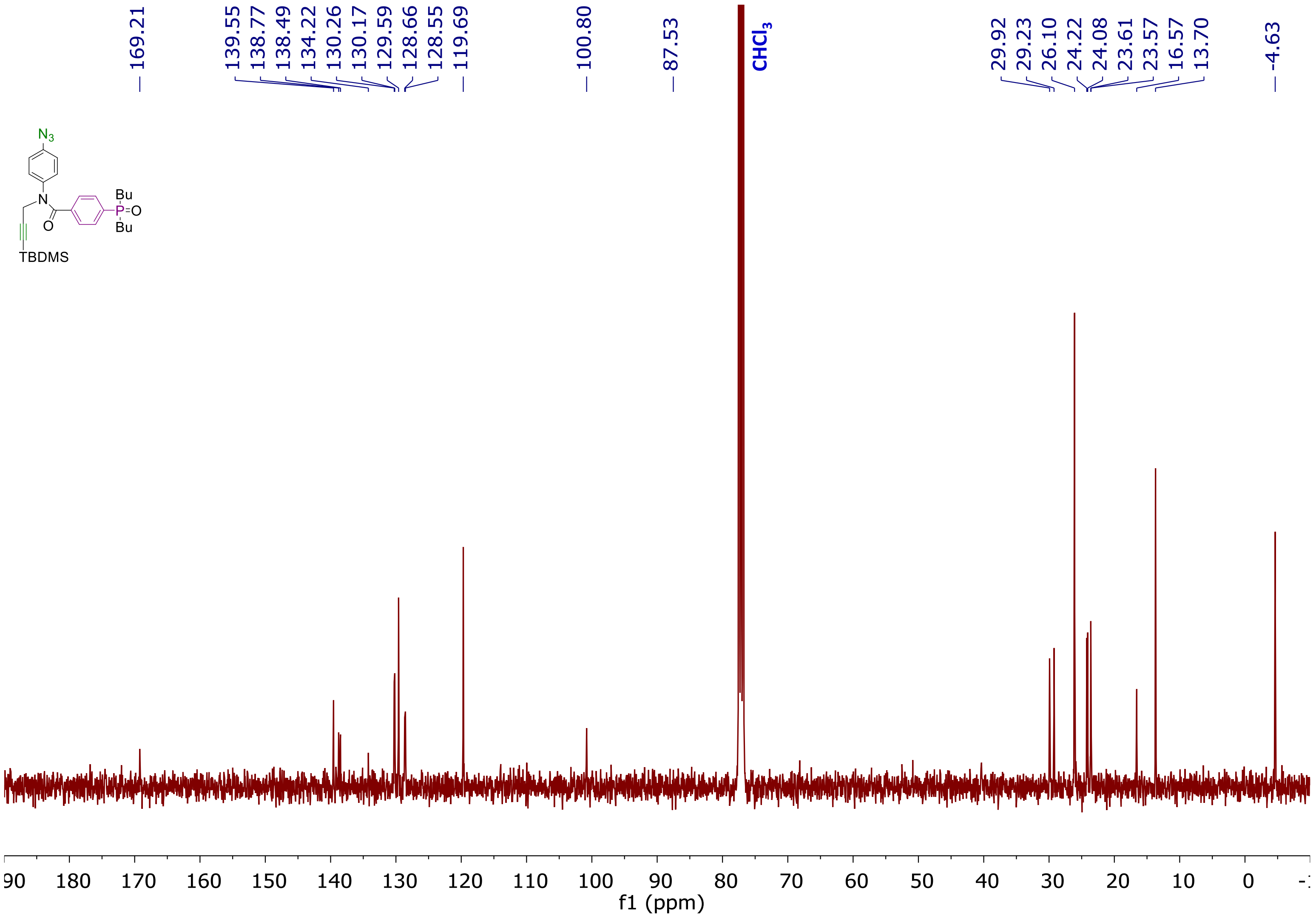

^{31}P NMR (161.3 MHz, $CDCl_3$) compound 5

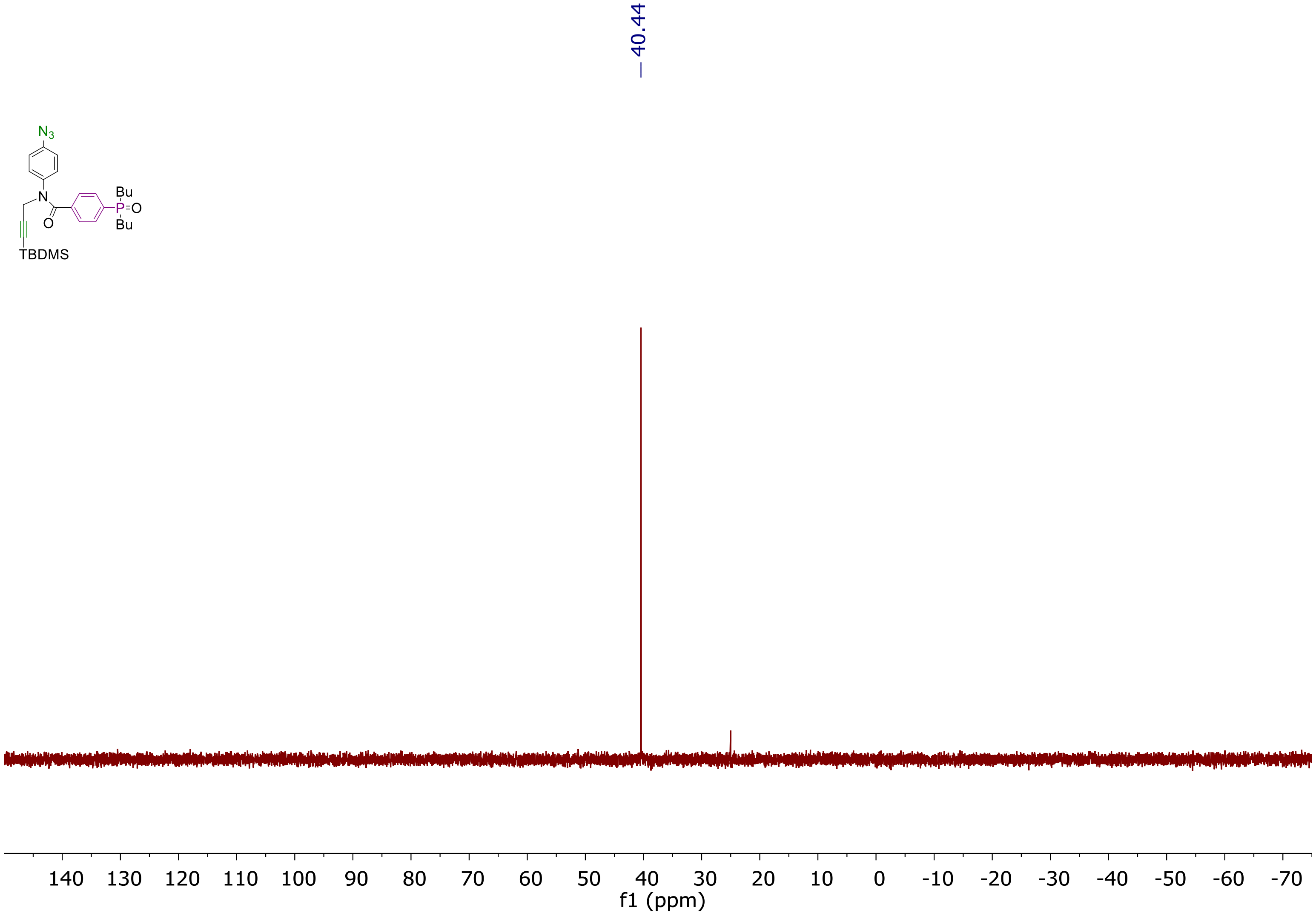

2.2. Synthesis of phenol building blocks

Scheme S2 shows the structure of compounds **14**, **19** and **S3**, which have been previously described.[S1] Compound **S3** is the starting material to obtain phenol 1-mer **25** through benzylation using NaH and subsequent TBAF-mediated phenol deprotection. Following this procedure, **25** was obtained in moderate yield due to deprotection and benzylation of the phenol moiety during the reaction, which afforded doubly benzylated 1-mer **S4** in good yield.

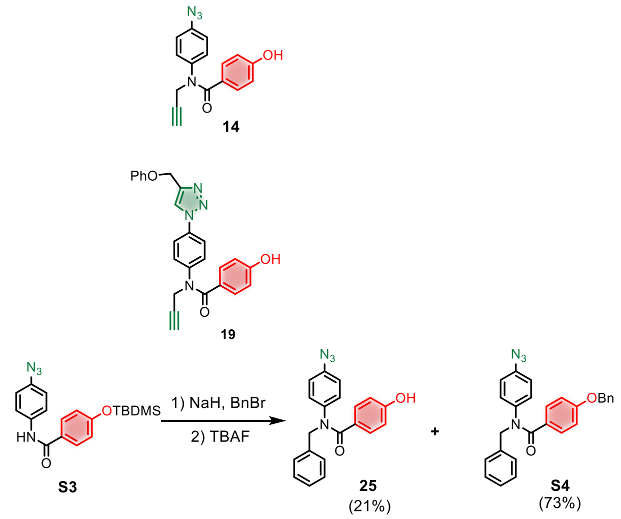

Scheme S2. Structure of phenol 1-mers **14** and **19** and synthesis of phenol 1-mer **25**.

Benzylation of S3 followed by phenol deprotection

Compound **S3**[S1](0.120 g, 0.33 mmol) was dissolved in dry THF (8 mL) and added to a suspension of NaH (60% dispersion in mineral oil, 0.065 g, 1.63 mmol) in THF (2 mL) under inert atmosphere at 0 °C. The reaction was allowed to reach room temperature for 15 min. Benzyl bromide (0.19 mL, 1.63 mmol) was added dropwise and the reaction was vigorously stirred overnight. Satd. aq. NH_4Cl was carefully added at 0 °C and the reaction was extracted with EtOAc (3x). The combined organic phase was evaporated to dryness, dissolved in dry THF (5 mL) and then treated with TBAF solution (1M in THF, 0.33 mL, 0.33 mmol). The reaction was stirred for 10 minutes at room temperature and quenched with 5% soln. HCl and extracted with EtOAc (3x) followed by washing with H_2O and brine. The organic layer was dried over anhydrous $MgSO_4$ and concentrate under vacuum. The obtained residue was purified by flash chromatography (from 0% to 50% of EtOAc in Pet. Ether) to afford monobenzylated 1-mer **25** (0.024 g, 21%) as a yellow amorphous solid along with dibenzylated 1-mer **S4** (0.104 g, 73%) as a yellow oil.

Characterization of compound 25

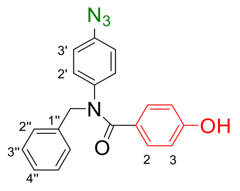

^{1}H NMR (400 MHz, $CDCl_3$): δ_H = 7.51 (s, 1H, OH), 7.26 (bs, 5H, Ph), 7.15 (d, 2H, *J* = 8.5 Hz, 2-H), 6.87 (d, 2H, *J* = 8.5 Hz, 2'H), 6.80 (d, 2H, *J* = 8.5 Hz, 3'-H), 6.54 (d, 2H, *J* = 8.5 Hz, 3-H), 5.08 (s, 2H, N-CH_2).

^{13}C NMR (100.6 MHz, $CDCl_3$): δ_C = 171.2 (CO), 158.3 (4-C), 140.5 (1'-C), 138.5 (4'-C), 137.2 (1''-C, phenyl), 131.0 (2-C), 129.1 (2'-C), 128.7, 128.5 and 127.7 (2''-C, 3''-C and 4''-C, Ph), 126.7 (1-C), 119.7 (3'-C), 115.1 (3-C), 54.3 (N-CH_2).

HRMS (ES+): calcd for $C_{20}H_{17}N_4O_2$ 345.1346 $[M+H]^+$, found 345.1350 $[M+H]^+$.

FT-IR (ATR): ν_{max} 3232, 2918, 2827, 2123, 2093, 1608, 1505, 1228 and 1220 cm^{-1}.

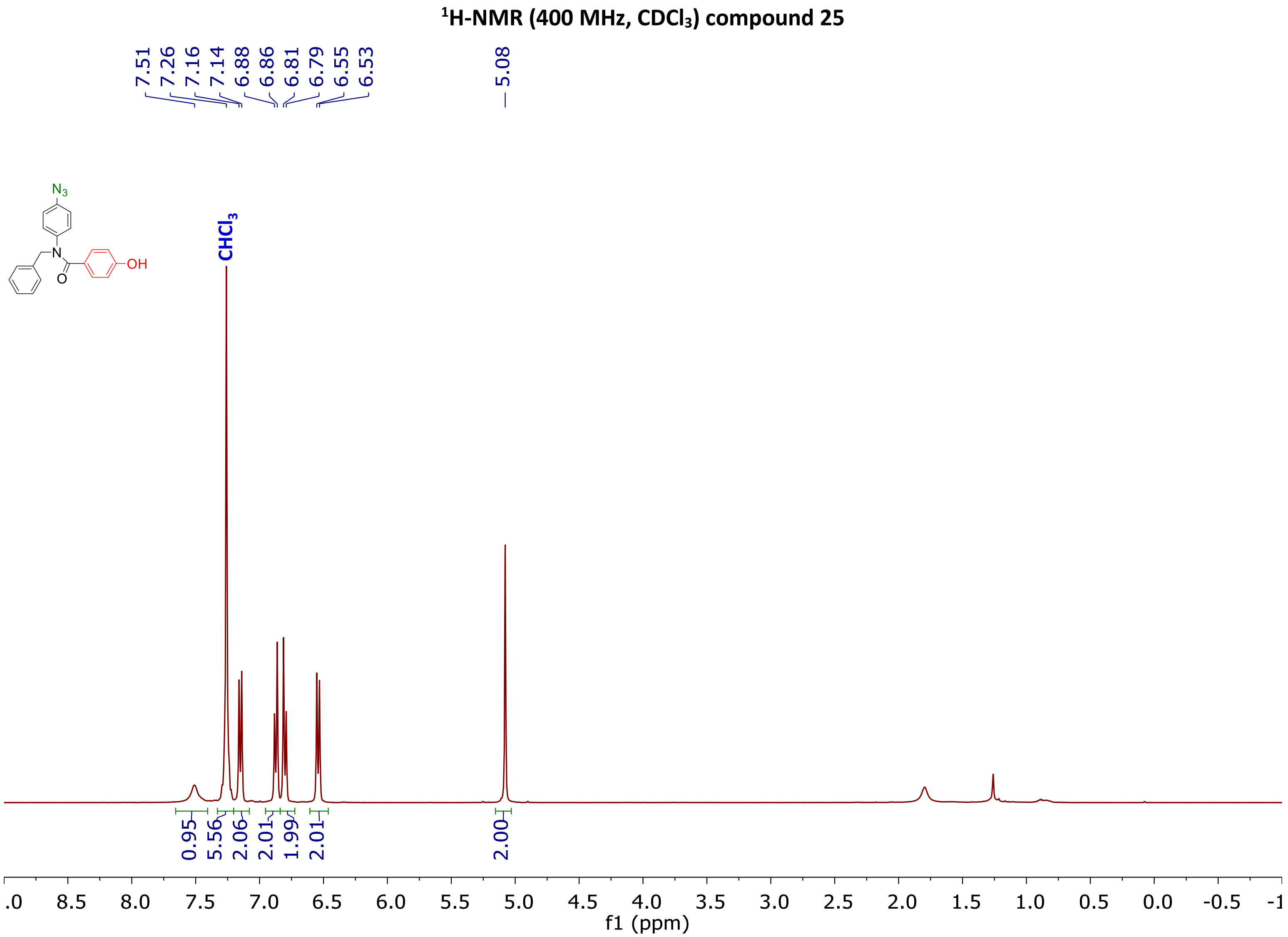

1H-NMR (400 MHz, CDCl3) compound 25
7.51
7.26
7.16
7.14
6.88
6.86
6.81
6.79
6.55
6.53
5.08
N3
N
O
OH
CHCl3
0.95
5.56
2.06
2.01
1.99
2.01
2.00
f1 (ppm)

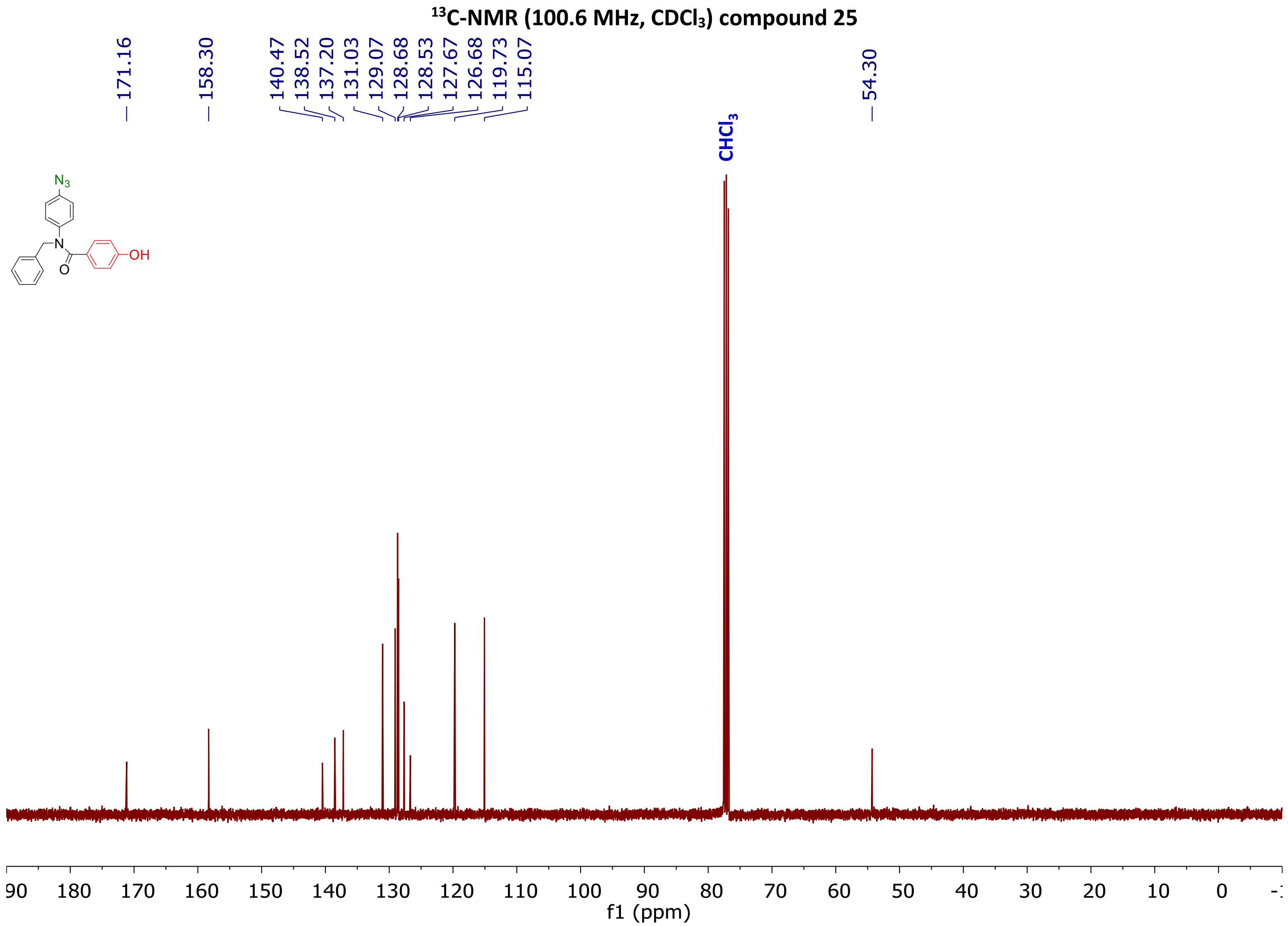

^{13}C-NMR (100.6 MHz, CDCl$_3$) compound 25
171.16
158.30
140.47
138.52
137.20
131.03
129.07
128.68
128.53
127.67
126.68
119.73
115.07
54.30
CHCl$_3$
N$_3$
N
O
OH
90
180
170
160
150
140
130
120
110
100
90
80
70
60
50
40
30
20
10
0
f1 (ppm)

Characterization of compound S4

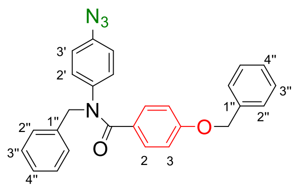

^{1}H NMR (400 MHz, CDCl$_3$): δ_H = 7.38-7.25 (m, 12H, 2x Ph, 2-H), 6.88 (d, 2H, J = 8.5 Hz, 2'H), 6.81 (d, 2H, J = 8.5 Hz, 3'-H), 6.76 (d, 2H, J = 8.5 Hz, 3-H), 5.09 (s, 2H, N-CH$_2$), 5.00 (s, 2H, O-CH$_2$).

^{13}C NMR (100.6 MHz, CDCl$_3$): δ_C = 170.1 (CO), 160.0 (4-C), 140.9 (1'-C), 138.3 (4'-C), 137.6 (1''-C, N-Bn), 136.5 (1''-C, O-Bn), 131.1 (2-C), 129.1 (2'-C), 128.7, 128.7, 128.6, 128.2, 128.1, 127.6 and 127.6 (2''-C, 3''-C and 4''-C, Ph; 1-C), 119.7 (3'-C), 114.2 (3-C), 70.1 (O-CH$_2$), 54.1 (N-CH$_2$).

HRMS (ES+): calcd for $C_{27}H_{23}N_4O_2$ Exact Mass: 435.1816 [M+H]$^+$, found 435.1824 [M+H]$^+$.

FT-IR (ATR): ν_{max} 2921, 2124, 2093, 1641, 1605, 1505, 1296, 1248, 1174 and 838 cm^{-1}.

^{1}H-NMR (400 MHz, $CDCl_3$) compound S4

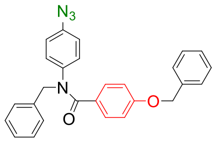

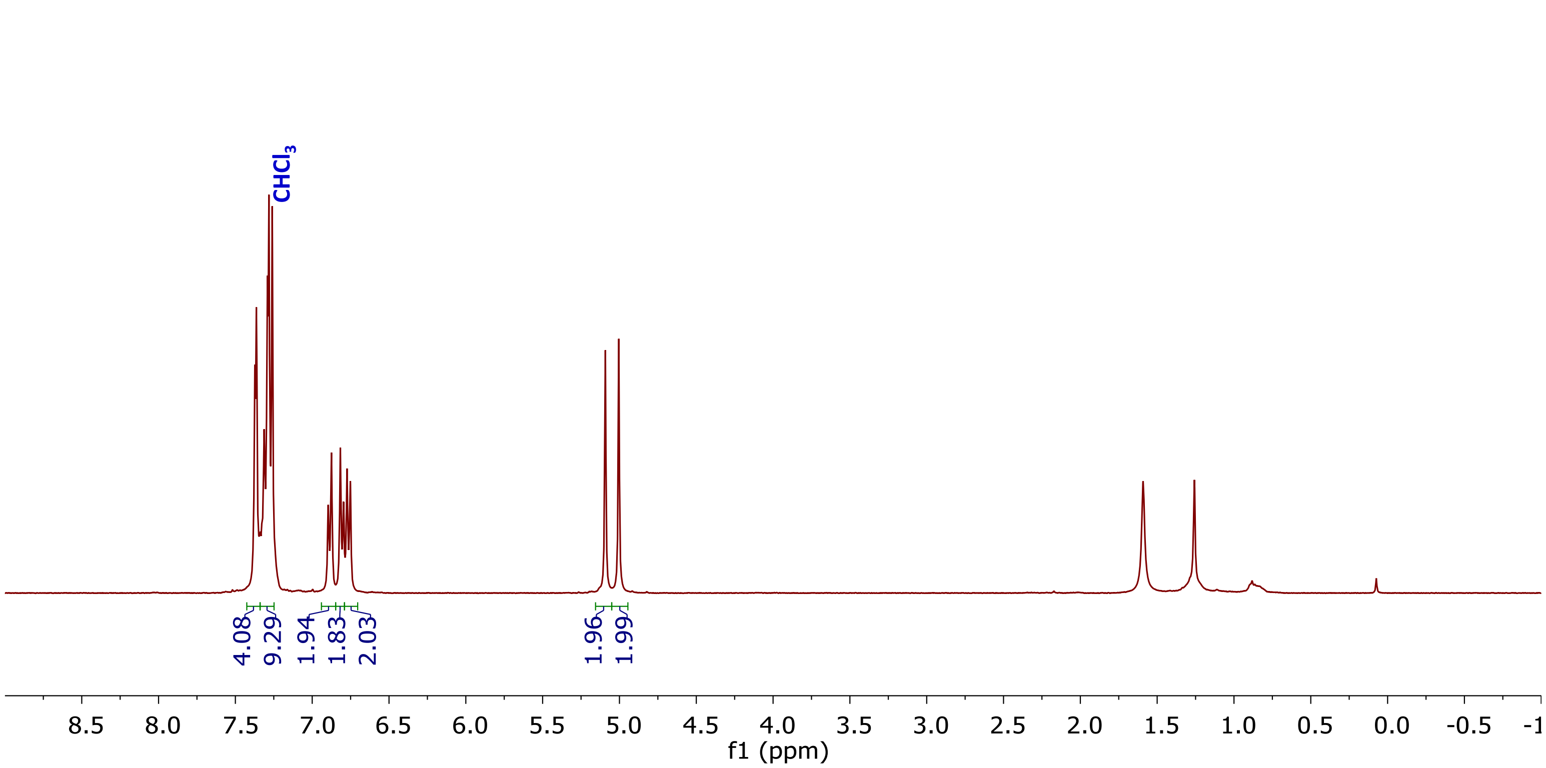

^{13}C-NMR (100.6 MHz, $CDCl_3$) compound S4

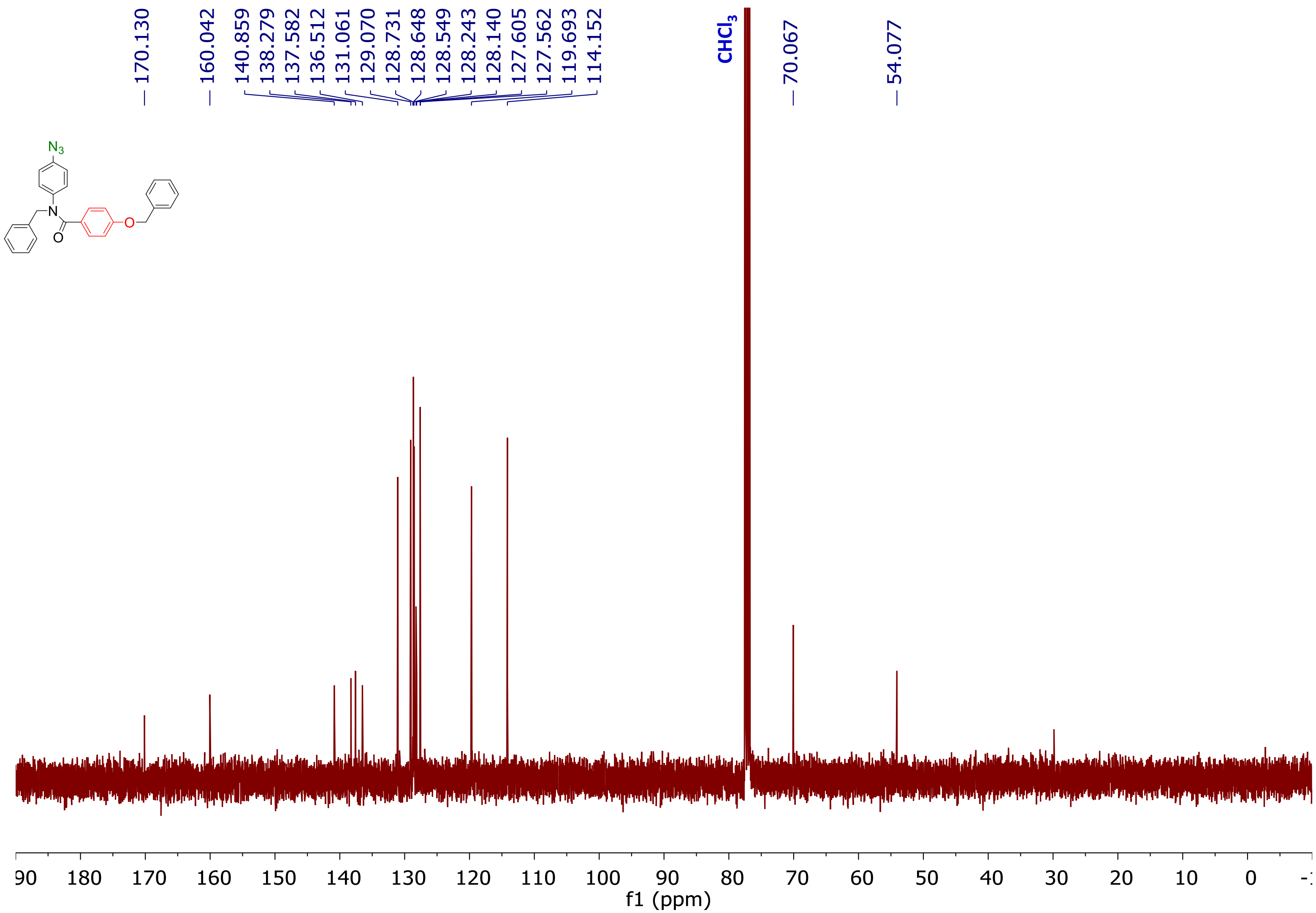

3. Synthesis and characterization of oligomers

3.1. Phosphine oxide homo-oligomers (A_N)

As shown in Scheme S3, phosphine oxide homo-oligomers were prepared through non-templated CuAAC oligomerization of phosphine oxide 1-mer **4**, after deprotection of the TMS group using TBAF (compound **S1**). 1-(Azidomethyl)-4-*tert*-butylbenzene[S1] was used as end-capping group to provide a distribution of oligomers from 1-mer **6** to 4-mer **9**. These oligomers isolated and then capped individually using 2-butyloctyl propargyl ether (compound **S5**) to provide fully capped oligomers 10-13. Figure S1 shows the UPLC traces of the starting 1-mer **S1**, the oligomerization reaction crude mixture and the isolated oligomers **6**-**9** prior to the subsequent capping using **S5**. Figure S2 shows the methylene region of the ^{1}H NMR spectra and the ^{31}P NMR spectra for the final phosphine oxide oligomers **10**-**13**. In the spectra, the extra methylene and phosphine oxide groups corresponding to the increasing number of repeating units can be easily visualized.

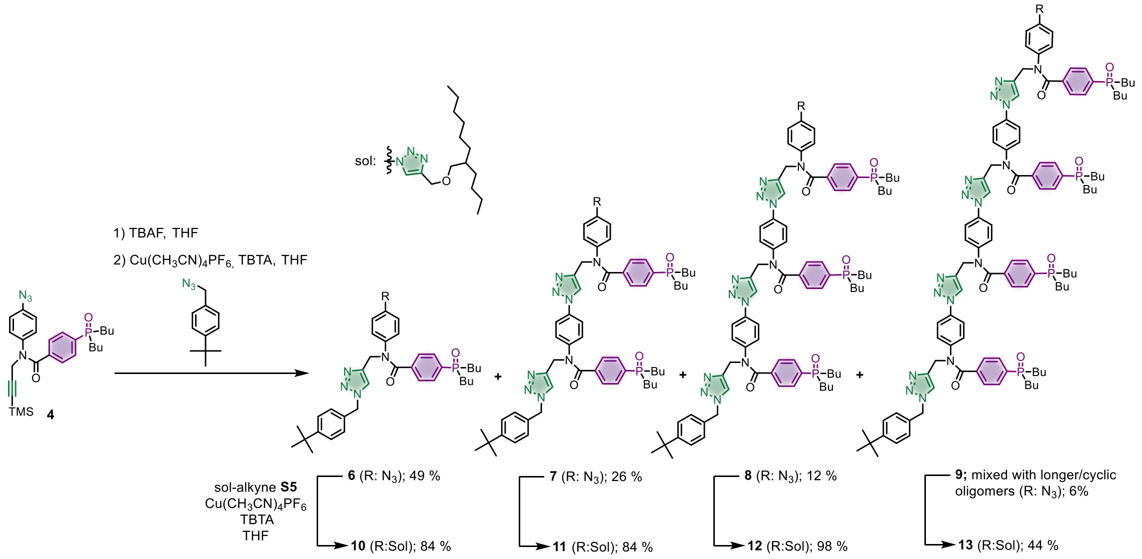

Scheme S3. Synthesis of phosphine oxide oligomers **10**-**13**.

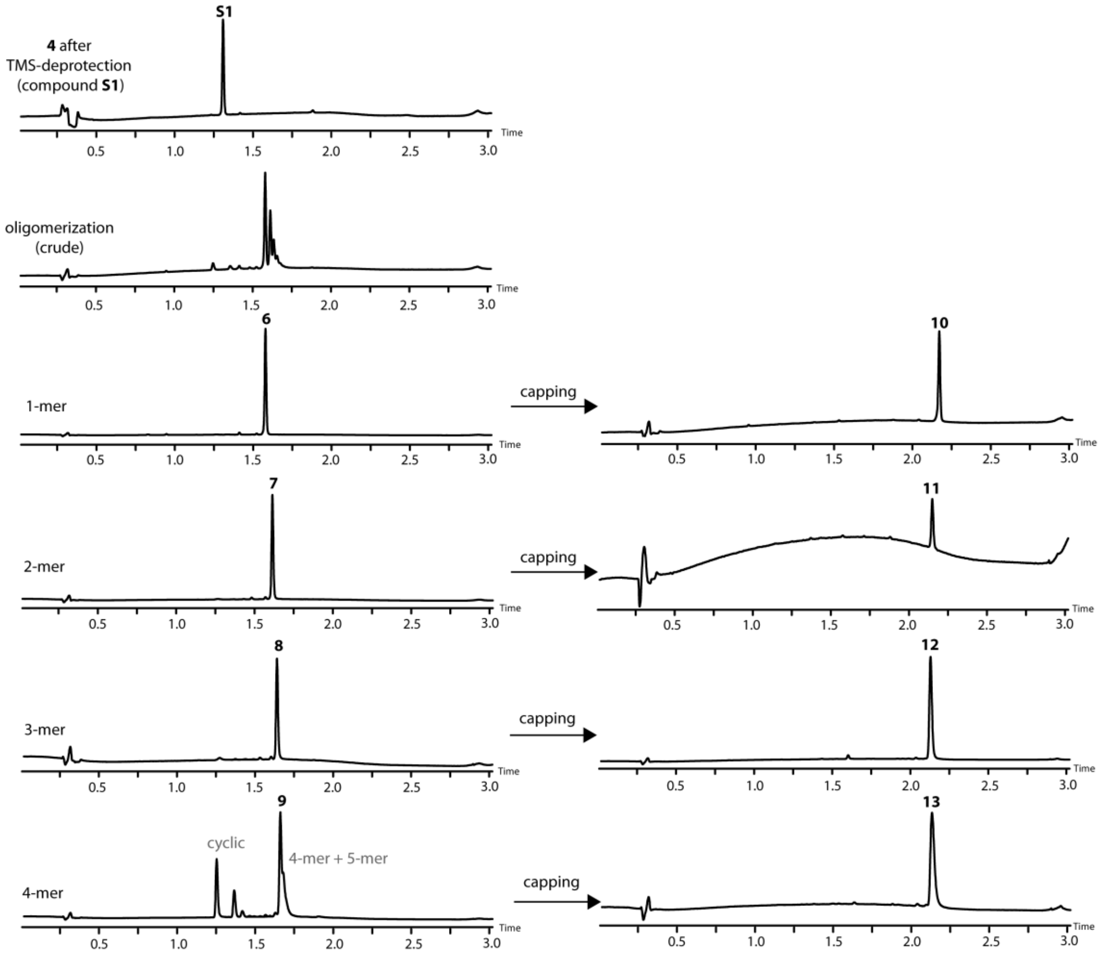

Figure S1. UPLC traces for the end-capping oligomerization of phosphine oxide 1-mer **S1**. *UPLC Conditions*: C18 column at 40 °C (254 nm) using water + 0.1% formic acid (A) and CH_3CN + 0.1% formic acid (B) with flow rate of 0.6 ml/min; Gradient of 0-2 minutes 5%-100% B + 1 minute 100% B.

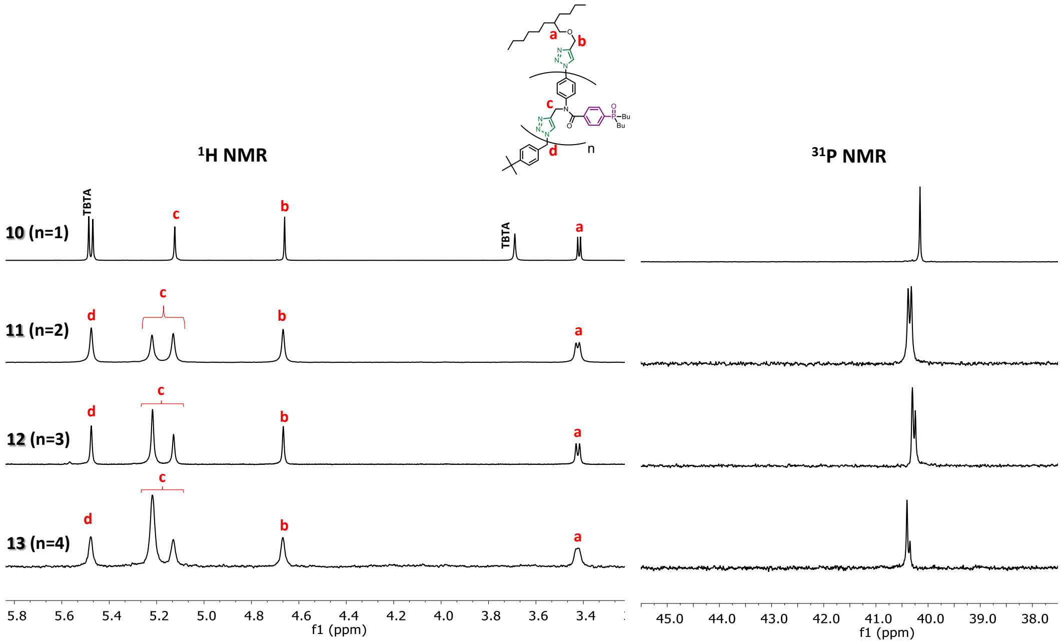

Figure S2. Methylene region of the 400 MHz ^{1}H NMR and full 162 MHz ^{31}P NMR spectra for oligomers **10**-**13** ($CDCl_3$, 298 K).

General procedure for the synthesis of phosphine oxide oligomers

Compound **4** (0.069 g, 0.14 mmol) was dissolved in dry THF (5 mL) and then treated with TBAF solution (1M in THF, 0.33 mL, 0.33 mmol). The reaction was stirred for 10 minutes at room temperature and quenched with 5% soln. HCl and extracted with EtOAc (3x) followed by washing with H_2O and brine. The organic layer was dried over anhydrous $MgSO_4$ and concentrate under vacuum. The obtained residue was purified by flash chromatography (from 50% to 100% of EtOAc in pet. ether) to afford alkyne-deprotected 1-mer **S1** (0.054 g, 91%). **S1** (0.054 g, 0.12 mmol) and 1-(azidomethyl)-4-*tert*-butylbenzene[S1] (0.018 g, 0.09 mmol), were dissolved in dry THF (60 mL) under N_2 atmosphere. $Cu(CH_3CN)_4PF_6$ (0.013 g, 0.04 mmol) and TBTA (0.02 g, 0.04 mmol) were added to the reaction and the solution was stirred at room temperature for 2 days. The reaction was then diluted with EtOAc and washed with EDTA soln. (2x), H_2O (1x) and brine. The organic layer was dried over $MgSO_4$ and concentrate under vacuum. The residue was purified by flash column chromatography on silica gel (gradient from 0% to 20% of MeOH in CH_2Cl_2) to afford azido 1-mer derivative **6** (0.038 g, 49%; containing some TBTA as impurity) as a pale yellow oil, 2-mer derivative **7** (0.017 g, 17%) as a pale yellow amorphous solid, 3-mer derivative **8** (0.008 g, 12%) as a pale yellow oil and 4-mer derivative **9** mixed with longer oligomers (0.009 g, 6%; not fully characterized and used as it was in the subsequent capping step).

Characterization of 1-mer 6

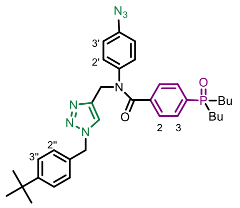

^{1}H NMR (400 MHz, CDCl$_3$): δ_H = 7.63 (s, 1H, CH_{triaz}), 7.50 (m, 2H, 3-H), 7.38 (d, 2H, *J* = 8.5 Hz, 3''-H, ^{t}Bu cap), 7.35 (m, 2H, 2-H), 7.18 (d, 2H, *J* = 8.5 Hz, 2''-H, ^{t}Bu cap), 7.08 (d, 2H, *J* = 8.5 Hz, 2'-H), 6.82 (d, 2H, *J* = 8.5 Hz, 3'-H), 5.47 (s, 2H, N-CH_2, ^{t}Bu cap), 5.08 (s, 2H, N-CH_2, internal), 1.90 (m, 2H, 1''-H, Bu), 1.73 (m, 2H, 1''-H, Bu; overlapped by water peak), 1.49 (m, 2H, 2''-H, Bu), 1.30 (m, 15H, 2''-H and 3''-H, Bu; ^{t}Bu), 0.83 (t, 6H, *J* = 7.0 Hz, 4''-H, Bu).

^{13}C NMR (125.7 MHz, CDCl$_3$): δ_C = 169.9 (CO), 151.9 (4''-C, ^{t}Bu cap), 143.8 (C_{triaz}), 139.9 (1'-C), 138.4 (4'-C), 137.8 (d, *J* = 3.0 Hz, 1-C), 134.9 (d, *J* = 89.5 Hz, 4-C), 131.5 (1''-C, ^{t}Bu cap), 130.1 (d, *J* = 9.0 Hz, 3-C), 129.1 (2'-C), 129.0 (d, *J* = 12.0 Hz, 2-C), 128.0 (2''-C, ^{t}Bu cap), 126.0 (3''-C, ^{t}Bu cap), 123.7 (CH_{triaz}), 119.7 (3'-C), 54.1 (N-CH_2, ^{t}Bu cap), 47.1 (N-CH_2), 34.7 (C, ^{t}Bu), 31.3 (CH_3, ^{t}Bu), 29.4 (d, *J* = 68.5 Hz, 1''-C, Bu), 24.0 (d, *J* = 14.5 Hz, 2''-C, Bu), 23.4 (d, *J* = 3.0 Hz, 3''-C, Bu), 13.6 (4''-C, Bu).

HRMS (ES+): calcd for $C_{35}H_{45}N_7O_2P$ 626.3367 $[M+H]^+$, found 626.3350 $[M+H]^+$.

FT-IR (ATR): ν_{max} 2959, 2932, 2867, 2127, 2097, 1646, 1505, 1456, 1324 and 723 cm^{-1}.

^{1}H-NMR (400 MHz, $CDCl_3$) compound 6

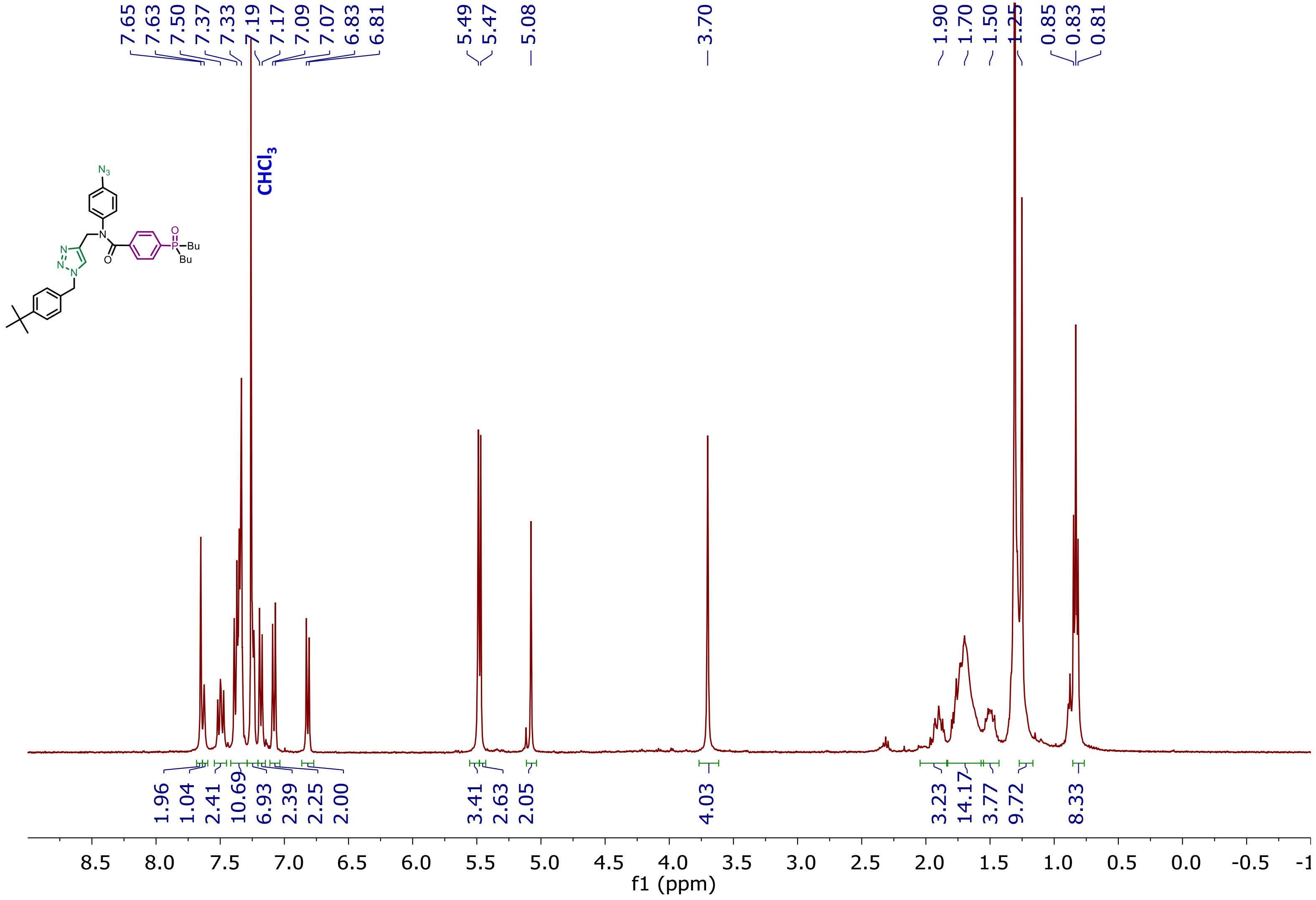

^{13}C-NMR (125.7 MHz, $CDCl_3$) compound 6

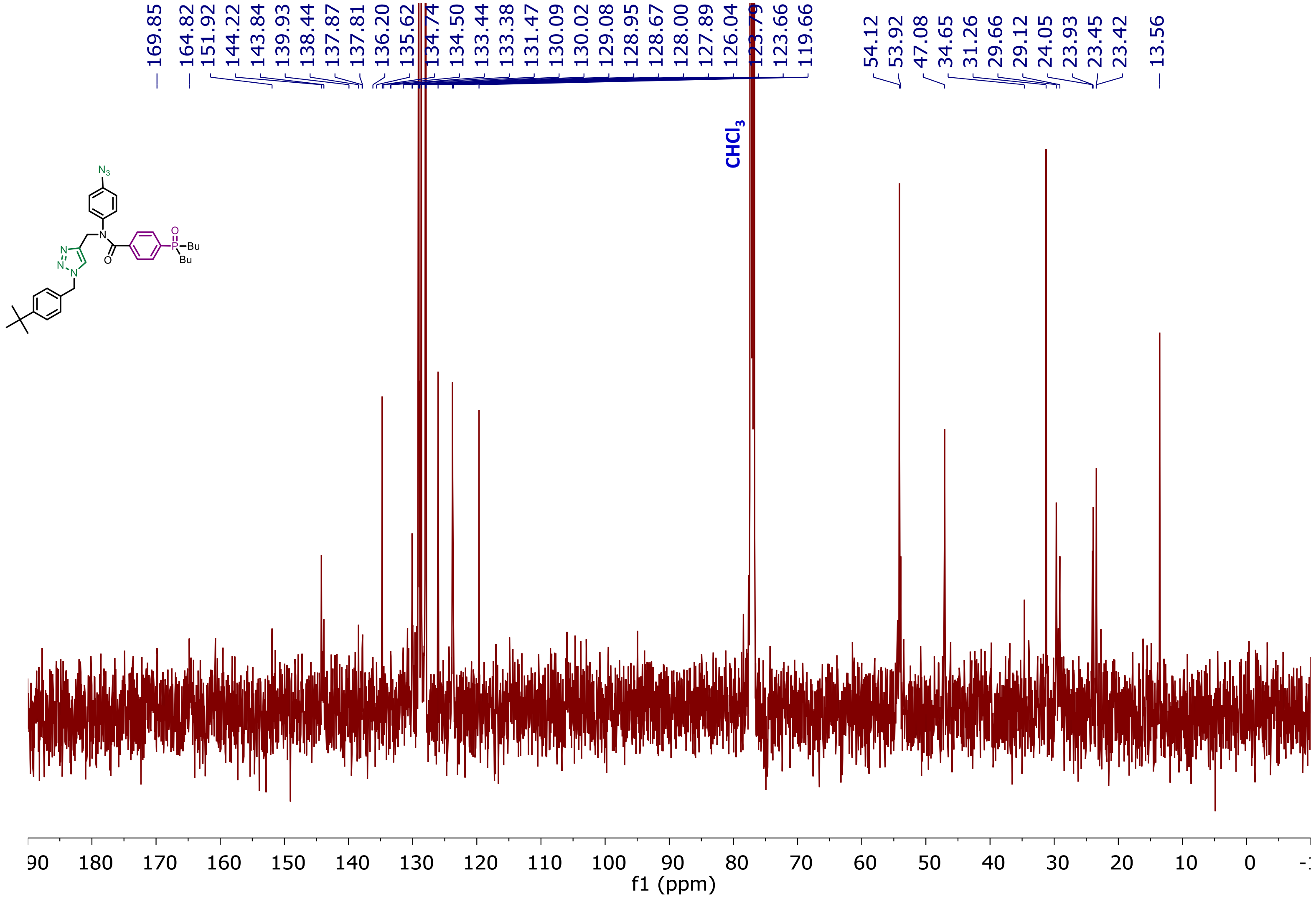

Characterization of 2-mer 7

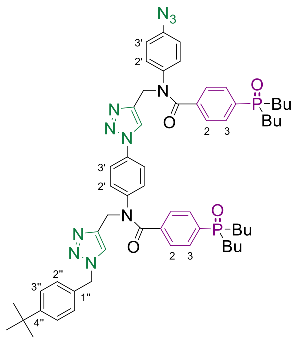

^{1}H NMR (500 MHz, CDCl$_3$): δ_H = 8.15 (s, 1H, CH$_{triaz}$, internal), 7.65 (s, 1H, CH$_{triaz}$, ^{t}Bu cap), 7.61 (d, 2H, J = 9.0 Hz, 3'-H, internal), 7.51 (m, 4H, 3-H), 7.39 (m, 6H, 2-H; 3''-H, ^{t}Bu cap), 7.31 (d, 2H, J = 9.0 Hz, 2'-H, internal), 7.20 (d, 2H, J = 8.5 Hz, 2''-H, ^{t}Bu cap), 7.11 (d, 2H, J = 8.5 Hz, 2'-H, N$_3$ ring), 6.84 (d, 2H, J = 8.5 Hz, 3'-H, N$_3$ ring), 5.47 (s, 2H, N-CH$_2$, ^{t}Bu cap), 5.16 (s, 2H, N-CH$_2$, internal), 5.13 (s, 2H, N-CH$_2$, terminal), 1.90 (m, 4H, 1''-H, Bu), 1.73 (m, 4H, 1''-H, Bu; overlapped by water peak), 1.49 (m, 4H, 2''-H, Bu), 1.30 (m, 21H, 2''-H and 3''-H, Bu; ^{t}Bu), 0.83 (t, 6H, J = 7.0 Hz, 4''-H, Bu), 0.80 (t, 6H, J = 7.0 Hz, 4''-H, Bu).

^{13}C NMR (125.7 MHz, CDCl$_3$): δ_C =169.6 and 169.3 (CO), 152.1 (4''-C, ^{t}Bu cap), 144.6 (C$_{triaz}$, internal), 143.8 (C$_{triaz}$, terminal; 1'-C, internal), 140.0 (1'-C, N$_3$ ring), 139.3 (4'-C, N$_3$ ring), 138.3 (d, J = 3.0 Hz, 1-C), 138.2 (d, J = 3.0 Hz, 1-C), 135.4 (4'-C, internal), 135.3 (d, J = 89.0 Hz, 4-C), 134.9 (d, J = 89.5 Hz, 4-C), 131.5 (1''-C, ^{t}Bu cap), 130.3 (d, J = 9.0 Hz, 3-C), 130.2 (d, J = 9.0 Hz, 3-C), 129.1 (2'-C, N$_3$ ring), 129.0 (d, J = 12.0 Hz, 2-C), 128.9 (d, J = 12.0 Hz, 2-C), 128.8 (2'-C, internal), 128.1 (2''-C, ^{t}Bu cap), 126.2 (3''-C, ^{t}Bu cap), 123.9 (CH$_{triaz}$, ^{t}Bu cap), 121.9 (CH$_{triaz}$, internal), 121.1 (3'-C, internal), 119.9 (3'-C, N$_3$ ring), 54.1 (N-CH$_2$, ^{t}Bu cap), 46.4 and 46.3 (N-CH$_2$), 34.8 (C, ^{t}Bu), 31.4 (CH$_3$, ^{t}Bu), 29.5 (d, J = 68.5 Hz, 1''-C, Bu), 29.5 (d, J = 68.5 Hz, 1''-C, Bu), 24.1 (d, J = 14.5 Hz, 2''-C, Bu), 24.1 (d, J = 14.5 Hz, 2''-C, Bu), 23.6 (d, J = 4.0 Hz, 3''-C, Bu), 13.7 and 13.7 (4''-C, Bu).

^{31}P NMR (161.9 MHz, CDCl$_3$): δ_P = 40.3 and 40.3.

HRMS (ES+): calcd $C_{59}H_{74}N_{11}O_4P_2$ 1062.5395 [M+H]$^+$, found 1062.5405 [M+H]$^+$.

FT-IR (ATR): ν_{max} 2957, 2928, 2871, 2125, 2094, 1645, 1519, 1506, 1395, 1295 and 1168 cm^{-1}.

^{1}H-NMR (500 MHz, $CDCl_3$) compound 7

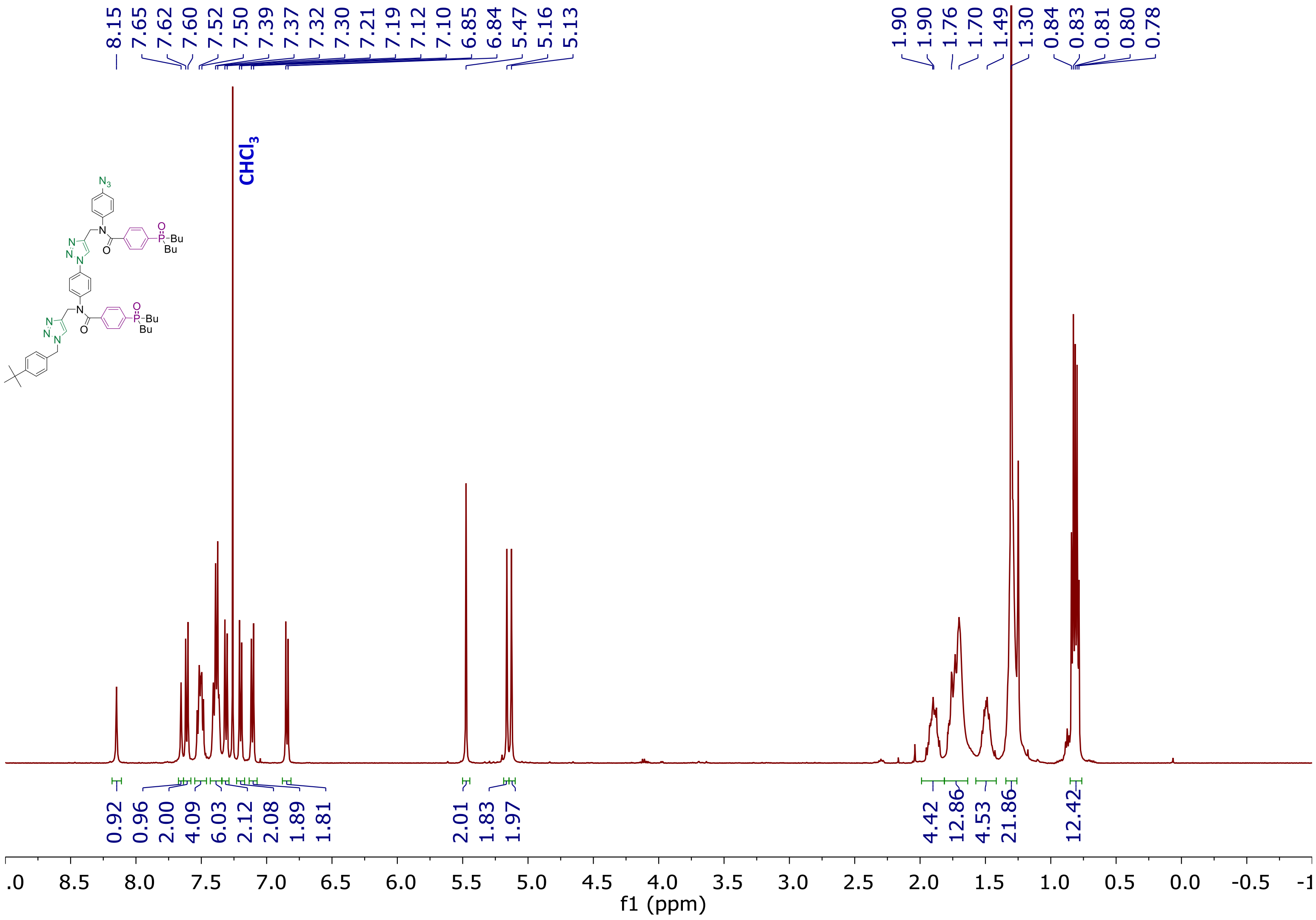

^{13}C-NMR (125.7 MHz, $CDCl_3$) compound 7

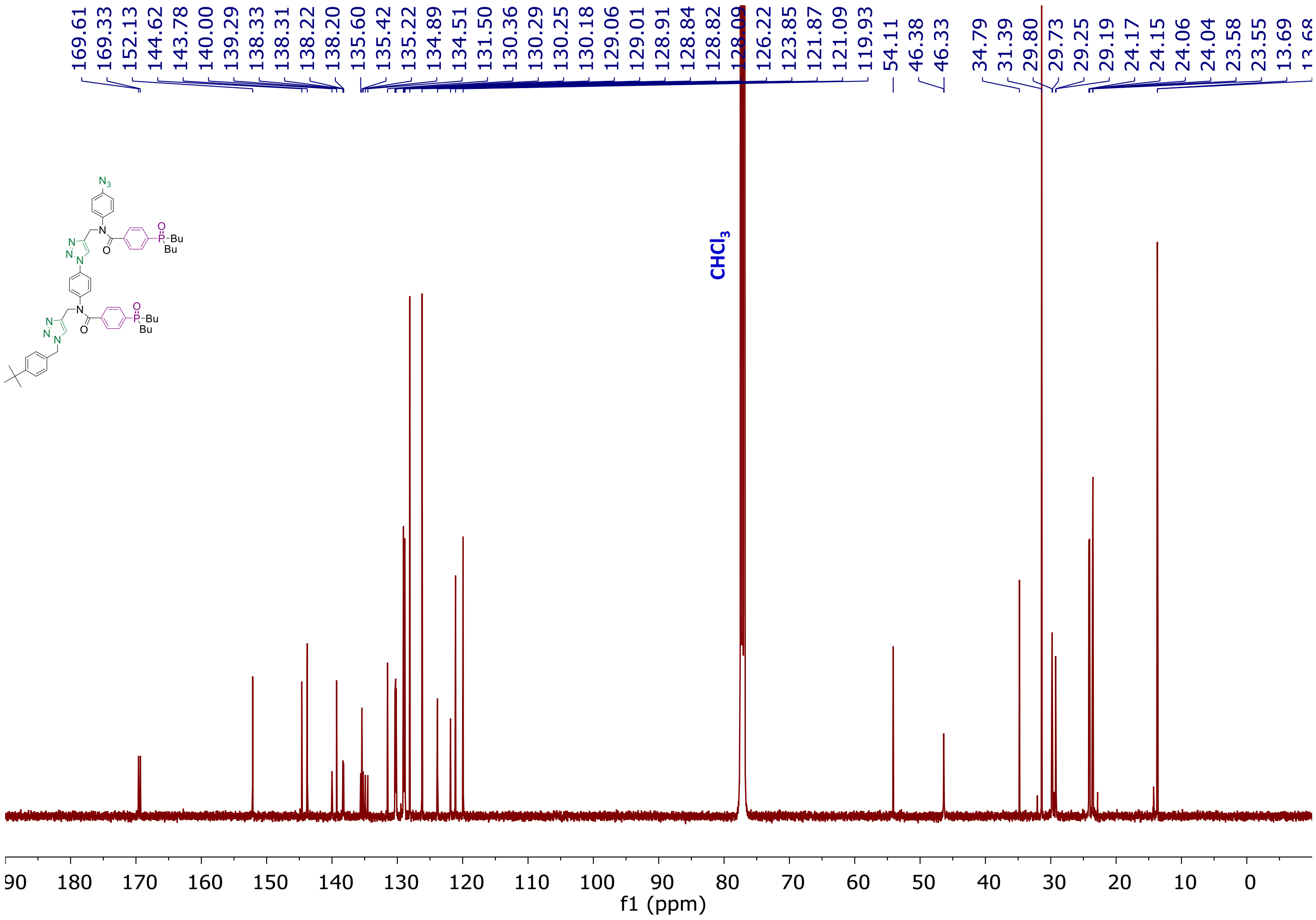

^{31}P NMR (161.9 MHz, CDCl$_3$) compound 7

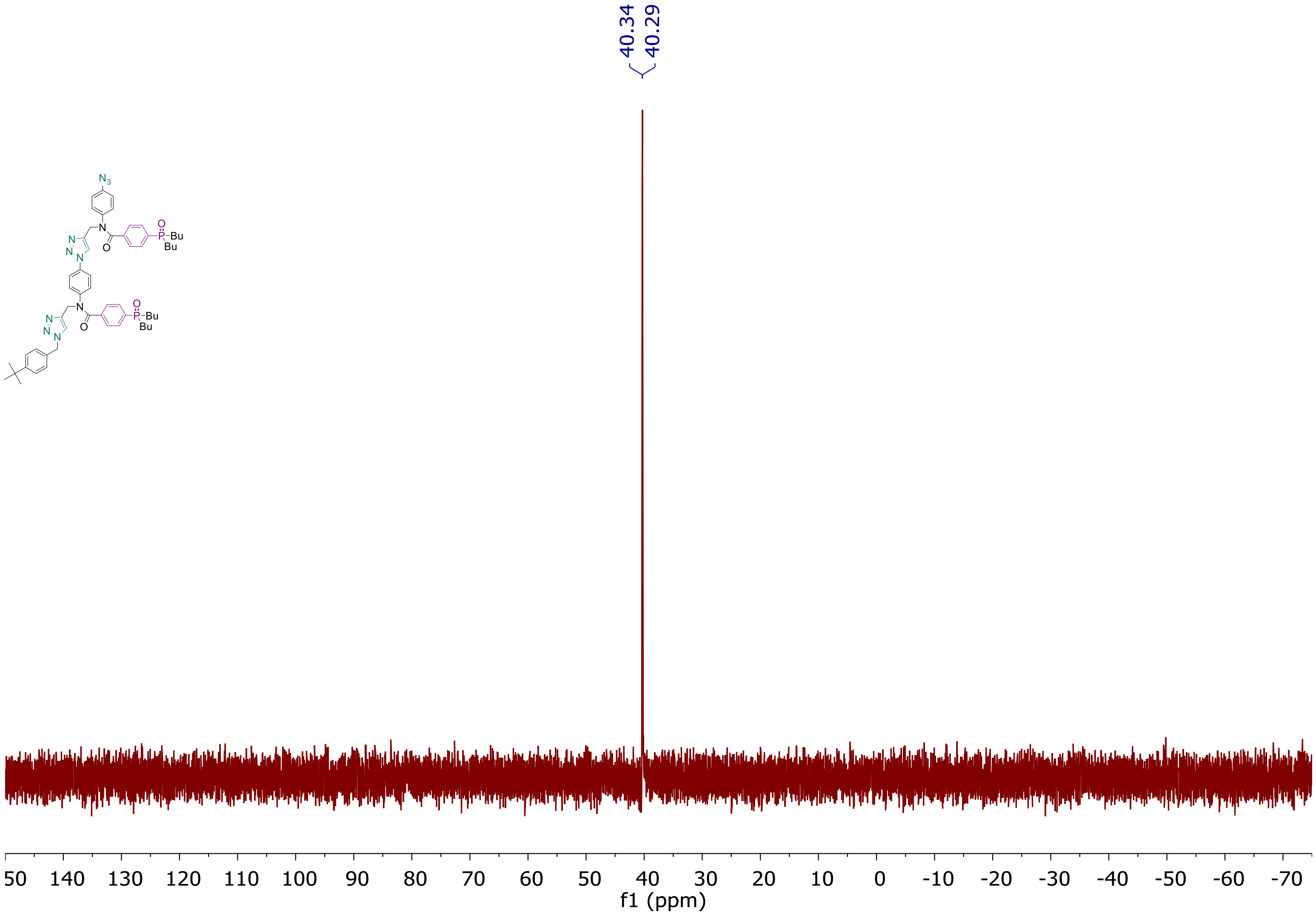

Characterization of 3-mer 8

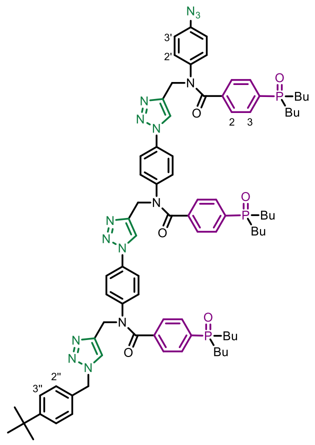

^{1}H NMR (500 MHz, CDCl$_3$): δ_H = 8.17 (s, 1H, CH$_{triaz}$, internal), 8.15 (s, 1H, CH$_{triaz}$, internal), 7.66 (s, 1H, CH$_{triaz}$, ^{t}Bu cap), 7.64 (d, 2H, *J* = 9.0 Hz, 3'-H, internal), 7.62 (d, 2H, *J* = 9.0 Hz, 3'-H, internal), 7.51 (m, 6H, 3-H), 7.40 (m, 8H, 2-H; 3''-H, ^{t}Bu cap), 7.33 (m, 4H, 2'-H, internal), 7.20 (d, 2H, *J* = 8.0 Hz, 2''-H, ^{t}Bu cap), 7.11 (d, 2H, *J* = 8.5 Hz, 2'-H, N$_3$ ring), 6.84 (d, 2H, *J* = 8.5 Hz, 3'-H, N$_3$ ring), 5.48 (s, 2H, N-CH$_2$, ^{t}Bu cap), 5.22 (s, 2H, N-CH$_2$, internal), 5.16 (s, 2H, N-CH$_2$, internal), 5.13 (s, 2H, N-CH$_2$, terminal), 1.89 (m, 6H, 1''-H, Bu), 1.74 (m, 6H, 1''-H, Bu; overlapped by water peak), 1.50 (m, 6H, 2''-H, Bu), 1.30 (m, 27H, 2''-H and 3''-H, Bu; ^{t}Bu), 0.81 (m, 18H, 4''-H, Bu).

^{13}C NMR (125.7 MHz, CDCl$_3$): δ_C = 169.6, 169.5 and 169.3 (CO), 152.1 (4''-C, ^{t}Bu cap), 144.6 and 144.4 (C$_{triaz}$, internal), 143.9 and 143.8 (1'-C, internal), 143.7 (C$_{triaz}$, terminal), 140.01 (1'-C, N$_3$ ring), 139.3 (4'-C, N$_3$ ring), 138.3, 138.2 and 138.0 (d, *J* = 3.0 Hz, 1-C), 135.6 (4'-C, internal), 135.5 (d, *J* = 89.0 Hz, 4-C), 135.4 (4'-C, internal), 135.3 and 134.9 (d, *J* = 89.0 Hz, 4-C), 131.5 (1''-C, ^{t}Bu cap), 130.4, 130.3 and 130.2 (d, *J* = 9.0 Hz, 3-C), 129.1 (2'-C, N$_3$ ring), 129.0, 129.0 and 128.9 (d, *J* = 12.0 Hz, 2-C), 128.9 (d, *J* = 12.0 Hz, 2-C), 128.9 (2'-C, internal),129.0, 128.98, 128.91, 128.86, 128.82, 128.1 (2''-C, ^{t}Bu cap), 126.2 ((3''-C, ^{t}Bu cap), 123.9 (CH$_{triaz}$, ^{t}Bu cap), 122.0 and 121.9 (CH$_{triaz}$, internal), 121.2 and 121.1 (3'-C, internal), 119.9 (3'-C, N$_3$ ring), 54.1 (N-CH$_2$, ^{t}Bu cap), 46.4, 46.4 and 46.3 (N-CH$_2$), 34.8 (C, ^{t}Bu), 31.4 (CH$_3$, ^{t}Bu), 29.5 and 29.5 (d, *J* = 68.5 Hz, 1''-C, Bu), 24.1, 24.1 and 24.1 (d, *J* = 14.5 Hz, 2''-C, Bu), 23.6 (d, *J* = 4.0 Hz, 3''-C, Bu), 13.7 and 13.7 (4''-C, Bu).

^{31}P NMR (202.5 MHz, CDCl$_3$): δ_P = 41.1 and 41.1.

HRMS (ES+): calcd for $C_{83}H_{103}N_{15}O_6P_3$ 1498.7423 [M+H]$^+$, found 1498.7436 [M+H]$^+$.

FT-IR (ATR): ν_{max} 2957, 2928, 2867, 2143, 1651, 1519, 1506, 1396, 1169 and 844 cm^{-1}.

^{1}H-NMR (500 MHz, $CDCl_3$) compound 8

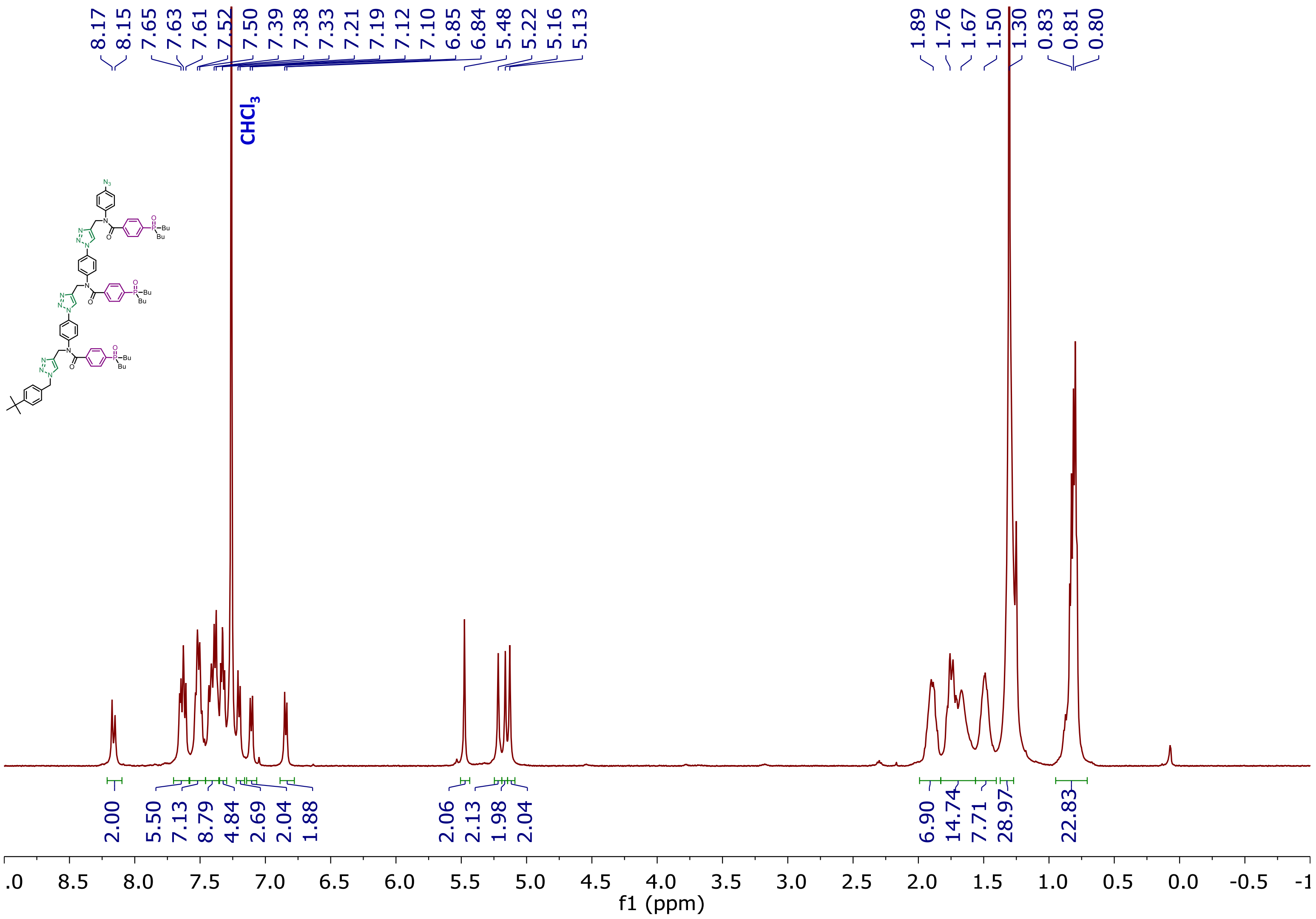

^{13}C-NMR (125.8 MHz, $CDCl_3$) compound 8

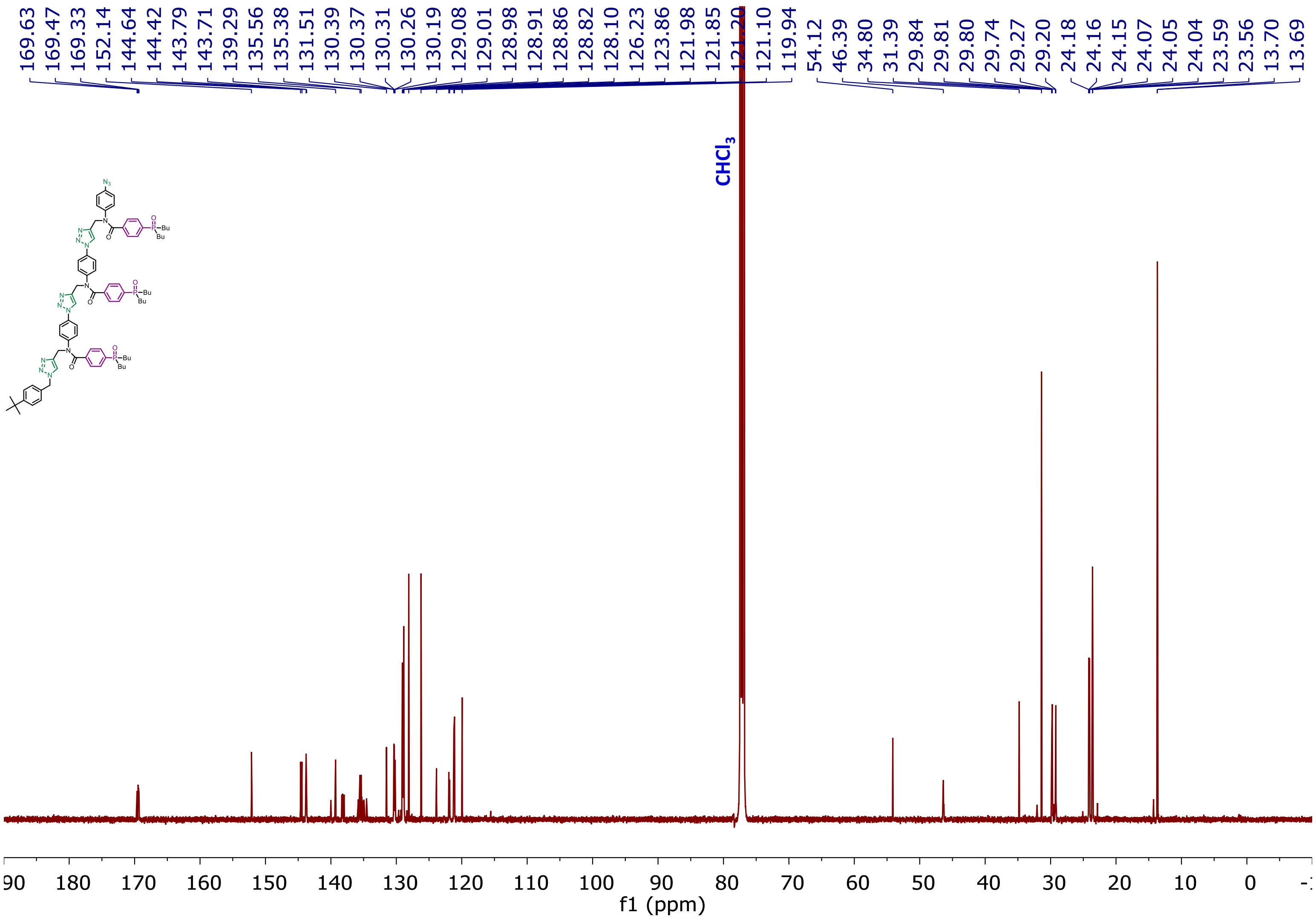

^{31}P NMR (202.5 MHz, $CDCl_3$) compound 8

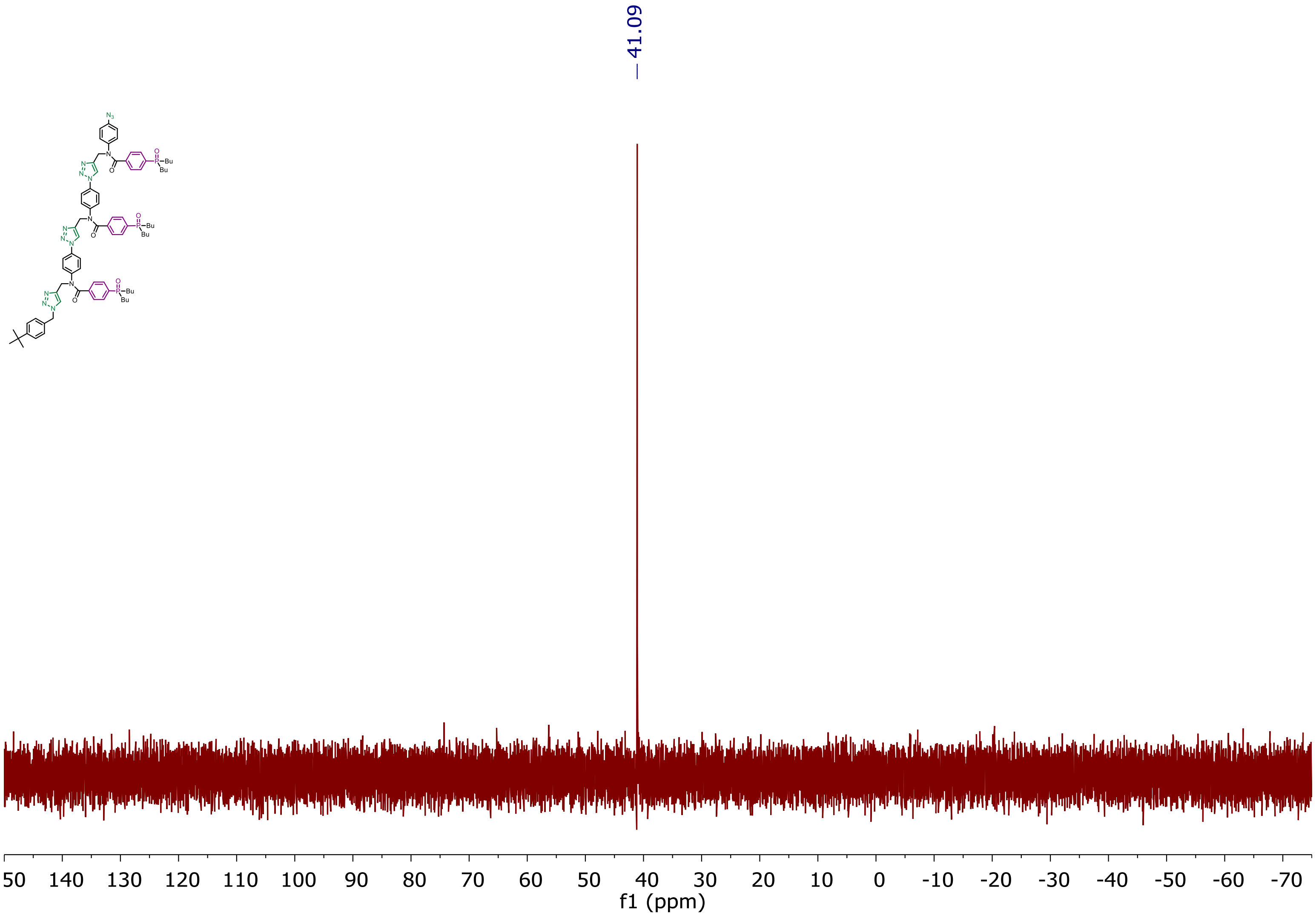

Synthesis of 2-butyloctyl propargyl ether (compound S5)

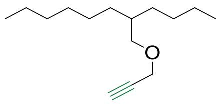

2-Butyl-1-octanol (2.5 g, 13.42 mmol) was dissolved in dry THF (5 mL) and added to a suspension of NaH (60% dispersion in mineral oil, 1.01 g, 26.83 mmol) in THF (20 mL) under inert atmosphere at 0 °C. The reaction was allowed to reach room temperature for 15 min. Propargyl bromide (80% in toluene, 4.00 mL, 26.83 mmol) was added dropwise and the reaction was vigorously stirred overnight. Satd. aq. NH_4Cl was carefully added at 0 °C and the reaction was extracted with EtOAc (3x). The combined organic phase was washed with brine (1x), dried with anhydrous $MgSO_4$, filtered, and the solvents evaporated. The obtained residue was purified by flash chromatography using pet. ether as eluent to afford **S5** (2.05 g, 68%) as a pale yellow oil.

^{1}H NMR (400 MHz, CDCl$_3$): δ_H = 4.12 (d, 2H, *J* = 2.5 Hz, O-CH$_2$-alkyne), 3.39 (d, 2H *J* = 5.8 Hz, O-CH$_2$-alkyl), 2.40 (t, 1H, *J* = 2.5 Hz, CH, alkyne), 1.56 (m, 1H, CH), 1.27 (m, 16H, CH$_2$), 0.89 (m, 6H, CH$_3$).

^{13}C NMR (100.6 MHz, CDCl$_3$): δ_C = 80.4 (C, alkyne), 74.1 (CH, alkyne), 73.5 (O-CH$_2$-alkyl), 58.4 (O-CH$_2$-alkyne), 38.3 (CH), 32.0, 31.5, 31.2, 29.9, 29.2, 26.9, 23.2 and 22.8 (CH$_2$), 14.3 (CH$_3$).

FT-IR (ATR): ν_{max} 3312, 2956, 2925, 2856, 1718, 1466, 1357, 1097, 662 and 624 cm^{-1}.

^{1}H-NMR (400 MHz, $CDCl_3$) compound S5

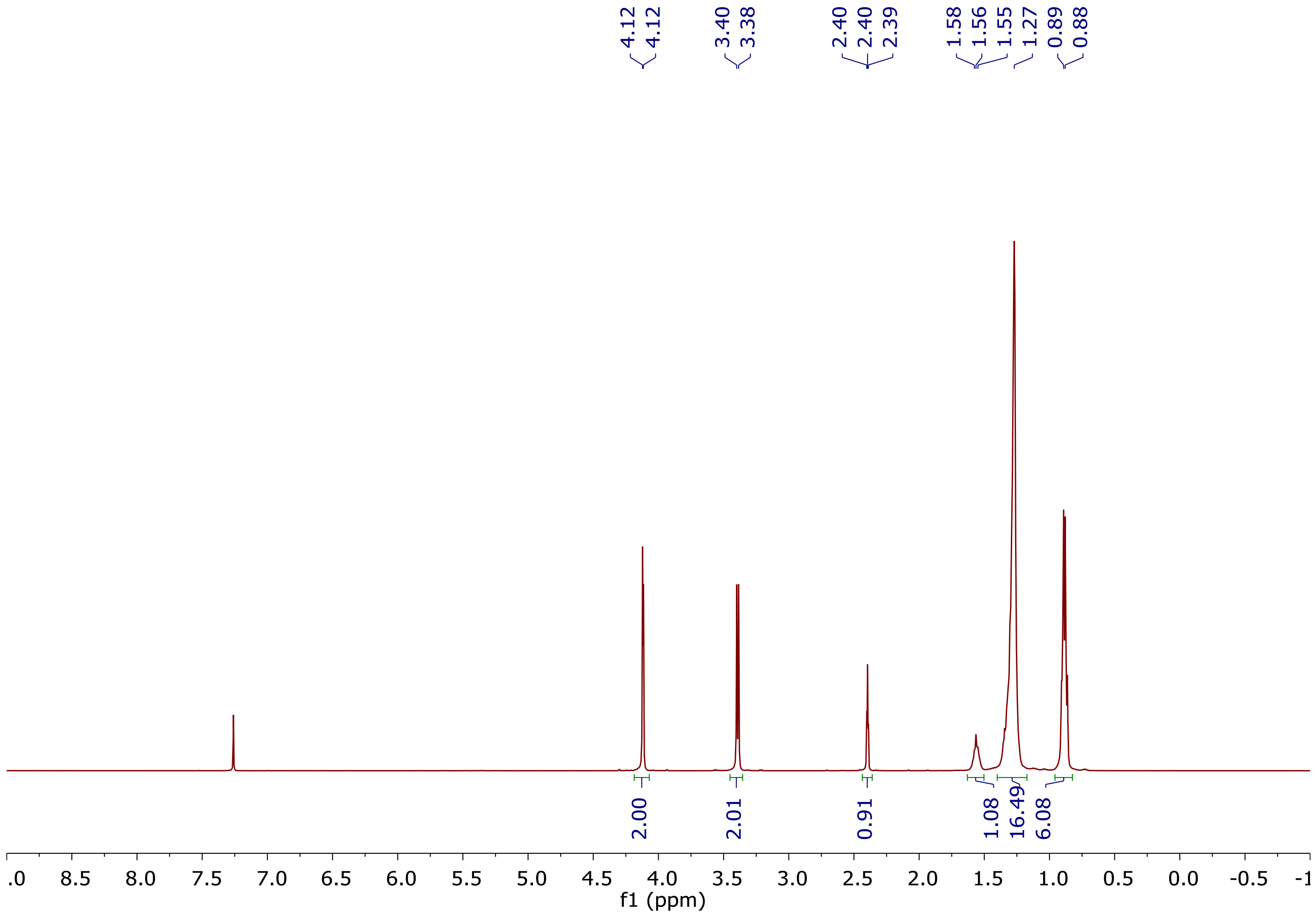

^{13}C-NMR (100.6 MHz, $CDCl_3$) compound S5

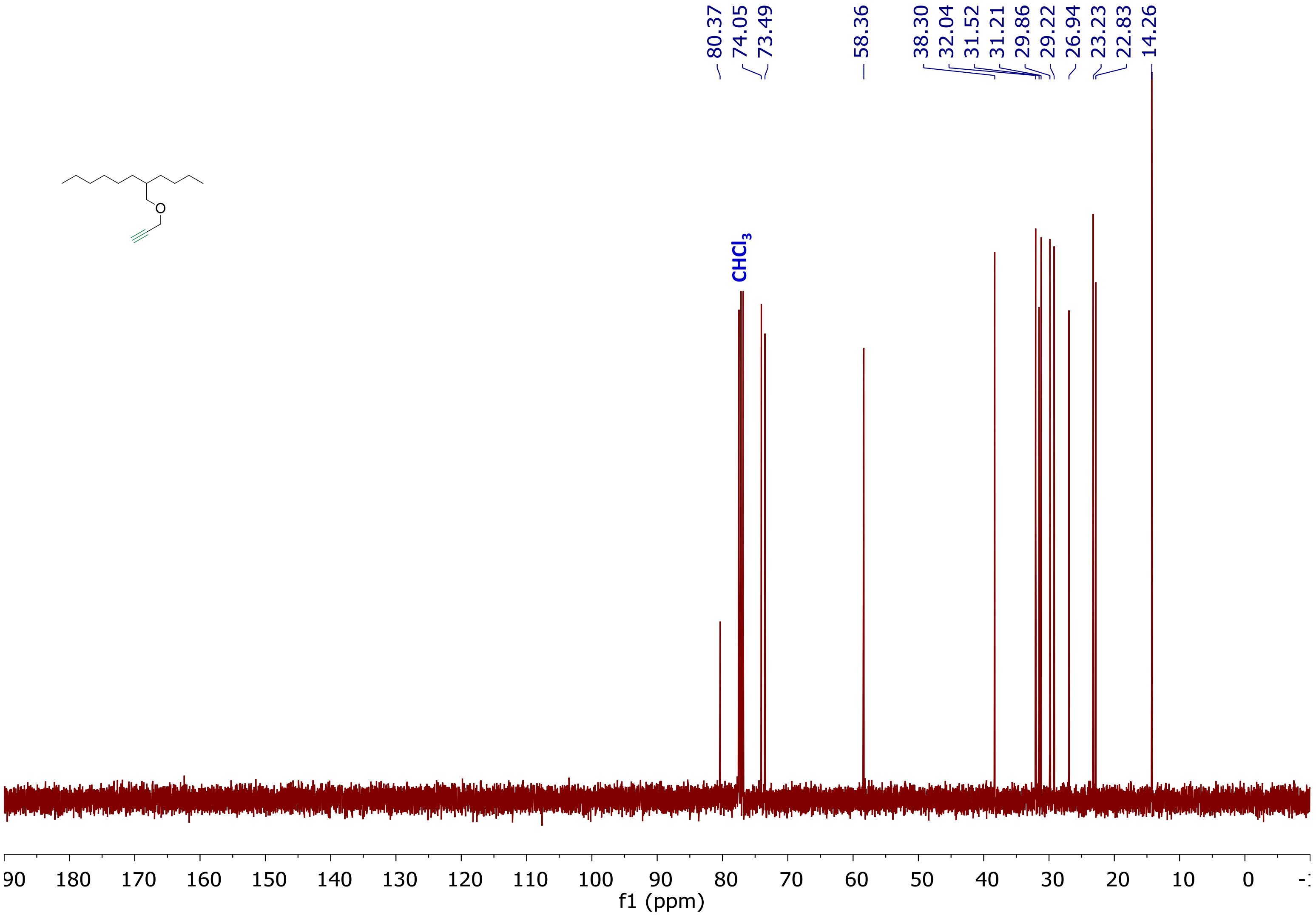

General procedure for the capping of azide-derived phosphine oxide oligomers

The corresponding azido oligomer **6-9** (0.037 g, 0.06 mmol) and **S5** (0.016 g, 0.07 mmol) were dissolved in dry THF (2 mL) under N_2 atmosphere. $Cu(CH_3CN)_4PF_6$ (0.002 g, 0.006 mmol) and TBTA (0.003 g, 0.006 mmol) were added to the reaction and the solution was stirred overnight at room temperature. The reaction was then diluted with EtOAc and washed with EDTA soln. (2x), H_2O (1x) and brine. The organic layer was dried over $MgSO_4$ and concentrate under vacuum. The residue was purified using the method specified in each case.

Characterization of 1-mer 10

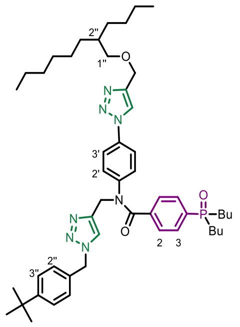

Purification method: Flash column chromatography using a C_{18} reverse phase column (gradient from 5% to 100% of CH_3CN in water) to afford **10** (0.040 g, 80%) as a colorless oil.

^{1}H NMR (500 MHz, CDCl$_3$): δ_H = 7.87 (s, 1H, CH$_{triaz}$, aliphatic end), 7.65 (s, 1H, CH$_{triaz}$, ^{t}Bu end), 7.58 (d, 2H, *J* = 9.0 Hz, 3'-H), 7.51 (dd, 2H, *J* = 10.0, 8.5 Hz, 3-H), 7.40 (m, 2H, 2-H), 7.37 (d, 2H, *J* = 8.5 Hz, 3''-H, ^{t}Bu cap), 7.31 (d, 2H, *J* = 8.5 Hz, 2'-H), 7.19 (d, 2H, *J* = 8.5 Hz, 2''-H, ^{t}Bu cap), 5.47 (s, 2H, N-CH$_2$, ^{t}Bu cap), 5.12 (s, 2H, N-CH$_2$, internal), 4.66 (s, 2H, O-CH$_2$), 3.42 (d, 2H, *J* = 6.0 Hz, 1''-H, aliphatic cap), 1.89 (m, 2H, 1''-H, Bu), 1.74 (m, 2H, 1''-H, Bu), 1.58 (m, 1H, 2''-H, aliphatic cap), 1.48 (m, 2H, 2''-H, Bu), 1.31 (m, 15H, 2''-H and 3''-H, Bu; ^{t}Bu), 1.25 (m, 16H, CH$_2$, aliphatic cap), 0.86 (m, 6H, CH$_3$, aliphatic cap), 0.84 (t, 6H, *J* = 7.0 Hz, 4''-H, Bu).

^{13}C NMR (125.8 MHz, CDCl$_3$): δ_C = 169.3 (CO), 152.1 (4''-C, ^{t}Bu cap), 147.0 (C$_{triaz}$, aliphatic cap), 143.8 (C$_{triaz}$, ^{t}Bu cap), 143.7 (1'-C), 138.2 (d, *J* = 2.5 Hz, 1-C), 135.6 (4'-C), 135.2 (d, *J* = 89.0 Hz, 4-C), 131.5 (1''-C, ^{t}Bu cap), 130.3 (d, *J* = 9.0 Hz, 3-C), 128.9 (d, *J* = 11.0 Hz, 2-C), 128.8 (2'-C), 128.1 (2''-H, ^{t}Bu cap), 126.2 (3''-C, ^{t}Bu cap), 123.8 (CH$_{triaz}$, ^{t}Bu cap), 121.2 (3'-C), 120.3 (CH$_{triaz}$, aliphatic end), 74.4 (1''-C, aliphatic cap), 64.7 (O-CH$_2$), 54.1 (N-CH$_2$, ^{t}Bu cap), 46.4 (N-CH$_2$, internal), 38.4 (2''-C, aliphatic cap), 34.8 (C, ^{t}Bu), 32.0 and 31.5 (CH$_2$, aliphatic cap), 31.4 (CH$_3$, ^{t}Bu), 31.1 and 29.9 (CH$_2$, aliphatic cap), 29.5 (d, *J* = 68.5 Hz, 1''-C, Bu), 29.2 and 26.9 (CH$_2$, aliphatic cap), 24.1 (d, *J* = 14.5 Hz, 2''-C, Bu), 23.6 (d, *J* = 4.0 Hz, 3''-C, Bu), 23.2 and 22.8 (CH$_2$, aliphatic cap), 14.3 (CH$_3$, aliphatic cap), 13.7 (4''-C, Bu).

^{31}P NMR (202.5 MHz, CDCl$_3$): δ_P = 41.2.

HRMS (ES+): calcd for $C_{50}H_{73}N_7O_3P$ 850.5507 [M+H]$^+$, found 850.5492 [M+H]$^+$.

FT-IR (ATR): ν_{max} 2957, 2928, 2858, 1711, 1649, 1519, 1395, 1221, 1104, 1044 and 751 cm^{-1}.

^{1}H-NMR (500 MHz, $CDCl_3$) compound 10

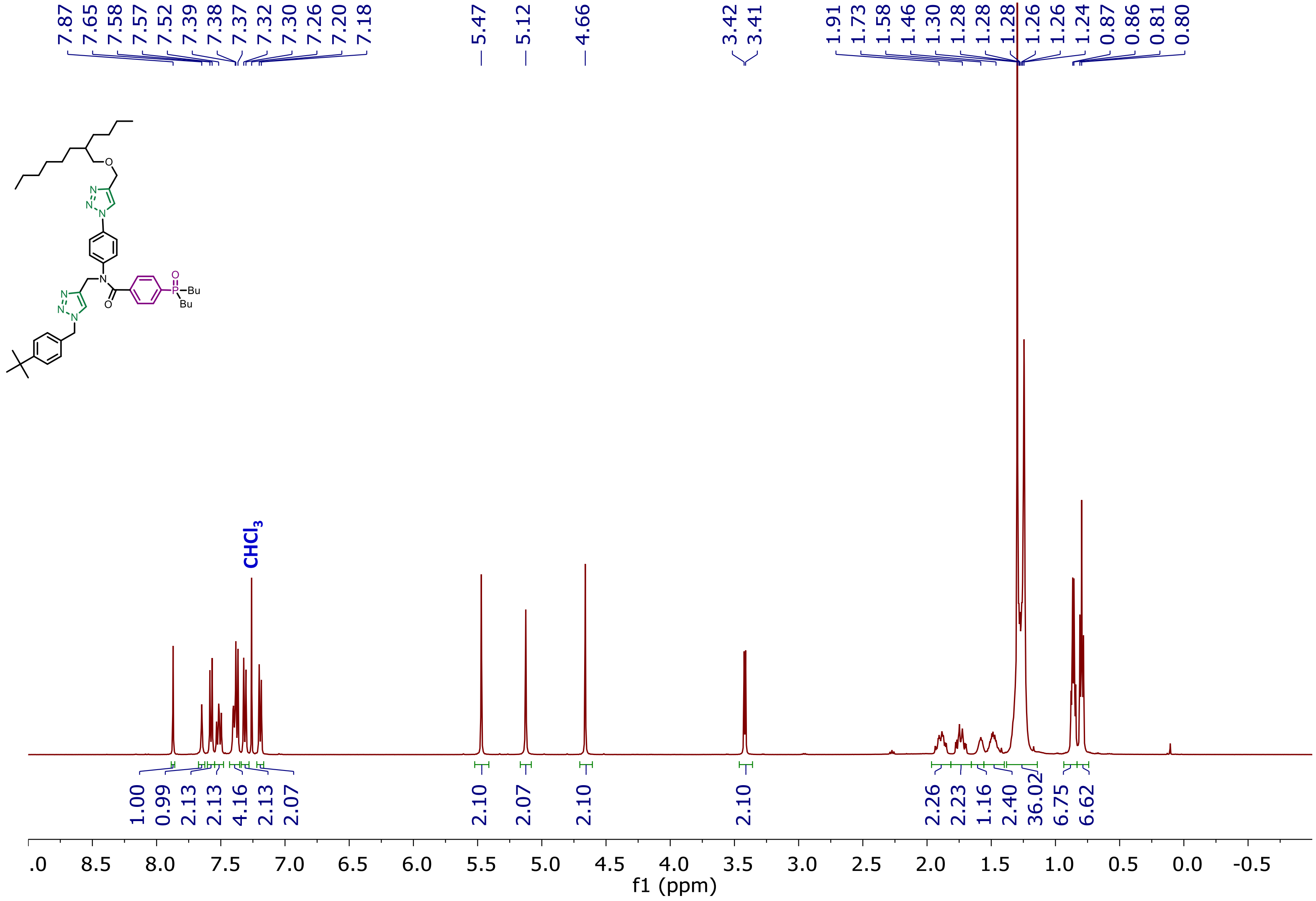

^{13}C-NMR (125.8 MHz, CDCl$_3$) compound 10

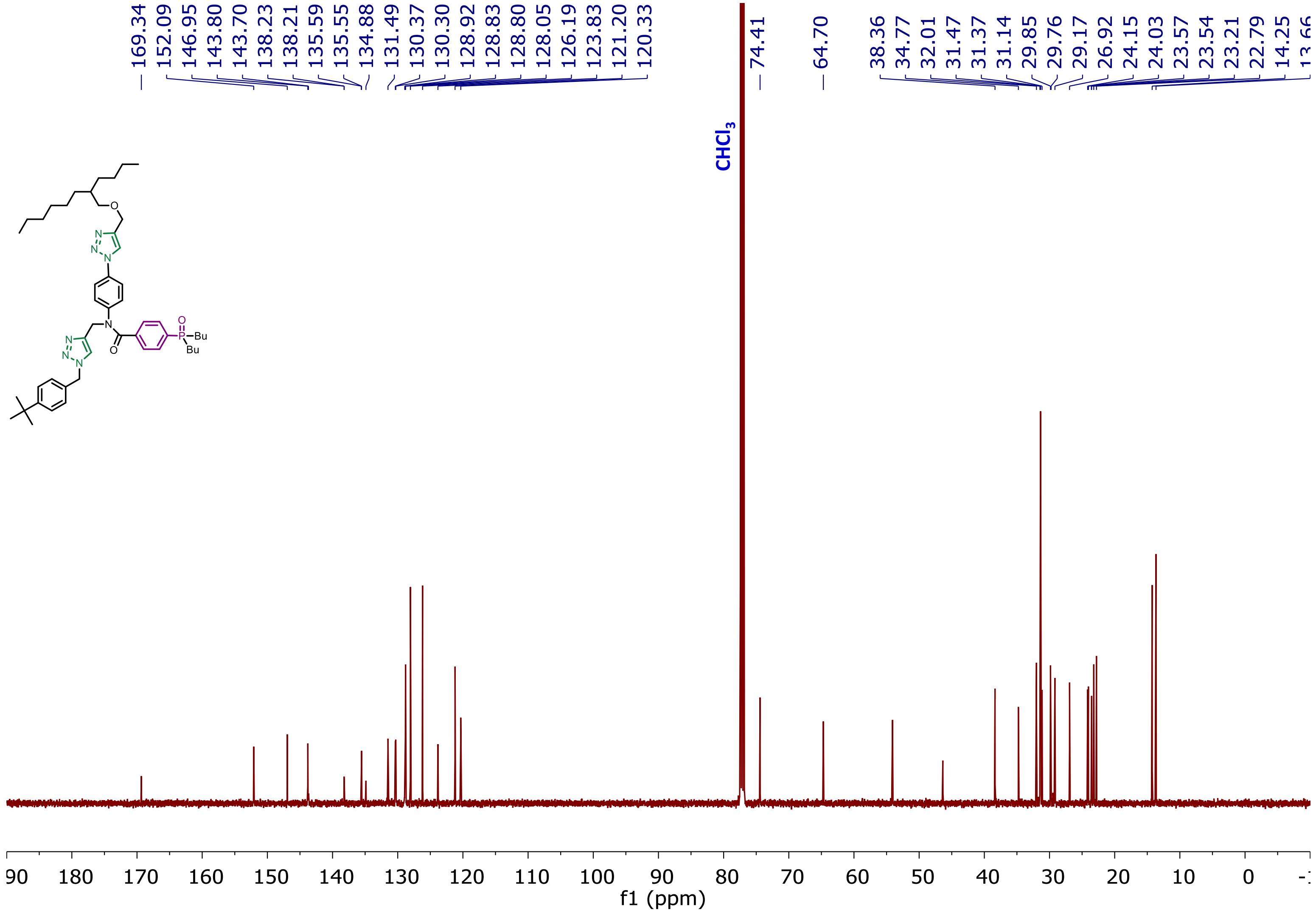

^{31}P NMR (202.5 MHz, $CDCl_3$) compound 10

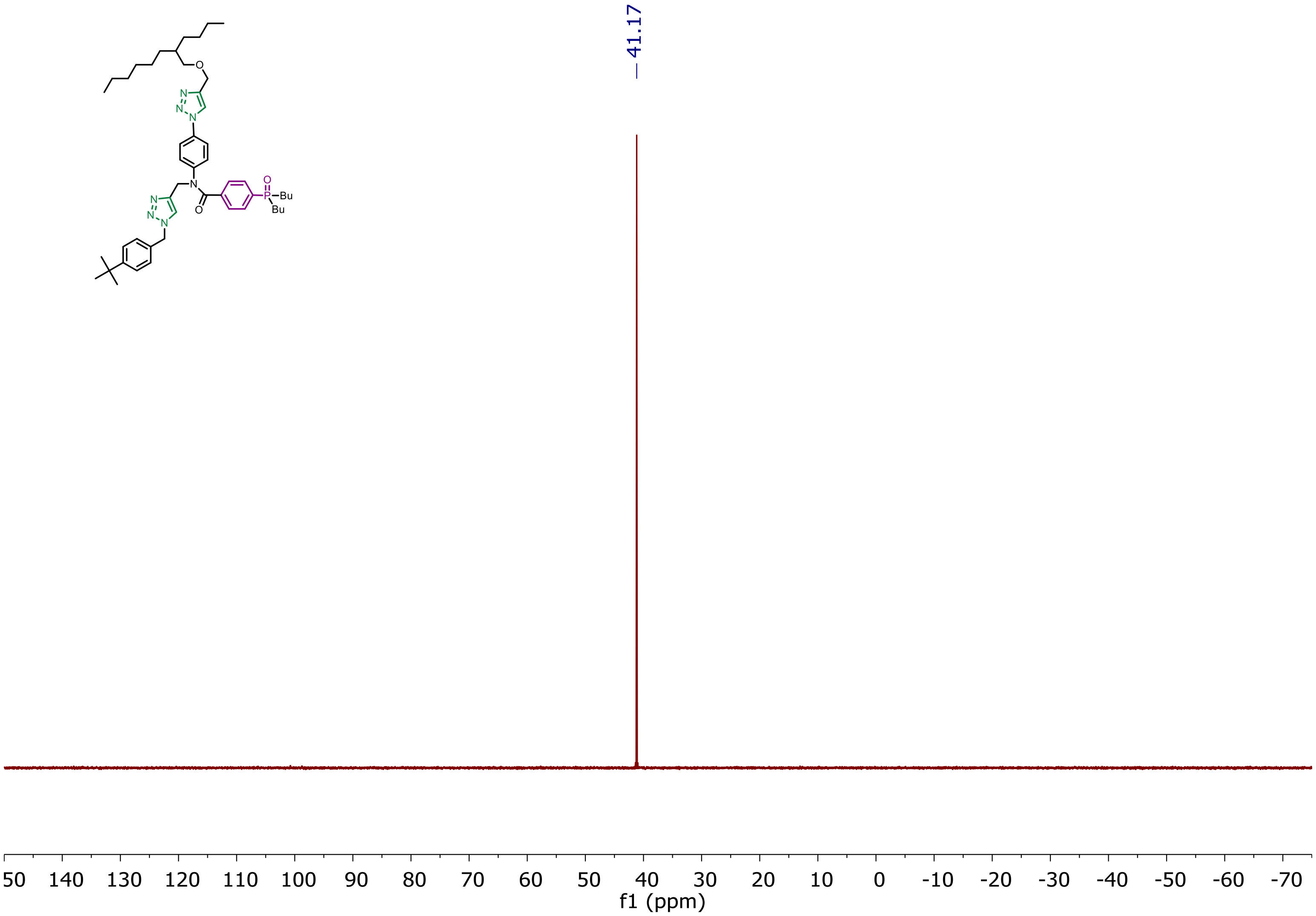

Characterization of 2-mer 11

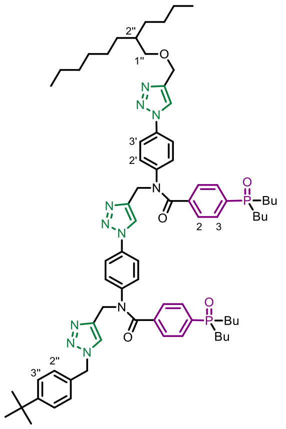

Purification method: Flash column chromatography using silica gel (gradient from 0% to 10% of CH_2Cl_2 in MeOH) to afford **11** (0.006 g, 84%) as a pale yellow oil.

^{1}H NMR (500 MHz, $CDCl_3$): δ_H = 8.17 (s, 1H, CH_{triaz}, internal), 7.88 (s, 1H, CH_{triaz}, aliphatic end), 7.66 (s, 1H, CH_{triaz}, ^{t}Bu end), 7.62 (d, 2H, *J* = 9.0 Hz, 3'-H), 7.61 (d, 2H, *J* = 9.0 Hz, 3'-H), 7.52 (m, 4H, 3-H), 7.42 (m, 4H, 2-H), 7.38 (d, 2H, *J* = 8.5 Hz, 3''-H, ^{t}Bu cap), 7.34 (d, 2H, *J* = 9.0 Hz, 2'-H), 7.32 (d, 2H, *J* = 9.0 Hz, 2'-H), 7.20 (d, 2H, *J* = 8.5 Hz, 2''-H, ^{t}Bu cap), 5.48 (s, 2H, N-CH_2, ^{t}Bu cap), 5.22 (s, 2H, N-CH_2, internal), 5.13 (s, 2H, N-CH_2, internal), 4.67 (s, 2H, O-CH_2), 3.42 (d, 2H, *J* = 6.0 Hz, 1''-H, aliphatic cap), 1.90 (m, 4H, 1''-H, Bu), 1.75 (m, 4H, 1''-H, Bu), 1.58 (m, 1H, 2''-H, aliphatic cap), 1.49 (m, 4H, 2''-H, Bu), 1.31 (m, 21H, 2''-H and 3''-H, Bu; ^{t}Bu), 1.25 (m, 16H, CH_2, aliphatic cap), 0.87 (m, 6H, CH_3, aliphatic cap), 0.80 (t, 12H, *J* = 7.0 Hz, 4''-H, Bu).

^{13}C NMR (125.8 MHz, $CDCl_3$): δ_C = 169.5 and 169.3 (CO), 152.1 (4''-C, ^{t}Bu cap), 147.0 (C_{triaz}, aliphatic cap), 144.5 (C_{triaz}, internal), 143.9 (1'-C), 143.8 (C_{triaz}, ^{t}Bu cap), 143.7 (1'-C), 138.2 and 138.0 (d, *J* = 2.5 Hz, 1-C), 135.7 (4'-C), 135.5 (d, *J* = 89.0 Hz, 4-C), 135.4 (4'-C), 135.3 (d, *J* = 89.0 Hz, 4-C), 131.5 (1''-C, ^{t}Bu cap), 130.4 (d, *J* = 9.0 Hz, 3-C), 130.3 (d, *J* = 9.0 Hz, 3-C), 129.0 (d, *J* = 11.0 Hz, 2-C), 129.0 and 128.8 (2'-C), 128.1 (2''-H, ^{t}Bu cap), 126.2 (3''-C, ^{t}Bu cap), 123.9 (CH_{triaz}, ^{t}Bu end), 122.0 (CH_{triaz}, internal), 121.4 and 121.1 (3'-C), 120.4 (CH_{triaz}, aliphatic end), 74.4 (1''-C, aliphatic cap), 64.7 (O-CH_2), 54.1 (N-CH_2, ^{t}Bu cap), 46.4 and 46.4 (N-CH_2, internal), 38.4 (2''-C, aliphatic cap), 34.8 (C, ^{t}Bu), 32.0 and 31.5 (CH_2, aliphatic cap), 31.4 (CH_3, ^{t}Bu), 31.2 and 29.9 (CH_2, aliphatic cap), 29.5 and 29.5 (d, *J* = 68.5 Hz, 1''-C, Bu), 29.2 and 26.9 (CH_2, aliphatic cap), 24.1 and 24.1 (d, *J* = 14.5 Hz, 2''-C, Bu), 23.6 (d, *J* = 4.0 Hz, 3''-C, Bu), 23.2 and 22.8 (CH_2, aliphatic cap), 14.3 and 14.3 (CH_3, aliphatic cap), 13.7 (4''-C, Bu).

^{31}P NMR (161.7 MHz, $CDCl_3$): δ_P = 40.4 and 40.3.

HRMS (ES+): calcd for $C_{74}H_{102}N_{11}O_5P_2$ 1286.7535 $[M+H]^+$, found 1286.7545 $[M+H]^+$.

FT-IR (ATR): ν_{max} 2956, 2925, 2855, 1712, 1649, 1519, 1362, 1221, 1105 and 1019 cm^{-1}.

^{1}H-NMR (500 MHz, $CDCl_3$) compound 11

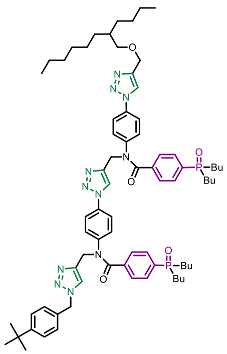

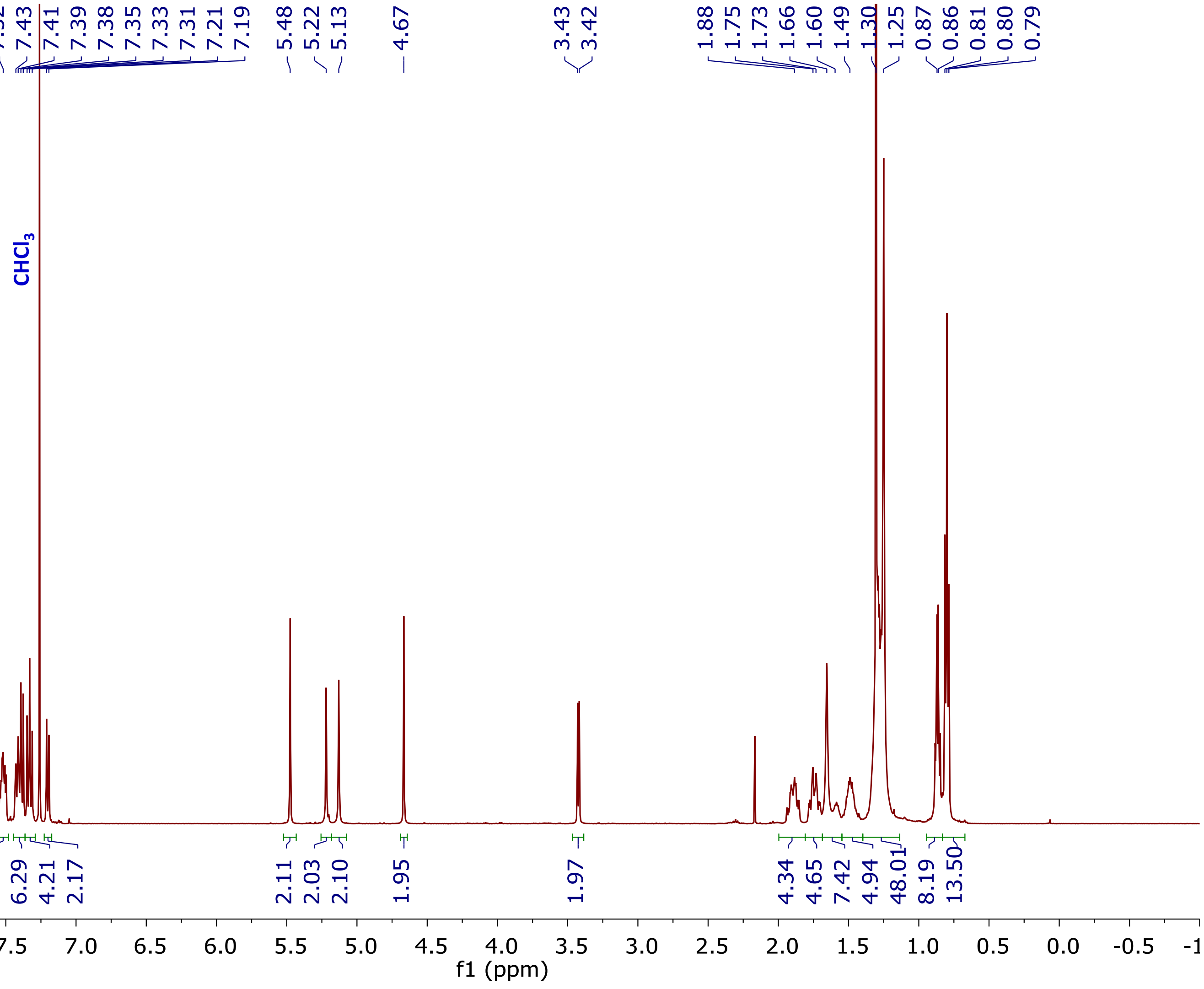

^{13}C-NMR (125.8 MHz, $CDCl_3$) compound 11

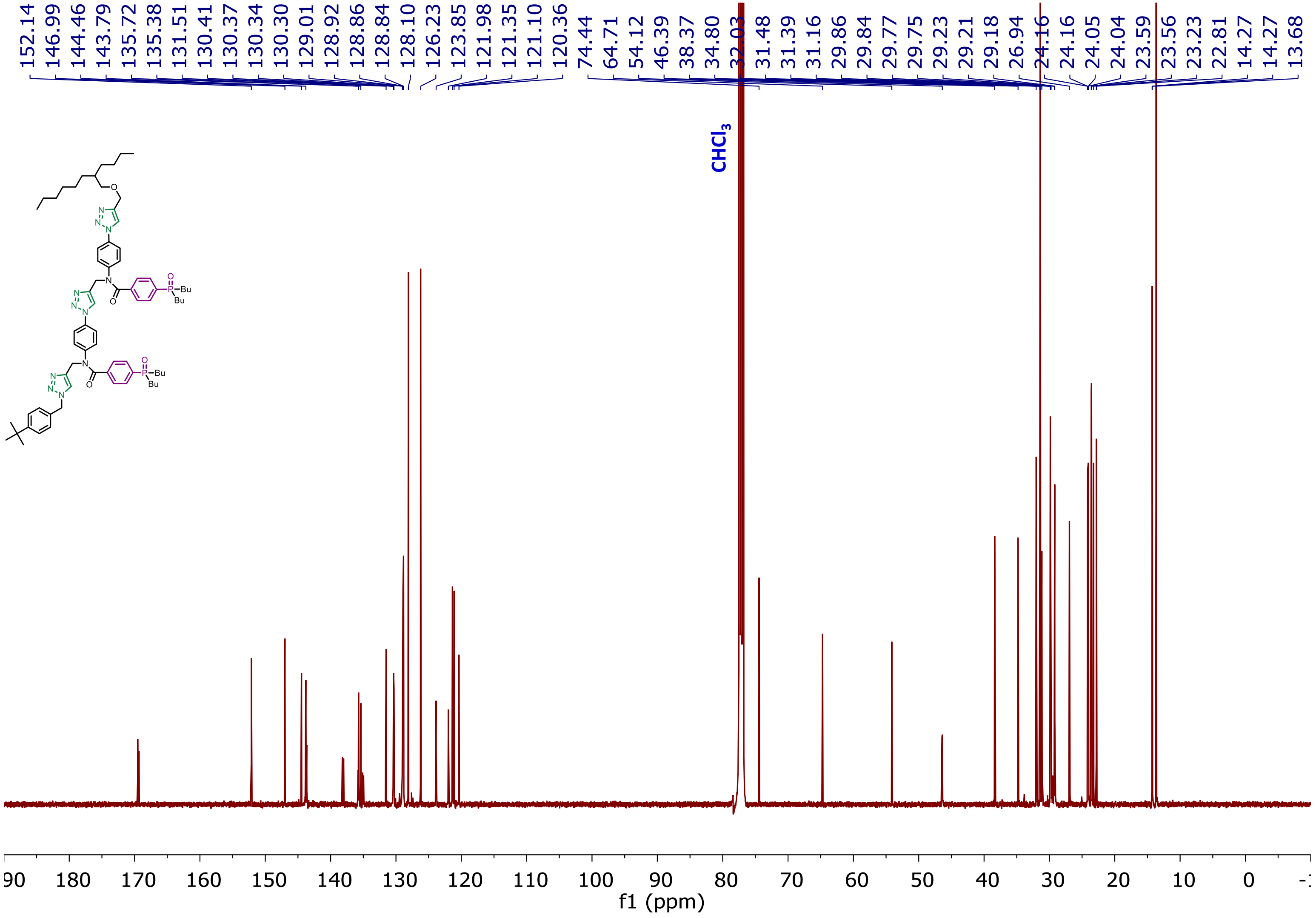

^{31}P NMR (161.7 MHz, CDCl$_3$) compound 11

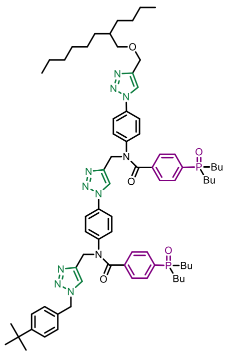

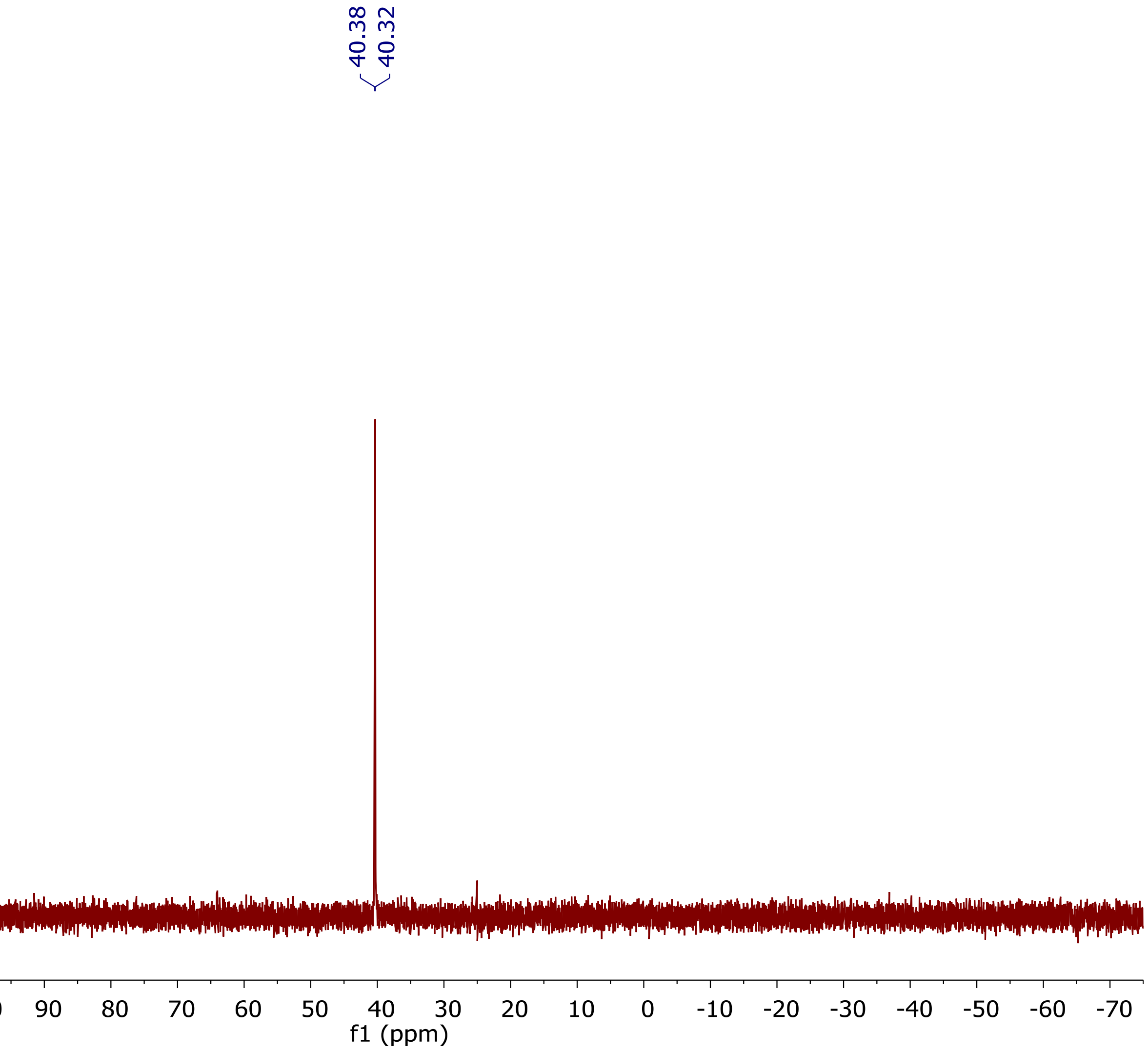

Characterization of 3-mer 12

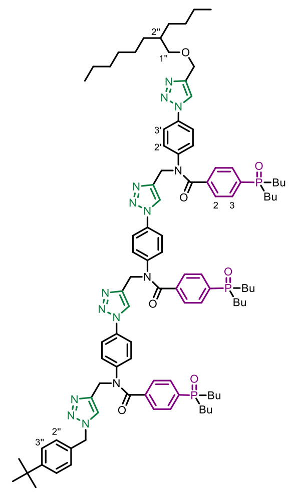

Purification method: Flash column chromatography using silica gel (gradient from 0% to 10% of CH_2Cl_2 in MeOH) to afford **12** (0.009 g, 98%) as a pale yellow oil.

^{1}H NMR (500 MHz, CDCl$_3$): δ_H = 8.18 (s, 1H, CH$_{triaz}$, internal), 8.17 (s, 1H, CH$_{triaz}$, internal), 7.88 (s, 1H, CH$_{triaz}$, aliph. end), 7.65 (s, 1H, CH$_{triaz}$, ^{t}Bu end), 7.64 (d, 2H, *J* = 9.0 Hz, 3’-H), 7.61 (d, 2H, *J* = 9.0 Hz, 3’-H), 7.61 (d, 2H, *J* = 9.0 Hz, 3’-H), 7.52 (m, 6H, 3-H), 7.42 (m, 6H, 2-H), 7.38 (d, 2H, *J* = 8.5 Hz, 3’’-H, ^{t}Bu cap), 7.33 (m, 6H, 2’-H), 7.20 (d, 2H, *J* = 8.5 Hz, 2’’-H, ^{t}Bu cap), 5.47 (s, 2H, N-CH$_2$, ^{t}Bu cap), 5.21 (s, 4H, N-CH$_2$, internal), 5.13 (s, 2H, N-CH$_2$, internal), 4.66 (s, 2H, O-CH$_2$), 3.42 (d, 2H, *J* = 6.0 Hz, 1’’-H, aliph. cap), 1.90 (m, 6H, 1’’-H, Bu), 1.74 (m, 6H, 1’’-H, Bu), 1.58 (m, 1H, 2’’-H, aliph. cap), 1.49 (m, 6H, 2’’-H, Bu), 1.31 (m, 27H, 2’’-H and 3’’-H, Bu; ^{t}Bu), 1.25 (m, 16H, CH$_2$, aliph. cap), 0.86 (m, 6H, CH$_3$, aliph. cap), 0.80 (t, 18H, *J* = 7.0 Hz, 4’’-H, Bu).

^{13}C NMR (125.8 MHz, CDCl$_3$): δ_C = 169.5, 169.5 and 169.3 (CO), 152.1 (4’’-C, ^{t}Bu cap), 147.0 (C$_{triaz}$, aliph. cap), 144.5 and 144.4 (C$_{triaz}$, internal), 143.9 and 143.9 (1’-C), 143.8 (C$_{triaz}$, ^{t}Bu cap), 143.7 (1’-C), 138.2, 138.0 and 137.9 (d, *J* = 2.5 Hz, 1-C), 135.7 and 135.5 (4’-C), 135.5 and 135.5 (d, *J* = 89.0 Hz, 4-C), 135.4 (4’-C), 135.3 (d, *J* = 89.0 Hz, 4-C), 131.5 (1’’-C, ^{t}Bu cap), 130.7, 130.3 and 130.3 (d, *J* = 9.0 Hz, 3-C), 129.0, 129.0 and 129.0 (d, *J* = 11.0 Hz, 2-C), 128.9, 128.9 and 128.8 (2’-C), 128.1 (2’’-H, ^{t}Bu cap), 126.2 (3’’-C, ^{t}Bu cap), 123.9 (CH$_{triaz}$, ^{t}Bu cap), 122.0 (CH$_{triaz}$, internal), 121.3, 121.2 and 121.1 (3’-C), 120.4 (CH$_{triaz}$, aliph. end), 74.4 (1’’-C, aliph. cap), 64.7 (O-CH$_2$), 54.1 (N-CH$_2$, ^{t}Bu cap), 46.4 and 46.4 (N-CH$_2$, internal), 38.4 (2’’-C, aliph. cap), 34.8 (C, ^{t}Bu), 32.0 and 31.5 (CH$_2$, aliph. cap), 31.4 (CH$_3$, ^{t}Bu), 31.2 and 29.9 (CH$_2$, aliph. cap), 29.5, 29.5 and 29.5 (d, *J* = 68.5 Hz, 1’’-C, Bu), 29.2 and 26.9 (CH$_2$, aliph. cap), 24.1 (d, *J* = 14.5 Hz, 2’’-C, Bu), 23.6 (d, *J* = 4.0 Hz, 3’’-C, Bu), 23.2 and 22.8 (CH$_2$, aliph. cap), 14.3 and 14.3 (CH$_3$, aliph. cap), 13.7 (4’’-C, Bu).

^{31}P NMR (162.0 MHz, CDCl$_3$): δ_P = 40.3 and 40.2.

HRMS (ES+): calcd for $C_{98}H_{131}N_{15}O_7P_3$ 1722.9563 [M+H]$^+$, found 1722.9521 [M+H]$^+$.

FT-IR (ATR): ν_{max} 2957, 2926, 2871, 2856, 1711, 1650, 1519, 1362, 1222, 1106 and 848 cm^{-1}.

^{1}H-NMR (500 MHz, $CDCl_3$) compound 12

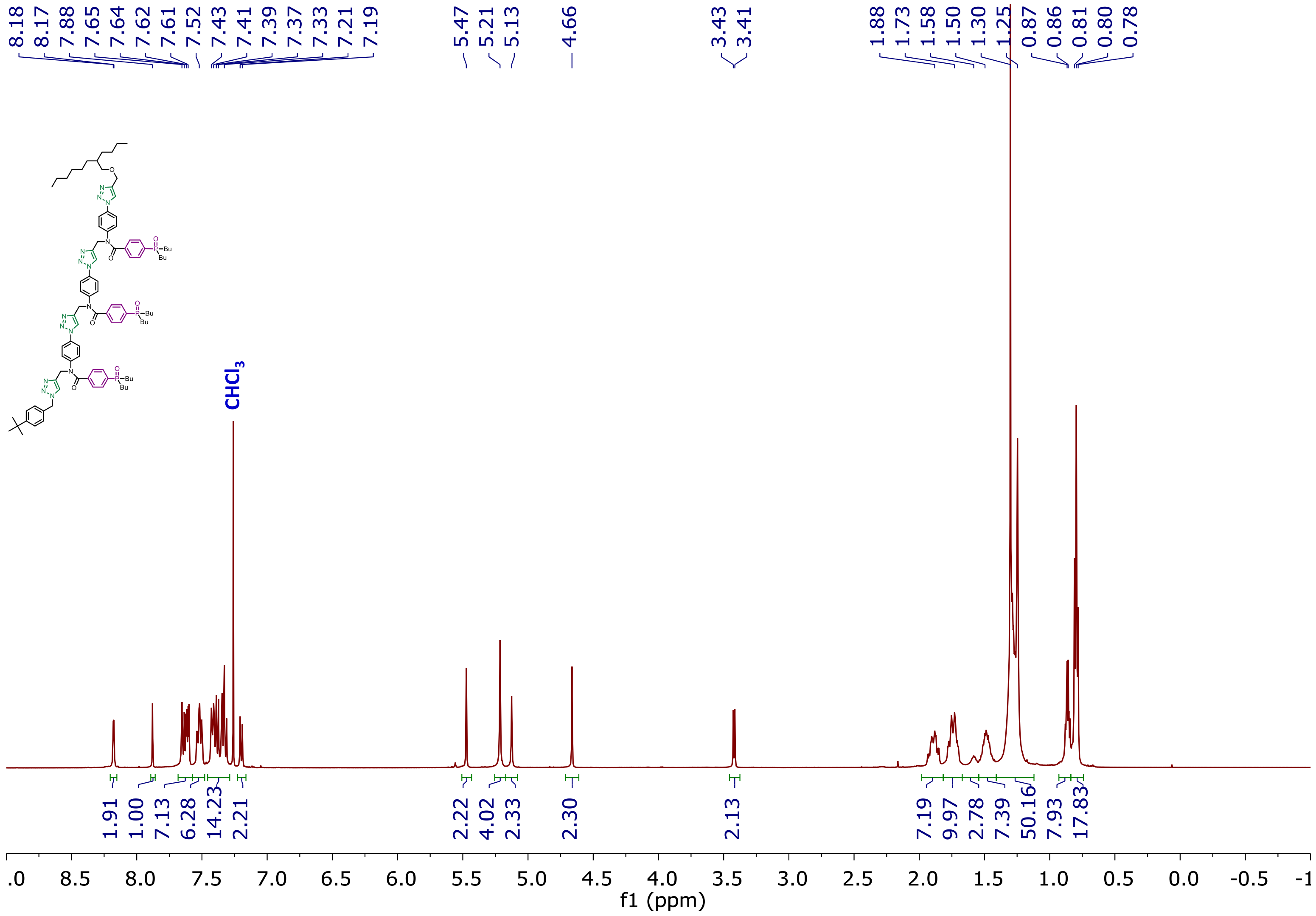

^{13}C-NMR (125.8 MHz, $CDCl_3$) compound 12

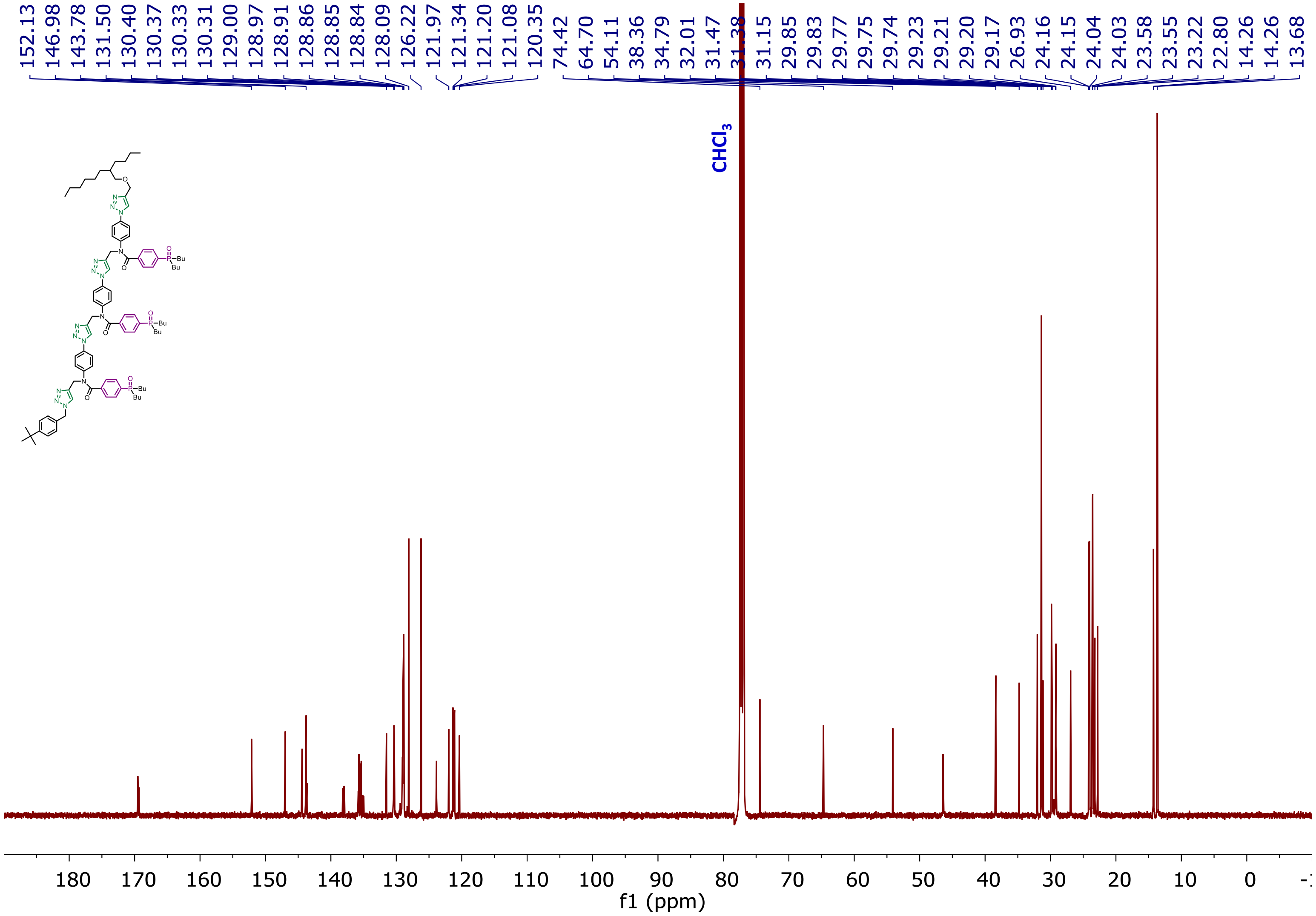

^{31}P NMR (162.0 MHz, $CDCl_3$) compound 12

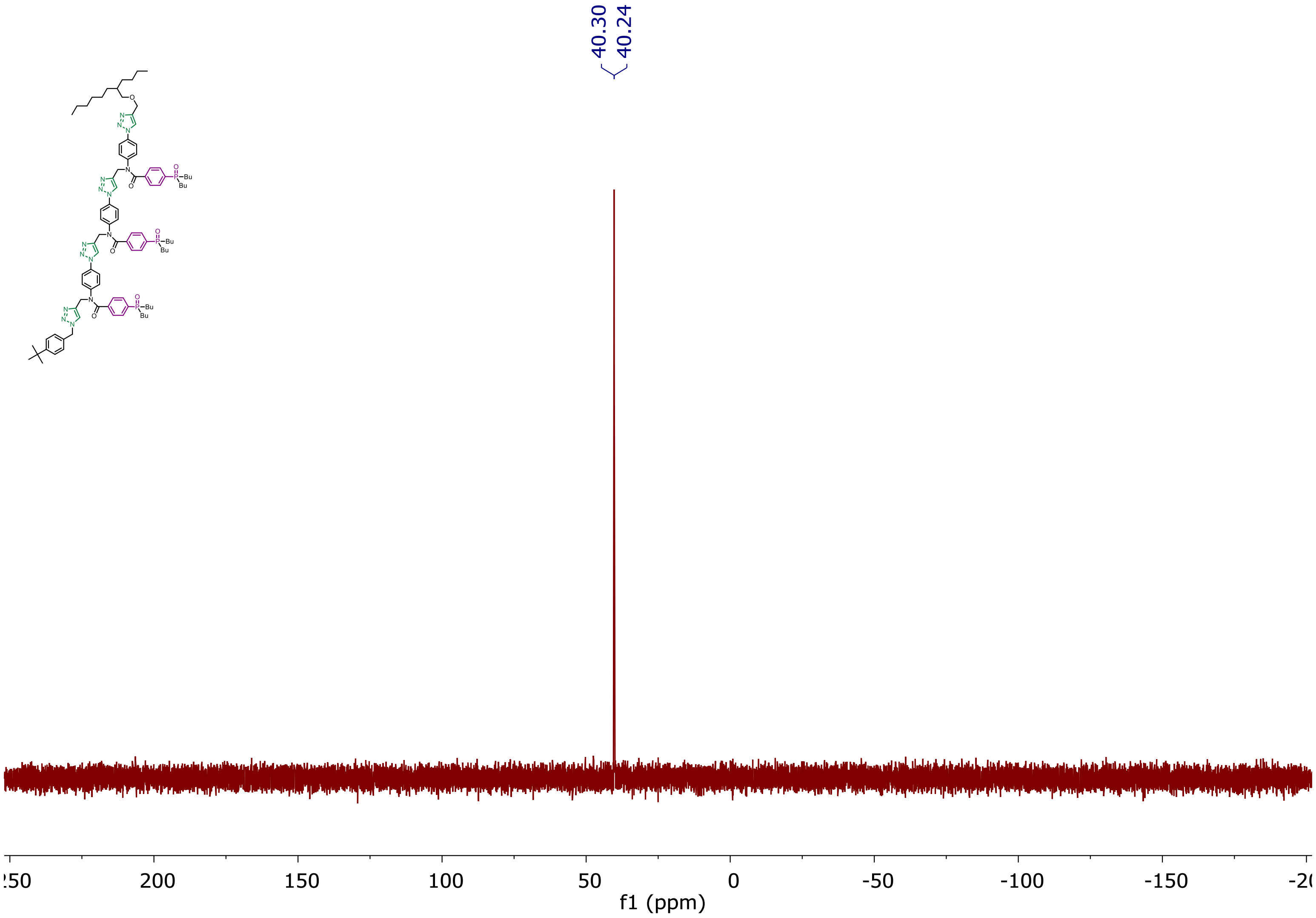

Characterization of 4-mer 13

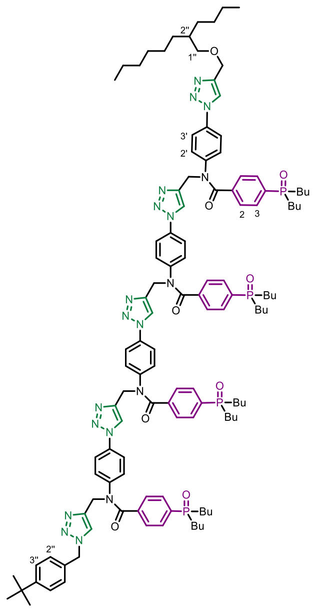

Purification method: Flash column chromatography using silica gel (gradient from 0% to 10% of CH_2Cl_2 in MeOH) to afford **DN842-F1** (0.004 g, 44%) as a pale yellow oil.

^{1}H NMR (500 MHz, $CDCl_3$): δ_H = 8.19 (s, 1H, CH_{triaz}, internal), 8.19 (s, 1H, CH_{triaz}, internal), 8.18 (s, 1H, CH_{triaz}, internal), 7.88 (s, 1H, CH_{triaz}, aliph. end), 7.66 (s, 1H, CH_{triaz}, ^{t}Bu end), 7.65 (d, 4H, *J* = 9.0 Hz, 3'-H), 7.62 (d, 2H, *J* = 9.0 Hz, 3'-H), 7.61 (d, 2H, *J* = 9.0 Hz, 3'-H), 7.52 (m, 8H, 3-H), 7.42 (m, 8H, 2-H), 7.39 (d, 2H, *J* = 8.5 Hz, 3''-H, ^{t}Bu cap), 7.33 (m, 8H, 2'-H), 7.20 (d, 2H, *J* = 8.5 Hz, 2''-H, ^{t}Bu cap), 5.48 (s, 2H, N-CH_2, ^{t}Bu cap), 5.22 (s, 6H, N-CH_2, internal), 5.13 (s, 2H, N-CH_2, internal), 4.67 (s, 2H, O-CH_2), 3.42 (d, 2H, *J* = 6.0 Hz, 1''-H, aliph. cap), 1.90 (m, 8H, 1''-H, Bu), 1.74 (m, 8H, 1''-H, Bu), 1.58 (m, 1H, 2''-H, aliph. cap), 1.49 (m, 8H, 2''-H, Bu), 1.31 (m, 33H, 2''-H and 3''-H, Bu; ^{t}Bu), 1.25 (m, 16H, CH_2, aliph. cap), 0.86 (m, 6H, CH_3, aliph. cap), 0.80 (t, 24H, *J* = 7.0 Hz, 4''-H, Bu).

^{13}C NMR (125.8 MHz, $CDCl_3$): δ_C = 169.5, 169.5 and 169.3 (CO), 152.2 (4''-C, ^{t}Bu cap), 147.0 (C_{triaz}, aliph. cap), 144.5, 144.4 and 144.4 (C_{triaz}, internal), 143.8 (1'-C), 143.8 (C_{triaz}, ^{t}Bu cap), 143.7 (1'-C), 138.2, 138.0 and 138.0 (d, *J* = 2.5 Hz, 1-C), 135.7, 135.5 and 135.5 (4'-C), 135.5 (d, *J* = 89.0 Hz, 4-C), 135.4 (4'-C), 135.3 (d, *J* = 89.0 Hz, 4-C), 131.5 (1''-C, ^{t}Bu cap), 130.4 (d, *J* = 9.0 Hz, 3-C), 129.1 and 129.0 (d, *J* = 11.0 Hz, 2-C), 129.0, 128.9 and 128.9 (2'-C), 128.1 (2''-H, ^{t}Bu cap), 126.2 (3''-C, ^{t}Bu cap), 123.9 (CH_{triaz}, ^{t}Bu cap), 122.0 (CH_{triaz}, internal), 121.4, 121.2 and 121.1 (3'-C), 120.4 (CH_{triaz}, aliph. end), 74.4 (1''-C, aliph. cap), 64.7 (O-CH_2), 54.1 (N-CH_2, ^{t}Bu cap), 46.5 and 46.4 (N-CH_2, internal), 38.4 (2''-C, aliph. cap), 34.8 (C, ^{t}Bu), 32.0 and 31.5 (CH_2, aliph. cap), 31.4 (CH_3, ^{t}Bu), 31.2 and 29.9 (CH_2, aliph. cap), 29.5 (d, *J* = 68.5 Hz, 1''-C, Bu),29.2 and 26.9 (CH_2, aliph. cap), 24.1 (d, *J* = 14.5 Hz, 2''-C, Bu), 23.6 (d, *J* = 4.0 Hz, 3''-C, Bu), 23.2 and 22.8, (CH_2, aliph. cap), 14.3 and 14.3 (CH_3, aliph. cap), 13.7 (4''-H, Bu).

^{31}P NMR (161.9 MHz, $CDCl_3$): δ_P = 40.4 and 40.3.

HRMS (ES+): calcd for $C_{122}H_{160}N_{19}O_9P_4$ 1080.0832 $[M+2H]^{2+}$, found 1080.0845 $[M+2H]^{2+}$.

FT-IR (ATR): ν_{max} 2958, 2924, 2869, 2853, 1711, 1649, 1519, 1417, 1379, 1222 and 1170 cm^{-1}.

^{1}H-NMR (500 MHz, $CDCl_3$) compound 13

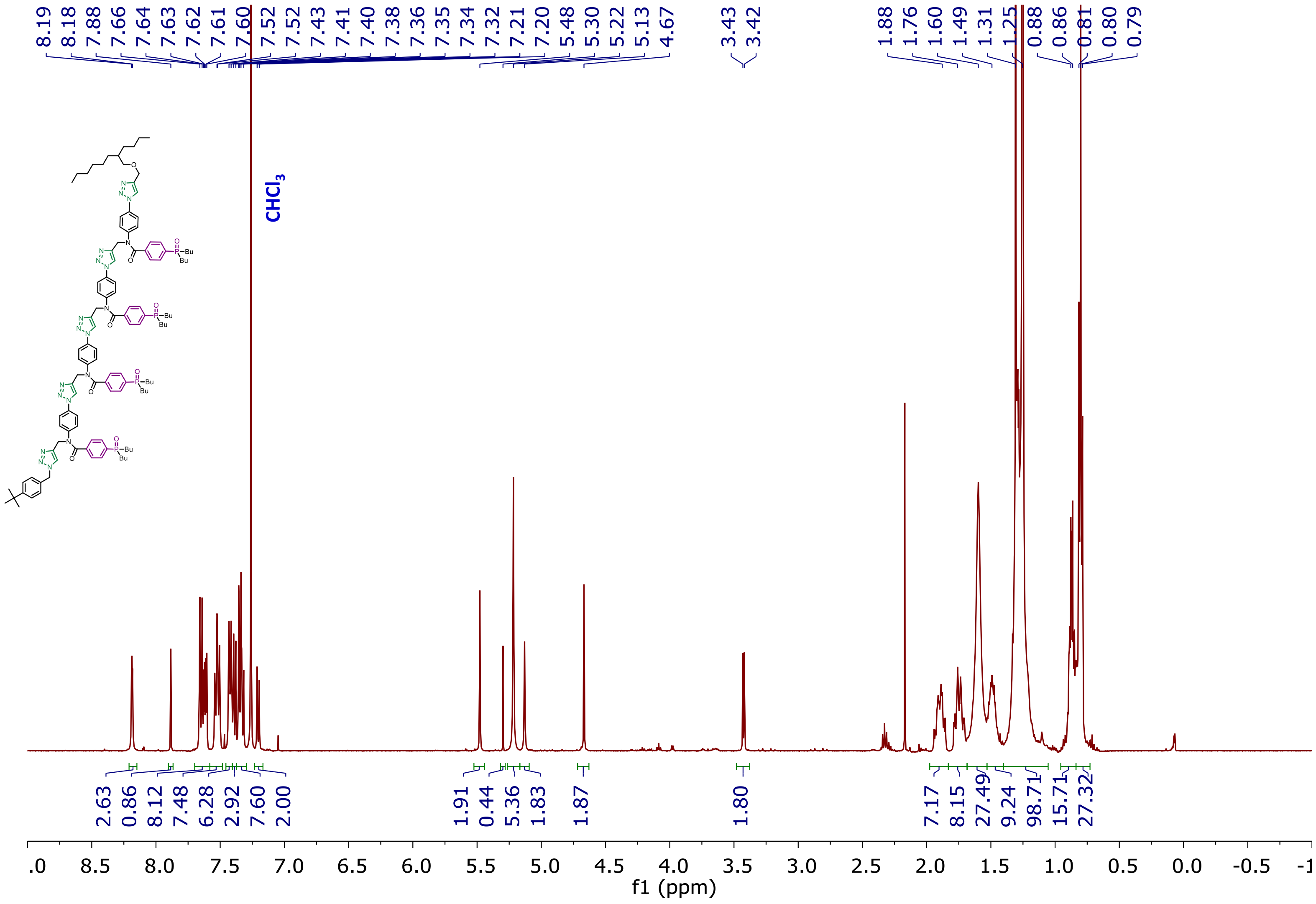

^{13}C-NMR (125.7 MHz, $CDCl_3$) compound 13

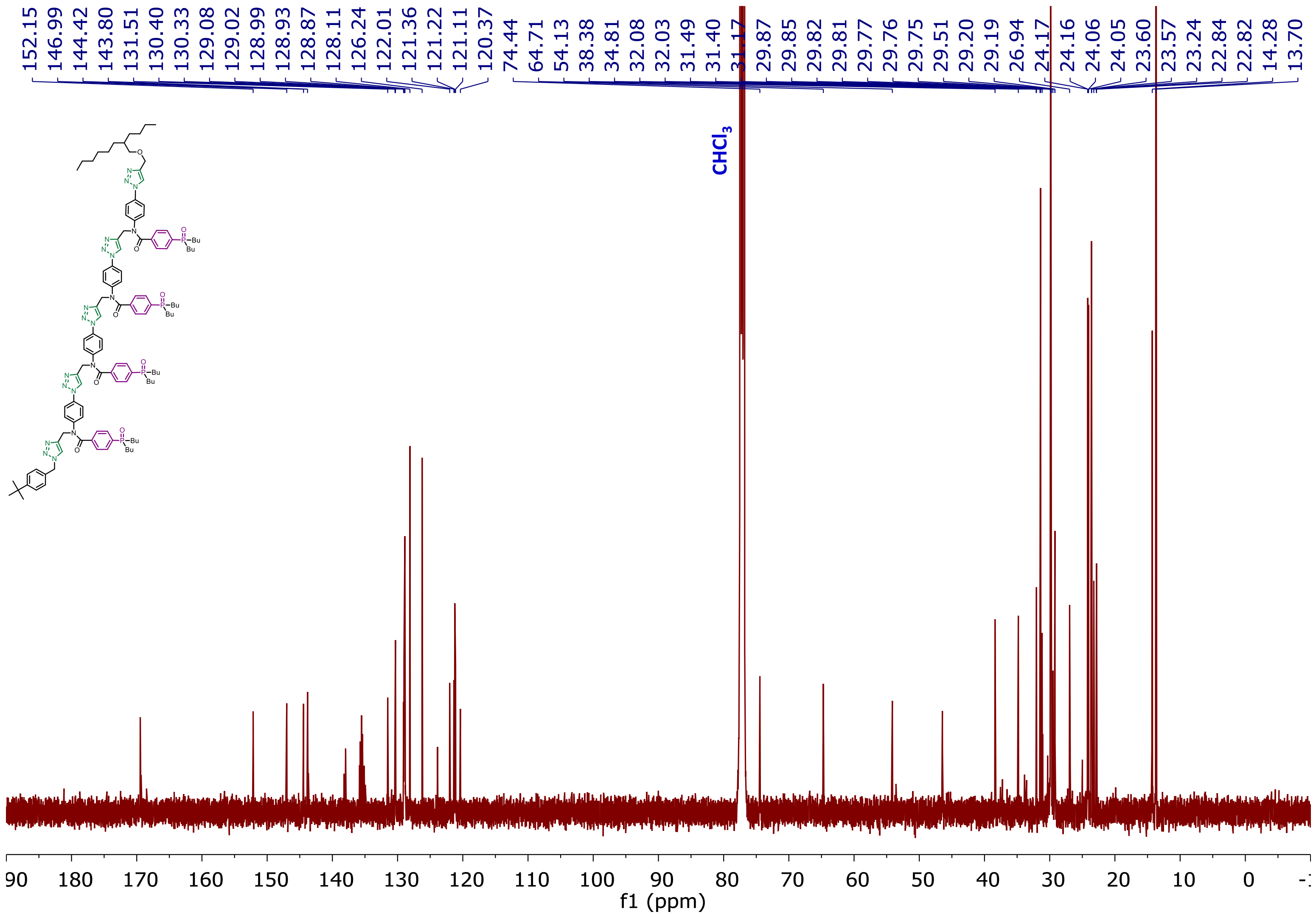

^{31}P NMR (161.9 MHz, $CDCl_3$) compound 13

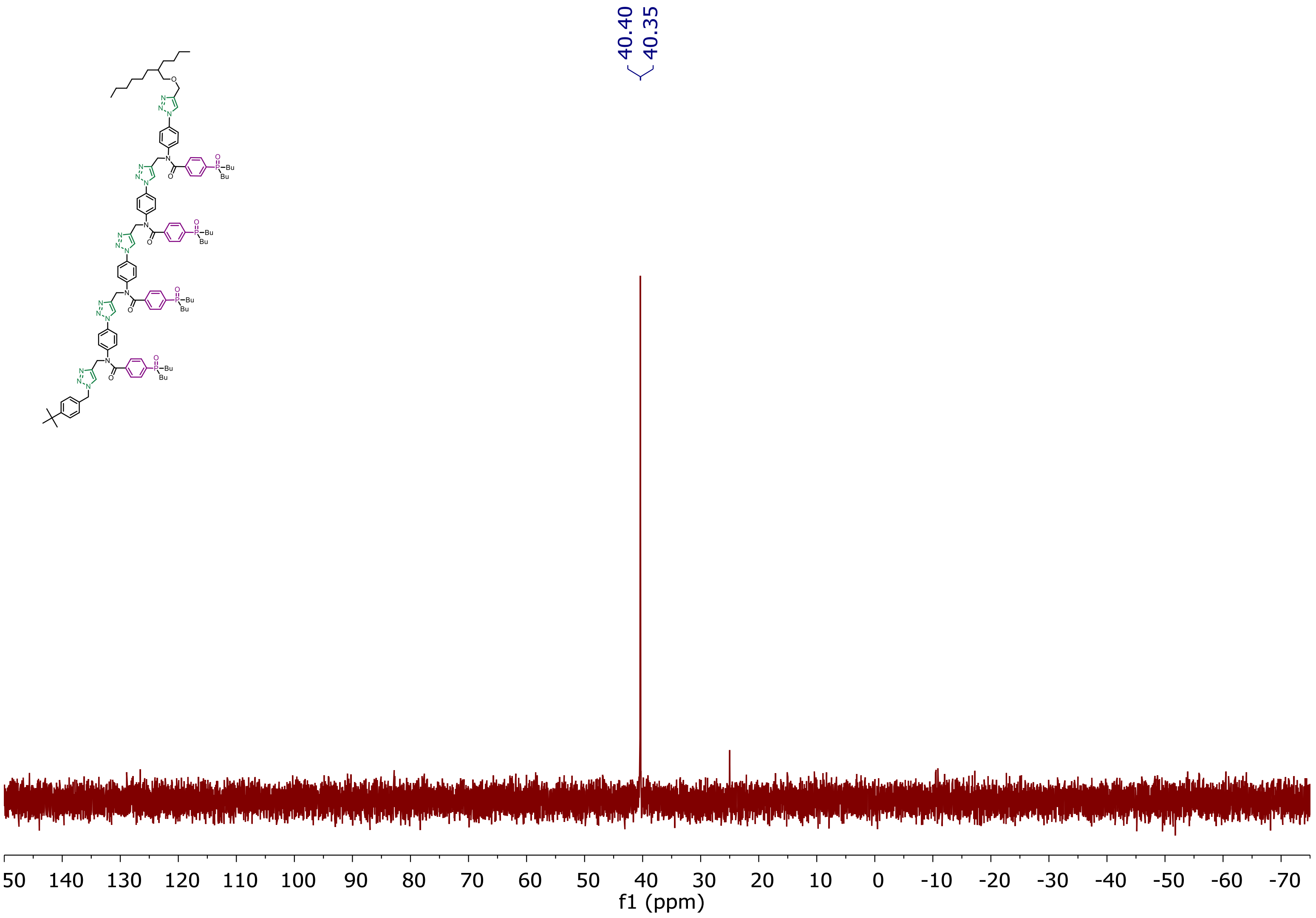

3.2. Phenol homo-oligomers (D_N)

As shown in Scheme S4, phenol homo-oligomers were prepared through non-templated CuAAC oligomerization of phenol 1-mer **14**[S1] using 1-(Azidomethyl)-4-*tert*-butylbenzene[S1] as end-capping group, followed by *in situ* capping of the azido terminal group with 2-butyloctyl propargyl ether (**S5**). This method provided a distribution of fully capped oligomers from 1-mer **15** to 4-mer **18**. Figure S3 shows the UPLC traces of the starting material **14**, the oligomerization reaction crude mixture before and after capping as well as the isolated oligomers **15**-**18**. Figure S4 shows the methylene and aromatic regions of the ^{1}H NMR spectra for phenol oligomers **15**-**18**. In the spectra, the extra methylene group corresponding to the increasing number of repeating units can be easily visualized.

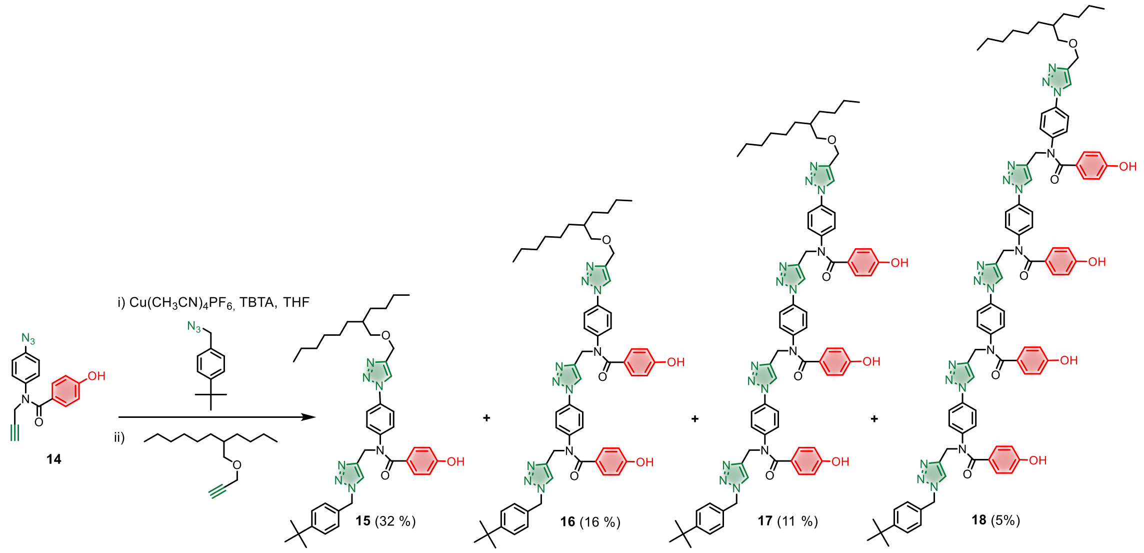

Scheme S3. Synthesis of phenol oligomers **15**-**18**.

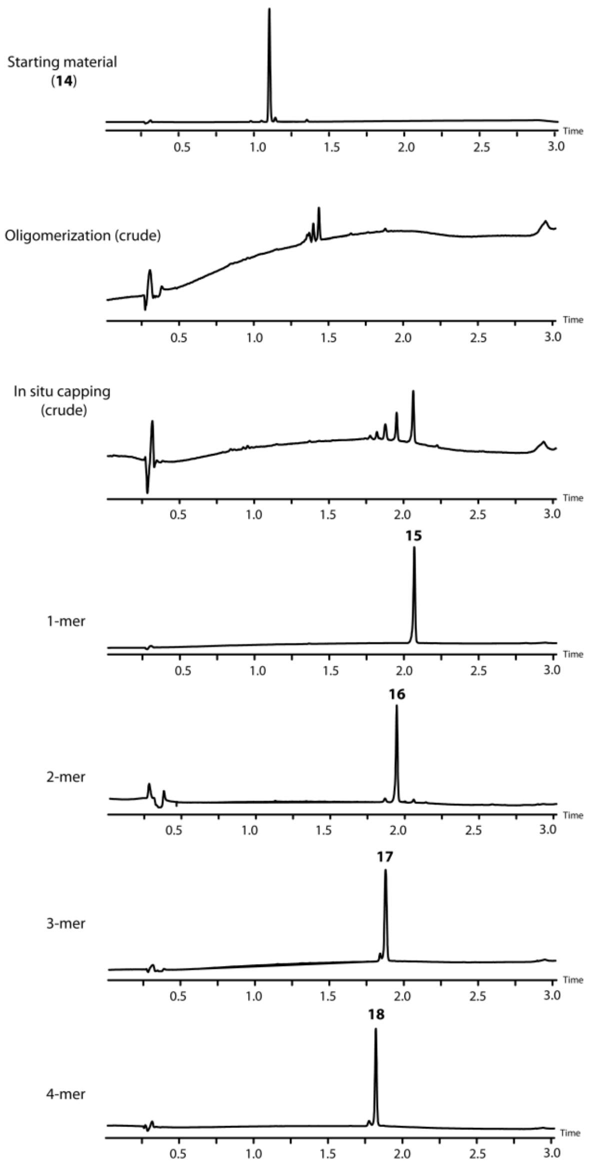

Figure S3. UPLC traces for the oligomerization and capping of phenol **14**. *UPLC Conditions*: C18 column at 40 °C (254 nm) using water + 0.1% formic acid (A) and CH_3CN + 0.1% formic acid (B) with flow rate of 0.6 ml/min; Gradient of 0-2 minutes 5%-100% B + 1 minute 100% B.

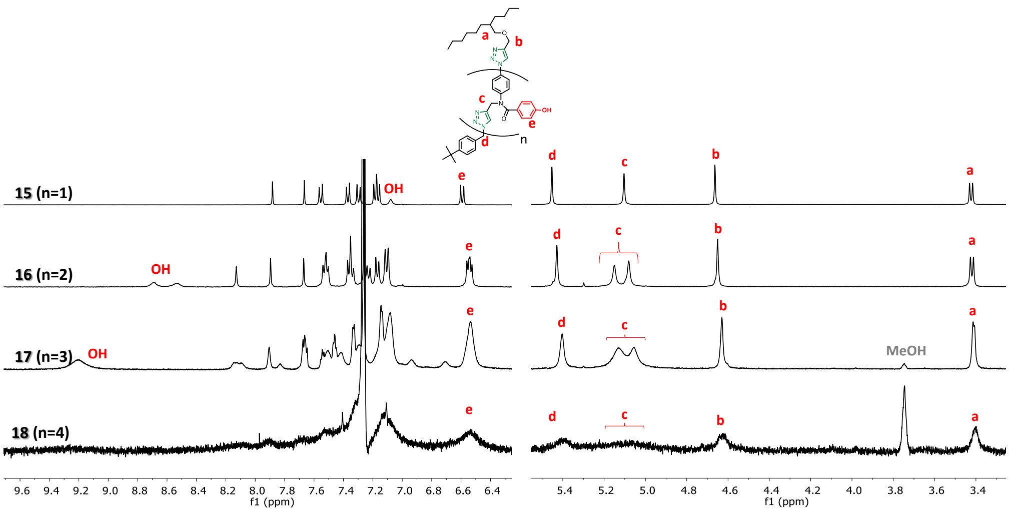

Figure S4. Methylene and aromatic regions of the 400 MHz ^{1}H NMR for oligomers **15**-**18** ($CDCl_3$, 298 K).

Synthesis of phenol oligomers by oligomerization followed by *in situ* capping

Compound **14** (0.135 g, 0.46 mmol), and 1-(azidomethyl)-4-*tert*-butylbenzene (0.131 g, 0.69 mmol), were dissolved in dry THF (120 mL) under N_2 atmosphere. $Cu(CH_3CN)_4PF_6$ (0.017 g, 0.05 mmol) and TBTA (0.025 g, 0.05 mmol) were added to the reaction and the solution was stirred at room temperature for 2 days. Then, compound **S5** (0.207 g, 0.92 mmol), $Cu(CH_3CN)_4PF_6$ (0.017 g, 0.05 mmol) and TBTA (0.025 g, 0.05 mmol) were added. After overnight stirring at room temperature, the solution was then diluted with EtOAc and washed with EDTA soln. (2x), H_2O (1x) and brine. The organic layer was dried over $MgSO_4$ and concentrate under vacuum. The residue was purified by flash column chromatography on silica gel (gradient from 0% to 100% of EtOAc in pet. ether and then gradient from 0 to 10% of MeOH in CH_2Cl_2) to afford 1-mer **15** (0.103 g, 32%) as a white amorphous solid, 2-mer **16**(0.039 g, 16%) as a white amorphous solid, 3-mer **17** (0.021 g, 11%) as a white amorphous solid and 4-mer **18** 0.007 g, 4%) as a white amorphous solid.

Characterization of 1-mer 15

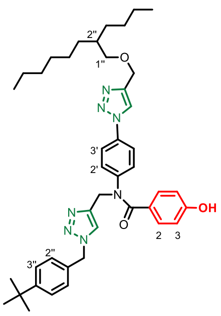

^{1}H NMR (400 MHz, CDCl$_3$): δ_H = 7.88 (s, 1H, CH_{triaz}, aliphatic end), 7.67 (s, 1H, CH_{triaz}, ^{t}Bu end), 7.55 (d, 2H, J = 9.0 Hz, 3'-H), 7.37 (d, 2H, J = 8.0 Hz, 3''-H, ^{t}Bu cap), 7.30 (d, 2H, J = 9.0 Hz, 2'-H), 7.18 (d, 2H, J = 8.0 Hz, 2''-H, ^{t}Bu cap), 7.16 (d, 2H, J = 8.5 Hz, 2-H), 7.08 (s, 1H, OH), 6.59 (d, 2H J = 8.5 Hz, 3-H), 5.45 (s, 2H, N-CH_2, ^{t}Bu cap), 5.10 (s, 2H, N-CH_2, internal), 4.66 (s, 2H, O-CH_2), 3.42 (d, 2H, J = 6.0 Hz, 1''-H, aliphatic cap), 1.58 (m, 1H, 2''-H, aliphatic cap, overlapped by water peak), 1.31 (s, 9H, ^{t}Bu), 1.25 (m, 16H, CH_2, aliphatic cap), 0.86 (m, 6H, CH_3, aliphatic cap).

^{13}C NMR (125.8 MHz, CDCl$_3$): δ_C = 170.1 (CO), 158.5 (4-C), 152.1 (4''-C, ^{t}Bu cap), 146.8 (C_{triaz}, aliphatic cap), 145.0 (1'-C), 144.3 (C_{triaz}, ^{t}Bu cap), 134.9 (4'-C), 131.5 (2-C), 131.4 (1''-C, ^{t}Bu cap), 128.5 (2'-C), 128.1 (2''-H, ^{t}Bu cap), 126.5 (1-C), 126.2 (3''-C, ^{t}Bu cap), 124.0 (CH_{triaz}, ^{t}Bu cap), 121.4 (3'-C), 120.7 (CH_{triaz}, aliphatic end), 115.1 (3-C), 74.5 (1''-C, aliphatic cap), 64.6 (O-CH_2), 54.1 (N-CH_2, ^{t}Bu cap), 46.4 (N-CH_2, internal), 38.4 (2''-C, aliphatic cap), 34.8 (C, ^{t}Bu), 32.0 and 31.5 (CH_2, aliphatic cap), 31.4 (CH_3, ^{t}Bu), 31.2, 29.9, 29.2, 26.9, 23.2 and 22.8 (CH_2, aliphatic cap), 14.3 and 14.3 (CH_3, aliphatic cap).

HRMS (ES-): calcd for $C_{42}H_{56}N_7O_3$ 704.4294 $[M-H]^-$, found 704.4301 $[M-H]^-$.

FT-IR (ATR): ν_{max} 3140, 2956, 2925, 2857, 1711, 1640, 1608, 1517, 1362, 1222 and 845 cm^{-1}.

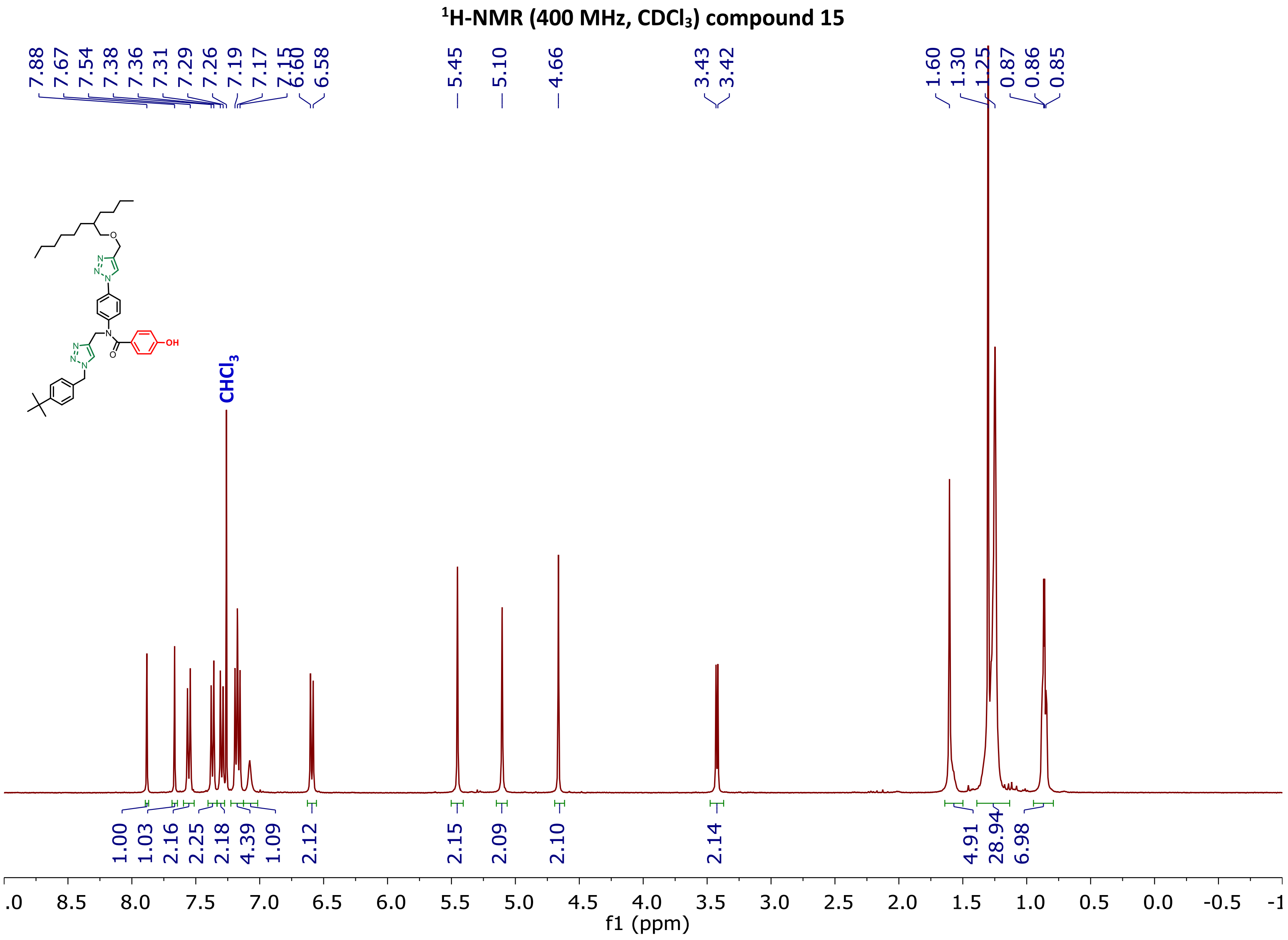

¹H-NMR (400 MHz, CDCl₃) compound 15
CHCl₃
OH
f1 (ppm)

^{13}C-NMR (125.8 MHz, $CDCl_3$) compound 15

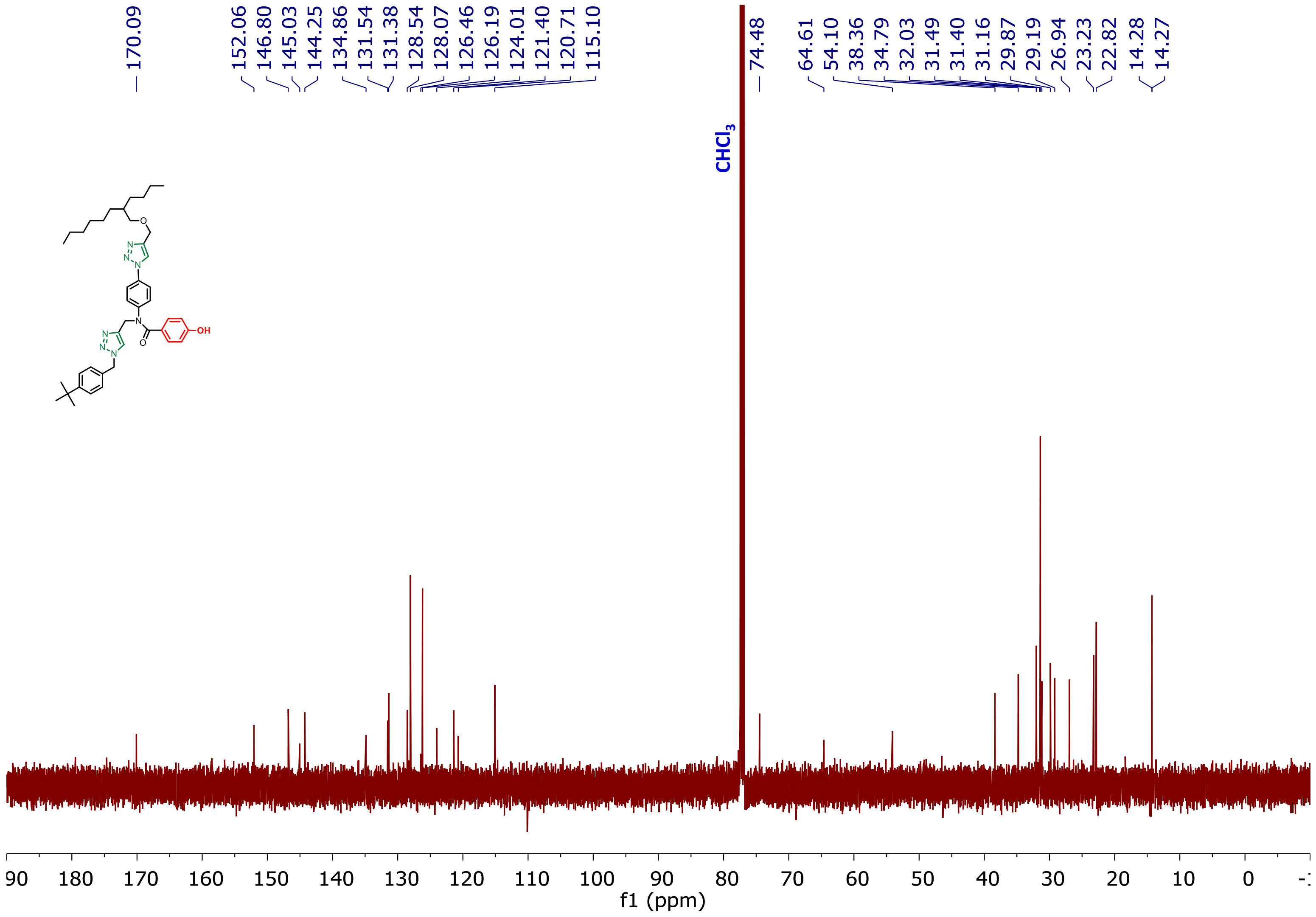

Characterization of 2-mer 16

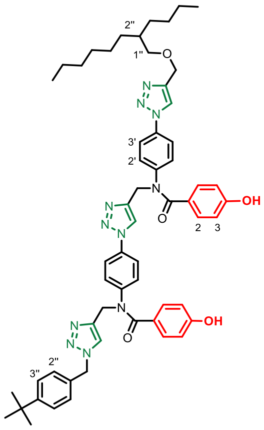

^{1}H NMR (500 MHz, $CDCl_3$): δ_H = 8.89 (s, 1H, OH), 8.77 (s, 1H, OH), 8.14 (s, 1H, CH_{triaz}, internal), 7.90 (s, 1H, CH_{triaz}, aliph. end), 7.67 (s, 1H, CH_{triaz}, ^{t}Bu end), 7.52 (d, 2H, J = 9.0 Hz, 3'-H), 7.50 (d, 2H, J = 9.0 Hz, 3'-H), 7.36 (d, 2H, J = 8.0 Hz, 3''-H, ^{t}Bu cap), 7.33 (d, 2H, J = 9.0 Hz, 2'-H), 7.22 (d, 2H, J = 9.0 Hz, 2'-H), 7.17 (d, 2H, J = 8.0 Hz, 2''-H, ^{t}Bu cap), 7.10 (m, 4H, 2-H), 6.55 (d, 2H J = 8.5 Hz, 3-H), 6.54 (d, 2H J = 8.5 Hz, 3-H), 5.43 (s, 2H, N-CH_2, ^{t}Bu cap), 5.15 (s, 2H, N-CH_2, internal), 5.08 (s, 2H, N-CH_2, internal), 4.65 (s, 2H, O-CH_2), 3.42 (d, 2H, J = 6.0 Hz, 1''-H, aliph. cap), 1.58 (m, 1H, 2''-H, aliph. cap), 1.29 (s, 9H, ^{t}Bu), 1.24 (m, 16H, CH_2, aliph. cap), 0.85 (m, 6H, CH_3, aliph. cap).

^{13}C NMR (125.8 MHz, $CDCl_3$): δ_C = 170.5 and 170.3 (CO), 159.3 and 159.1 (4-C), 152.1 (4''-C, ^{t}Bu cap), 146.7 (C_{triaz}, aliph. cap), 145.1 and 145.0 (1'-C), 144.6 (CH_{triaz}, internal), 144.2 (C_{triaz}, ^{t}Bu cap), 134.9 and 134.6 (4'-C), 131.6 and 131.5 (2-C), 131.4 (1''-C, ^{t}Bu cap), 128.5 and 128.4 (2'-C), 128.1 (2''-H, ^{t}Bu cap), 126.2 (3''-C, ^{t}Bu cap), 125.8 and 125.6 (1-C), 124.1 (CH_{triaz}, ^{t}Bu cap), 122.4 (CH_{triaz}, internal), 121.6 and 121.0 (3'-C), 120.9 (CH_{triaz}, aliph. end), 115.2 and 115.1 (3-C), 74.5 (1''-C, aliph. cap), 64.5 (O-CH_2), 54.1 (N-CH_2, ^{t}Bu cap), 46.7 and 46.4 (N-CH_2, internal), 38.3 (2''-C, aliph. cap), 34.8 (C, ^{t}Bu), 32.0 and 31.5 (CH_2, aliph. cap), 31.4 (CH_3, ^{t}Bu), 31.1, 29.9, 29.2, 26.9, 23.2 and 22.8 (CH_2, aliph. cap), 14.3 (CH_3, aliph. cap).

HRMS (ES+): calcd for $C_{58}H_{68}N_{11}O_5$ 998.5399 $[M+H]^+$, found 998.5413 $[M+H]^+$.

FT-IR (ATR): ν_{max} 3142, 2957, 2926, 2856, 1711, 1608, 1587, 1569, 1434, 1279 and 1172 cm^{-1}.

^{1}H-NMR (500 MHz, $CDCl_3$) compound 16

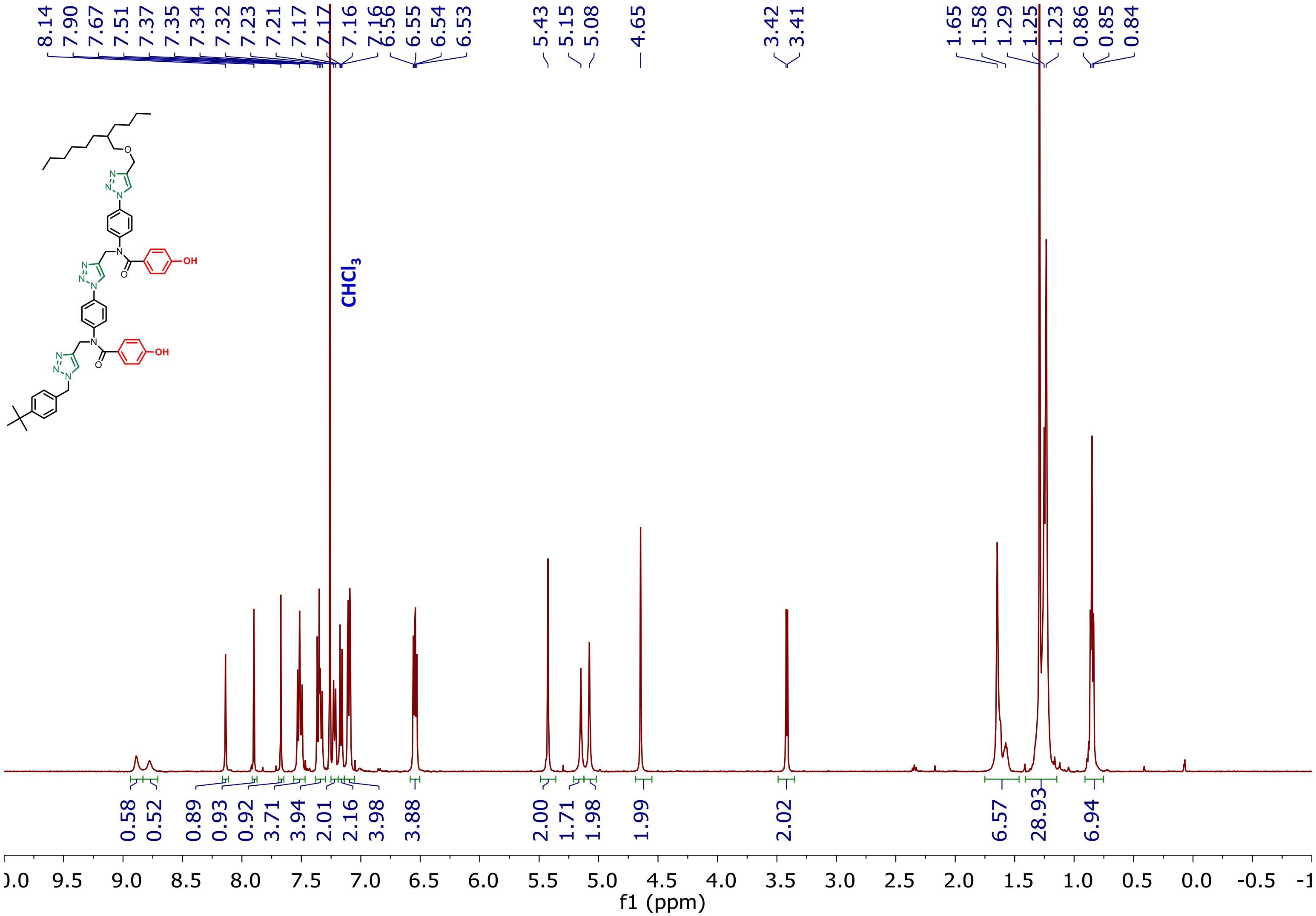

^{13}C-NMR (125.7 MHz, $CDCl_3$) compound 16

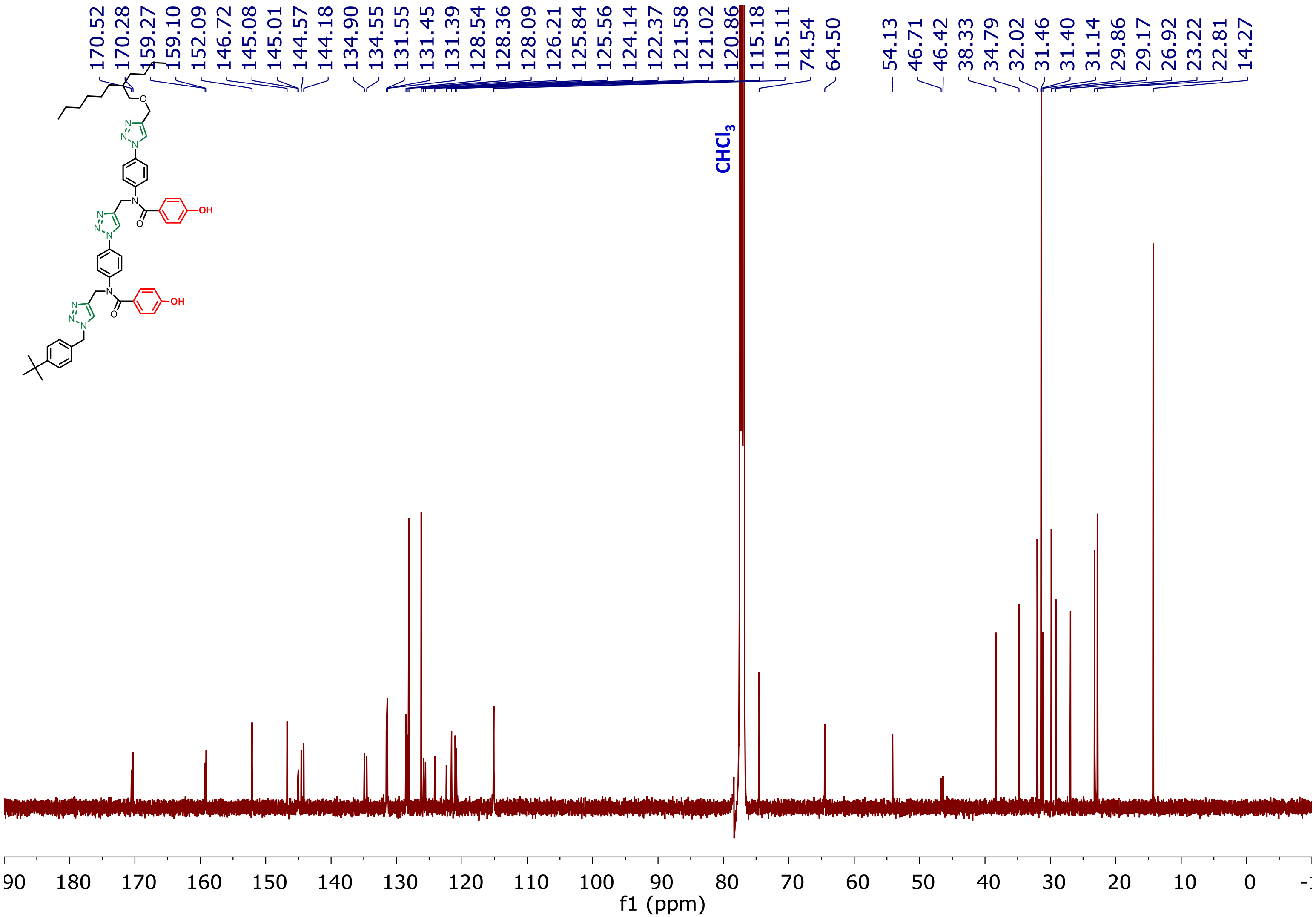

Characterization of 3-mer 17

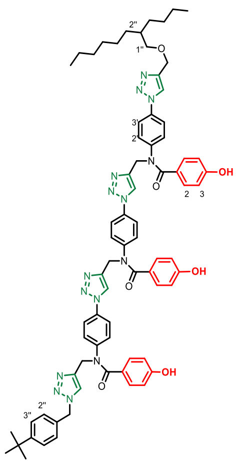

^{1}H NMR (500 MHz, CDCl$_3$): δ_H = 9.29 (s, 3H, OH), 8.12 (s, 2H, CH$_{triaz}$, internal), 7.91 (s, 1H, CH$_{triaz}$, aliph. end), 7.67 (s, 1H, CH$_{triaz}$, ^{t}Bu end), 7.49 (m, 2H, 3'-H), 7.39 (m, 2H, 3'-H), 7.32 (m, 4H, 3'-H; 3''-H, ^{t}Bu cap), 7.29 (m, 4H, 2'-H), 7.10 (m, 10H, 2-H; 2'-H; 2''-H, ^{t}Bu cap), 6.53 (m, 6H, 3-H), 5.39 (s, 2H, N-CH$_2$, ^{t}Bu cap), 5.12 (s, 4H, N-CH$_2$, internal), 5.05 (s, 2H, N-CH$_2$, internal), 4.62 (s, 2H, O-CH$_2$), 3.40 (d, 2H, *J* = 5.5 Hz, 1''-H, aliph. cap), 1.56 (m, 1H, 2''-H, aliph. cap), 1.25 (s, 9H, ^{t}Bu), 1.23 (m, 16H, CH$_2$, aliph. cap), 0.83 (t, 6H, *J* = 6.5 Hz, CH$_3$, aliph. cap).

^{13}C NMR (125.8 MHz, CDCl$_3$): δ_C = 170.8, 170.7 and 170.5 (CO), 159.7, 159.6 and 159.3 (4-C), 152.1 (4''-C, ^{t}Bu cap), 146.7 (C$_{triaz}$, aliph. cap), 145.8 (1'-C), 144.7 and 144.6 (CH$_{triaz}$, internal), 144.0 (C$_{triaz}$, ^{t}Bu cap), 135.0, 134.9 and 134.6 (4'-C), 132.3 (1''-C, ^{t}Bu cap)), 132.1, 131.5 and 131.4 (2-C), 128.7, 128.6 and 128.5 (2'-C), 128.1 (2''-H, ^{t}Bu cap), 126.2 (3''-C, ^{t}Bu cap), 125.7, 125.5 and 125.4 (1-C), 124.3 (CH$_{triaz}$, ^{t}Bu cap), 122.2 (CH$_{triaz}$, internal), 121.4 and 121.2 (3'-C), 120.9 (CH$_{triaz}$, aliph. end), 115.4, 115.3 and 115.2 (3-C), 74.5 (1''-C, aliph. cap), 64.5 (O-CH$_2$), 54.2 (N-CH$_2$, ^{t}Bu cap), 46.8, 46.2 and 46.2 (N-CH$_2$, internal), 38.3 (2''-C, aliph. cap), 34.8 (C, ^{t}Bu), 32.0 and 31.4 (CH$_2$, aliph. cap), 31.4 (CH$_3$, ^{t}Bu), 31.1, 29.9, 29.1, 26.9, 23.2 and 22.8 (CH$_2$, aliph. cap), 14.3 (CH$_3$, aliph. cap).

HRMS (ES+): calcd for $C_{74}H_{80}N_{15}O_7$ 1290.6360 $[M+H]^+$, found 1290.6346 $[M+H]^+$.

FT-IR (ATR): ν_{max} 3136, 2955, 2920, 2851, 1606, 1515, 1361, 1278, 1278, 1169 and 844 cm^{-1}.

^{1}H-NMR (500 MHz, $CDCl_3$) compound 17

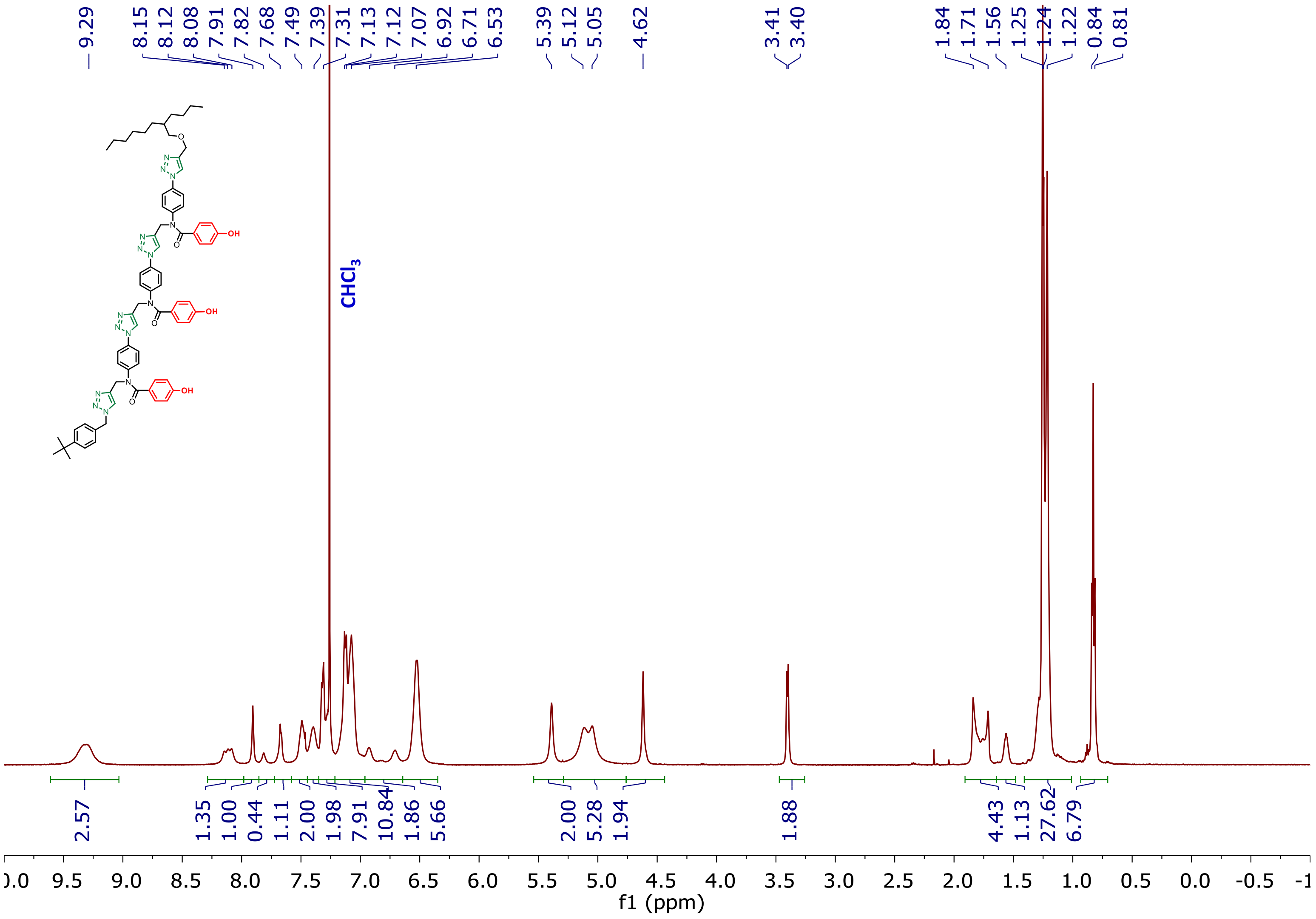

^{13}C-NMR (125.7 MHz, $CDCl_3$) compound 17

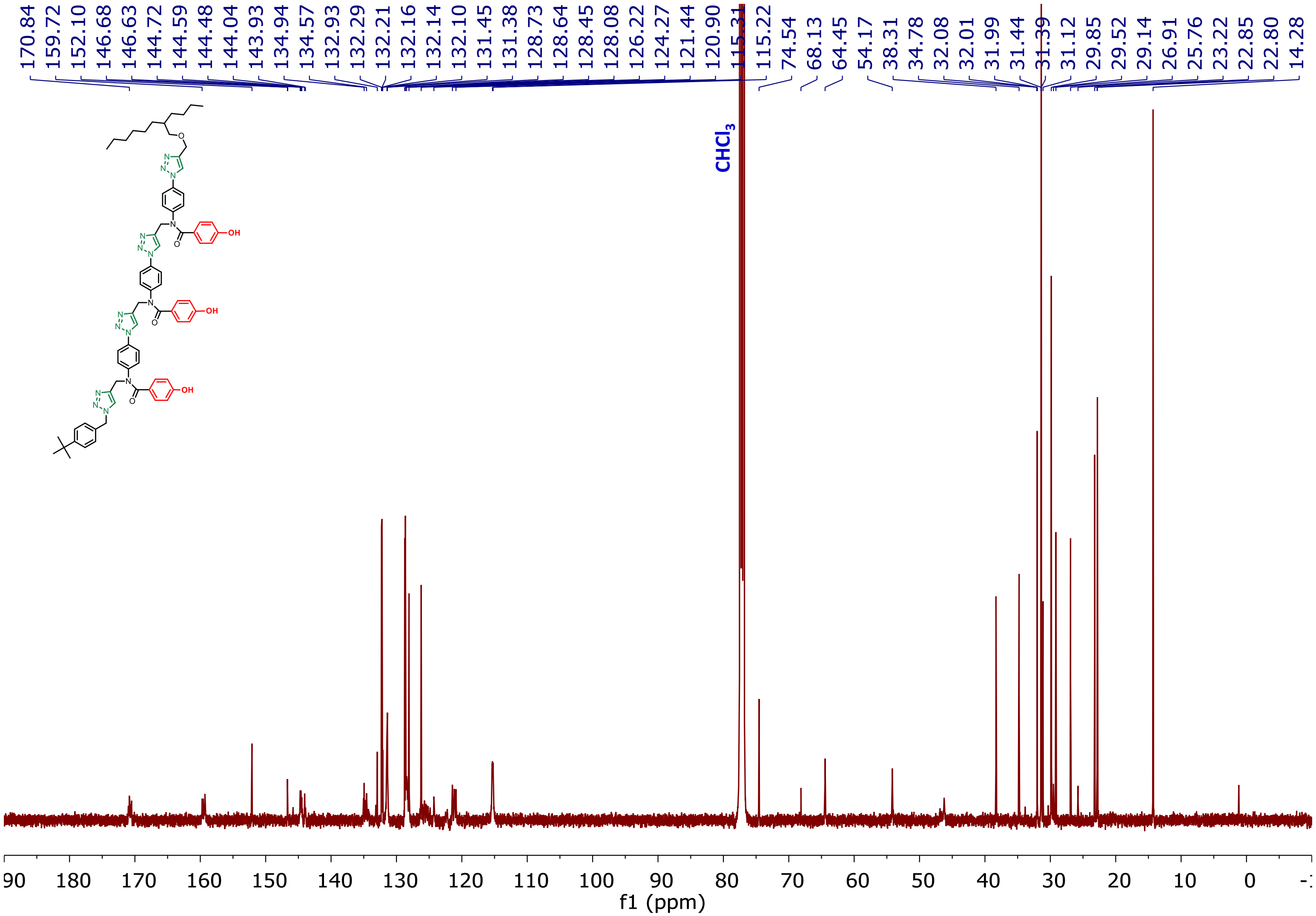

Characterization of 4-mer 18

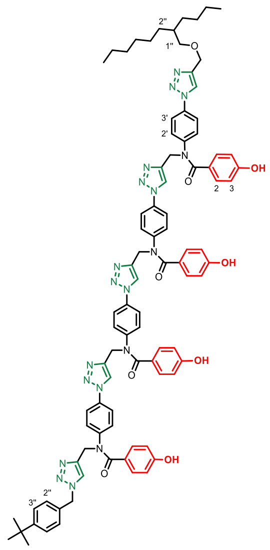

^{1}H NMR (500 MHz, DMSO-*d*$_6$): δ_H = 8.73 (s, 1H, CH$_{triaz}$), 8.70 (s, 1H, CH$_{triaz}$), 8.68 (s, 1H, CH$_{triaz}$), 8.05 (s, 1H, CH$_{triaz}$), 7.77 (d, 6H, *J* = 9.0 Hz, 3'-H), 7.64 (d, 2H, *J* = 9.0 Hz, 3'-H), 7.39 (m, 6H, 2'-H), 7.28 (m, 4H, 2'-H; 3''-H, ^{t}Bu cap), 7.19 (m, 6H, 2-H; 2''-H, ^{t}Bu cap), 7.10 (m, 4H, 2-H), 6.57 (d, 2H *J* = 9.0 Hz, 3-H), 5.47 (s, 2H, N-CH$_2$, ^{t}Bu cap), 5.16 (s, 4H, N-CH$_2$, internal), 5.10 (s, 2H, N-CH$_2$, internal), 4.52 (s, 2H, O-CH$_2$), 3.42 (1''-H, aliph. Cap, overlapped by water peak), 1.47 (m, 1H, 2''-H, aliph. cap), 1.23 (s, 9H, ^{t}Bu), 1.18 (m, 16H, CH$_2$, aliph. cap), 0.79 (m, 6H, CH$_3$, aliph. cap).

^{13}C NMR (125.8 MHz, DMSO-*d*$_6$): δ_C = 169.2, 169.2 and 169.1 (CO), 159.1 and 159.0 (4-C), 150.5 (4''-C, ^{t}Bu cap), 145.3 (1'-C), 144.7 144.6 and 143.7 (C$_{triaz,}$), 143.5 (1'-C), 134.2, 134.2, 133.2, and 133.2 (4'-C), 130.9 (1''-C, ^{t}Bu cap), 130.8 (2-C), 128.8, 128.8, 128.5, 128.0, 127.4, 127.4 and 127.3, 125.7, 125.7, 125.6, 125.4 and 125.4 (C$_{arom}$), 123.9 (CH$_{triaz}$), 122.0 and 121.5 (3'-C), 120.4 and 120.3 (CH$_{triaz}$), 114.6 and 114.5 (3-C), 72.3 (1''-C, aliph. cap), 63.2 (O-CH$_2$), 52.4 (N-CH$_2$), 45.4 (N-CH$_2$), 37.5 (2''-C, aliph. cap), 34.2 (C, ^{t}Bu), 31.3 and 31.2 (CH$_2$, aliph. cap), 31.0 (CH$_3$, ^{t}Bu), 30.8, 30.5, 29.0, 26.1, 23.1 and 22.5 (CH$_2$, aliph. cap), 13.9 and 13.9 (CH$_3$, aliph. cap).

HRMS (ES+): calcd for $C_{90}H_{92}N_{19}O_9$ 1582.7320 [M+H]$^+$, found 1582.7307 [M+H]$^+$.

FT-IR (ATR): ν_{max} 3128, 2955, 2918, 2850, 1707, 1632, 1607, 1517, 1280, 1234 and 845 cm^{-1}.

^{1}H-NMR (500 MHz, DMSO-d_6) compound 18

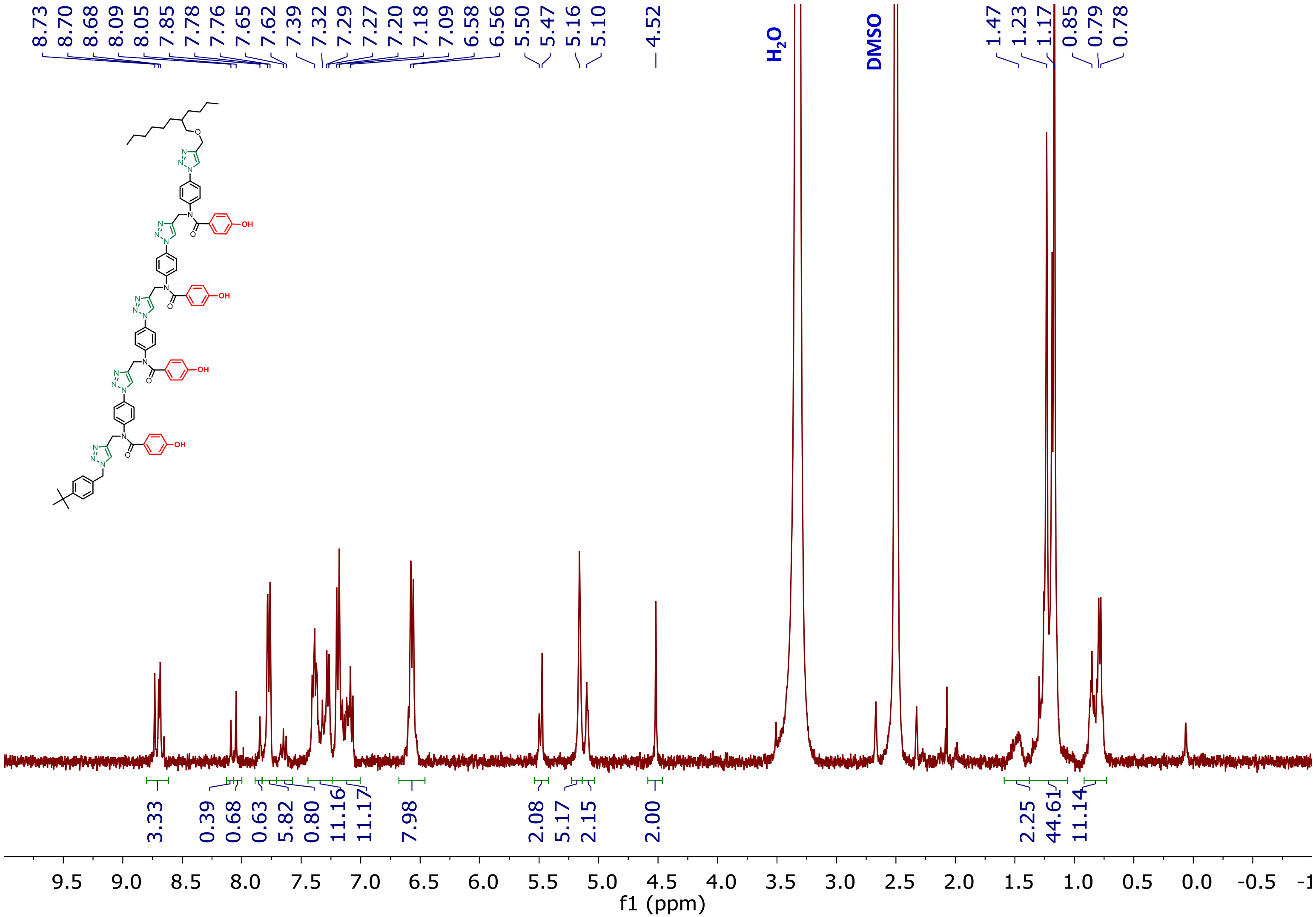

^{13}C-NMR (125.7 MHz, DMSO-d_6) compound 18

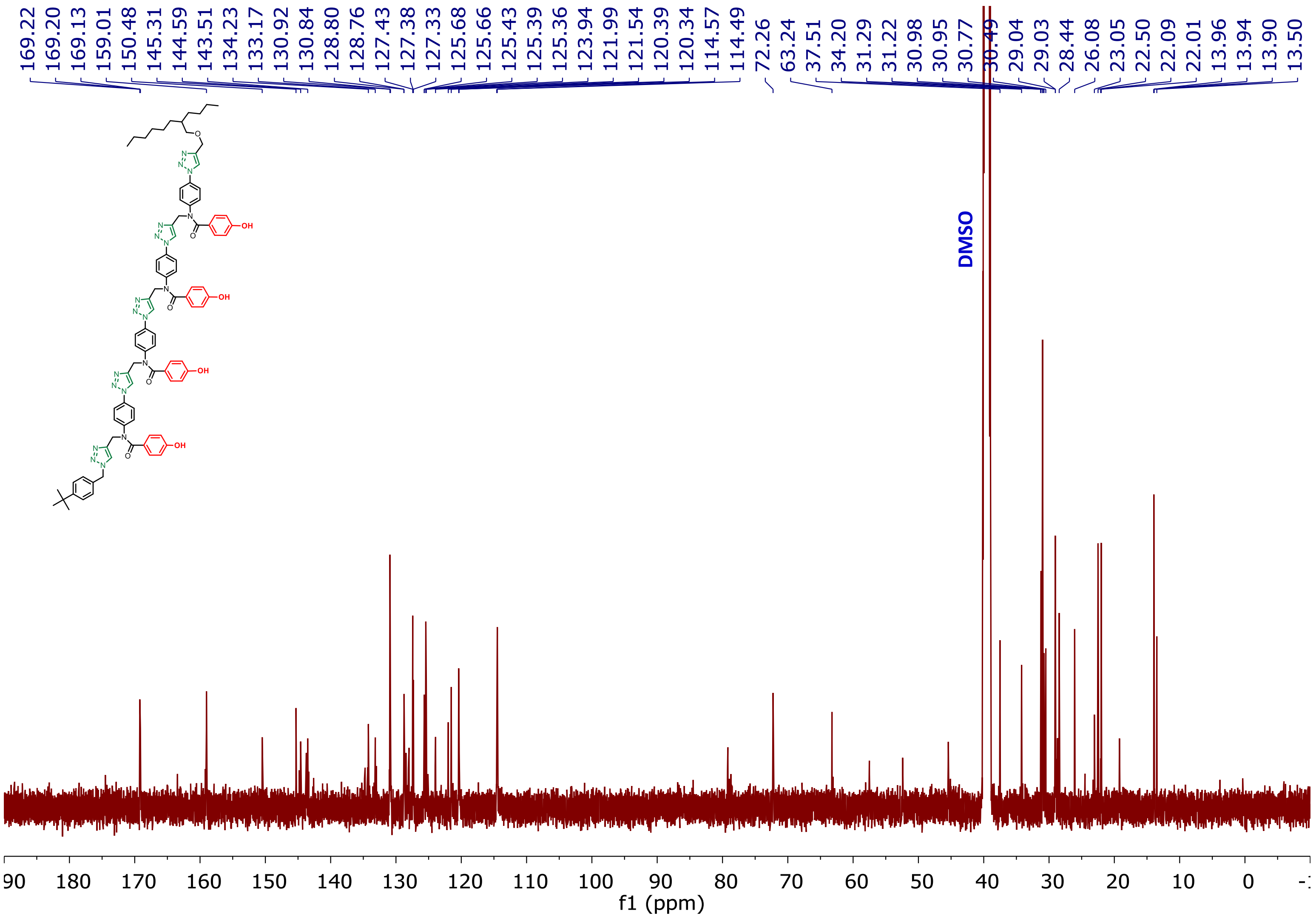

3.3. Phenol-phosphine oxide hetero-dimer (AD)

As shown in Scheme S4, the self-complementary 2-mer **20** was prepared in excellent yield by CuAAC coupling of azido-derived 1-mer 6 with capped phenol 1-mer **19**[S1]

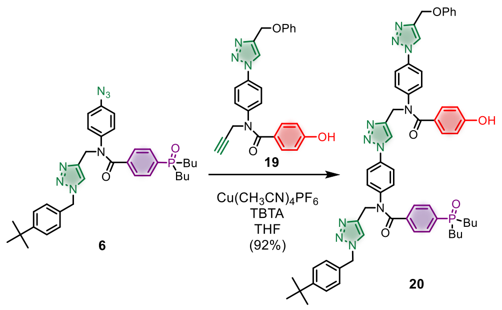

Scheme S4. Synthesis of self-complementary 2-mer **20**.

Synthesis of self-complementary 2-mer 20

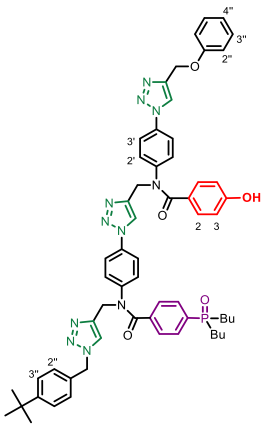

Compound **6** (0.005 g, 0.008 mmol) and compound **19** (0.003 g, 0.007 mmol) were dissolved in dry THF (120 mL) under N_2 atmosphere. $Cu(CH_3CN)_4PF_6$ (0.3 mg, $7{\cdot}10^{-4}$ mmol) and TBTA (0.4 mg, $7{\cdot}10^{-4}$ mmol) were added to the reaction and the solution was stirred overnight at room temperature. Then, the solution was diluted with EtOAc and washed with EDTA soln. (2x), H_2O (1x) and brine. The organic layer was dried over $MgSO_4$ and concentrate under vacuum. The residue was purified by flash column chromatography on silica gel (gradient from 0% to 100% of EtOAc in pet. ether and then gradient from 0 to 9% of MeOH in CH_2Cl_2) to afford **20** (0.007 g, 92%) as a white amorphous solid.

^{1}H NMR (500 MHz, $CDCl_3$): δ_H = 9.20 (s, 1H, OH), 8.17 (s, 1H, CH_{triaz}), 8.02 (s, 1H, CH_{triaz}), 7.65 (s, 1H, CH_{triaz}), 7.59 (d, 2H, *J* = 9.0 Hz, 3'-H), 7.58 (d, 2H, *J* = 9.0 Hz, 3'-H), 7.48 (m, 2H, 3-H, PO), 7.38 (m, 2H, 2-H, PO), 7.38 (d, 2H, *J* = 8.5 Hz, 3''-H, ^{t}Bu cap), 7.32 (d, 2H, *J* = 9.0 Hz, 2'-H), 7.28 (m, 4H, 2'-H; 3''-H, PhO cap), 7.19 (d, 2H, *J* = 8.5 Hz, 2''-H, ^{t}Bu cap), 7.14 (d, 2H, *J* = 8.5 Hz, 2-H, phenol), 7.00 (d, 2H, *J* = 8.0 Hz, 2''-H, PhO cap), 6.96 (t partially overlapped, *J* = 7.5 Hz, 4''-H, PhO cap), 6.64 (d, 2H *J* = 8.5 Hz, 3-H, phenol), 5.27 (s, 2H, O-CH_2), 5.18 (s, 2H, N-CH_2, internal), 5.12 (s, 2H, N-CH_2, internal), 1.87 (m, 2H, 1''-H, Bu), 1.73 (m, 2H, 1''-H, Bu), 1.43 (m, 2H, 2''-H, Bu), 1.30 (m, 1j5H, 2''-H and 3''-H, Bu; ^{t}Bu), 0.75 (t, 6H, *J* = 7.0 Hz, 4''-H, Bu).

^{13}C NMR (125.8 MHz, $CDCl_3$): δ_C = 170.5 and 169.3 (CO), 159.7 (4-C), 158.3 (1''-C, PhO cap), 152.1 (4''-C, ^{t}Bu cap), 145.3 (C_{triaz}), 145.1 (1'-C), 145.0 (C_{triaz}), 143.8 (C_{triaz}), 143.6 (1'-C), 138.5 (d, *J* = 2.5 Hz, 1-C, PO), 135.5 and 134.8 (4'-C), 134.4 (d, *J* = 90.5 Hz, 4-C, PO), 131.5 (1''-C, ^{t}Bu cap), 131.3 (2-C, phenol), 130.3 (d, *J* = 9.0 Hz, 3-C, PO), 129.8 (3''-C, PhO cap), 129.0 (d, *J* = 11.0 Hz, 2-C, PO), 128.8 and 128.4 (2'-C), 128.1 (2''-C, ^{t}Bu cap), 126.2 (3''-C, ^{t}Bu cap), 125.4 (1-C, phenol), 123.9 and 122.1 (CH_{triaz}), 121.5 (4''-C, PhO cap), 121.4 and 121.1 (3'-C), 121.0 (CH_{triaz}), 115.3 (3-C, phenol), 114.9 (2''-C, PhO cap), 62.0 (O-CH_2), 54.1 (N-CH_2, ^{t}Bu cap), 46.6 and 46.3 (N-CH_2, internal), 34.8 (C, ^{t}Bu), 31.4 (CH_3, ^{t}Bu), 29.2 (d, *J* = 68.5 Hz, 1''-C, Bu), 23.8 (d, *J* = 14.5 Hz, 2''-C, Bu), 23.5 (d, *J* = 4.0 Hz, 3''-C, Bu), 13.6 (4''-C, Bu).

^{31}P NMR (202.5 MHz, $CDCl_3$): δ_P = 43.2.

HRMS (ES+): calcd for $C_{60}H_{65}N_{11}O_5P$ 1050.4902 $[M+H]^+$, found 1050.4933 $[M+H]^+$.

FT-IR (ATR): ν_{max} 2960, 2926, 2855, 1644, 1606, 1518, 1282, 1237, 1168, 1045, 845, 755 cm^{-1}.

^{1}H-NMR (500 MHz, $CDCl_3$) compound 20

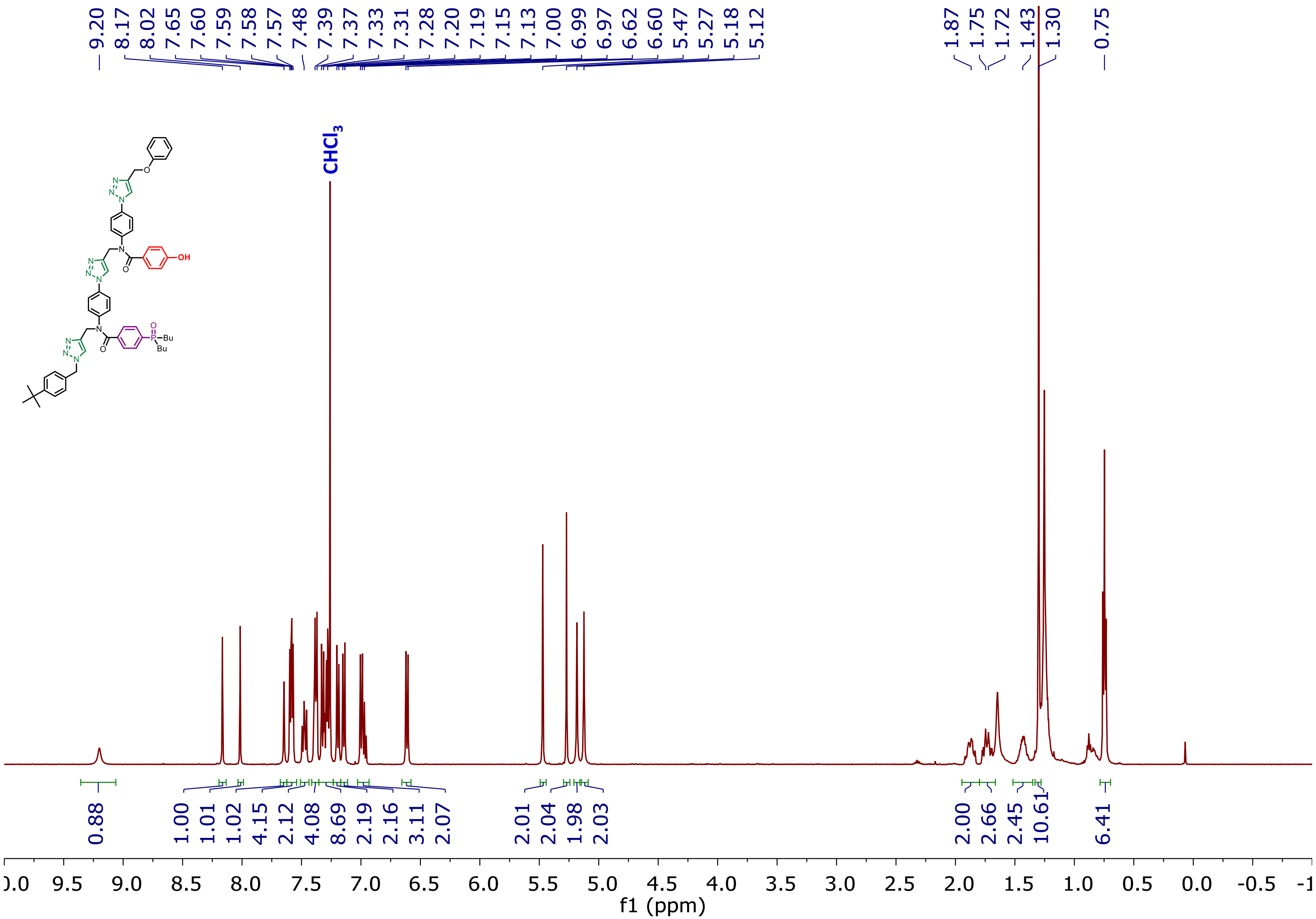

^{13}C-NMR (125.7 MHz, $CDCl_3$) compound 20

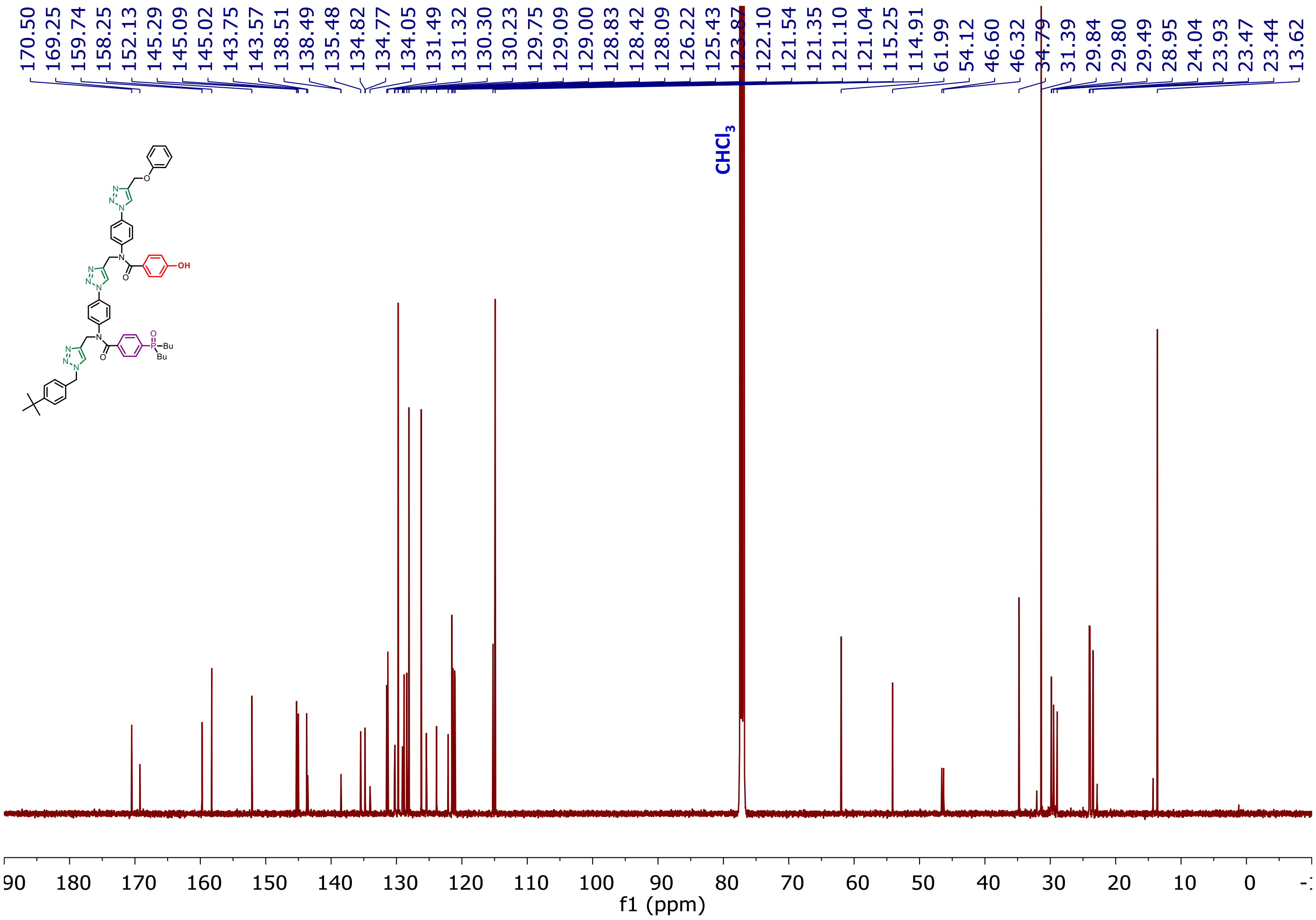

^{31}P NMR (202.5 MHz, CDCl3) compound 20

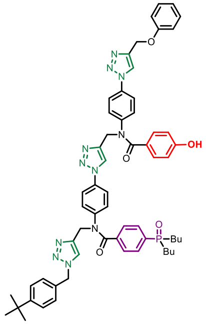

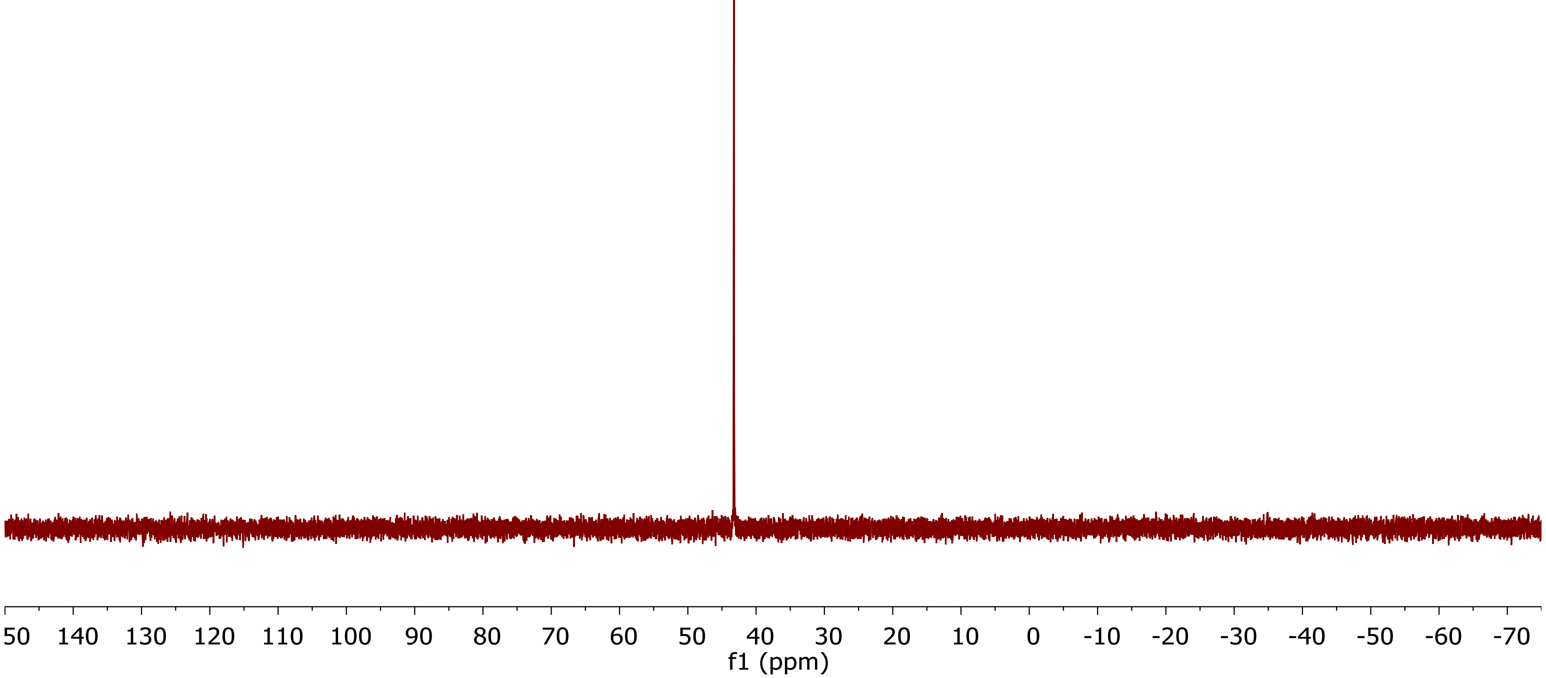

4. Binding studies

4.1. Molecular modelling

Molecular mechanics calculations were performed using MacroModel version 11.9.011, (Release 2019-1, Schrödinger Inc.).[S2] All structures were minimized first and the minimized structures were then used as the starting molecular structures for all MacroModel conformational searches. The force field used was MMFFs as implemented in this software ($CHCl_3$ solvation). The charges were defined by the force field library and no cut off were used for non-covalent interaction. A Polak-Ribiere Conjugate Gradient (PRCG) was used and each structure was subjected to 10000 iterations. The minima converged on a gradient with a threshold of 0.01. Conformational search was performed from previously minimized structures using 10000 steps. Images were created using PyMol.[S3]

Calculations were performed on simplified oligomer duplexes in which the capping groups were simplified to methyl and phenyl in order to reduce the computational cost. H-bond were fixed by constraining the distance between the phenol hydrogen and the phosphine oxide oxygen to 2 ± 1 Å and three different starting conformations were analysed in each case. The calculation outcomes for each duplex were sorted by energy and the 25 lowest-energy conformations were analysed.

Figure S5 shows the lowest energy conformation for 2-mer, 3-mer and 4-mer homo-oligomeric duplexes. Both parallel and antiparallel arrangements were calculated, being the lowest energy conformation the parallel for AA·DD and the antiparallel for AAA·DDD and AAAA·DDDD.

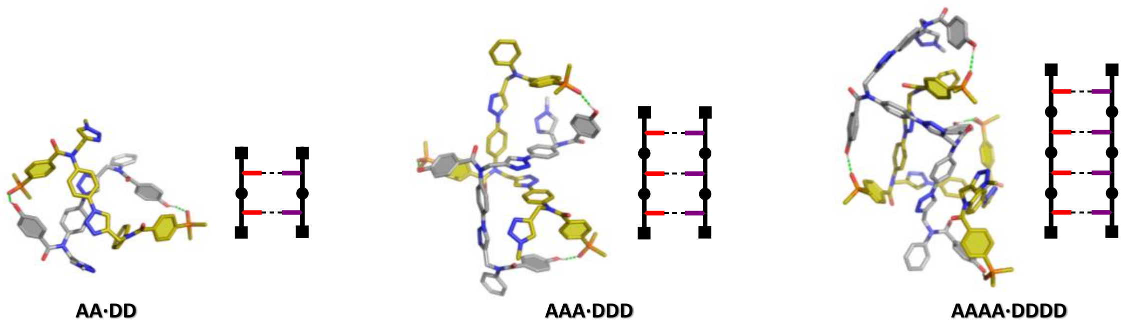

Figure S5. Molecular models from a MacroModel conformational search for AA·DD, AAA·DDD and AAAA·DDDD duplexes (MMFFs, $CHCl_3$ solvation). Both parallel and antiparallel arrangements were modelled and the lowest energy conformation for each duplex is shown (parallel for AA·DD; antiparallel for AAA·DDD and AAAA·DDDD)

4.2. NMR dilution of phenol 1-mer (15)

The ^1H NMR dilution of 1-mer **15** was performed in a Bruker 500 MHz AVIII HD Smart Probe spectrometer. Two stock solutions of **15** in CD_2Cl_2 were prepared (41.83 and 3.35 mM). A known volume of neat CD_2Cl_2 (600 μL) was added to an NMR tube. Increasing known volumes of **15** were added (first from the 3.35 mM stock solution and the from the 41.83 mM one) and the spectrum recorded after each addition. The chemical shifts of the **15** were monitored as a function of its concentration and analyzed using a purpose written software in Microsoft Excel (fitted to a dimerization isotherm). Errors were calculated as two times the standard deviation from the average value (95% confidence limit). Figure S6 shows the ^1H NMR spectra and the plot of the change in chemical shift as a function of concentration for the dilution of **15**. The dimerization constant obtained is 54 ± 16 M^{-1} in CD_2Cl_2 at 298 K.

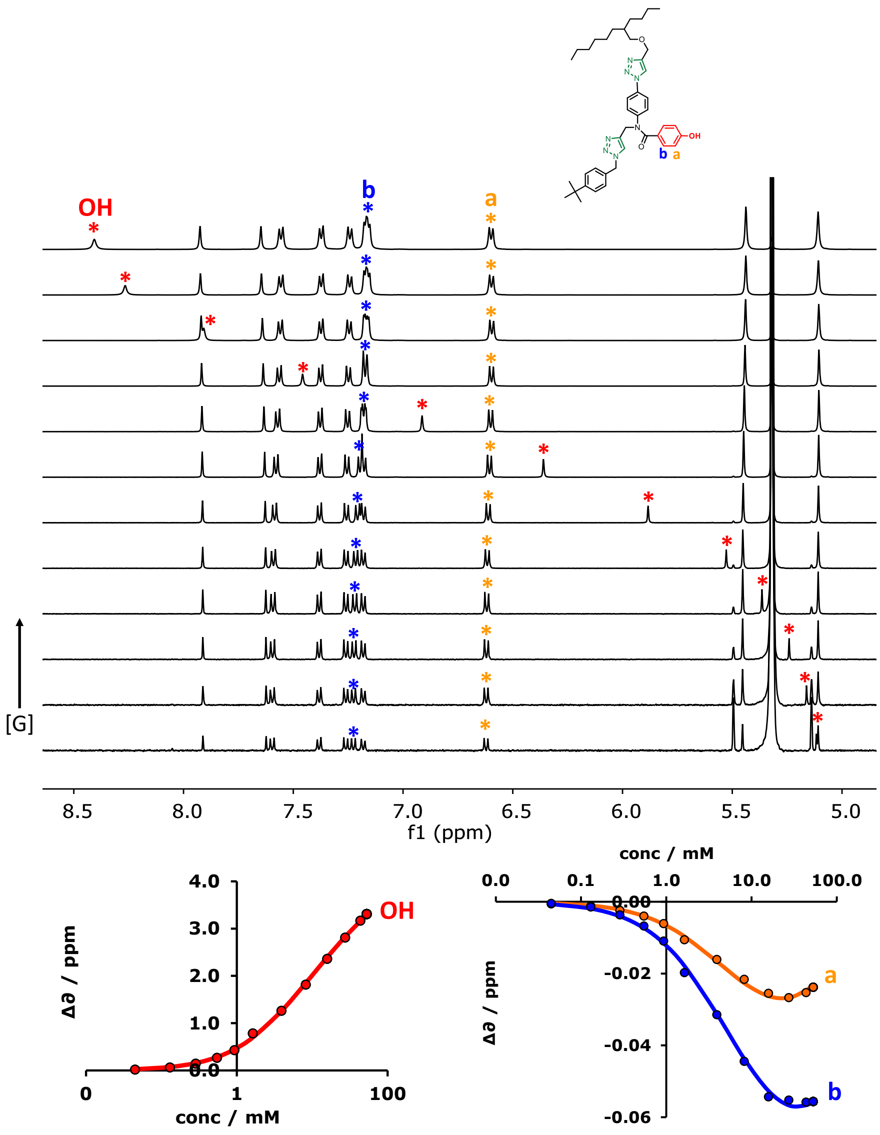

Figure S6. 500 MHz ^1H NMR spectra for dilution of 1-mer **15** at 298 K in CD_2Cl_2 and plot of the change in chemical shift as a function of concentration (the line represents the best fit to a dimerization isotherm). The red signal corresponds to the phenol OH proton, the orange signal to the protons *ortho* to the phenol oxygen and the blue signal to the protons *meta* to the phenol oxygen.

4.3. NMR titrations of homo-oligomers

All binding constants were measured by ^{31}P NMR titrations in a Bruker 500 MHz AVIII HD Smart Probe. The host (phosphine oxide derivatives **10**, **11** and **12**) was dissolved in CD2Cl2 at a known concentration. The guest (phenol derivatives **15**, **16** and **17**) was dissolved in the host solution and made to a known concentration. A known volume of host was added to an NMR tube and the spectrum was recorded. Known volumes of guest in host solution were added to the NMR tube, and the spectra were recorded after each addition. The chemical shifts of the host spectra were monitored as a function of guest concentration and analysed using a purpose written software in Microsoft Excel (fitted to a 1:1 binding isotherm). Errors were calculated as two times the standard deviation from the average value (95% confidence limit).

Figure S7, Figure S8 and Figure S9 show the ^{31}P NMR spectra and the plot of the change in ^{31}P chemical shift as a function of guest concentration for the titration of 1-mer **15** into 1-mer **10** (13.51 mM), 2-mer **16** into 2-mer **11** (0.50 mM) and 3-mer **17** into 3-mer **12** (0.22 mM). Phenol self-association was taken into account for the fitting of the titration data, including a fixed guest dimerization constant of 54 ± 16 M^{-1}, which was obtained from the dilution of phenol 1-mer **15** (section 4.2). Measured binding constant are quoted in Table 1 in the main text. For the 2-mer titration (Figure S8), the solubility of phenol **11** in CD_2Cl_2 is not enough to carry out a full titration because precipitation occurs guest is added at higher concentrations. For the 4-mers **13** and **18**, the expected binding constant is too high to be measure by ^{31}P NMR titration.

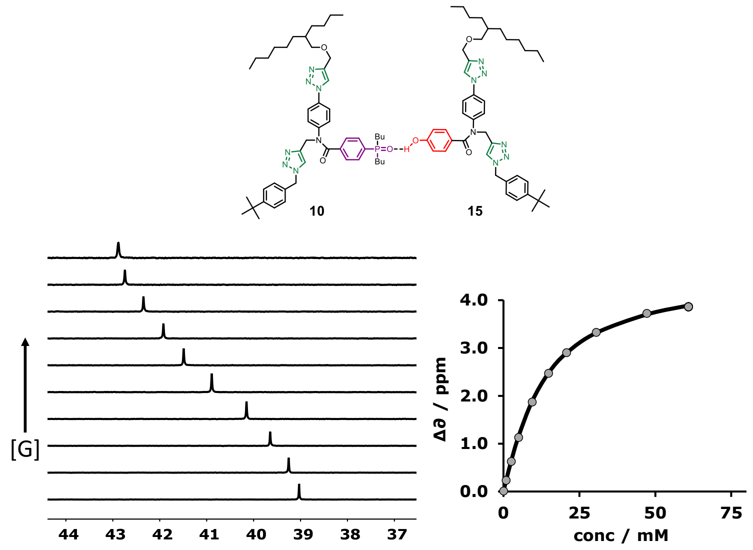

Figure S7. 202 MHz ^{31}P NMR spectra for titration of **15** into **10** (13.51 mM) at 298 K in CD_2Cl_2 and plot of the change in chemical shift of the ^{31}P signal as a function of guest concentration (the line represents the best fit to a 1:1 binding isotherm).

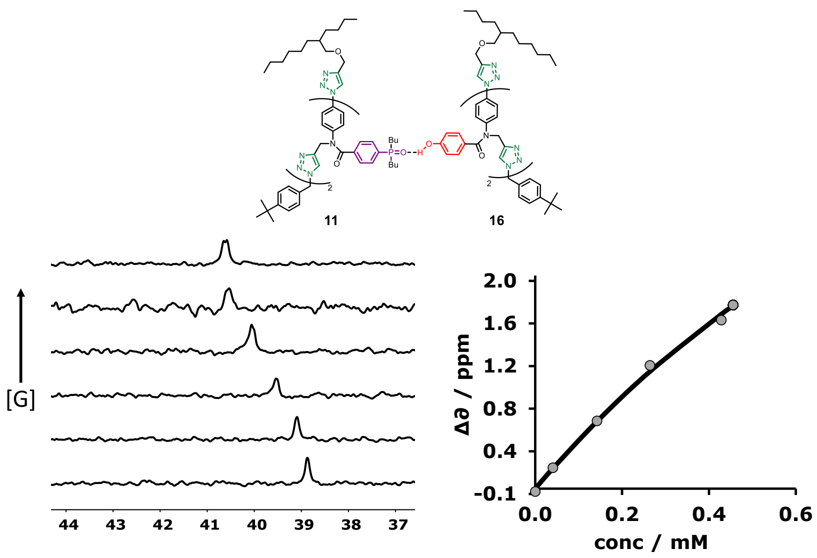

Figure S8. 202 MHz ^{31}P NMR spectra for titration of **16** into **11** (0.50 mM) at 298 K in CD_2Cl_2 and plot of the change in chemical shift of the ^{31}P signal as a function of guest concentration (the line represents the best fit to a 1:1 binding isotherm).

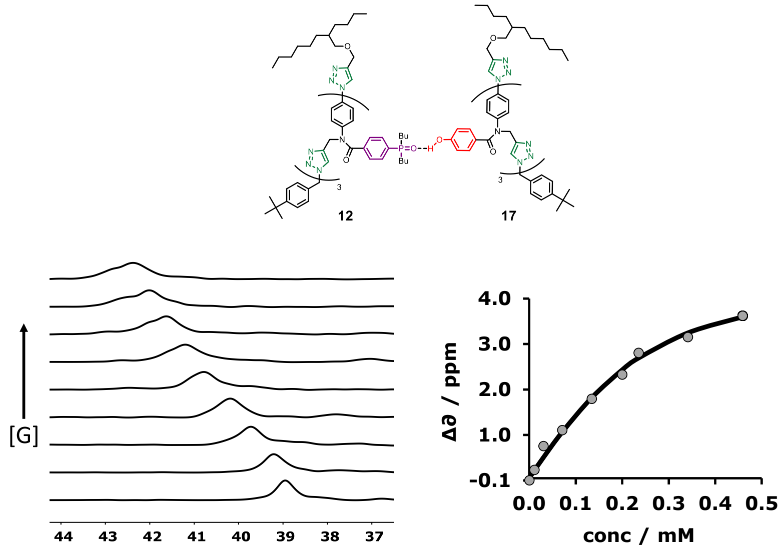

Figure S9. 202 MHz ^{31}P NMR spectra for titration of **17** into **12** (0.22 mM) at 298 K in CD_2Cl_2 and plot of the change in chemical shift of the ^{31}P signal as a function of guest concentration (the line represents the best fit to a 1:1 binding isotherm).

4.4. NMR dilution of AD 2-mer (20)

The ^{31}P and ^{1}H NMR dilution of AD 2-mer **20** was performed in a Bruker 500 MHz AVIII HD Smart Probe spectrometer. A stock solution of **20** in CD_2Cl_2 was prepared (6.29 mM). A known volume of neat CD_2Cl_2 (600 µL) was added to an NMR tube. Increasing known volumes of **20** were added from the stock solution and the spectrum recorded after each addition. The chemical shifts of the **20** were monitored as a function of its concentration and analyzed using a purpose written software in Microsoft Excel (fitted to a dimerization isotherm). Errors were

calculated as two times the standard deviation from the average value (95% confidence limit). Figure S10 shows the ^{31}P and ^{1}H NMR spectra and the plot of the change in chemical shift as a function of concentration for the dilution of **15**. The dimerization constant obtained is quoted in Table 1 in the main text.

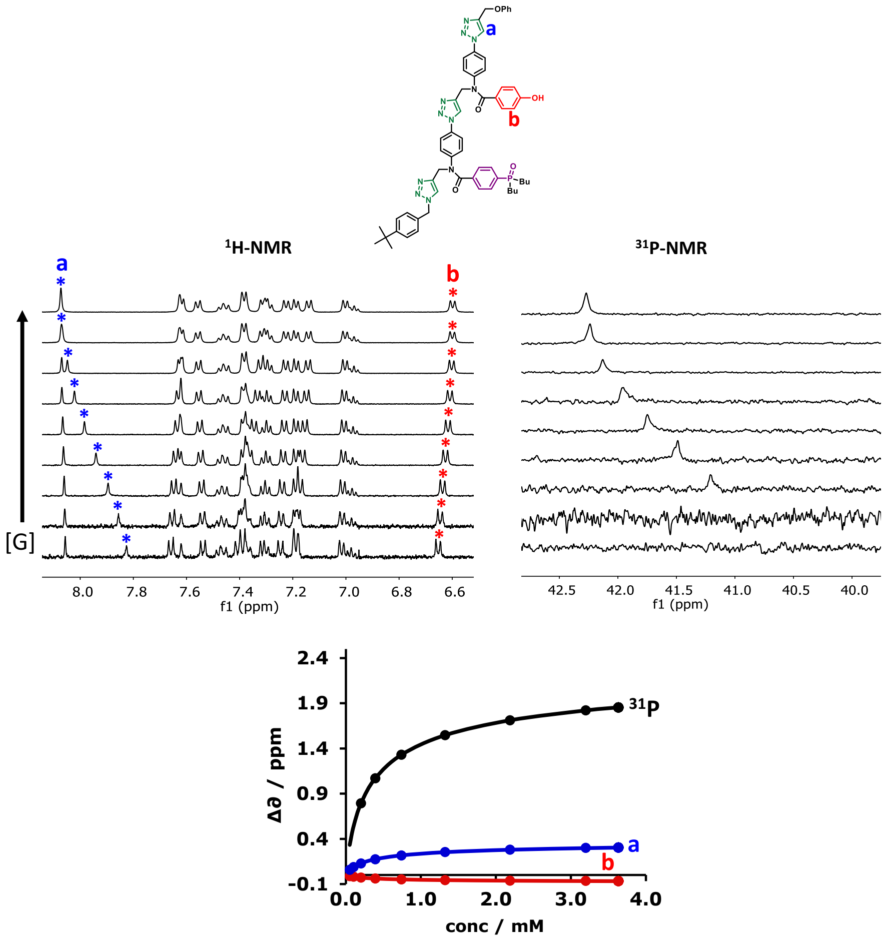

Figure S10. 500 MHz ^{1}H and 202 ^{31}P NMR spectra for dilution of **20** at 298 K in CD_2Cl_2 and plot of the change in chemical shift as a function of concentration (the line represents the best fit to a dimerization isotherm). The black curve corresponds to the ^{31}P signal, the blue one to a terminal triazole proton and the red one to the protons *ortho* to the phenol oxygen.

4.4. NMR duplex denaturation experiments

^{31}P NMR denaturation experiments were performed in a Bruker 500 MHz AVIII HD Smart Probe spectrometer. An equimolar solution of complementary homo-oligomers (phosphine oxide oligomers **11**-**13** and phenol oligomers **16**-**18**) was produced at a concentration of 1 mM in CD_2Cl_2. A known volume of solution was added to an NMR tube and the spectrum recorded. Known volumes of DMSO-d_6 in CD_2Cl_2 and neat DMSO-d_6 were added and the spectrum recorded after each addition. The chemical shifts of the acceptor homo-oligomer spectra were monitored as a function of DMSO-d_6 concentration. Free ^{31}P NMR shifts were monitored for a

1 mM solution of 1-mer phosphine oxide **10** in CD_2Cl_2 with the same concentrations of DMSO-d_6 to account for solvent effects.

Figure S11 shows the ^{31}P NMR spectra for the denaturation of 2-mer, 3-mer and 4-mer duplexes, including the titration of the single strand phosphine oxide 1-mer (**10**) to account for solvent effects. Figure S12 shows the plot of the change in ^{31}P chemical shift as a function of DMSO-d_6 concentration for the duplexes, after subtraction of the solvent effect measured by the free chemical shift of A (**10**) upon addition of DMSO-d_6. The denaturation data did not fit to a simple two-state isotherm for any of the duplexes so partially denatured species must be considered for the fitting (see references S4 and S5 for more details).

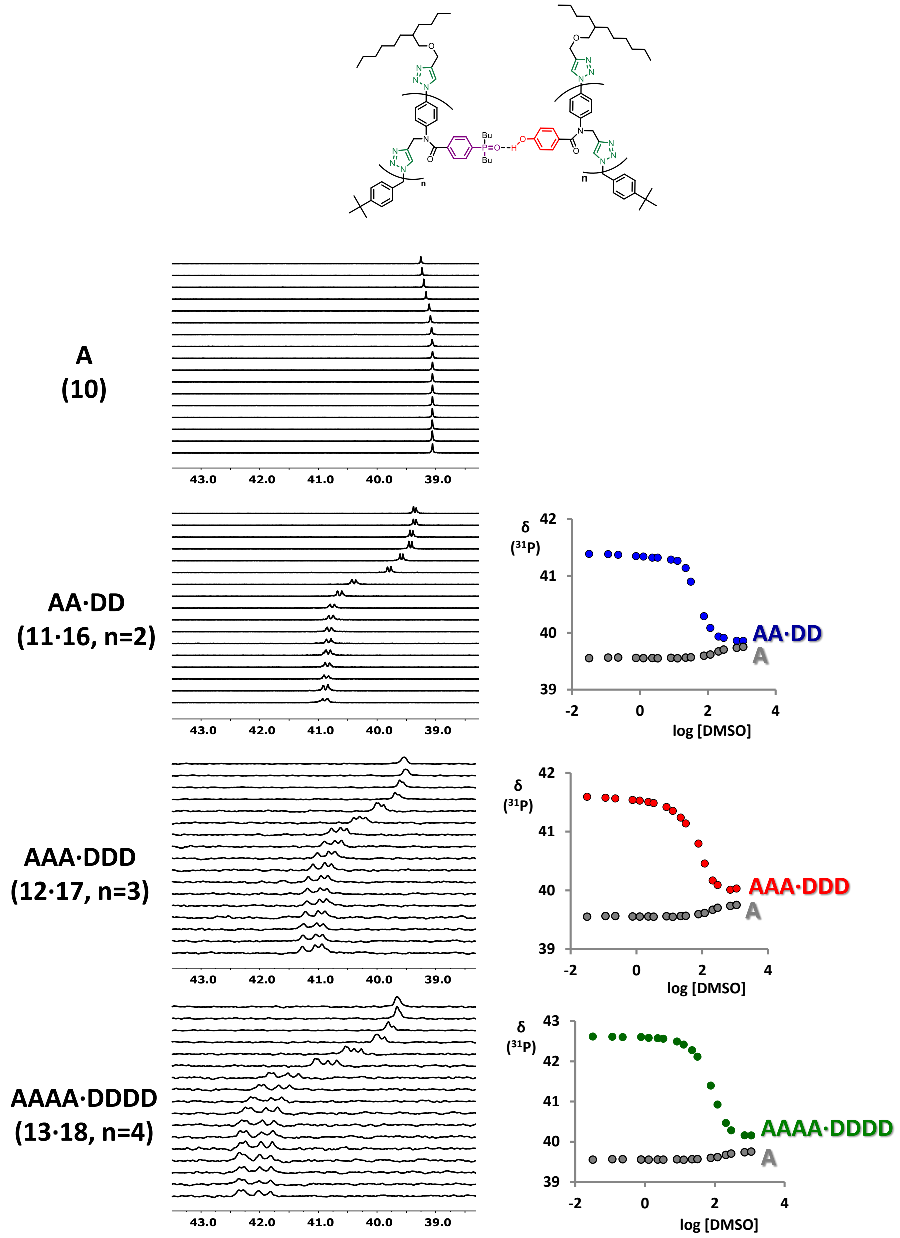

Figure S11. 202 MHz ^{31}P NMR spectra for titration of DMSO-d_6 into A (**10**, 1 mM), and DMSO-d_6 denaturation of equimolar 1 mM solutions of DD and AA (**11·16**), DDD and AAA (**12·17**), and DDDD and AAAA (**13·18**) in CD_2Cl_2 at 298 K. The plot of the change in ^{31}P chemical shift as a function of DMSO-d_6 concentration for A and the corresponding duplex is shown on the right-hand side.

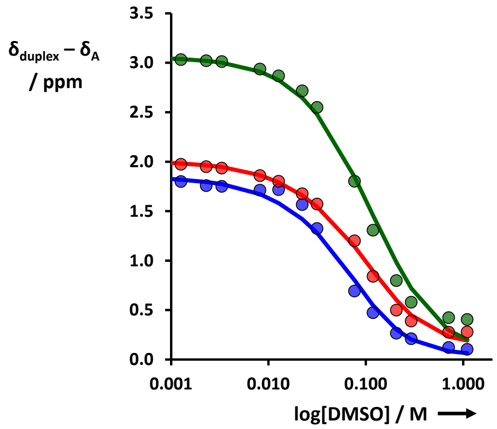

Figure S12. Duplex denaturation data plotted as a function of DMSO-d_6 concentration in CD_2Cl_2 at 298 K for AA·DD (blue), AAA·DDD (red), and AAAA·DDDD (green). The dots represent the experimental values and the lines are the calculated denaturation isotherms, taking into account partially denatured species.

5. Primer loading

Scheme S5 shows the sequence of reactions for the loading of the primer to template **21**: protection of the phenol groups (step 1), ester coupling of protected template **22** with phenol primer **19** (step 2) and deprotection of phenol groups (step 3). These three steps can be performed with just one chromatography at the end. Simple aqueous workups are enough to get pure intermediates **22** and **23**, as can be seen in the UPLC traces shown in Figure S13. Figure S13b-d show the crude reaction mixtures for the three steps while Figure S13e correspond to the UPLC trace of isolated **24**.

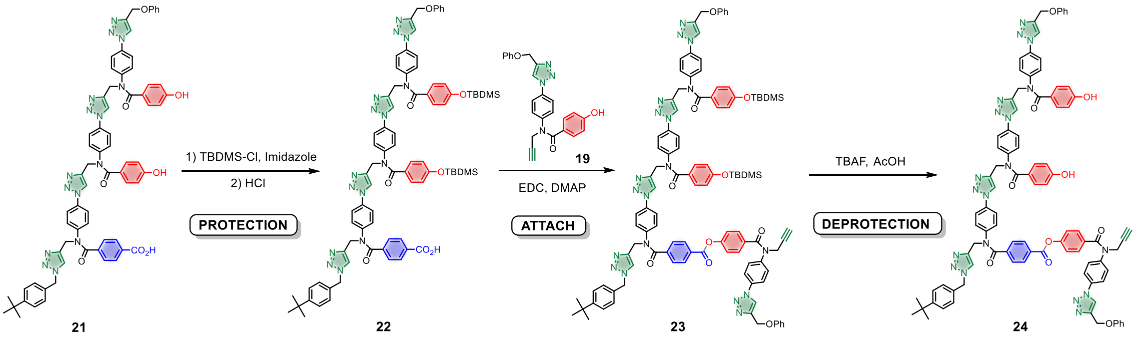

Scheme S5. Sequence of reactions for the covalent loading of primer **19** into template **21**.

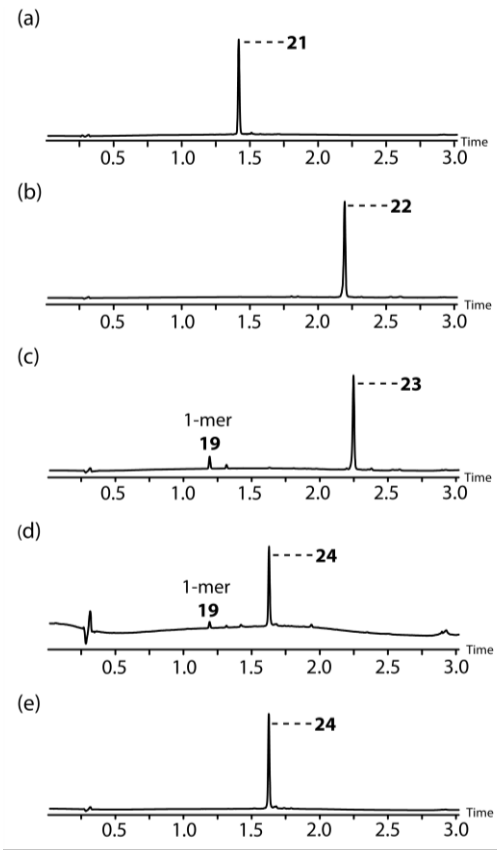

Figure S13. UPLC traces for the sequence of reactions used to load the primer **19** to the template **21**: starting template **21** (a); crude reaction mixtures for the protection of phenol groups (b), esterification of **22** with primer **19** (c) and phenol deprotection of phenol groups; isolated compound **24** (e). *Conditions:* C18 column at 40 °C (254 nm) using water + 0.1% formic acid (A) and CH_3CN + 0.1% formic acid (B) with flow rate of 0.6 ml/min; Gradient of 0-2 min 5% -100% B + 1 min 100% B.

STEP 1. Phenol protection

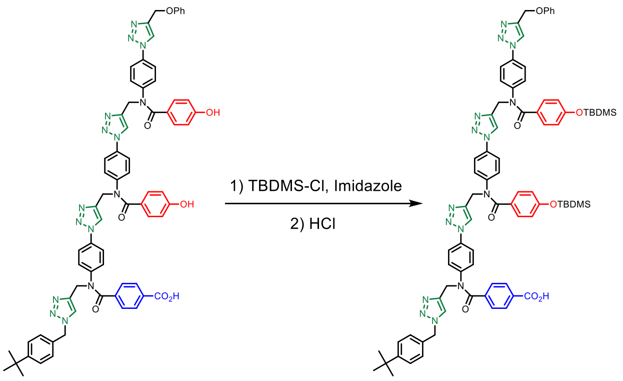

21 **22**

A solution of compound **21**[S1] (0.022 g, 0.018 mmol) in DMF (1 mL) was treated with imidazole (0.037 g, 0.541 mmol) and TBDMS-Cl (0.041 g, 0.270 mmol). After 15 h of stirring at room temperature, the reaction was quenched by addition of 0.1N HCl soln. until reaching pH= 3-4. The solution was stirred at room temperature for 45 min, then diluted with H_2O and extracted with EtOAc (3x). The combined organic layer was washed with 5% LiCl soln. (2x), H_2O (1x) and brine (1x), dried with anhydrous $MgSO_4$, filtered, and the solvent evaporated. The obtained residue (compound **22**) was used in the next step without further purification. UPLC traces shown below correspond to: (a) the starting template; (b) the obtained reaction crude after the protection step with the MS of the product obtained (compound **22**, MW: 1454.8). *UPLC Conditions*: C18 column at 40 °C (254 nm) using water + 0.1% formic acid (A) and CH_3CN + 0.1% formic acid (B) with flow rate of 0.6 ml/min; Gradient of 0-2 min 5% -100% B + 1 min 100% B.

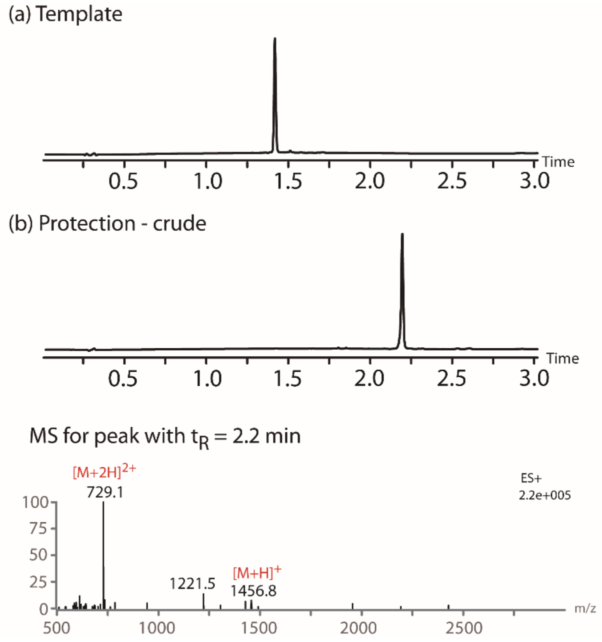

STEP 2. Covalent attachment of the primer

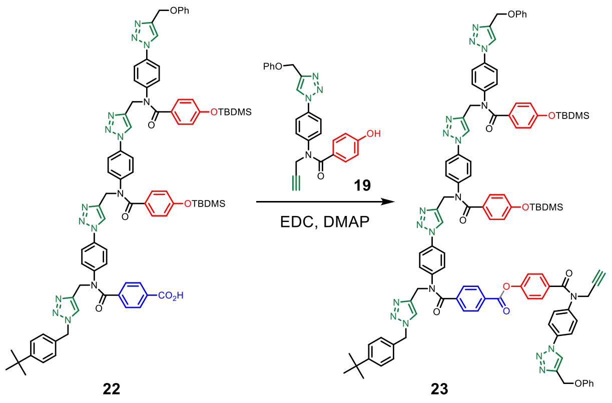

Compound **22** (0.018 mmol), compound **19** (0.008 g, 0.018 mmol), EDC (0.007 g, 0.036 mmol) and DMAP (0.004 g, 0.036 mmol) were dissolved in dry CH_2Cl_2 (1 mL) under N_2 atmosphere. The reaction was stirred overnight at room temperature. Once finished, the reaction was diluted with EtOAc and washed with 0.1N HCl soln. (2x), H_2O (2x) and brine (1x). The solution was dried with anhydrous $MgSO_4$, filtered and the solvent evaporated. The obtained residue (compound **23**) was used in the next step without further purification. UPLC traces shown below correspond to: (a) the starting carboxylic acid derivative; (b) the obtained reaction crude after the coupling step with the MS of the product obtained (compound **23**, MW: 1861.3). *UPLC Conditions*: C18 column at 40 °C (254 nm) using water + 0.1% formic acid (A) and CH_3CN + 0.1% formic acid (B) with flow rate of 0.6 ml/min; Gradient of 0-2 min 5% -100% B + 1 min 100% B.

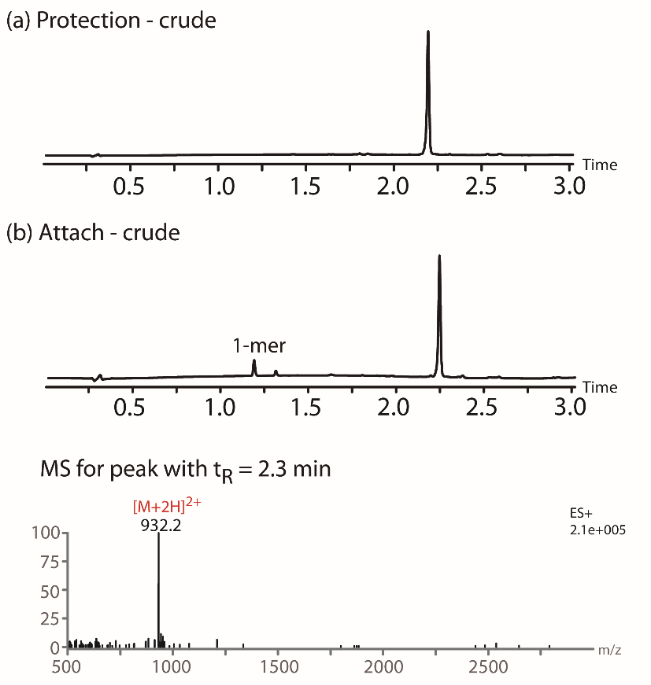

STEP 3: Deprotection of phenol units

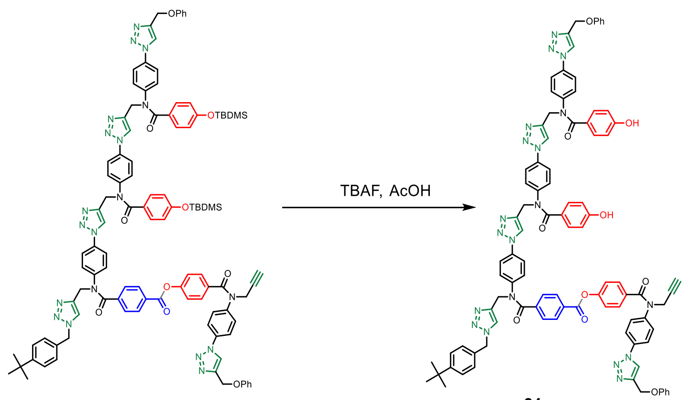

23 24

Compound **23** (0.018 mmol) was dissolved in dry THF (1 mL) under N_2 atmosphere. After cooling down the solution to 0 °C, acetic acid (2 µL, 0.036 mmol) and TBAF (1M in THF, 36 µL, 0.036 mmol) were added. After 10 minutes of stirring at 0 °C, the reaction was quenched with 0.1N HCl soln. and diluted with EtOAc. The organic layer was separated and washed with 0.1N HCl soln. (2x), H_2O (1x) and brine (1x). The solution was dried with anhydrous $MgSO_4$, filtered and the solvents evaporated. The obtained residue was purified by flash chromatography (grandient from 0% to 65% of EtOAc in pet. ether followed by a gradient from 0% to 7% of MeOH in CH_2Cl_2) to afford compound **24** (0.019 g, 65% over three steps) as a white amorphous solid. UPLC traces shown below correspond to: (a) the starting protected derivative; (b) the obtained reaction crude and (c) pure compound after the deprotection step with the MS of the product obtained (compound **24**, MW: 1632.8). *UPLC Conditions*: C18 column at 40 °C (254 nm) using water + 0.1% formic acid (A) and CH_3CN + 0.1% formic acid (B) with flow rate of 0.6 ml/min; Gradient of 0-2 min 5% -100% B + 1 min 100% B.

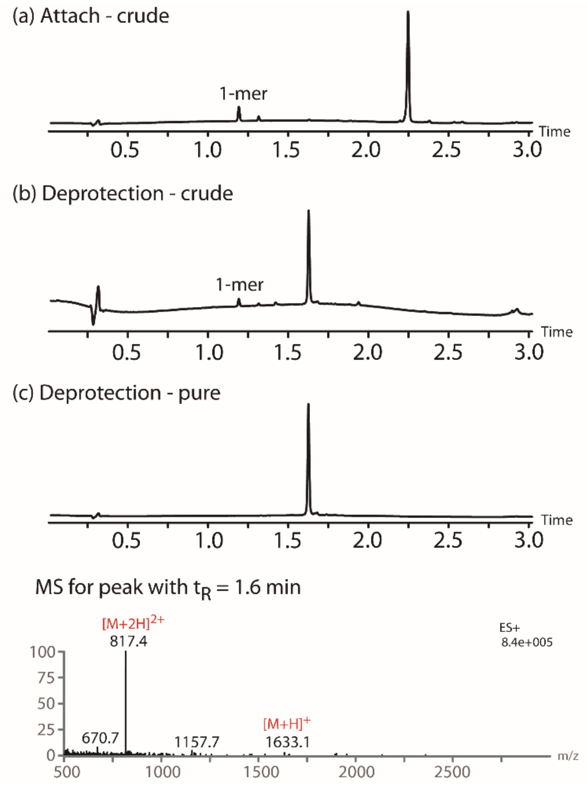

Characterization of compound 24

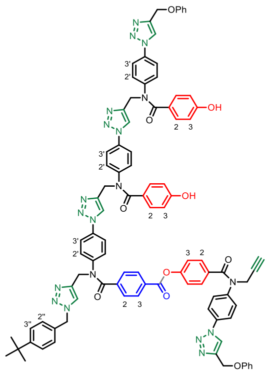

^{1}H NMR (500 MHz, CDCl$_3$): δ_H = 8.14 (s, 1H, CH$_{triaz}$, internal), 8.01 (s, 1H, CH$_{triaz}$, PhO cap), 8.00 (s, 1H, CH$_{triaz}$, PhO cap), 7.90 (d, 2H, J = 8.5 Hz, 3-H, ester), 7.67 (s, 1H, CH$_{triaz}$, ^{t}Bu cap), 7.63 (d, 2H, J = 9.0 Hz, 3'-H), 7.54 (s, 1H, CH$_{triaz}$, internal), 7.52 (m, 4H, 3'-H), 7.37 (m, 6H, 2-H, primer; 2-H, ester; 3''-H, ^{t}Bu cap), 7.26 (m partially overlapped, 12H, 2'-H; 3''-H, PhO caps), 7.19 (d, 2H, J = 8.5 Hz, 2''-H, ^{t}Bu cap), 7.12 (d, 2H, J = 8.5 Hz, 2-H, phenol), 7.09 (d, 2H, J = 8.5 Hz, 2-H, phenol), 7.00 (d, 2H, J = 6.5 Hz, 3-H, primer), 6.95 (m, 6H, 2''-H and 4''-H, PhO cap), 6.57 (d, 2H, J = 8.5 Hz, 3-H, phenol), 6.55 (d, 2H, J = 8.5 Hz, 3-H, phenol), 5.46 (s, 2H, N-CH$_2$, ^{t}Bu cap), 5.22 (s, 2H, O-CH$_2$, PhO cap), 5.21 (s, 2H, O-CH$_2$, PhO cap), 5.15 (s, 2H, N-CH$_2$, internal), 5.13 (s, 2H, N-CH$_2$, internal), 5.11 (s, 2H, N-CH$_2$, terminal), 4.67 (d, 2H, J = 2.5 Hz, N-CH$_2$, alkyne), 2.27 (t, 1H, J = 2.5 Hz, CH, alkyne), 1.29 (s, 9H, ^{t}Bu).

^{13}C NMR (125.7 MHz, CDCl$_3$): δ_C = 170.5 and 170.4 (CO amide, phenol), 169.5 (CO amide, primer), 169.2 (CO amide, acid), 163.7 (CO, ester), 159.3 and 159.2 (4-C, phenol), 158.2 and 158.1 (1''-C, PhO caps), 152.2 (4-C, primer), 152.2 (4''-C, ^{t}Bu cap), 145.4 and 145.2 (C$_{triaz}$, PhO caps), 144.9, 144.9, 144.8 and 144.7 (C$_{triaz}$, internal; 1'-C, internal), 143.7 (C$_{triaz}$, ^{t}Bu cap), 143.4 (1'-C, terminal), 143.0 (1'-C, primer), 140.4 (1-C, ester), 135.6, 135.4, 134.8 and 134.7 (4'-C), 132.2 (1-C, ester), 131.4 and 131.3 (2-C, phenol), 130.5, 130.4, 130.0, 129.8, 129.8, 129.1, 129.0, 128.9, 128.5 and 128.4 (C$_{arom}$), 128.1 (2''-C, ^{t}Bu cap), 126.2 (3''-C, ^{t}Bu cap), 125.8 and 125.7 (1-C, phenol), 124.0 (CH$_{triaz}$, ^{t}Bu cap), 122.3, 121.6, 121.6, 121.5, 121.4, 121.3, 121.2 and 121.1 (C$_{triaz.}$; 3'-C, internal), 115.2 (3-C, phenol), 114.9 and 114.9 (2''-C, PhO caps), 78.5 (C, alkyne), 73.3 (CH, alkyne), 61.8 and 61.8 (O-CH$_2$), 54.2 (N-CH$_2$, ^{t}Bu cap), 46.5, 46.5, 46.4 and 46.1 (N-CH$_2$), 40.1 (N-CH$_2$, primer), 34.8 (C, ^{t}Bu), 31.4 (CH$_3$, ^{t}Bu).

HRMS (ES+): calcd $C_{94}H_{78}N_{19}O_{10}$ 1632.6174 [M+H]$^+$, found 1632.6138 [M+H]$^+$.

FT-IR (ATR): ν_{max} 2955, 2849, 1739, 1643, 1604, 1518, 1381, 1237 and 755 cm^{-1}.

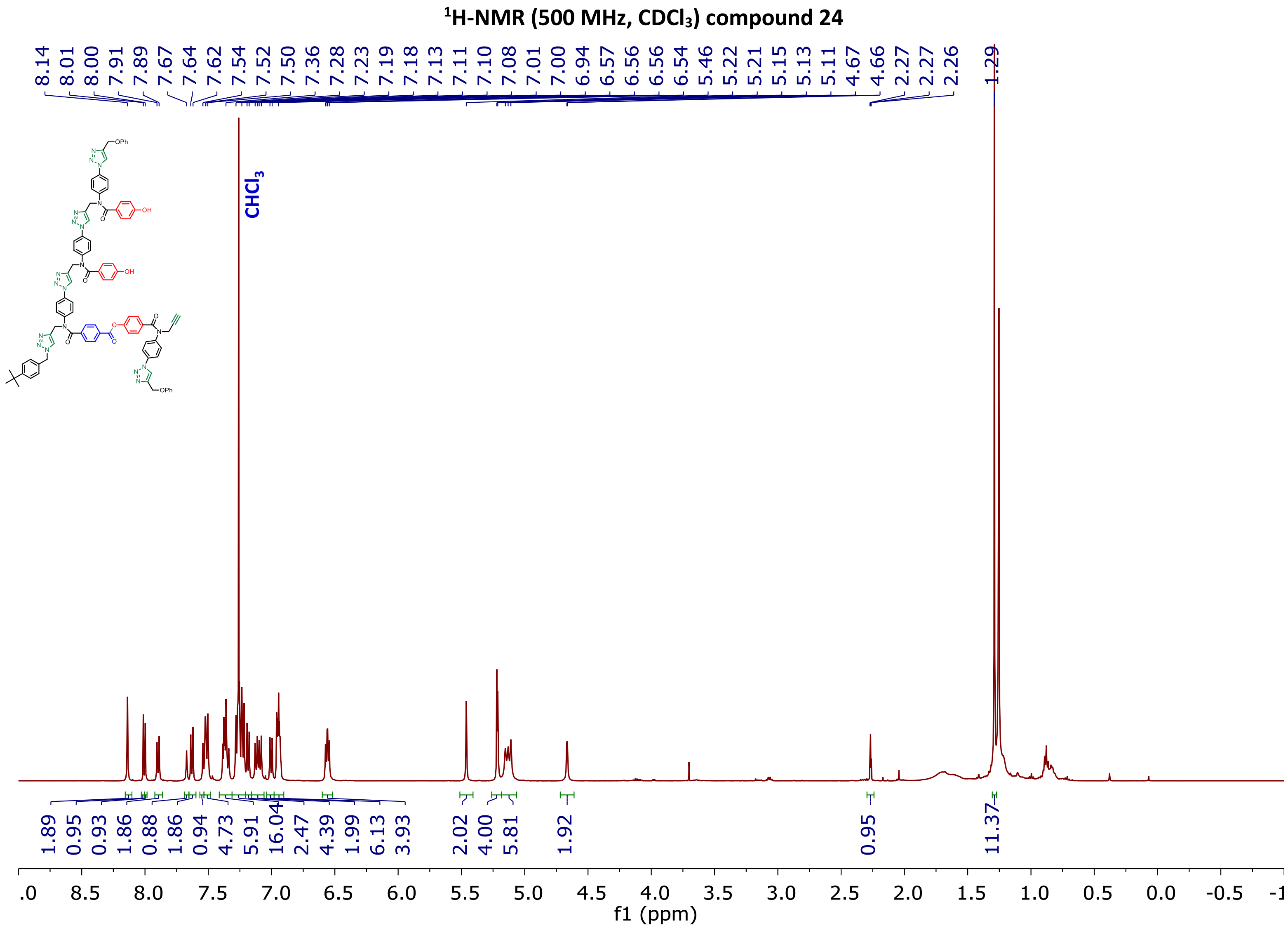

^{1}H-NMR (500 MHz, $CDCl_3$) compound 24
8.14 8.01 8.00 7.91 7.89 7.67 7.64 7.62 7.54 7.52 7.50 7.36 7.28 7.23 7.19 7.18 7.13 7.11 7.10 7.08 7.01 7.00 6.94 6.57 6.56 6.56 6.54 5.46 5.22 5.21 5.15 5.13 5.11 4.67 4.66 2.27 2.27 2.26 1.29
$CHCl_3$
1.89 0.95 0.93 1.86 0.88 1.86 0.94 4.73 5.91 16.04 2.47 4.39 1.99 6.13 3.93 2.02 4.00 5.81 1.92 0.95 11.37
f1 (ppm)

^{13}C-NMR (125.7 MHz, $CDCl_3$) compound 24

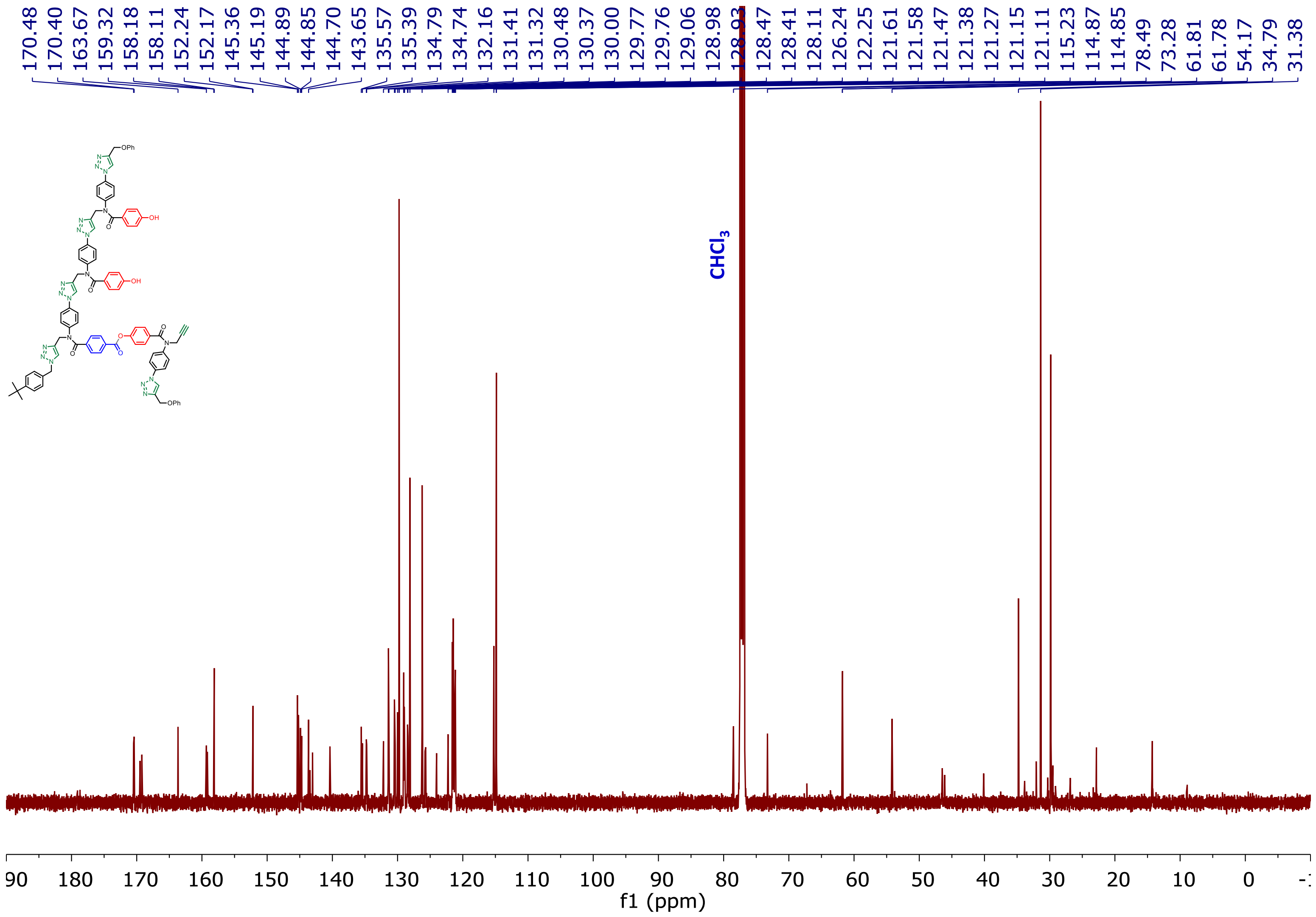

6. Template-directed synthesis

6.1. Calculation of template effect

The template effect in the synthesis of complementary daughter strands was calculated from the UPLC traces shown in Figure 6, along with the corresponding control experiments using a non-coding alkyne (see ESI for details). For each experiment, we recorded a UPLC trace of the starting mixture prior to the addition of the copper(II) catalyst. In order to counter the difference in extinction coefficient for the templated (**27** or **28**) and non-templated (**26**) products, we monitored the disappearance of azide-derived phenol **25** and phosphine oxides **5** or **7**, used in excessed in regard to alkyne **24**. The initial chromatogram areas for these compounds (A_0) are expressed in equations S1 and S2, where *c* is the path length, ε is the extinction coefficient and $[\text{phenol}]_0$ and $[\text{PO}]_0$ are the initial concentrations of starting materials:

$$\text{A}_0\ (phenol) = c\ [\text{phenol}]_0\ \varepsilon_{phenol} \qquad \text{Eq. S1}$$

$$\text{A}_0\ (POx) = c\ [\text{PO}]_0\ \varepsilon_{PO} \qquad \text{Eq. S2}$$

The areas corresponding to the remaining of starting materials once the reaction is completed (A_f) are expressed in equations S3 and S4, where $[\text{phenol}]_f$ and $[\text{PO}]_f$ are their final concentrations:

$$\text{A}_\text{f}\ (phenol) = c\ [\text{phenol}]_\text{f}\ \varepsilon_{phenol} \qquad \text{Eq. S3}$$

$$\text{A}_\text{f}\ (POx) = c\ [\text{PO}]_\text{f}\ \varepsilon_{PO} \qquad \text{Eq. S4}$$

Equation S5 shows the ratio of phenol and phosphine oxide starting materials before and after reaction (χ):

$$\chi = \frac{\text{A}_\text{f}\ (phenol)\ /\ \text{A}_\text{f}\ (PO)}{\text{A}_0\ (phenol) /\ \text{A}_0\ (PO)} = \frac{(c\ [\text{phenol}]_\text{f}\ \varepsilon_{phenol})/(c'\ [\text{PO}]_\text{f}\ \varepsilon_{PO})}{(c\ [\text{phenol}]_0\ \varepsilon_{phenol})/(c'\ [\text{PO}]_0\ \varepsilon_{PO})} = \frac{[\text{phenol}]_\text{f}\ /[\text{PO}]_\text{f}}{[\text{phenol}]_0\ /\ [\text{PO}]_0\ )} \qquad \text{Eq. S5}$$

From equation S5, χ can be therefore written in terms of molar fraction of starting and final phenol and phosphine oxide (equation S6):

$$\chi = \frac{\text{phenol}_\text{f}\ /\text{PO}_\text{f}}{\text{phenol}_0\ /\text{PO}_0} \qquad \text{Eq. S6}$$

Both azide starting materials are used in 1.5-fold excess in regard to the alkyne so if *phenol*$_0$ and *PO*$_0$ are 1.5 then equation S6 can be written as:

$$\chi = \frac{\text{phenol}_\text{f}}{\text{PO}_\text{f}} \qquad \text{Eq. S7}$$

On the other hand, the molar fraction of the remaining starting materials after the reaction can be expressed from the starting molar fractions as shown in equation S8:

$$phenol_f + PO_f = phenol_0 + PO_0 - 1 \qquad \text{Eq. S8}$$

As $phenol_0$ = PO_0 = 1.5, then the remaining molar fraction ($phenol_f$ and PO_f) can be expressed as shown in equations S9 and S10:

$$phenol_f = 2 - PO_f = \frac{2\chi}{1+\chi} \quad \text{Eq. S9}$$

$$PO_f = 2 - phenol_f = \frac{2}{1+\chi} \quad \text{Eq. S10}$$

Thus, the molar fractions $phenol_f$ and PO_f can be expressed as a function of χ, which is obtained directly from the chromatogram areas before and after the reaction takes places. Using equations 8-10, the amount of azide-derived phenol and phosphine oxide consumed in the reaction can be expressed as:

$$phenol_{reacted} = phenol_0 - phenol_f = phenol_0 - \chi \frac{PO_0 + phenol_0 - 1}{1+\chi} \quad \text{Eq. S11}$$

$$PO_{reacted} = PO_0 - PO_f = PO_0 - \frac{PO_0 + phenol_0 - 1}{1+\chi} \quad \text{Eq. S12}$$

The template effect can be assessed from the rate acceleration of the reaction in the presence of the template (*k'*). This in turn can be calculated from equation S11 and S12 by comparing the rate of the templating reaction (K_{obs}) with the control one ($k_{control}$). K_{obs} can be expressed as the ratio of the reaction rate of the phosphine oxide and the phenol, taking into consideration than the rate for phosphine oxide is the contribution of uncatalyzed and the template-catalysed reaction, while the rate of reaction of the phenol is only uncatalyzed (equation S13):

$$k_{obs} = \frac{k_{uncat}(PO) + k_{cat}(PO)}{k_{uncat}(phenol)} = \frac{PO_{reacted}}{phenol_{reacted}} \quad \text{Eq. S13}$$

In the control reaction, there is no template-directed enhancement of the reaction rate so the $k_{control}$ can be expressed as in equation S14:

$$k_{control} = \frac{k_{uncat}(PO)}{k_{uncat}(phenol)} = \frac{PO_{reacted}}{phenol_{reacted}} \quad \text{Eq. S14}$$

k' is defined as:

$$k' = \frac{k_{cat}(PO)}{k_{uncat}(PO)} \quad \text{Eq. S15}$$

Using equations S13 and S14, equation S15 can be therefore expressed as:

$$k' = \frac{k_{obs}}{k_{control}} - 1 \quad \text{Eq. S16}$$

6.2. Templating reaction using 1-mers 25 and 5

Compound **24** is used as template for the template-directed synthesis of its complementary duplex from the alkyne functionality provided by the primer. Two different azides are used: **5** is the complementary phosphine oxide 1-mer while **25** is the non-complementary one. CuAAC reaction with **24** leads to the formation of two products: **26** is the non-templated product from the reaction of **24** and **25** while **27** is the templated product from the reaction of **24** and **5** (Scheme S6). In this reaction, the template effect provided by H-bonding between the phenol groups in the template and the phosphine oxide of the 1-mer is studied by comparison with a control reaction where no template is present. As shown in Scheme S7, a simple alkyne (**S6**) is used as control in order to calculate the template effect (as explained later in this section).

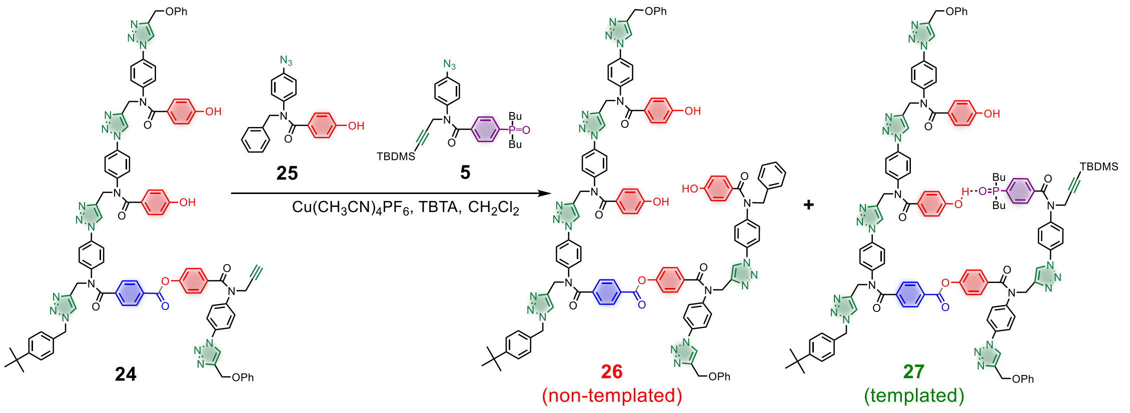

Scheme S6. Template-directed synthesis of the sequence complementary duplex from **24** using 1-mers **25** and **5**.

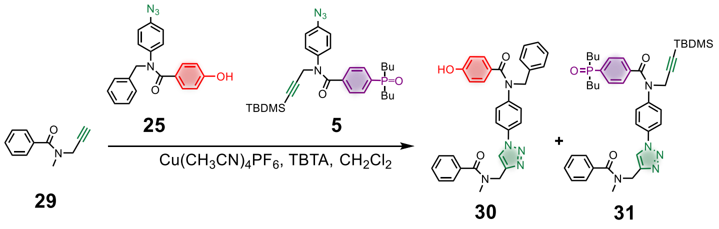

Scheme S7. Control reaction where no template effect is possible.

General procedure for the templating and control reactions shown in schemes S6 and S7.

From freshly prepared stock solutions in dry CH_2Cl_2, the starting alkyne (**24** or **29**, $3.4{\cdot}10^{-5}$ mmol), **25** (0.017 mg, $5.1{\cdot}10^{-5}$ mmol) and **5** (0.028 mg, $5.1{\cdot}10^{-5}$ mmol) were mixed in a 1.75 mL vial containing a magnetic stirrer. The solvent was evaporated under N_2 stream and dry CH_2Cl_2 (0.34 mL) was added. A 30 µL aliquot was taken for UPLC analysis (t_0). To this solution, a premixed solution of $Cu(CH_3CN)_4PF_6$ (0.022 mg, $6.8{\cdot}10^{-5}$ mmol) and TBTA (0.032 mg, $6.8{\cdot}10^{-5}$ mmol) in dry CH_2Cl_2 (10 µL) was added. The vial was flushed briefly with N_2, sealed and left stirring at room temperature for 2 days. Another 30 µL aliquot was taken after this time for UPLC analysis (t_f). Figure S14 shows the UPLC traces for the templating and control experiments, corresponding to three repetitions of the experiment. Table S1 includes the peak areas from these chromatograms for phenol **25** and phosphine oxide **5** before (t_0) and after (t_f)

Cu-TBTA was added along with the calculated k' (see previous section 6.1 for the equations to calculate *k'*).

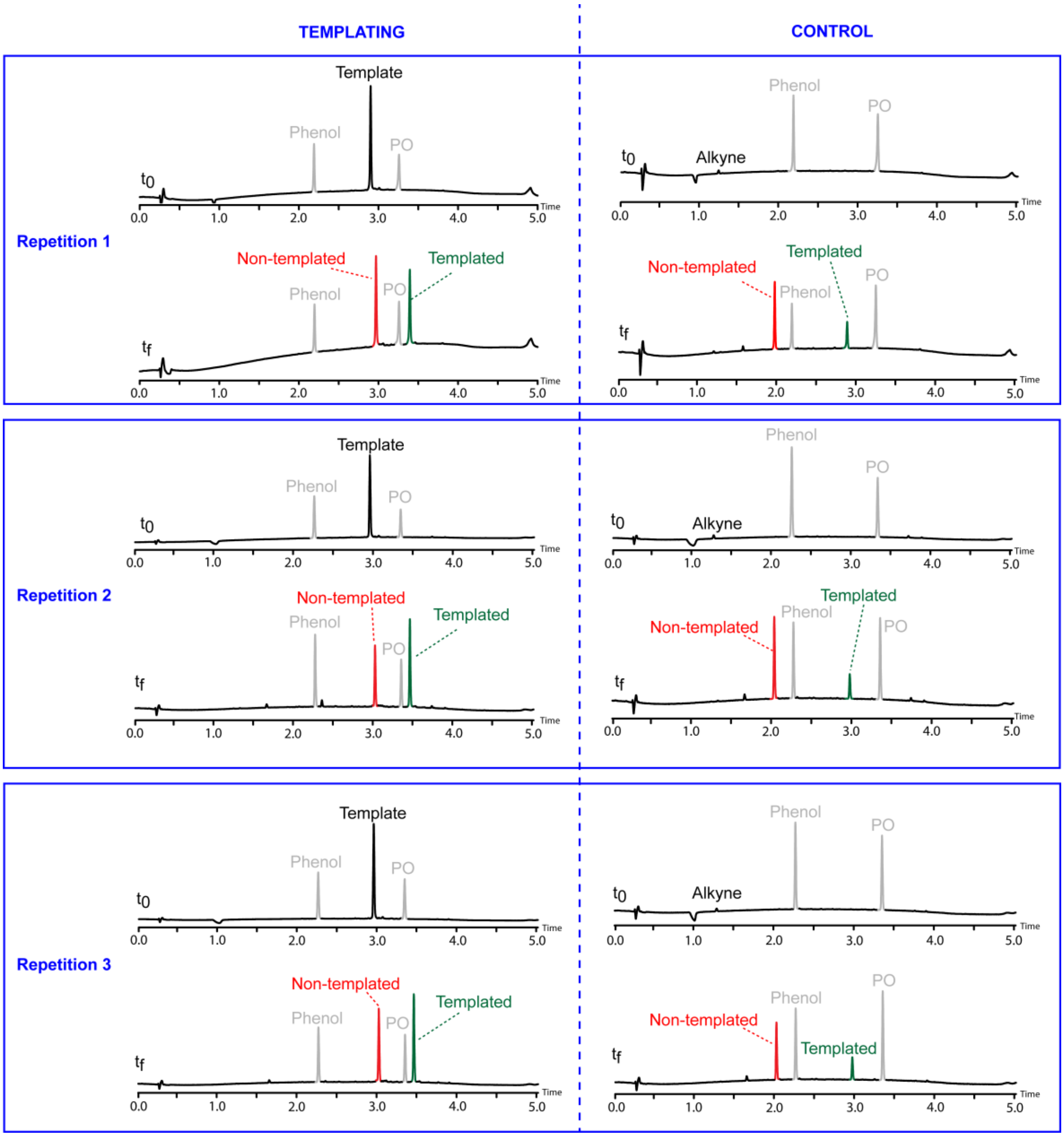

Figure S14. UPLC traces for three repetitions of the templating (left, scheme S6) and control (right, scheme S7) reactions before (t_0) and after (t_f) Cu-TBTA was added. All the peaks are labelled: Phenol (compound **25**), PO (compound **5**), template (compound **24**), alkyne (compound **S6**), non-templated (compound **26** for the templating reactions on the left; compound **S7** for the control reactions on the right) and templated (compound **27** for the templating reactions on the left; compound **S8** for the control reactions on the right). *Conditions:* C18 column at 40 °C (254 nm) using water + 0.1% formic acid (A) and CH_3CN + 0.1% formic acid (B) with flow rate of 0.6 ml/min; Gradient of 0-4 min 5% -100% B + 1 min 100% B.

Table S1. Peak areas from the UPLC chromatograms shown in Figure S14 for phenol **25** and phosphine oxide **5** before (t_0) and after (t_f) Cu-TBTA was added along with calculated *k'*.

			Phenol 25	Phosph. ox. 5	χ	*k*	*k'*
Repetition 1	Templating	t_0	633	596	0.81	0.72	1.00
		t_f	469	543			
	Control	t_0	1075	1072	0.54	0.36	
		t_f	469	857			
Repetition 2	Templating	t_0	2872	1898	0.97	0.94	1.64
		t_f	1965	1336			
	Control	t_0	3503	2356	0.62	0.36	
		t_f	1177	1281			
Repetition 3	Templating	t_0	3306	2838	0.96	0.93	1.06
		t_f	1735	1547			
	Control	t_0	1981	1716	0.68	0.45	
		t_f	1450	1844			
				K' average			1.23 ± 0.71

Synthesis of non-templated product 26 (for characterization purposes)

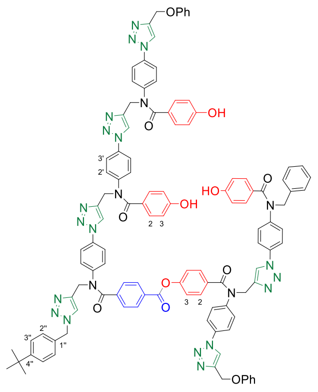

A solution of **24** (2.3 mg, 1.4·10^{-3} mmol) and **25** (1 mg, 2.8·10^{-3} mmol) in CH_2Cl_2 (2 mL) under N_2 atmosphere was treated with $Cu(CH_3CN)_4PF_6$ (2.1 mg, 5.6·10^{-3} mmol) and TBTA (3.0 mg, 5.6·10^{-3} mmol). The solution was stirred overnight at room temperature. The reaction was then diluted with EtOAc and washed with EDTA soln. (2x), H_2O (1x) and brine. The organic layer was dried over $MgSO_4$ and concentrate under vacuum. The residue was purified by flash column chromatography on silica gel (gradient from 0% to 10% of MeOH in CH_2Cl_2) to afford **26** (3 mg, 94%) as a white amorphous solid. UPLC traces shown below correspond to: (a) the starting materials; (b) the obtained reaction crude and (c) the isolated compound **26**. *UPLC Conditions*: C18 column at 40 °C (254 nm) using water + 0.1% formic acid (A) and CH_3CN + 0.1% formic acid (B) with flow rate of 0.6 ml/min; Gradient of 0-4 min 5% -100% B + 1 min 100% B.

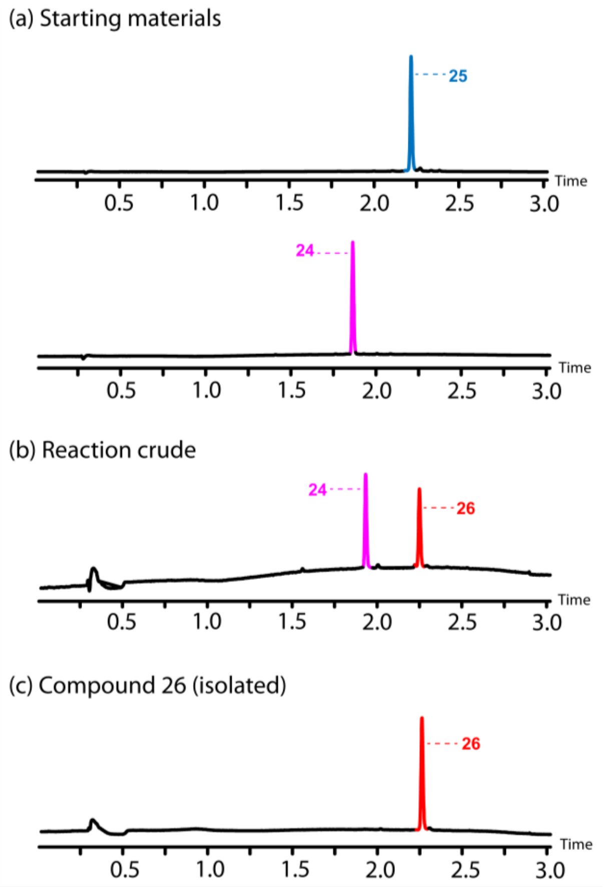

Characterization of compound 26

^{1}H NMR (500 MHz, $CDCl_3$): δ_H = 8.16 (s, 1H, CH_{triaz}), 8.13 (s, 1H, CH_{triaz}), 8.11 (s, 1H, CH_{triaz}), 8.00 (s, 1H, CH_{triaz}), 7.96 (s, 1H, CH_{triaz}), 7.89 (d, 2H, *J* = 8.0 Hz, 3-H, ester), 7.67 (s, 1H, CH_{triaz}), 7.55 (d, 2H, *J* = 8.5 Hz, 3'-H), 7.53 (d, 2H, *J* = 8.5 Hz, 3'-H), 7.47 (m, 4H, 3'-H), 7.40 (d, 2H, *J* = 8.5 Hz, 3'-H), 7.37 (d, 2H, *J* = 8.5 Hz, 3''-H, ^{t}Bu caps), 7.31 (m, 6H, 2-H, ester; 2'-H), 7.21 (m, 19H, 2-H, phenol; 2-H, primer; Bn; 2'-H; 3''-H, PhO cap; 2''-H, ^{t}Bu caps), 7.13 (d, 2H, *J* = 8.5 Hz, 2-H, phenol), 7.03 (m, 4H, 2-H, phenol; 2'-H), 6.97 (d, 2H, *J* = 8.0 Hz, 2''-H, PhO), 6.94 (m, 6H, 3-H, primer; 2''-H and 4''-H, PhO cap), 6.58 (d, 2H, *J* = 8.0 Hz, 3-H, phenol), 6.56 (d, 2H, *J* = 8.5 Hz, 3-H, phenol), 6.52 (d, 2H, *J* = 8.0 Hz, 3-H, phenol), 5.47 (s, 2H, N-CH_2, ^{t}Bu cap), 5.23 (s, 4H, O-CH_2, PhO cap), 5.16 (s, 4H, N-CH_2), 5.12 (s, 4H, N-CH_2), 1.30 (s, 9H, ^{t}Bu).

^{13}C NMR (125.7 MHz, $CDCl_3$): δ_C = 170.8, 170.3, 169.7 and 169.3 (CO, amide), 163.7 (CO, ester), 159.4 and 159.3 (4-C, phenol), 158.8 and 158.2 (1''-C, PhO caps), 152.3 and 152.2 (4-C, primer; 4''-C, ^{t}Bu cap), 145.3, 145.2, 145.0, 144.9, 144.7, 144.4, 144.1, 143.7 and 143.5 (1'-C; C_{triaz}), 140.6 (1-C, ester), 137.2, 135.4, 135.1, 134.9, 134.7 and 134.4 (4-C; 4'-C), 132.1 (1-C, primer), 131.4 (1''-C, ^{t}Bu cap), 131.3, 131.2, 130.5, 130.2, 130.0, 129.8, 129.1, 128.9, 128.8, 128.8, 128.5, 128.4, 128.1, 127.8 and 126.5 (C_{arom}), 126.3 (3''-C, ^{t}Bu cap), 125.8 and 125.7 (1-C, phenol), 123.9, 122.8, 122.5, 122.1, 121.8, 121.6, 121.5, 121.4, 121.1 and 120.9 (CH_{triaz}; 3'-C), 115.4, 115.3 and 115.2 (3-C, phenol) 115.1 and 114.9 (4''-C, PhO caps), 61.8 and 61.7 (O-CH_2, PhO caps), 54.2 and 54.0 (N-CH_2, ^{t}Bu cap; Bn), 46.6, 46.5, 46.4, 46.4 and 46.0 (N-CH_2), 34.8 (C, ^{t}Bu), 31.4 (CH_3, ^{t}Bu).

HRMS (ES+): calcd $C_{114}H_{94}N_{23}O_{12}$ 1976.7447 $[M+H]^+$, found 1976.7373 $[M+H]^+$.

FT-IR (ATR): ν_{max} 2952, 2922, 2852, 1736, 1642, 1605, 1517, 1237, 845 and 730 cm^{-1}.

^{1}H-NMR (500 MHz, $CDCl_3$) compound 26

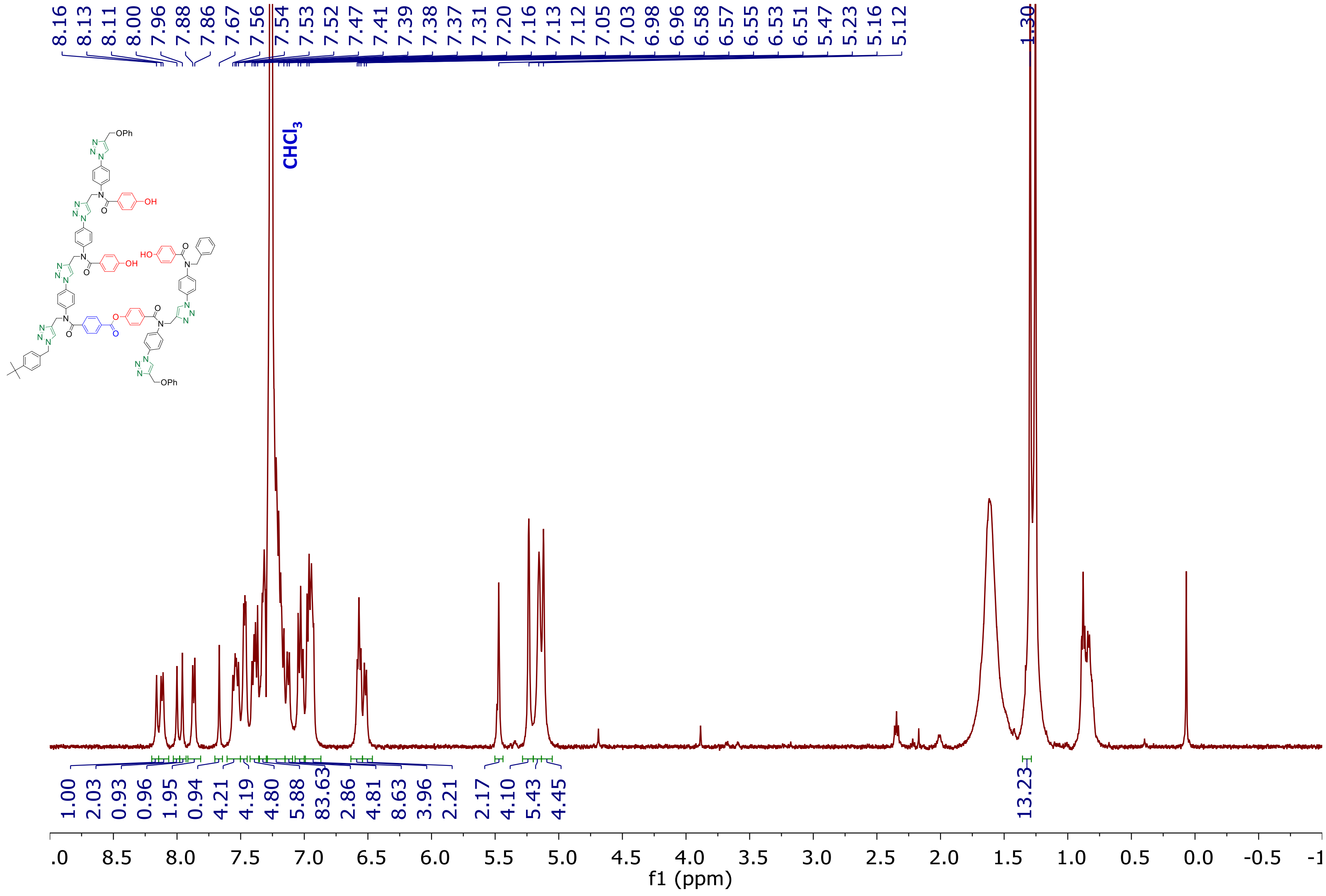

^{13}C-NMR (125.7 MHz, $CDCl_3$) compound 26

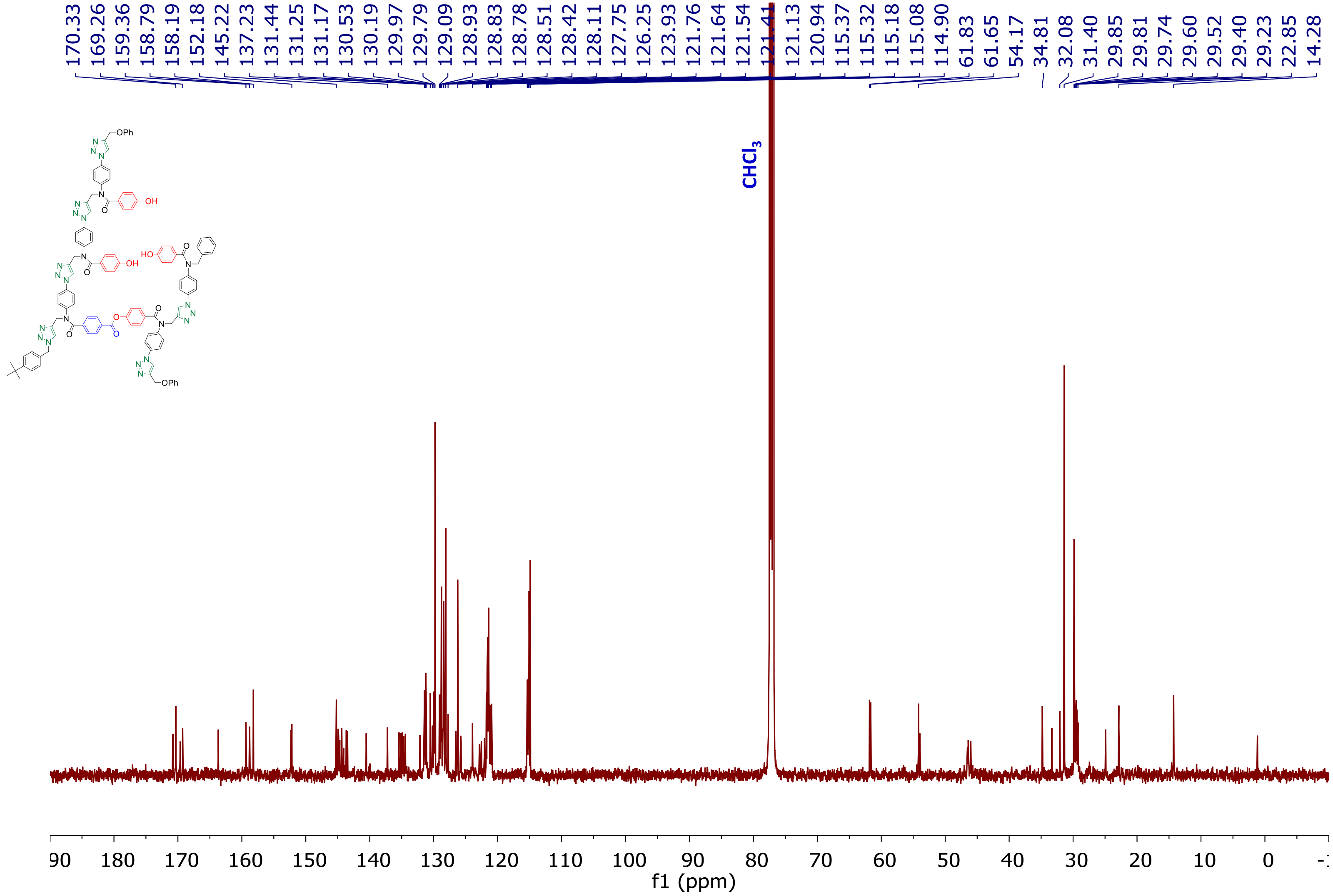

Synthesis of templated product 27 (for characterization purposes)

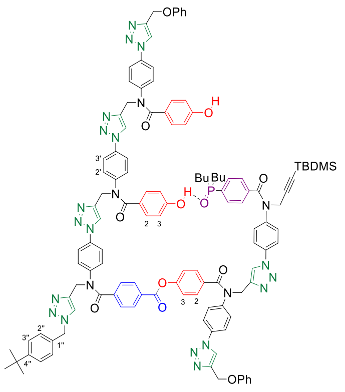

A solution of **24** (2.9 mg, 1.8·10^{-3} mmol) and **5** (1 mg, 1.8·10^{-3} mmol) in CH_2Cl_2 (2 mL) under N_2 atmosphere was treated with $Cu(CH_3CN)_4PF_6$ (2.7 mg, 7.2·10^{-3} mmol) and TBTA (3.9 mg, 7.2·10^{-3} mmol). The solution was stirred overnight at room temperature. The reaction was then diluted with EtOAc and washed with EDTA soln. (2x), H_2O (1x) and brine. The organic layer was dried over $MgSO_4$ and concentrate under vacuum. The residue was purified by flash column chromatography on silica gel (gradient from 0% to 10% of MeOH in CH_2Cl_2) to afford **27** (2 mg, 56%) as a white amorphous solid. UPLC traces shown below correspond to: (a) the starting materials; (b) the obtained reaction crude and (c) the isolated compound **27**. *UPLC Conditions*: C18 column at 40 ^{o}C (254 nm) using water + 0.1% formic acid (A) and CH_3CN + 0.1% formic acid (B) with flow rate of 0.6 ml/min; Gradient of 0-4 min 5% -100% B + 1 min 100% B.

(a) Starting materials

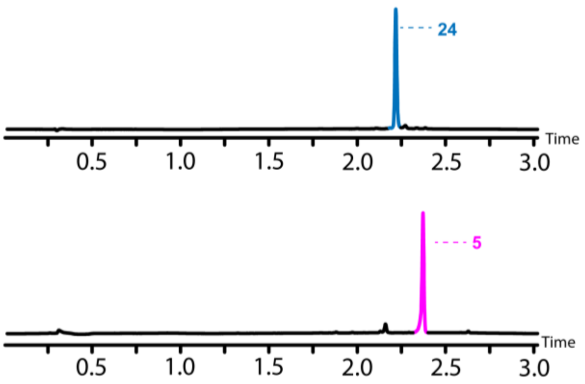

(b) Reaction crude

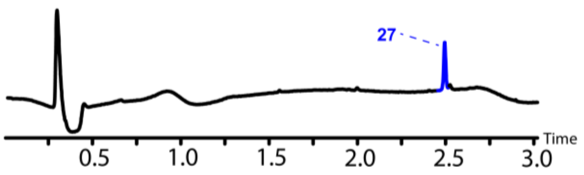

(c) Compound 27 (isolated)

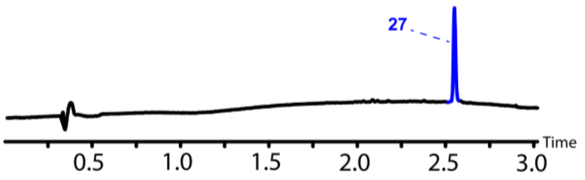

Characterization of compound 27

^{1}H NMR (500 MHz, CDCl$_3$): δ_H = 10.0 (s, 1H, OH), 8.92 (s, 1H, OH), 8.30 (s, 1H, CH_{triaz}), 8.30 (s, 1H, CH_{triaz}), 8.22 (s, 1H, CH_{triaz}), 8.00 (s, 1H, CH_{triaz}), 7.98 (s, 1H, CH_{triaz}), 7.89 (d, 2H, *J* = 8.0 Hz, 3-H, ester), 7.67 (m, 4H, 3'-H), 7.66 (s, 1H, CH_{triaz}, ^{t}Bu cap), 7.62 (d, 2H, *J* = 8.5 Hz, 3'-H), 7.59 (d, 2H, *J* = 8.5 Hz, 3'-H), 7.47 (m, 4H, 3-H, PO; 3'-H), 7.42 (m, 2H, 2-H, PO), 7.38 (d, 2H, *J* = 8.0 Hz, 3''-H, ^{t}Bu cap), 7.34 (d, 2H, *J* = 8.5 Hz, 2-H, ester), 7.30 (m, 14H, 2-H, primer; 2'-H; 3''-H, PhO caps), 7.20 (d, 2H, *J* = 8.5 Hz, 2''-H, ^{t}Bu cap), 7.19 (d, 2H, *J* = 8.5 Hz, 2'-H), 7.11 (d, 2H, *J* = 8.5 Hz, 2-H, phenol), 7.07 (d, 2H, *J* = 8.5 Hz, 2-H, phenol), 7.01 (m, 6H, 3-H, primer; 2''-H, PhO caps), 6.97 (t, 2H, *J* = 8.0 Hz, 4''-H, PhO caps), 6.58 (d, 2H, *J* = 8.0 Hz, 3-H, phenol), 6.57 (d, 2H, *J* = 8.0 Hz, 3-H, phenol), 5.48 (s, 2H, N-CH_2, ^{t}Bu cap), 5.32 (s, 2H, O-CH_2, PhO cap), 5.28 (s, 2H, O-CH_2, PhO cap), 5.19 (s, 4H, N-CH_2), 5.16 (s, 2H, N-CH_2), 5.13 (s, 2H, N-CH_2), 4.78 (s, 2H, N-CH_2, alkyne), 1.88 (m, 2H, 1''-H, Bu), 1.75 (m, 2H, 1''-H, Bu), 1.41 (m, 2H, 2''-H, Bu), 1.30 (s, 9H, ^{t}Bu), 1.23 (m, 6H, 2''-H and 3''-H, Bu), 0.84 (s, 9H, ^{t}Bu, TBDMS), 0.72 (t, 6H, *J* = 6.9 Hz, 4''-H, Bu), -0.05 (s, 6H, CH3, TBDMS)

^{13}C NMR (125.7 MHz, CDCl$_3$): δ_C = 170.2, 170.1, 169.8, 169.1 and 169.1 (CO, amide), 163.6 (CO, ester), 159.9 and, 159.3 (4-C, phenol), 158.3 and 158.3 (1''-C, PhO caps), 152.2 and 152.1 (4-C, primer; 4''-C, ^{t}Bu cap), 145.5, 145.4, 145.3, 145.3, 145.2, 145.2, 144.5, 143.7, 143.7, 143.5 and 142.4 (1'-C; C_{triaz}), 140.5 (1-C, ester), 138.5 (d, *J* = 2.5 Hz, 1-C, PO), 135.7, 135.5, 135.3, 134.9 and 134.8 (4-C, PO; 4'-C), 132.1 (1-C, primer), 131.5 (1''-C, ^{t}Bu cap), 131.2 and 131.2 (2-C, phenol), 130.5, 130.3, 130.2, 129.9, 129.9, 129.8, 129.8, 129.5, 129.0, 129.0, 128.8, 128.3, 128.1 and 128.1 (C_{arom}), 126.2 (3''-C, ^{t}Bu cap), 126.0 and 125.3 (1-C, phenol), 123.9 (CH_{triaz}, ^{t}Bu cap), 122.3, 122.2, 122.2, 121.6, 121.6, 121.4, 121.3, 121.2, 121.0, 121.0, 120.9 and 120.8 (CH_{triaz}; 3'-C; 4''-C, PhO caps), 115.2 and 115.1 (3-C, phenol), 115.0 and 114.9 (2''-H, PhO caps), 100.3 (C-Si), 89.2 (C, alkyne), 62.1 and 62.0 (O-CH_2, PhO caps), 54.1 (N-CH_2, ^{t}Bu cap), 46.6, 46.6, 46.1 and 46.1 (N-CH_2), 34.8 (C, ^{t}Bu), 32.1, 31.4 (CH_3, ^{t}Bu), 29.1 (d, *J* = 68.5 Hz, 1''-C, Bu), 26.1 (CH_3, ^{t}Bu, TBDMS), 23.9 (d, *J* = 14.5 Hz, 2''-C, Bu), 23.4 (d, *J* = 4.0 Hz, 3''-C, Bu), 16.6 (C, ^{t}Bu, TBDMS), 13.6 (4''-H, Bu), -4.63 (CH_3, TBDMS).

^{31}P NMR (202.5 MHz, CDCl$_3$): δ_P = 45.0.

HRMS (ES+): calcd $C_{124}H_{121}N_{23}O_{12}PSi$ 2182.9066 $[M+H]^+$, found 2182.8989 $[M+H]^+$.

FT-IR (ATR): ν_{max} 2958, 2923, 2851, 1646, 1604, 1518, 1381, 1282, 1242, 1043 and 845 cm^{-1}.

^{1}H-NMR (500 MHz, $CDCl_3$) compound 27

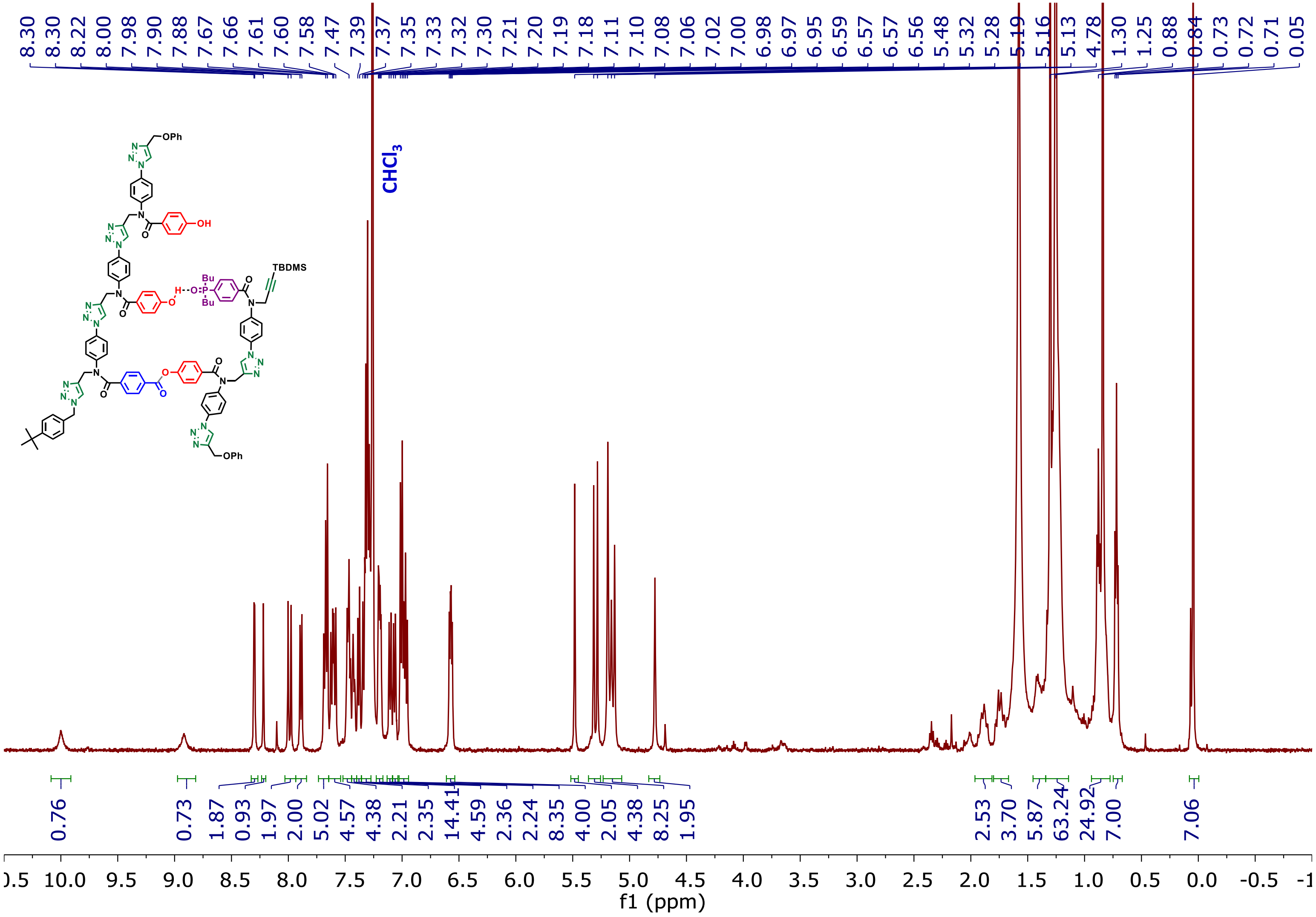

^{13}C-NMR (125.7 MHz, $CDCl_3$) compound 27

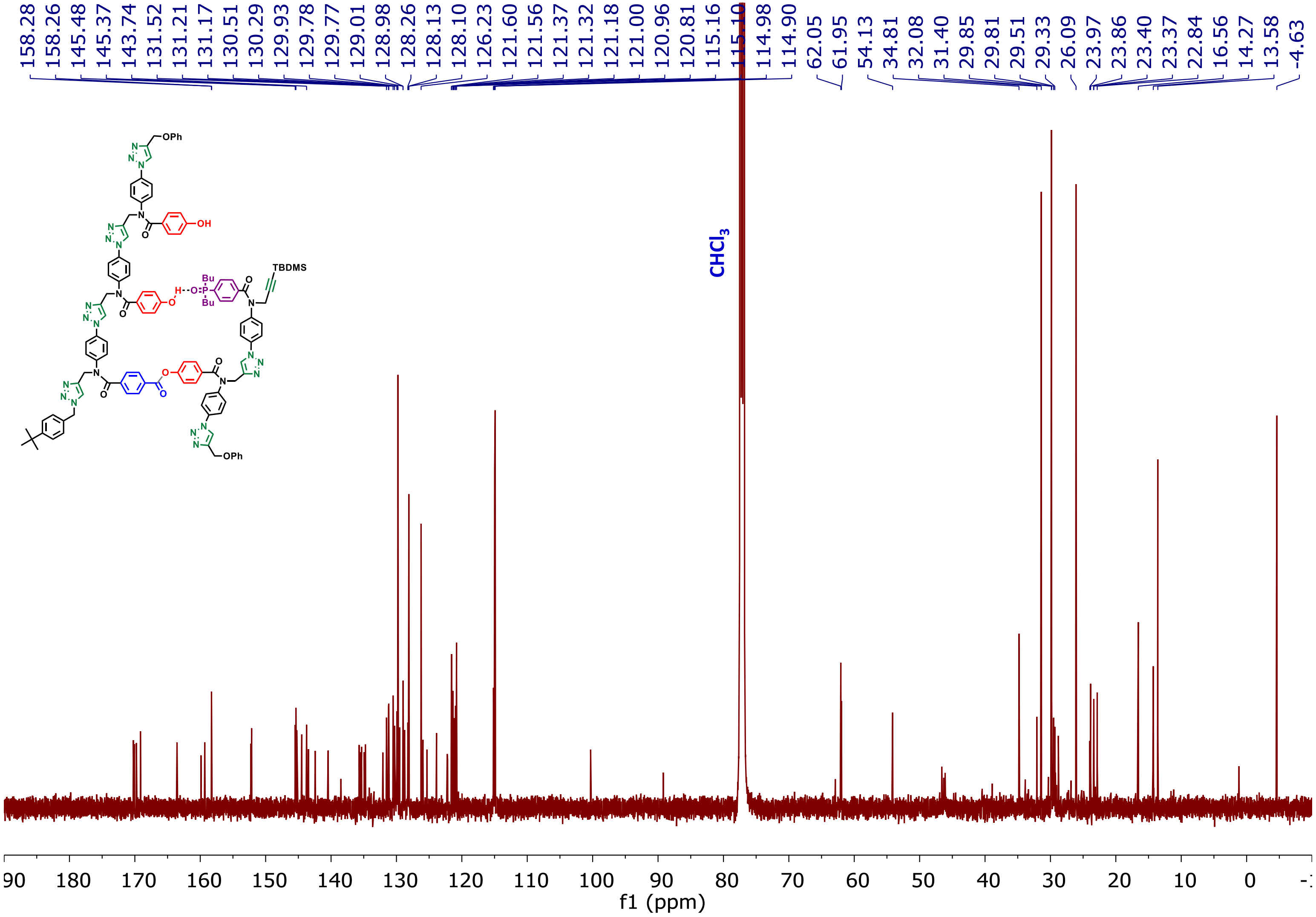

^{31}P NMR (202.5 MHz, $CDCl_3$) compound 27

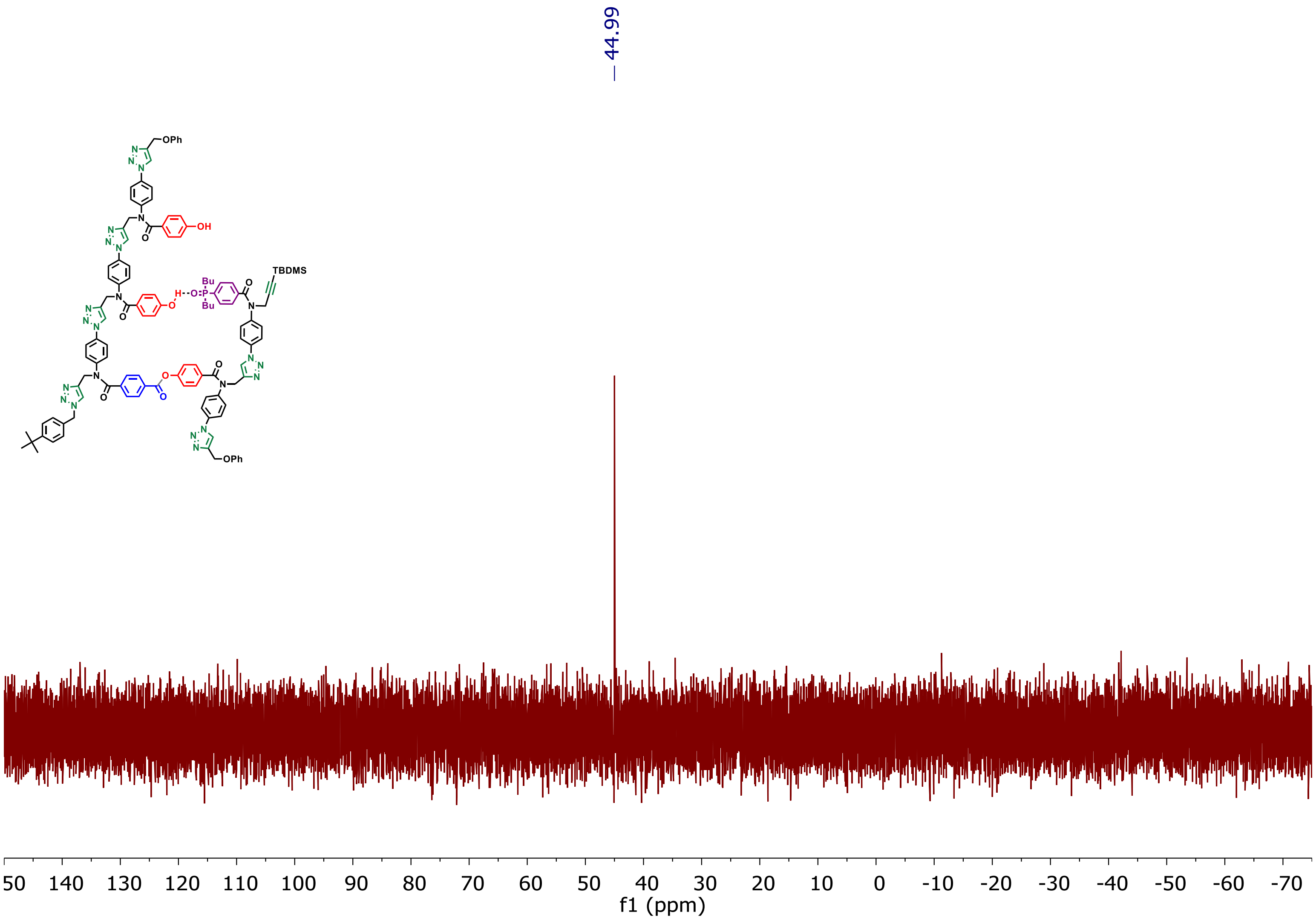

6.3. Templating reaction using 1-mer 25 and 2-mer 7

Compound **24** is used as template for the template-directed synthesis of its complementary duplex from the alkyne functionality provided by the primer. Two different azides are used: **7** is the complementary phosphine oxide 2-mer while **25** is the non-complementary one. CuAAC reaction with **24** leads to the formation of two products: **26** is the non-templated product from the reaction of **24** and **25** while **28** is the templated product from the reaction of **24** and **7** (Scheme S8). In the same way as for the 1-mers, the template effect provided by H-bonding between the phenol groups in the template and the phosphine oxides of the 2-mer is studied using the simple alkyne **S6** as control (Scheme S9).

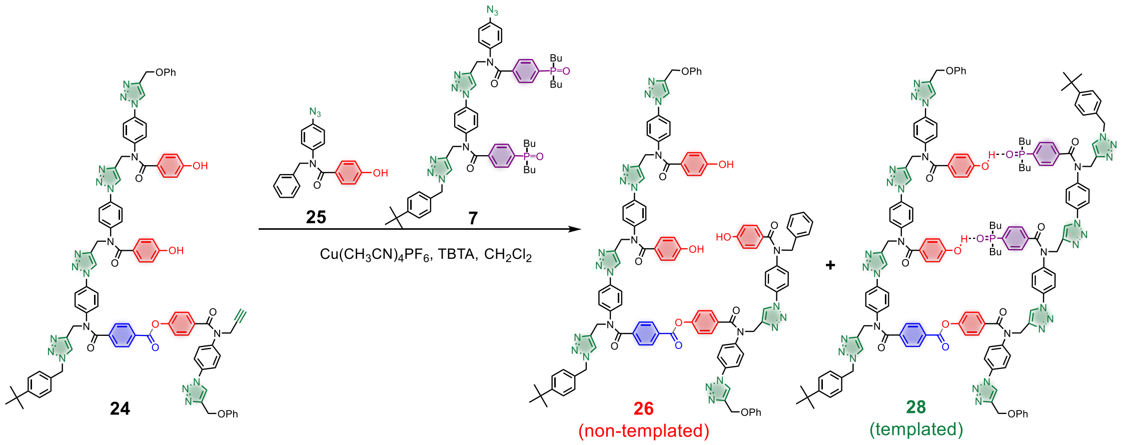

Scheme S8. Reaction schemes for the template-directed synthesis of the sequence complementary duplex from **24** using 1-mer **25** and 2-mer **7**.

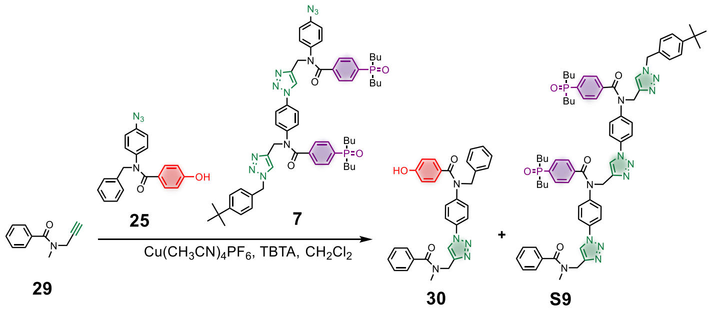

Scheme S9. Control reaction where no template effect is possible.

General procedure for the templating and control reactions shown in schemes S8 and S9.

From freshly prepared stock solutions in dry CH_2Cl_2, the starting alkyne (**24** or **29**, $3.4 \cdot 10^{-5}$ mmol), **25** (0.017 mg, $5.1 \cdot 10^{-5}$ mmol) and **7** (0.054 mg, $5.1 \cdot 10^{-5}$ mmol) were mixed in a 1.75 mL vial containing a magnetic stirrer. The solvent was evaporated under N_2 stream and dry CH_2Cl_2 (0.34 mL) was added. A 30 µL aliquot was taken for UPLC analysis (t_0). To this solution, a premixed solution of $Cu(CH_3CN)_4PF_6$ (0.022 mg, $6.8 \cdot 10^{-5}$ mmol) and TBTA (0.032 mg, $6.8 \cdot 10^{-5}$ mmol) in dry CH_2Cl_2 (10 µL) was added. The vial was flushed briefly with N_2, sealed and left stirring at room temperature for 2 days. Another 30 µL aliquot was taken after this time for UPLC analysis (t_f). Figure S15 shows the UPLC traces for the templating and control experiments, corresponding to three repetitions of the experiment. Table S2 includes the peak

areas from these chromatograms for phenol **25** and phosphine oxide **7** before (t_0) and after (t_f) Cu-TBTA was added along with the calculated *k'* (see previous section 6.1 for the equations to calculate *k'*).

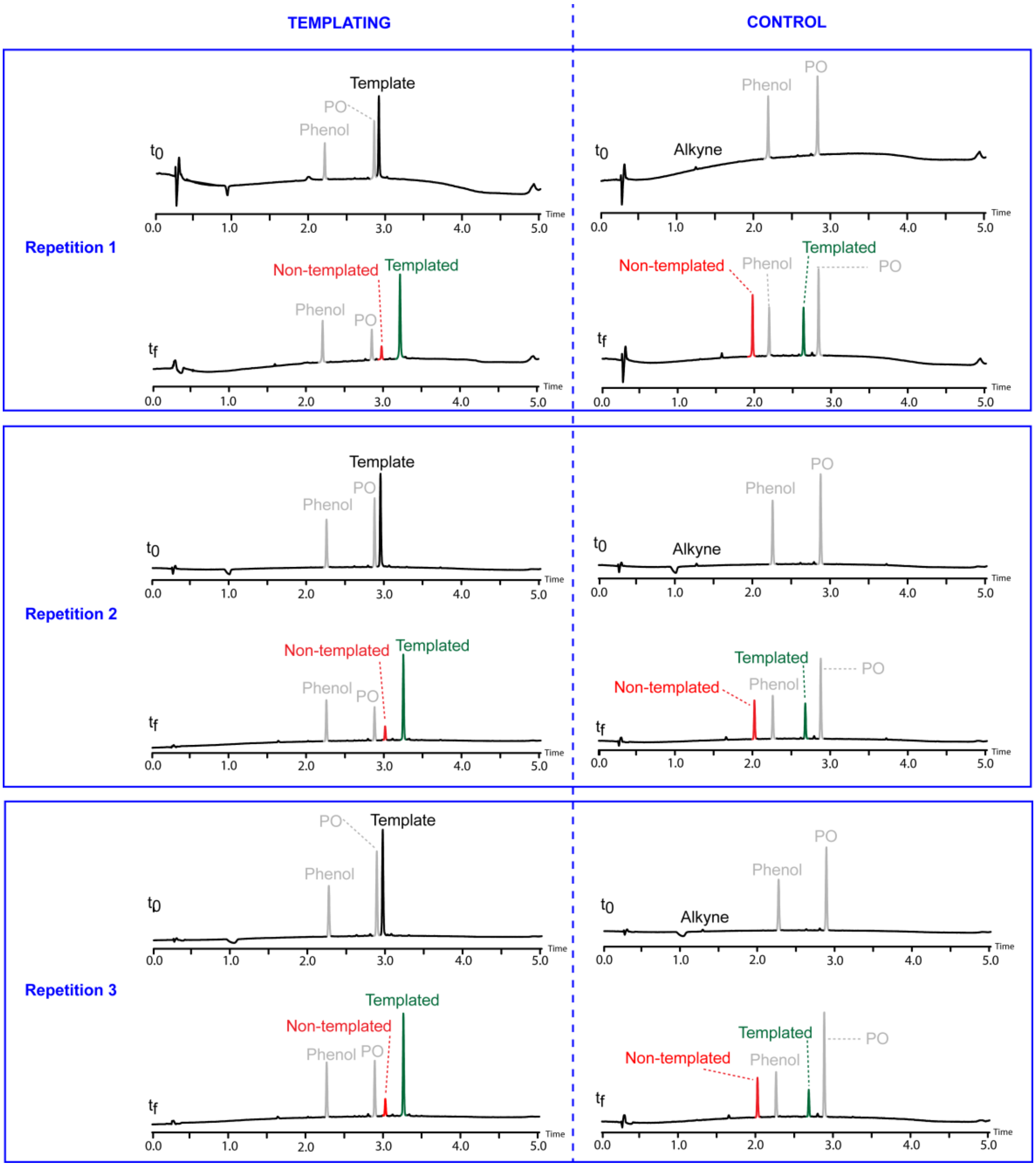

Figure S15. UPLC traces for three repetitions of the templating (left, scheme S8) and control (right, scheme S9) reactions before (t_0) and after (t_f) Cu-TBTA was added. All the peaks are labelled: Phenol (compound **25**), PO (compound **7**), template (compound **24**), alkyne (compound **S6**), non-templated (compound **26** for the templating reactions on the left; compound **S7** for the control reactions on the right) and templated (compound **28** for the templating reactions on the left; compound **S9** for the control reactions on the right). *Conditions:* C18 column at 40 °C (254 nm) using water + 0.1% formic acid (A) and CH_3CN + 0.1% formic acid (B) with flow rate of 0.6 ml/min; Gradient of 0-4 min 5% -100% B + 1 min 100% B.

Table S2. Peak areas from the UPLC chromatograms shown in Figure S14 for phenol **25** and phosphine oxide **7** before (t_0) and after (t_f) Cu-TBTA was added along with calculated *k*’.

			Phenol 25	Phosph. ox. 7	χ	*k*	*k*’
**Repetition 1**	**Templating**	t_0	253	416	2.14	3.78	**5.61**
		t_f	596	466			
	**Control**	t_0	516	684	0.71	0.57	
		t_f	488	911			
**Repetition 2**	**Templating**	t_0	1900	2609	1.74	3.33	**4.99**
		t_f	2433	1925			
	**Control**	t_0	2057	2781	0.75	0.56	
		t_f	1358	2448			
**Repetition 3**	**Templating**	t_0	3459	5432	1.60	2.73	**5.06**
		t_f	2719	2662			
	**Control**	t_0	2947	4549	0.68	0.45	
		t_f	820	1857			
				**K’ average**			**5.22 ± 0.68**

^{31}P NMR spectra of the templated products **27** and **28** at mM concentration show a pronounced downfield shift due to the intramolecular phosphine oxide-phenol H-bond, when compared to the control **7** (Figure S16). The ^{31}P chemical shifts of **27** and **28** are in agreement with the bound chemical shift for the duplex forming homo-oligomers, listed in Table 1 of the text.

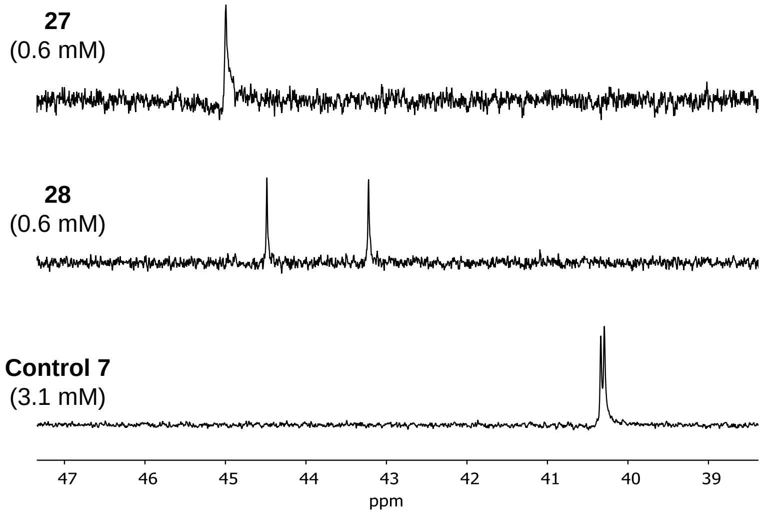

Figure S16. 500 MHz ^{31}P NMR spectra for **27** and **28** and control 2-mer phosphine oxide **7** ($CDCl_3$, 298 K).

Synthesis of templated product 28 (for characterization purposes)

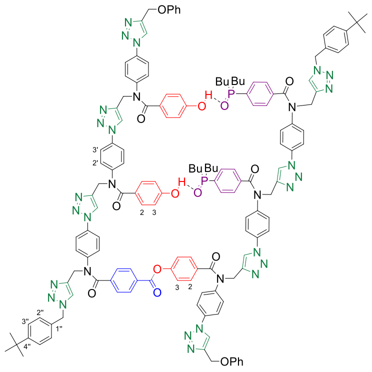

A solution of **24** (6 mg, $3.5\cdot10^{-3}$ mmol) and **7** (4 mg, $3.7\cdot10^{-3}$ mmol) in CH_2Cl_2 (2 mL) under N_2 atmosphere was treated with $Cu(CH_3CN)_4PF_6$ (0.3 mg, $7.1\cdot10^{-4}$ mmol) and TBTA (0.4 mg, $7.1\cdot10^{-4}$ mmol). The solution was stirred overnight at room temperature. The reaction was then diluted with EtOAc and washed with EDTA soln. (2x), H_2O (1x) and brine. The organic layer was dried over $MgSO_4$ and concentrate under vacuum. The residue was purified by flash column chromatography on silica gel (gradient from 0% to 10% of MeOH in CH_2Cl_2) to afford **28** (5 mg, 52%) as a white amorphous solid. UPLC traces shown below correspond to: (a) the starting materials; (b) the obtained reaction crude and (c) the isolated duplex **28**. *UPLC Conditions*: C18 column at 40 ºC (254 nm) using water + 0.1% formic acid (A) and CH_3CN + 0.1% formic acid (B) with flow rate of 0.6 ml/min; Gradient of 0-4 min 5% -100% B + 1 min 100% B.

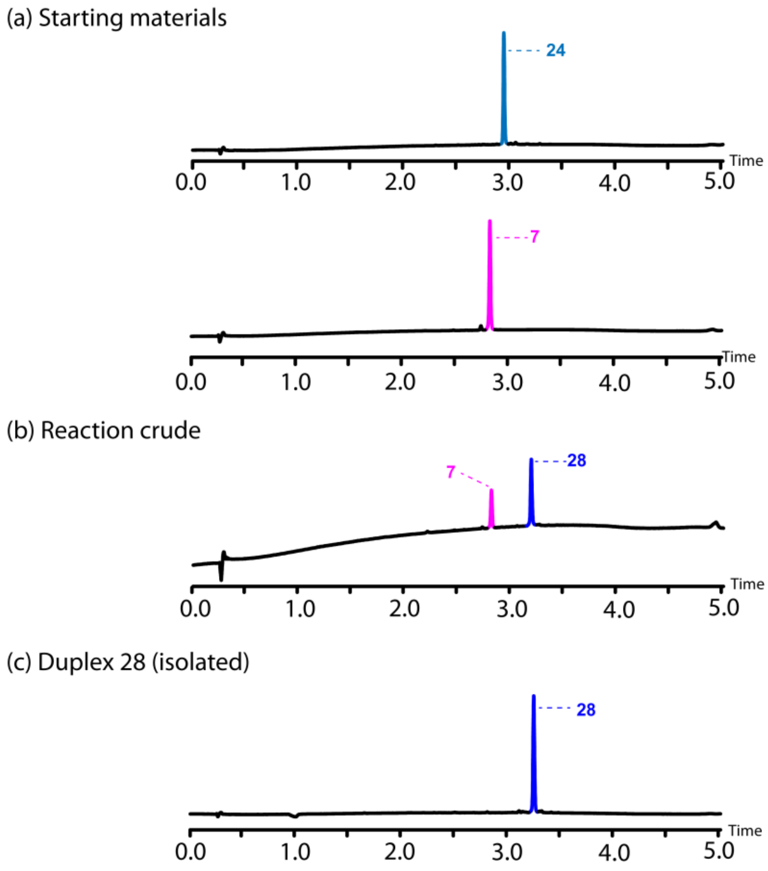

Characterization of compound 28

^{1}H NMR (500 MHz, $CDCl_3$): δ_H = 10.2 (s, 1H, OH), 9.63 (s, 1H, OH), 8.24 (s, 1H, CH_{triaz}), 8.23 (s, 1H, CH_{triaz}), 8.20 (s, 1H, CH_{triaz}), 8.18 (s, 1H, CH_{triaz}), 8.04 (s, 1H, CH_{triaz}), 8.01 (s, 1H, CH_{triaz}), 7.89 (d, 2H, *J* = 8.5 Hz, 3-H, ester), 7.66 (s, 1H, CH_{triaz}, ^{t}Bu cap), 7.66 (s, 1H, CH_{triaz}, ^{t}Bu cap), 7.60 (m, 12H, 3'-H), 7.47 (m, 4H, 3-H, PO), 7.39 (m, 8H, 2-H, PO; 3''-H, ^{t}Bu cap), 7.35-7.24 (m, 20H, 2-H, ester; 2-H, primer; 2'-H; 3''-H, PhO cap), 7.20 (d, 4H, *J* = 8.5 Hz, 2''-H, ^{t}Bu cap), 7.13 (d, 2H, *J* = 8.5 Hz, 2-H, phenol), 7.08 (d, 2H, *J* = 8.5 Hz, 2-H, phenol), 7.00 (d, 4H, *J* = 8.0 Hz, 2''-H, PhO caps), 6.98 (m, 2H, 4''-H, PhO caps), 6.96 (d, 2H, *J* = 8.5 Hz, 3-H, primer), 6.60 (d, 2H, *J* = 8.5 Hz, 3-H, phenol), 6.60 (d, 2H, *J* = 8.5 Hz, 3-H, phenol), 5.48 (s, 2H, N-CH_2, ^{t}Bu cap), 5.47 (s, 2H, N-CH_2, ^{t}Bu cap), 5.29 (s, 2H, O-CH_2, PhO cap), 5.28 (s, 2H, O-CH_2, PhO cap), 5.19 (s, 2H, N-CH_2), 5.19 (s, 2H, N-CH_2), 5.15 (s, 2H, N-CH_2), 5.13 (s, 2H, N-CH_2), 5.12 (s, 2H, N-CH_2), 1.86 (m, 4H, 1''-H, Bu), 1.72 (m, 4H, 1''-H, Bu), 1.45 (m, 4H, 2''-H, Bu), 1.30 (s, 18H, ^{t}Bu), 1.23 (m, 12H, 2''-H and 3''-H, Bu;), 0.77 (t, 6H, *J* = 7.0 Hz, 4''-H, Bu), 0.69 (t, 6H, *J* = 7.0 Hz, 4''-H, Bu).

^{13}C NMR (125.7 MHz, $CDCl_3$): δ_C = 170.3, 170.2, 169.5, 169.4, 169.3 and 169.2 (CO, amide), 163.6 (CO, ester), 160.1 and 159.9 (4-C, phenol), 158.3 and 158.3 (1''-C, PhO caps), 152.2, 152.2 and 152.1 (4-C, primer; 4''-C, ^{t}Bu caps), 145.4, 145.4, 145.3, 145.2, 145.1, 144.5, 144.3, 144.0, 143.8, 143.8, 143.7, 143.7 and 143.5 (1'-C; C_{triaz}), 140.4 (1-C, ester), 138.5 (d, *J* = 2.5 Hz, 1-C, PO), 138.4 (d, *J* = 2.5 Hz, 1-C, PO), 135.5, 135.5, 135.4, 135.3, 134.8, 134.7, 134.5, 134.1 and 133.8 (4-C, PO; 4'-C), 132.3 (1-C, primer), 131.5 and 131.5 (1''-C, ^{t}Bu caps), 131.3, 131.2, 130.5, 130.4, 130.3, 130.3, 130.3, 130.2, 130.0, 129.8, 129.2, 129.1, 129.0, 129.0, 128.9, 128.8, 128.3, 128.1 and 128.1 (C_{arom}), 126.2 and 126.2 (3''-C, ^{t}Bu caps), 125.4 and 125.1 (1-C, phenol), 123.9 and 123.9 (CH_{triaz}, ^{t}Bu cap), 122.2, 122.2 and 122.1 (CH_{triaz}), 121.6, 121.5, 121.4, 121.3, 121.3, 121.1, 121.1, 121.1 and 121.0 (3'-C; 4''-C, PhO caps), 121.0 and 120.9 (CH_{triaz}), 115.2 and 115.1 (3-C, phenol), 114.9 and 114.9 (2''-H, PhO caps), 62.0 and 62.0 (O-CH_2, PhO caps), 54.2 and 54.1 (N-CH_2, ^{t}Bu caps), 46.6, 46.5, 46.4, 46.3 and 46.1 (N-CH_2), 34.8 (C, ^{t}Bu), 31.4 (CH_3, ^{t}Bu), 29.2 (d, *J* = 68.5 Hz, 1''-C, Bu), 29.1 (d, *J* = 68.5 Hz, 1''-C, Bu), 24.0 (d, *J* = 14.0 Hz, 2''-C, Bu), 23.9 (d, *J* = 14.0 Hz, 2''-C, Bu), 23.5 (d, *J* = 4.0 Hz, 3''-C, Bu), 23.4 (d, *J* = 4.0 Hz, 3''-C, Bu), 13.6 and 13.6 (4''-C, Bu).

^{31}P NMR (161.9 MHz, $CDCl_3$): δ_P = 43.7 and 42.4.

HRMS (ES+): calcd $C_{153}H_{151}N_{30}O_{14}P_2$ 1347.5784 $[M+2H]^{2+}$, found 1347.5746 $[M+2H]^{2+}$.

FT-IR (ATR): ν_{max} 2955, 2920, 2851, 1735, 1712, 1649, 1519, 1362, 1221 and 823 cm^{-1}.

^{1}H-NMR (500 MHz, $CDCl_3$) compound 28

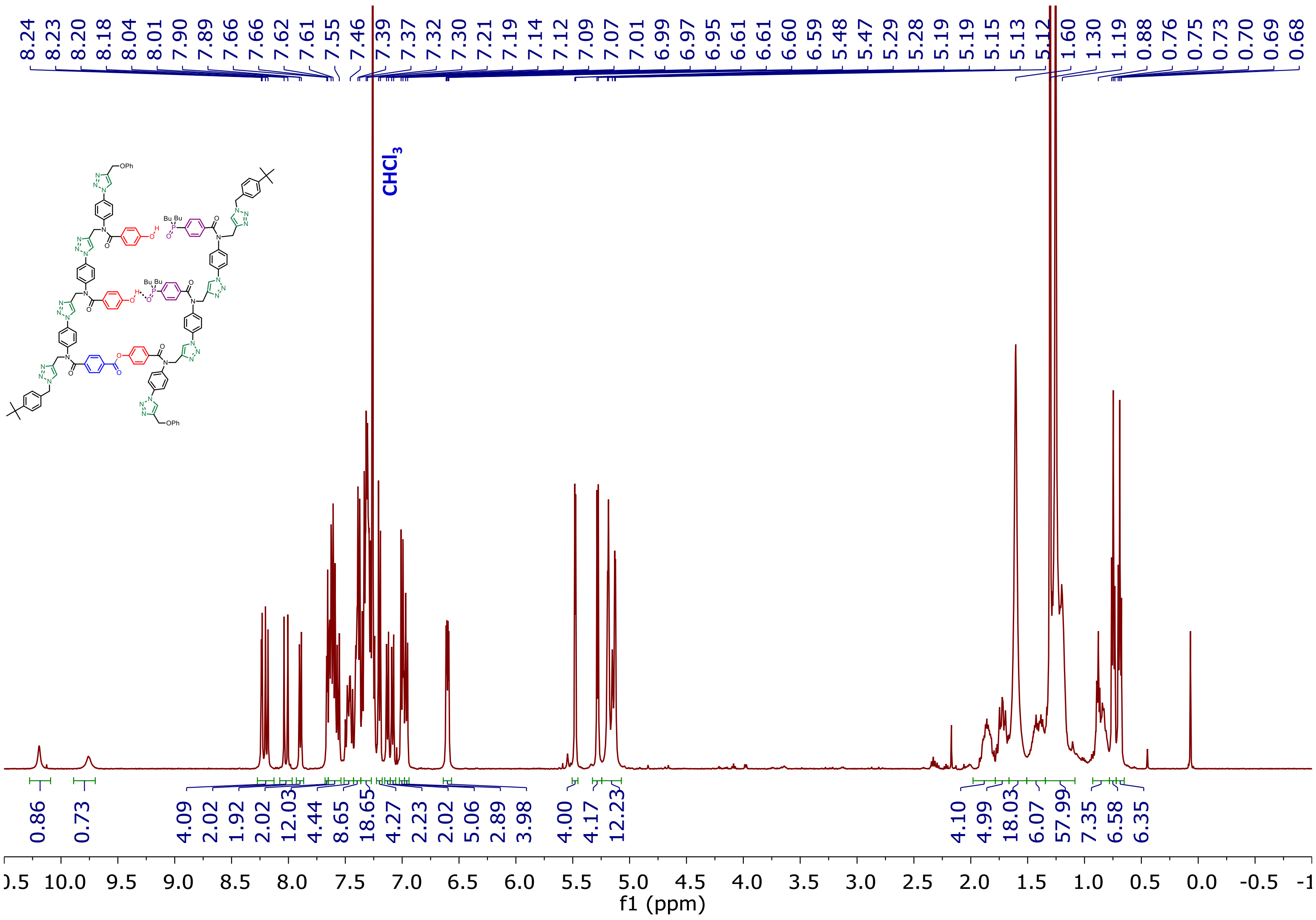

^{13}C-NMR (125.7 MHz, $CDCl_3$) compound 28

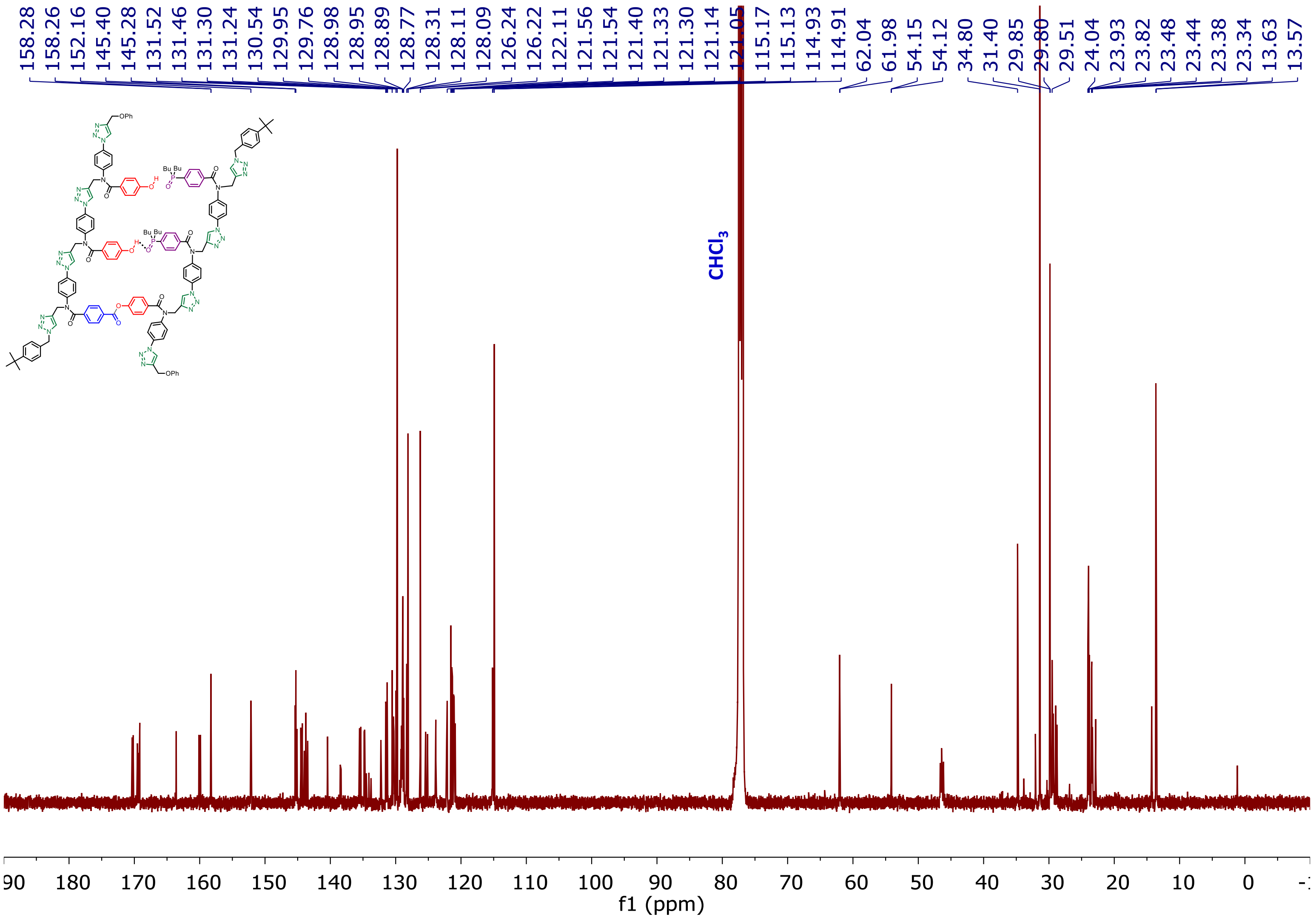

^{31}P NMR (161.9 MHz, CDCl$_3$) compound 28

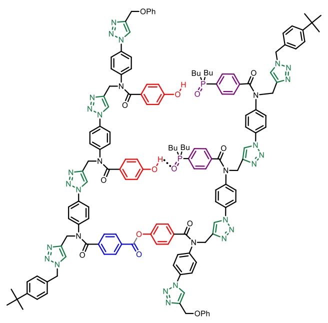

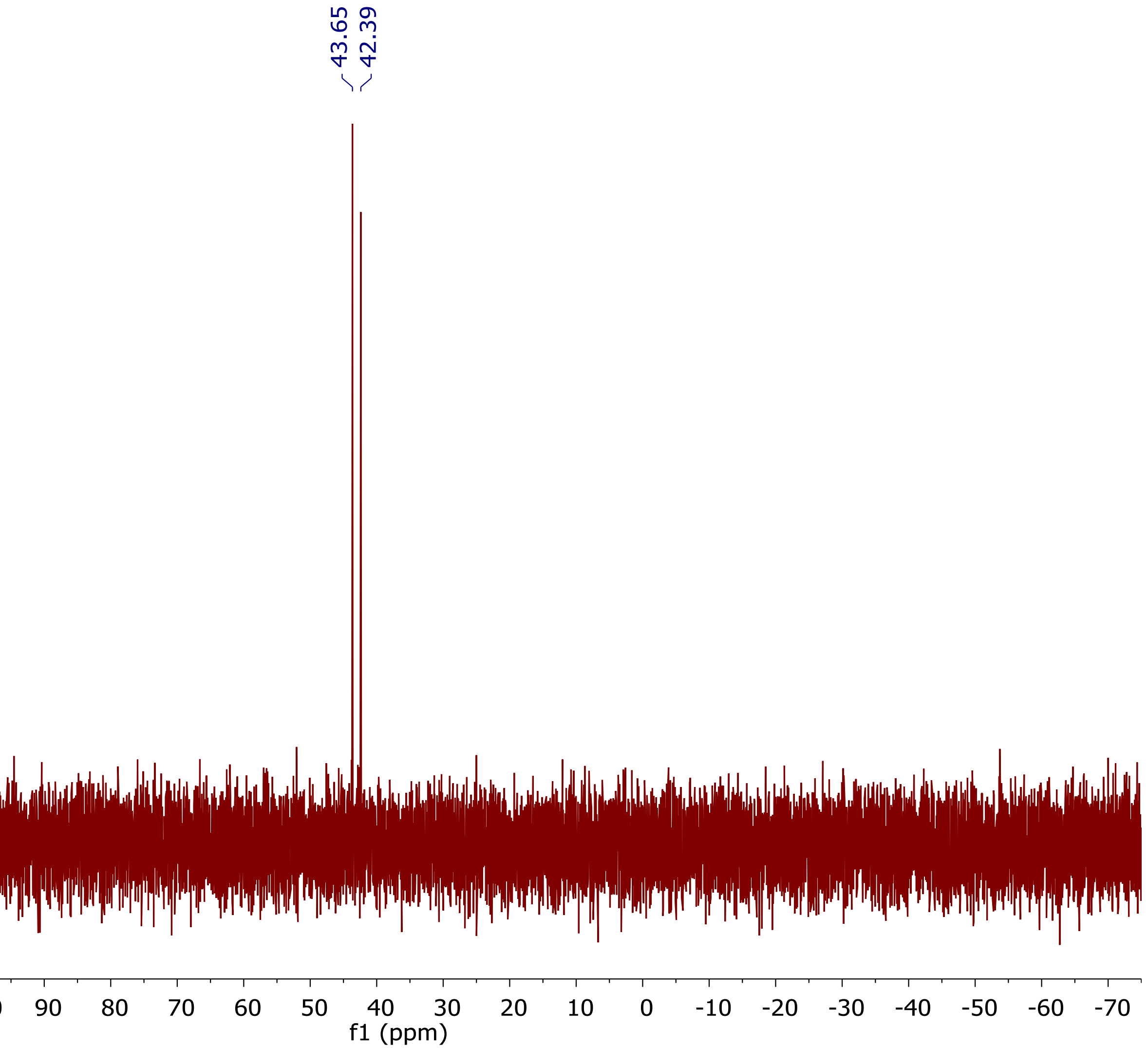

7. Hydrolysis of duplex 28 providing copy 32

Basic hydrolysis of the ester bond in duplex **28** afforded the corresponding complementary copy (**32**) along with the starting template **21**, as shown in scheme S10. Figure S17 shows the corresponding UPLC traces for the hydrolysis reaction and isolation of template and copy.

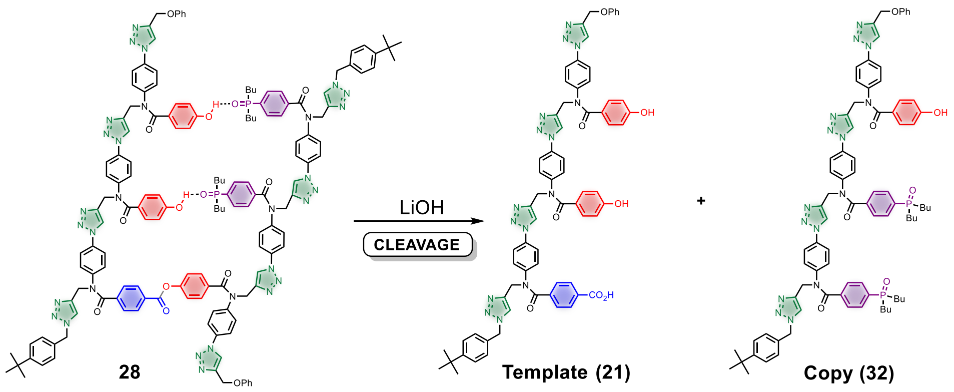

Scheme S10. Cleavage of the ester in 28 to give access to copy **32** along with template **21**.

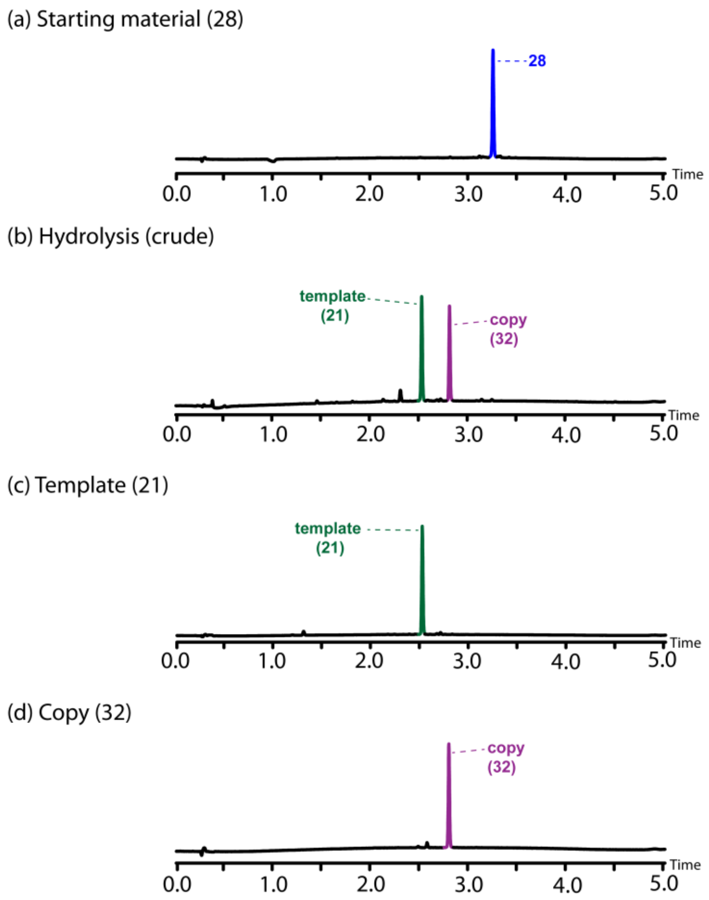

Figure S17. UPLC traces for the cleavage step: (a) the starting material; (b) the obtained reaction crude; (c) and (d) isolated template and copy strands. *UPLC Conditions*: C18 column at 40 °C (254 nm) using water + 0.1% formic acid (A) and CH_3CN + 0.1% formic acid (B) with flow rate of 0.6 ml/min; Gradient of 0-4 min 5% -100% B + 1 min 100% B.

Synthesis of compound 32

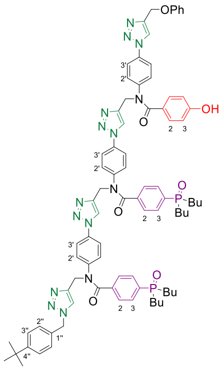

A solution of 28 (5 mg, 3.5·10-3 mmol) in THF (2 mL) and water (0.5 mL) was treated with LiOH (1M in water, 35 µL, 0.035 mmol). After 20 minutes of stirring at rt, the solution was acidified with 1M HCl aq. soln. to pH 2-3 and extracted with EtOAc (3x). The organic layer was washed with brine and dried over MgSO4. The residue was purified by flash column chromatography on silica gel (gradient from 0% to 10% of MeOH in CH2Cl2) to afford **32** (4 mg, 77%) as a white amorphous solid.

^{1}H NMR (500 MHz, $CDCl_3$): δ_H = 9.63 (s, 1H, OH), 8.12 (s, 1H, CH_{triaz}, phenol), 8.08 (s, 1H, CH_{triaz}, PO), 8.01 (s, 1H, CH_{triaz}, PO), 7.65 (s, 1H, CH_{triaz}, ^{t}Bu cap), 7.58 (m, 6H, 3'-H), 7.49 (m, 6H, 2-H and 3-H, PO), 7.38 (m, 4H, 2-H, PO; 3''-H, ^{t}Bu cap), 7.30 (m, 6H, 2'-H; 3''-H, PhO cap), 7.26 (m partially overlapped, 2H, 2'-H), 7.19 (d, 2H, *J* = 8.5 Hz, 2''-H, ^{t}Bu cap), 7.13 (d, 2H, *J* = 8.5 Hz, 2-H, phenol), 6.99 (d, 2H, *J* = 8.5 Hz, 2''-H, PhO cap), 6.97 (t, 1H, *J* = 8.5 Hz, 4''-H, PhO cap), 6.63 (d, 2H, *J* = 8.5 Hz, 3-H, phenol), 5.47 (s, 2H, N-CH_2, ^{t}Bu cap), 5.26 (s, 2H, O-CH_2, PhO cap), 5.26 (s, 2H, N-CH_2, PO), 5.19 (s, 2H, N-CH_2, phenol), 5.11 (s, 2H, N-CH_2, PO), 1.88 (m, 4H, 1''-H, Bu), 1.74 (m, 4H, 1''-H, Bu; overlapped by water peak), 1.45 (m, 4H, 2''-H, Bu), 1.30 (s, 9H, ^{t}Bu), 1.25 (m, 12H, 2''-H and 3''-H, Bu;), 0.76 (t, 6H, *J* = 7.0 Hz, 4''-H, Bu), 0.76 (t, 6H, *J* = 7.0 Hz, 4''-H, Bu).

^{13}C NMR (125.7 MHz, $CDCl_3$): δ_C = 170.5 (CO, phenol), 169.6 and 169.2 (CO, PO), 160.0 (4-C, phenol), 158.3 (1''-C, PhO cap), 152.1 (4''-C, ^{t}Bu cap), 145.3 and 145.3 (C_{triaz}), 145.1 (1'-C), 144.3 (C_{triaz}, PO), 143.8 and 143.7 (C_{triaz}, ^{t}Bu cap; 1'-C), 143.1 (1'-C), 138.4 (d, *J* = 2.5 Hz, 1-C, PO), 138.1 (d, *J* = 2.5 Hz, 1-C, PO), 135.5 and 135.3 (4'-C), 134.9 (d, *J* = 89.0 Hz, 4-C, PO), 134.7 (4'-C), 134.7 (d, *J* = 90.0 Hz, 4-C, PO), 131.5 (1''-C, ^{t}Bu cap), 130.3 (d, *J* = 9.0 Hz, 3-C, PO), 130.3 (d, *J* = 9.0 Hz, 3-C, PO), 129.7 (3''-C, PhO cap), 129.2, 129.1, 129.0, 128.9, 128.8, 128.1 and 128.1 (C_{arom}), 126.2 (3''-C, ^{t}Bu cap), 125.3 (1-C, phenol), 123.9 (CH_{triaz}, ^{t}Bu cap), 121.9 (CH_{triaz}, phenol), 121.6 (CH_{triaz}, PO), 121.5 (4''-C, PhO cap), 121.3, 121.2 and 121.2 (3'-C), 121.0 (CH_{triaz}, PO), 115.3 (3-C, phenol), 114.9 (2''-H, PhO cap), 62.0 (O-CH_2, PhO cap), 54.1 (N-CH_2, ^{t}Bu cap), 46.6, 46.5 and 46.0 (N-CH_2), 34.8 (C, ^{t}Bu), 31.4 (CH_3, ^{t}Bu), 29.3 (d, *J* = 68.5 Hz, 1''-C, Bu), 29.3 (d, *J* = 68.5 Hz, 1''-C, Bu), 24.0 (d, *J* = 14.5 Hz, 2''-C, Bu), 23.5 (d, *J* = 4.0 Hz, 3''-C, Bu), 23.5 (d, *J* = 4.0 Hz, 3''-C, Bu), 13.6 and 13.6 (4''-C, Bu).

^{31}P NMR (161.9 MHz, $CDCl_3$): δ_P = 42.0 and 41.7.

HRMS (ES+): calcd $C_{84}H_{94}N_{15}O_7P_2$ 1486.6930 $[M+H]^+$, found 1486.6910 $[M+H]^+$.

FT-IR (ATR): ν_{max} 2957, 2927, 2869, 2855, 1645, 1518, 1366, 1281, 1167, 845 and 754 cm^{-1}.

^{1}H-NMR (500 MHz, $CDCl_3$) compound 32

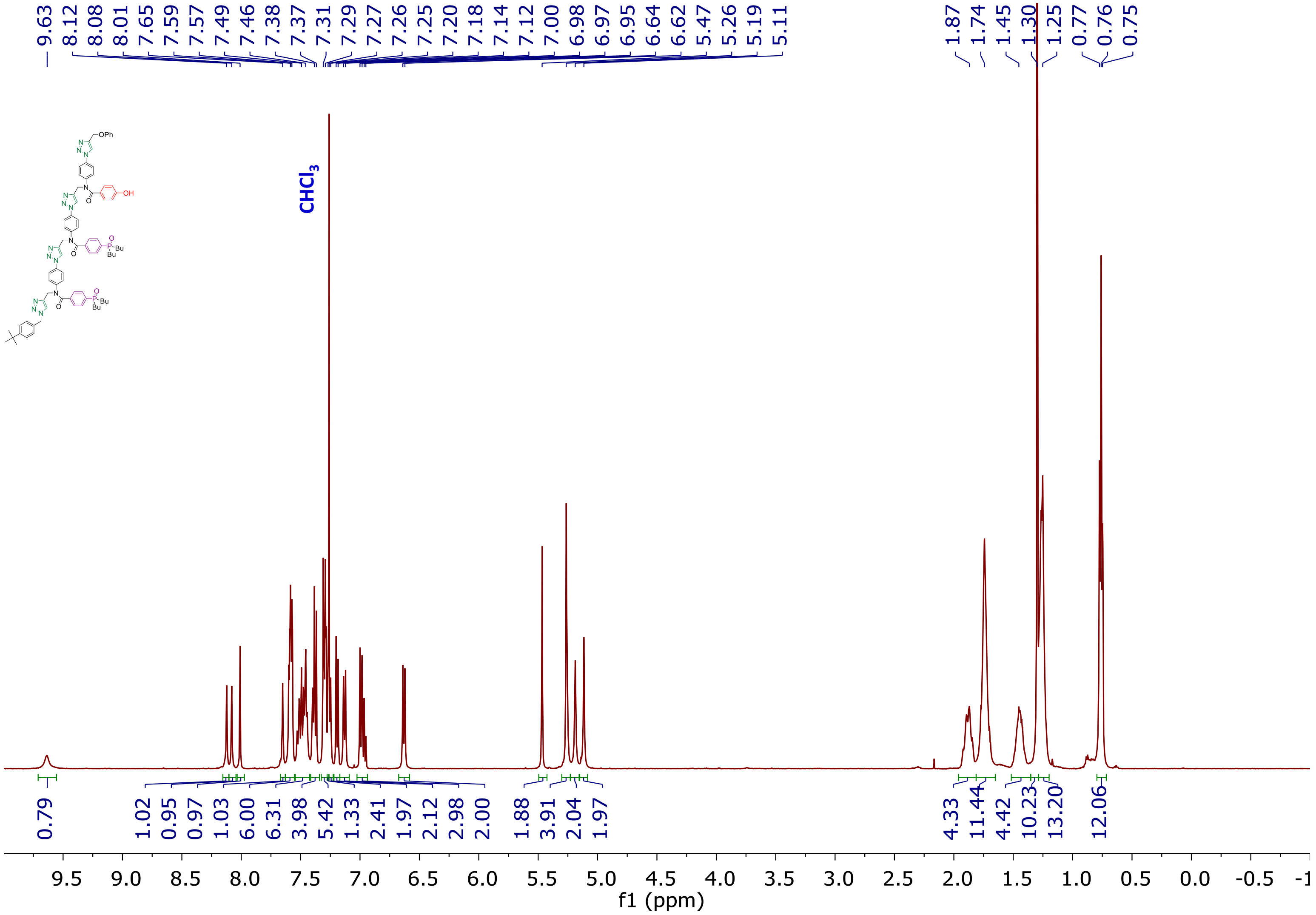

^{13}C-NMR (125.7 MHz, CDCl$_3$) compound 32

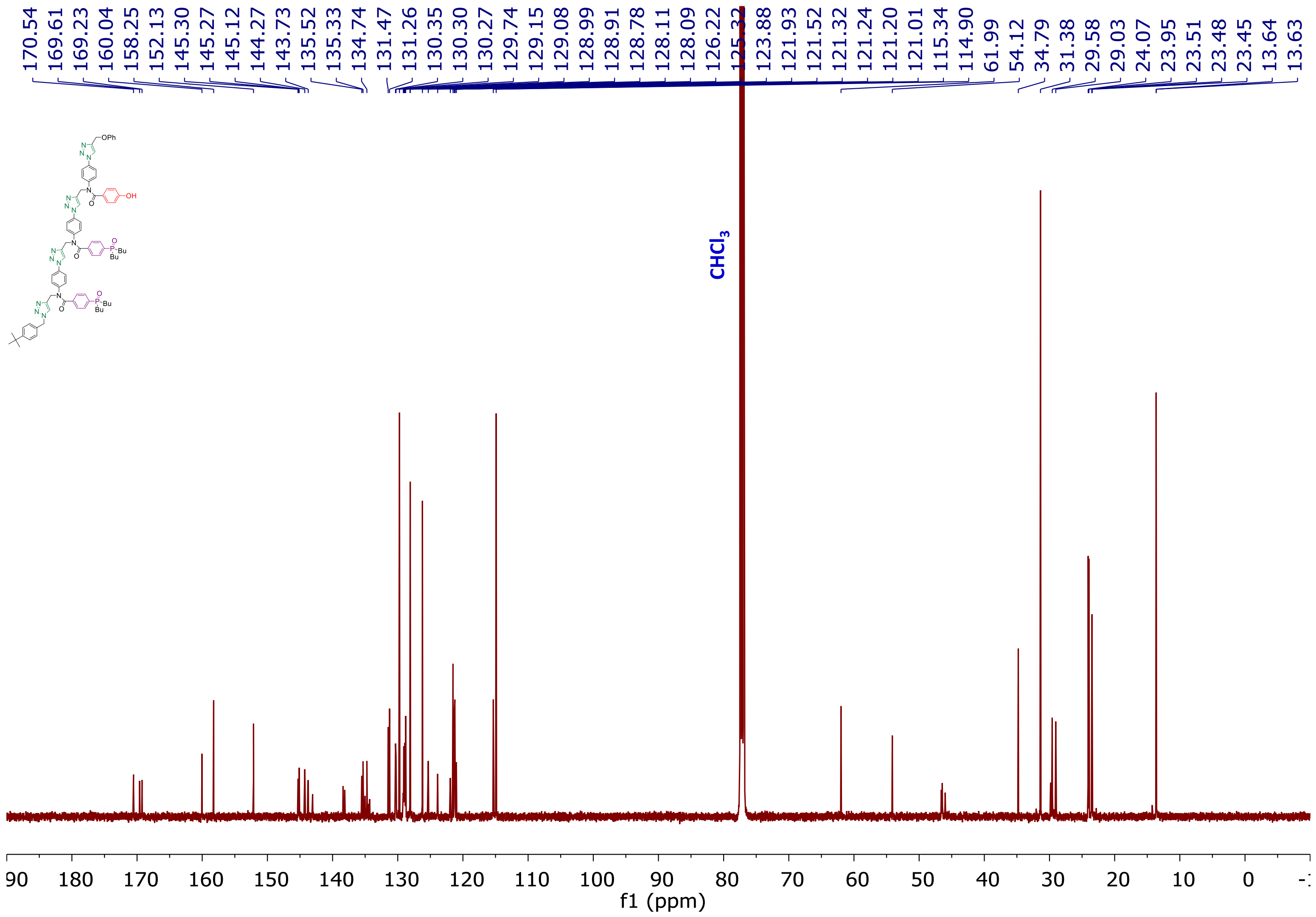

^{31}P NMR (161.9 MHz, $CDCl_3$) compound 29

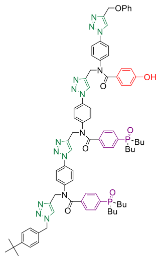

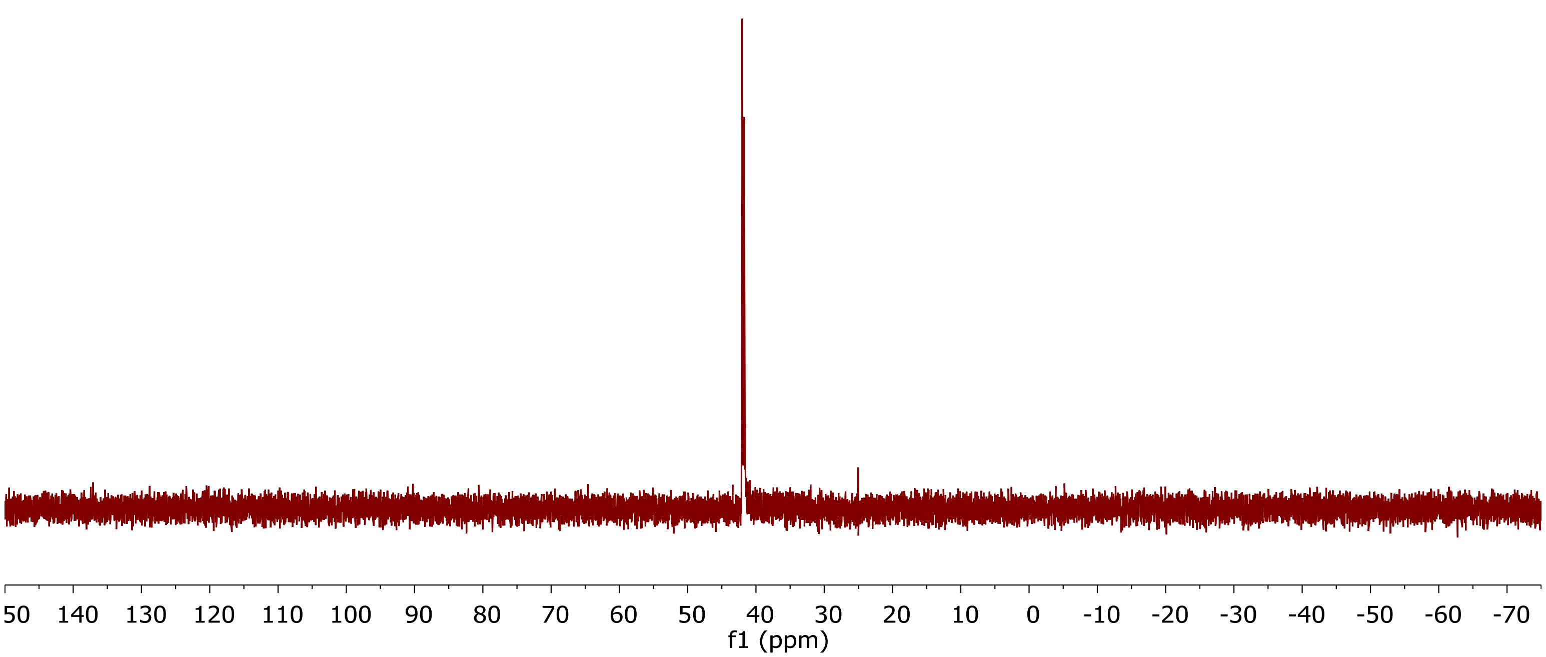

8. References

[S1] Núñez-Villanueva, D.; Ciaccia, M.; Iadevaia, G.; Sanna, E.; Hunter, C. A. Sequence information transfer using covalent template-directed synthesis. *Chem. Sci*. **2019**, *10*, 5258.

[S2] Schrödinger Release 2019-1: MacroModel, Schrödinger, LLC, New York, NY, 2019.

[S3] The PyMOL Molecular Graphics System (open-source PyMOL). Version 2.3.0a0. Schrödinger, LLC.

[S4] Swain, J. A.; Iadevaia, G.; Hunter, C. A. H-Bonded duplexes based on a phenylacetylene backbone. *J. Am. Chem. Soc.* **2018**, *140*, 11526.

[S5] Iadevaia, G.; Swain, J. A.; Núñez-Villanueva, D.; Bond, A. D.; Hunter, C. A. Folding and duplex formation in mixed sequence recognition-encoded m-phenylene ethynylene polymers. *Chem. Sci.* **2021**, *12*, 10218.
